# Intra‐Tissue Bacteriome and Cellular Profiles in Periodontal Granulation Tissue From Osseous Defects and Extraction Sockets

**DOI:** 10.1111/jcpe.70108

**Published:** 2026-02-24

**Authors:** Tianfan Cheng, Tianle Li, Tsz Yung Wong, Beibei Chen, Chongshan Liao, Xun Ding, Hui Chen, Wei Qiao, George Pelekos, Lijian Jin

**Affiliations:** ^1^ Division of Periodontology & Implant Dentistry, Faculty of Dentistry The University of Hong Kong Hong Kong SAR China; ^2^ Division of Restorative Dental Sciences, Faculty of Dentistry The University of Hong Kong Hong Kong SAR China; ^3^ Division of Applied Oral Sciences & Community Dental Care, Faculty of Dentistry The University of Hong Kong Hong Kong SAR China; ^4^ Department of Orthodontics Stomatological Hospital and Dental School of Tongji University Shanghai China

**Keywords:** bacteriome, granulation tissue, periodontal defect, periodontitis, single‐cell RNA‐sequencing

## Abstract

**Aim:**

To investigate the intra‐tissue bacteriome and cell profiles within periodontal granulation tissue (PGT) for exploring the biological essence and translational potentials.

**Materials and Methods:**

PGT samples were collected from 49 patients with severe periodontitis—including those from osseous defects (GT) during periodontal surgery, extraction sockets (ST) and extracted root surfaces (RT) during tooth extraction—while the excised pocket wall (PT) from surgical sites served as the control for diseased tissues. These samples underwent 16S rRNA sequencing, single‐cell sequencing and histological assessment.

**Results:**

GT and PT exhibited periodontal health–associated, commensal‐enriched bacteriome profiles, while RT and ST showed worse local periodontal condition, enriched periodontopathogens but depleted commensals. Notably, GT contained a higher proportion of mesenchymal stem cells (MSCs) and fibroblasts while fewer natural killer cells than in PT and ST. Pseudotime trajectory analysis revealed endothelial and epithelial differentiation fates from mesenchymal progenitors across tissues. Moreover, there were less inflammatory infiltration and immunoreactivity of CD4^+^ and NKG2D^+^ in GT than in PT and ST.

**Conclusions:**

Our findings suggest potential periodontal health–associated features of GT regarding clinical, bacteriome and cellular attributes. Future translational study is warranted to explore GT as an alternative source of MSCs for periodontal regeneration.

## 1 Introduction

Periodontitis is one of the major diseases with huge socio‐economic burdens (Nascimento et al. 2024), causing severe tooth loss and edentulism in adults and closely connected with common systemic comorbidities (Hajishengallis and Chavakis 2021).

Notably, the crucial periodontal ‘granulation tissue’ (PGT) within defects is routinely discarded during periodontal surgical procedures (Papapanou et al. 1989; Ribeiro et al. 2023) to access periodontal niches and promote healing (Bartold and Ivanovski 2025), despite its limited clinical significance (Lindhe and Nyman 1985). During wound healing, PGT transitions to a provisional matrix and woven bone, similarly as in tooth extraction (Trombelli et al. 2008) and periodontal surgery (Susin et al. 2015).

PGT is a highly vascularised cellular conglomerate and fibrin‐rich matrix, supporting periodontal wound healing (Sculean et al. 2014). However, there could arise persistent, uncontrolled infections and inflammation due to both extra‐ and intra‐cellular pathogens (Cheng et al. 2019; Ji and Choi 2020; Rajakaruna et al. 2018) and dysregulated host responses.

PGT shows increased CD8^+^ T lymphocytes and macrophages (Canullo et al. 2020; Kayar et al. 2020) but may also contain fibroblasts and mesenchymal stem cells (MSCs) (Adam et al. 2020; Apatzidou et al. 2018; Gousopoulou et al. 2023; Ronay et al. 2014), implying improvements in wound healing and periodontal regeneration strategies (Bartold and Ivanovski 2025).

Gingiva‐ and alveolar bone‐marrow‐derived MSCs (a‐BMMSCs) exhibit potential for periodontal reconstruction (Apatzidou et al. 2021; Apatzidou et al. 2024; Ge et al. 2012). Recent trials have explored PGT preservation (Adam et al. 2022; Moreno Rodriguez and Ortiz Ruiz 2022). Despite extensive omic studies on periodontal tissue (Caetano et al. 2021; Chen et al. 2022; Williams et al. 2021), omics data on PGT from different topographies and sources remain limited (Sam et al. 2025). Considering the pros and cons concerned, utilising advanced omics approaches is essential to address the underlying omics over whether to discard or not to discard PGT (Sam et al. 2024).

In this study, we arbitrarily classified three forms of PGT from different oral niches for collection and performed 16S rRNA sequencing (RNA‐seq), single‐cell RNA sequencing (scRNA‐seq) and histology, with inflamed gingival tissue as the reference of non‐granulation diseased tissue, to identify and explore the underlying intra‐tissue bacterial and cellular attributes of PGT, fate of tissues and treatment endpoints for future translational studies.

## 2 Materials and Methods

### 2.1 Subjects Recruitment

Participants with periodontitis (Stages III–IV) were recruited at the Prince Philip Dental Hospital and the Institute for Advanced Dentistry Multi‐Specialty Clinic of the University of Hong Kong (HKU) during January 2022 to March 2023. The study protocols were approved by the Institutional Review Board of HKU/Hospital Authority of Hong Kong West Cluster (IRB UW 21‐596) and followed the current Declaration of Helsinki. This study followed STROBE guidelines.

Patients scheduled for periodontal surgeries and/or extraction of periodontally hopeless teeth were enrolled without additional clinical procedure. Study details and questionnaires (Supporting Information 2.1) were provided, and informed consents were obtained. Inclusion and exclusion criteria are listed in Supporting Information 2.2.

### 2.2 Periodontal Examination

Periodontal charting was performed, and a diagnosis was made following the current classification (Tonetti et al. 2018). Full‐mouth periodontal charting recorded bleeding on probing (BOP), probing depth (PD), gingival recession (REC) and clinical attachment loss (CAL) at six sites using a UNC‐15 periodontal probe. Mobility (M), furcation involvement (FI), fremitus, full‐mouth plaque score (FMPS) and full‐mouth bleeding score (FMBS) were also assessed. The periodontal inflamed surface area (PISA) was calculated to quantify inflamed periodontal tissue and assess the extent of inflammation (Miki et al. 2021). Clinical data were collected by the respective dentist in charge, and radiographs were taken as part of the routine examination (Supporting Information 2.3).

### 2.3 Periodontal Therapy

Periodontal treatment was performed, including non‐surgical periodontal therapy (NSPT) and surgical therapy, without changing the normal treatment protocol. NSPT was performed by the same dentist, according to steps 1 and 2 therapy (Herrera et al. 2022; Sanz et al. 2020). Periodontally involved teeth with hopeless prognosis (360° bone loss to or beyond apex and negative/inconclusive pulp test) (Cortellini et al. 2011) were extracted before NSPT per operator's treatment plan. For delayed extractions post surgery, asymptomatic teeth were retained per patient preference, with NSPT attempts (Supporting Information 2.4).

### 2.4 Collection of Tissue Samples

**FIGURE 1:**
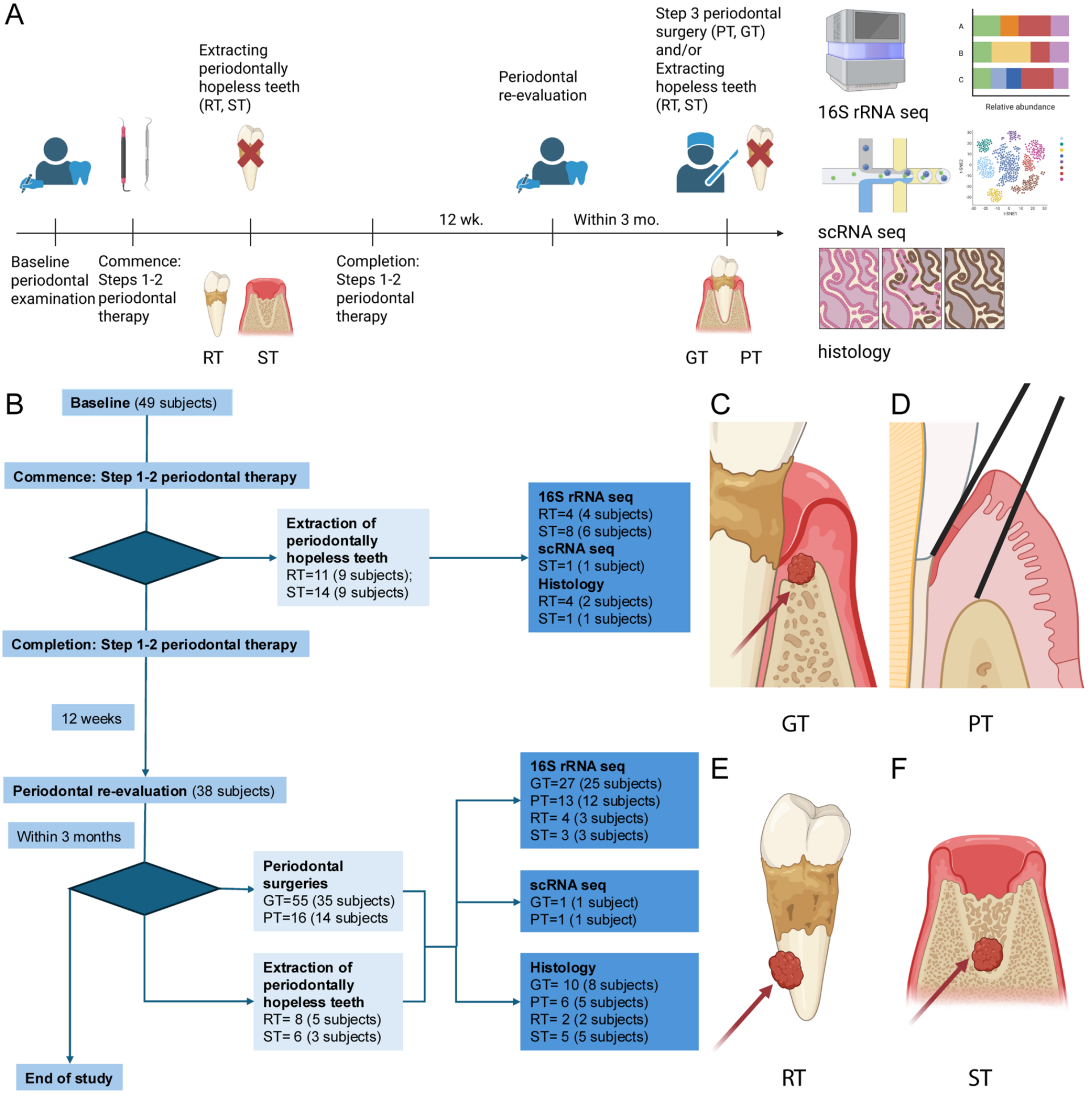
Study design. (A) Flow chart of recruitment of participants, timing of treatment, collection of tissue samples and downstream analysis, including 16S rRNA amplicon sequencing, single‐cell RNA sequencing and histology. (B) Flow chart of the study. Sampled tissues and subjects are labelled. Tissues used in 16S rRNA‐seq, scRNA‐seq and histology are also labelled. (C–F) Diagram of location of collecting GT, PT, RT and ST samples. Two lines represent the primary paramarginal incision and secondary intrasulcular incision when collecting PT samples during periodontal resentive surgeries. GT, osseous defect granulation tissue; NSPT, non‐surgical periodontal treatment; PT, inflamed gingival/supracrestal tissue; RT, granulation tissue attached on root surface of extracted tooth; ST, granulation tissue from tooth extraction sockets.

Four types of samples were collected (Figure 1), including (i) inflamed gingival tissue (PT), (ii) PGT in periodontal osseous defects (GT), (iii) PGT on the root surface of extracted teeth (RT), and (iv) PGT in extraction sockets of hopeless teeth (ST). PT was treated as the non‐granulation tissue control. Detailed sampling procedures for each type of tissue are described in Supporting Information 2.5.

### 2.5 Bacteriome DNA Extraction and 16S rRNA Sequencing

Total intra‐tissue bacteriome genomic DNA was extracted from freshly excised tissues using Molzym DNA isolation (Ultra‐Deep Microbiome prep) kit and sent for NGS 16S rRNA (V3–V4 regions) amplicon metagenomic sequencing (Novogene, China). The barcoded primer pair 341F (5′‐CCTAYGGGRBGCASCAG‐3′) and 806R (5′‐GGACTACNNGGGTATCTAAT‐3′) was used (Supporting Information 2.6).

### 2.6 Data Processing and Bacteriome Analysis

Raw data were processed with the DADA2 v1.22.0 (Callahan et al. 2016) pipeline. The amplicon sequence variants (ASVs) were assigned with taxonomy with a DADA2‐curated train set for the SILVA database v138.1. Microbiome community profiles, core microbiome, alpha diversity and beta diversity were evaluated with the *microbiome* v1.20.0, *mia* v1.6.0 and *vegan* v2.6–4 packages. Differentially abundant taxa among various tissues were selected using MaAsLin2 v1.16.0 (Mallick et al. 2021) with the fixed effects of tissue sample types only or adjustment with covariates of sex, age, obesity, smoking status and diabetes mellitus (DM). Microbial features were also selected by sPLS‐DA for tissue type classification using mixOmics v6.22.0 (Rohart et al. 2017) (See Supporting Information 2.7, 2.8 and 2.9 for data processing, feature selection and functional analysis). Unstratified functional prediction was performed using PICRUSt2 v2.5.0 (Douglas et al. 2020) and further analysed using ANCOM‐BC2 (Lin and Peddada 2024).

### 2.7 Single‐Cell Preparation, scRNA‐Seq Data Acquisition and Analysis

Freshly excised tissues were washed with HBSS, kept in MACS Tissue Storage Solution (Miltenyi Biotec, Germany) at 4°C overnight as in previous studies (Schütz et al. 2023) and shipped the next day to BGI (Hong Kong) for single‐cell preparation, 10× Genomics library construction and sequencing. Raw data were pre‐processed with the Cell Ranger v5.0.1 pipeline (Zheng et al. 2017) aligned to human genome (GRCh38 v2020‐A). Data were further analysed using Seurat package v5.0 (Hao et al. 2024) with cell identification by referring to known marker genes for oral mucosa (Williams et al. 2021) and previous studies (Behm et al. 2024; Liu et al. 2023; Morgan and Tergaonkar 2022; Shi et al. 2023; Xie et al. 2020) (see Supporting Information 2.10, 2.11 and 2.12 for scRNA‐seq acquisition, differential analysis and pseudotime analysis).

### 2.8 Histology and Immunohistochemistry Analysis

Selective samples were fixed in 10% neutral buffer formalin solution and examined using histology and immunohistochemistry (IHC). For histology, the slides were stained with Gill's haematoxylin and 1% eosin Y solution. For IHC, the antigen‐retrieved slides were incubated with primary antibodies of CD4 (1:500, Novus Biologicals) and NKG2D/CD314 (1:500, Novus Biologicals) and detected using the UltraVision Quanto Detection System HRP DAB (Thermo Fisher) at room temperature. Details are described in Supporting Information 2.13.

### 2.9 Statistics and Sample Size

Inter‐tissue difference of histological analysis was examined using the Kruskal−Wallis test with post hoc Dunn's test, controlling false discovery rate using Benjamini−Hochberg method. An a priori calculation of sample size for 16S rRNA‐seq using the *t*‐test was performed using G*Power v3.1.9.7 (Faul et al. 2009) referring to results of a previous study (Bao et al. 2020). Post hoc power was calculated by the Bray–Curtis dissimilarity and microbial features of this study. A prospective sample size of cells for scRNA‐seq was calculated using SCOPIT (Davis et al. 2019) based on the lowest proportion of cells reported in the Human oral mucosa cell atlas (Williams et al. 2021), requiring 3863 cells for 0.95 probability of success. A retrospective calculation was also performed based on our results. More details of statistics and sample size calculation are described in Supporting Information 2.14 and 2.15.

## 3 Results

### 3.1 Demographic and Clinical Profiles

Totally, 49 subjects with periodontitis were recruited (Figure 1; for demographics, see Table 1; for detailed information of each subject, see the additional Excel file in Supporting Information), including 63.3% (31/49) with Stage III and 36.7% (18/49) with Stage IV (Table S1); 89.8% (44/49) were Grade C and 10.2% (5/49) Grade B. Most patients (38/49) completed a course of non‐surgical periodontal treatment (NSPT), showing profoundly improved full‐mouth periodontal status (Tables S2 and S3).

**TABLE 1:** Demographic and systemic profile.

Variables		Total (*n* = 49)	Stage III (*n* = 31)	Stage IV (*n* = 18)
Sex	Female	27 (55.1%)	19 (61.3%)	8 (44.4%)
	Male	22 (44.9%)	12 (38.7%)	10 (55.6%)
Age		48.3 ± 12.0	46.5 ± 12.8	51.4 ± 10.2
Age group	18–29	5 (10.2%)	5 (16.1%)	0 (0%)
	30–59	33 (67.3%)	21 (67.7%)	12 (66.7%)
	≥ 60	11 (22.4%)	5 (16.1%)	6 (54.5%)
BMI		23.0 (21.4–24.7)	22.3 (21.2–24.6)	23.7 (22.2–24.7)
BMI (obesity group)	< 23 Normal	24 (49.0%)	18 (75%)	6 (33.3%)
	≥ 23 Overweight/obese	25 (51.0%)	13 (41.9%)	12 (66.7%)
Smoking status	Current	10 (20.4%)	6 (19.4%)	4 (22.2%)
	Former	5 (10.2%)	4 (12.9%)	1 (5.6%)
	Never	34 (69.4%)	21 (67.7%)	13 (72.2%)
Education level	Tertiary education	23 (47.0%)	16 (51.6%)	7 (38.9%)
	High school	17 (34.7%)	10 (32.3%)	7 (38.9%)
	Secondary school	7 (12.3%)	3 (9.7%)	4 (22.2%)
	Primary or below	2 (4.1%)	2 (6.5%)	0 (0.0%)
Drinking habit	Never	16 (32.7%)	12 (38.7%)	4 (22.2%)
	Occasional	31 (63.3%)	19 (61.3%)	12 (66.7%)
	Frequent	2 (4.1%)	0 (0.0%)	2 (11.1%)
Diabetes mellitus	No	44 (89.8%)	28 (90.3%)	16 (88.9%)
	Yes	5 (10.2%)	3 (9.7%)	2 (11.1%)
Hypertension	No	43 (87.8%)	27 (87.1%)	16 (88.9%)
	Yes	6 (12.2%)	4 (12.9%)	2 (11.1%)
Cardiovascular disease	No	47 (95.9%)	30 (96.8%)	17 (94.4%)
	Yes	2 (4.1%)	1 (3.2%)	1 (5.6%)
On medication	No	37 (75.5%)	23 (74.2%)	14 (77.8%)
	Yes	12 (24.5%)	8 (25.8%)	4 (33.3%)

*Note*: Data are presented as *n* (%) except for age and BMI. Stage III–IV refers to the different stages of periodontitis. Data in age are presented as mean ± standard deviation (SD) and data in BMI as median and interquartile range (IQR). Detailed information for each subject is available in Research Data.

### 3.2 Osseous GT Outmatches Socket‐Derived Ones in Local Periodontal Parameters of Involved Teeth

A total of 93 PGT samples, including 55 osseous PGT (GT), 18 root PGT (RT) and 20 socket PGT (ST) samples, as well as 16 inflamed periodontal tissue (PT) samples, were collected relative to treatment (Figures 1 and S1–S3 and Table S4). As anticipated, periodontally hopeless RT‐ and ST‐involved teeth exhibited worse tooth base than GT/PT‐involved ones (Table S5), which might have been the major rationale for the decision to extract. This result indicated that defect‐dependent intra‐tissue components may vary in response to local periodontal conditions.

### 3.3 Osseous GT Is Commensal‐Rich, While Root and Socket Tissues Are Periodontopathogen‐Abundant

**FIGURE 2:**
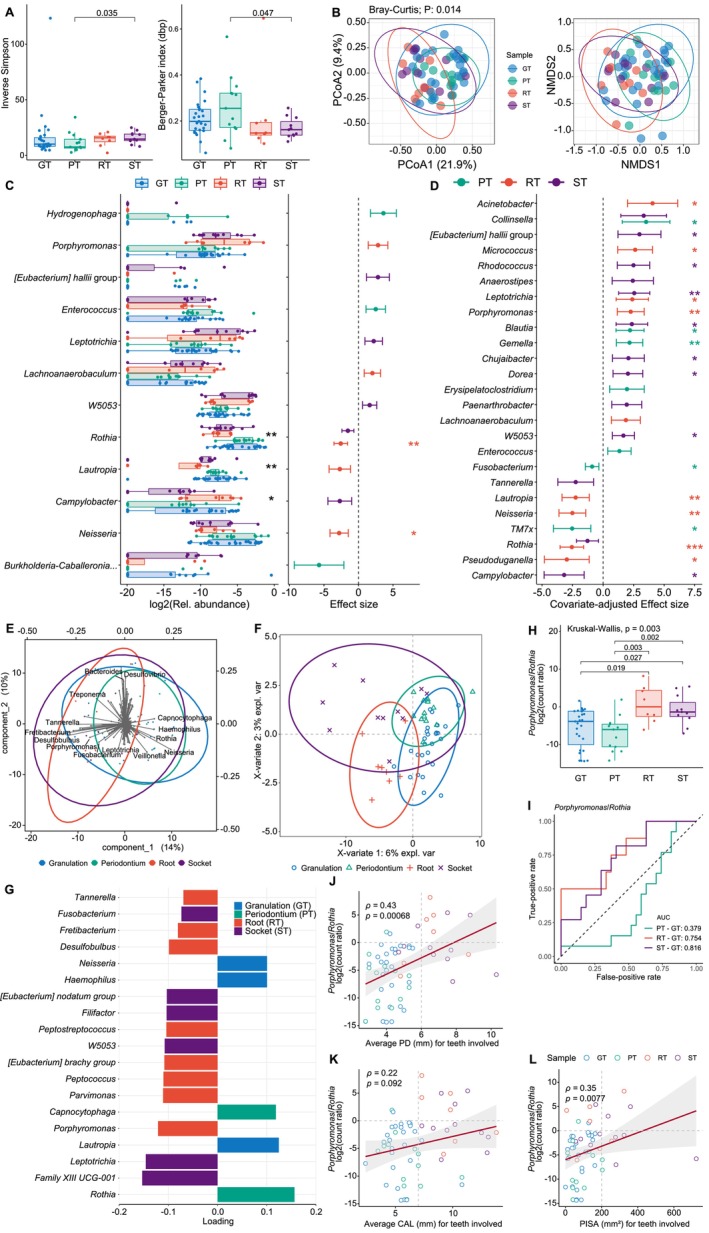
Multivariable statistical analysis to identify taxa associated with tissue types at genus level. (A) Alpha diversity indices of inverse Simpson (richness and evenness) and Berger–Parker index (dbp, dominance). (B) PCoA and NMDS of Bray–Curtis dissimilarity with PERMANOVA. (C) MaAsLin2 results without the adjustment of covariates using GT as the reference. MaAsLin2 effect sizes are shown in dot plot, with error bars representing 95% confidence intervals. The log_2_‐transformed relative abundance of selected covariate‐unadjusted genera are also shown in the box plot, along with significance of Kruskal‐Wallis test. *p < 0.05; **p < 0.01; ***p < 0.001. (D) MaAsLin2 results with the adjustment of covariates (sex, age, obesity, smoker and DM status) using GT as the reference. The taxa with *q* < 0.25 or *q* < 0.1 (for covariate adjustment) are shown with respective colours. *q < 0.05; **q < 0.01; ***q < 0.001. Genera with more than seven non‐zero count samples are shown. Full lists of results are available in Tables S7 and S8. (E) Principal component analysis biplot. (F) sPLS‐DA plot of the final model of genera. (G) sPLS‐DA‐selected important genera in Component 1. Bar length represents loading coefficient weight of selected genera, ranked in ascending order of importance, bottom to top; bar colour denotes the types of tissue in which the genus has the highest median abundance. Genera with loading > 0.7 are shown. (H) Ratios (log_2_) of the abundance (counts) of *Porphyromonas* to *Rothia* among different types of tissues. Kruskal–Wallis test with post hoc pairwise Wilcoxon signed‐rank test. *p‐*values were adjusted using the Benjamini–Hochberg procedure. (I) ROC of *Porphyromonas* to *Rothia* ratio for distinguishing the three types of tissue from GT. (J−L) Spearman correlation of ratios (log_2_) of the abundance (counts) of *Porphyromonas* to *Rothia* (P/R) among different types of tissues to corresponding site‐based average PD, average CAL and PISA of tooth/teeth involved (six probing sites). Proposed thresholds are shown as dashed lines: Log_2_(P/R) = 0; average PD = 6 mm; average CAL = 7 mm and PISA = 200 mm^2^ for granulation tissue–involved tooth. GT, osseous granulation tissue; PT, periodontal tissue; RT, root granulation tissue; ST, socket granulation tissue.

Therefore, we undertook 16S rRNA amplicon sequencing on intra‐tissue microbial DNA from 27 GT, 13 PT, 8 RT and 11 ST samples. After removing singletons and batch effects (Figure S4), we observed apparent alterations in intra‐tissue overall and core bacteriome composition across tissue types, hinting at more abundant commensals in GT and PT (Figures S5 and S6). GT and PT exhibited significantly lower diversity and higher dominance of alpha indices compared to ST (Figure 2A), indicating a shift in the microbiota. The significant disparity in Bray–Curtis dissimilarity (PERMANOVA *p* = 0.014, Figure 2B) and between GT and/or PT (GT/PT) and RT and/or ST (RT/ST) (pairwise PERMANOVA *p* < 0.05, Table S6) suggested distinct defect‐specific taxonomic signatures.

Utilising MaAsLin2, we further identified totally 12 differentially abundant genera compared to GT, reflecting inter‐tissue abundance change (*q* < 0.25, Figure 2C, Table S7), including RT/ST‐depleted commensals/symbiotes (*Rothia*, *Lautropia*) and RT‐enriched periodontopathogens (*Porphyromonas*), which was also verified using the Kruskal–Wallis test. Similar patterns were also observed at both the family and species levels (Figures S7, S8 and Table S7), suggesting relatively periodontal health–associated microbiota (commensal‐rich and periodontopathogen‐reduced) among GT at osseous defects. Additionally, *Porphyromonas*, *Fusobacterium* and the corresponding families/species were significantly associated with overweight/obesity (Table S8), echoing a potential periodontitis–obesity link (Hajishengallis and Chavakis 2021). There were also significant associations of bacterial taxa with age, sex, diabetes and smoking status. By controlling confounders, covariate‐adjusted MaAsLin2 revealed more tissue‐dependent differential taxa (Figure 2D).

Principal component analysis (PCA) biplot revealed three major directions of genus loading (Figure 2E), and the supervised sPLS‐DA distinguished GT/PT from RT/ST (Figure 2F), highlighting the contribution (loading > 0.1) of *Lautropia*, *Haemophilus* and *Neisseria* towards GT, *Rothia* and *Capnocytophaga* towards PT, and periodontopathogen genera towards RT/ST (Figure 2G, Table S9). The *Porphyromonas*/*Rothia* abundance ratio (P/R ratio), a periodontal dysbiosis signature (Boyer et al. 2020), further enhanced discernment of tissue types (Figure 2H), effectively classifying RT/ST versus GT (AUROC_RT−GT_ = 0.75; AUROC_ST−GT_ = 0.82, Figure 2I). There were significant correlations of *P*/*R* ratios with average PD (Spearman's *ρ* = 0.43, *p* = 0.00068) and PISA (*ρ* = 0.35, *p* = 0.0077) of tissue‐involved teeth, revealing their thresholds (Figure 2J−L). There were no significant differences in bacterial signature and local periodontal conditions for the subject‐paired GT−PT and RT−ST (*p* > 0.05, Figure S9, Tables S10 and S11). These results revealed that osseous defects (GT) and extraction sockets (RT/ST) exhibited distinct microbial niches, consistent with local periodontal parameter variations (Table S5). Importantly, there were no significant differences between GT subtypes or RT/ST before and after NSPT (Tables 2 and S12−S14), justifying the distinction between GT and RT/ST.

**TABLE 2:** Local periodontal parameters and microbial profiles of GT, RT and ST.

Parameter (GT)	Infrabony (*n* = 17)	Combined (*n* = 14)	*p* ^a^
Mean PD (mm)	4.2 ± 0.8	4.2 ± 1.0	0.823
Mean CAL (mm)	5.2 (4.7, 5.8)	6.0 (5.5, 6.3)	0.147
BOP (%)	50.0 (33.3, 66.7)	58.3 (50.0, 66.7)	0.440
PISA (mm^2^)	57.2 ± 42.0	110.9 ± 48.0	**0.003**
PR ratio (log_2_)	−3.6 (−11.6, −1.2)	−4.4 (−7.7, −0.6)	0.796
Alpha indices			> 0.05^b^
Bray‐Curtis distance			0.895^b^

Parameter (RT)	Before (*n* = 4)	After (*n* = 4)^c^	*p* ^a^
Mean PD (mm)	6.5 ± 1.6	6.1 ± 1.0	0.672
Mean CAL (mm)	9.4 ± 3.1	9.9 ± 0.2	0.751
BOP (%)	100.0 (95.8, 100.0)	100.0 (100.0, 100.0)	0.564
PISA (mm^2^)	214.5 ± 135.1	153.1 ± 32.0	0.517
*PR* ratio (log_2_)	1.1 ± 6.5	0.3 ± 3.9	0.854
Alpha indices			> 0.05^b^
Bray‐Curtis distance			0.735^b^

Parameter (ST)	Before (*n* = 8)	After (*n* = 3)^c^	*p* ^a^
Mean PD (mm)	7.6 ± 1.4	5.8 ± 1.7	0.206
Mean CAL (mm)	10.6 ± 2.3	9.6 ± 0.6	0.266
BOP (%)	100.0 (95.8, 100.0)	100.0 (100.0, 100.0)	0.447
PISA (mm^2^)	230.7 ± 110.7	184.6 ± 97.6	0.538
*PR* ratio (log_2_)	0.0 ± 4.0	−3.1 ± 3.4	0.258
Alpha indices			> 0.05^b^
Bray‐Curtis distance			0.251^b^

*Note*: GT samples are compared between infrabony and combined defect types. RT and ST are compared between before and after completion of NSPT.

Abbreviations: NSPT, non‐surgical periodontal treatment; PR ratio, porphyromonas‐to‐Rothia ratio.

^a^ Data are presented as mean ± standard deviation (SD) for normal distribution or median and interquartile range (IQR) for non‐normal distribution, which are tested by *t*‐test or Mann–Whitney *U* test.

^b^ Alpha diversity indices are tested by Mann–Whitney *U* test. Beta diversities are tested by PERMANOVA on Bray–Curtis distance.

^c^ Teeth with delayed extraction still received NSPT.

### 3.4 Tissue‐Oriented Disparity of Intra‐Tissue Bacteriome Alters Predicted Microbial Functions

**FIGURE 3:**
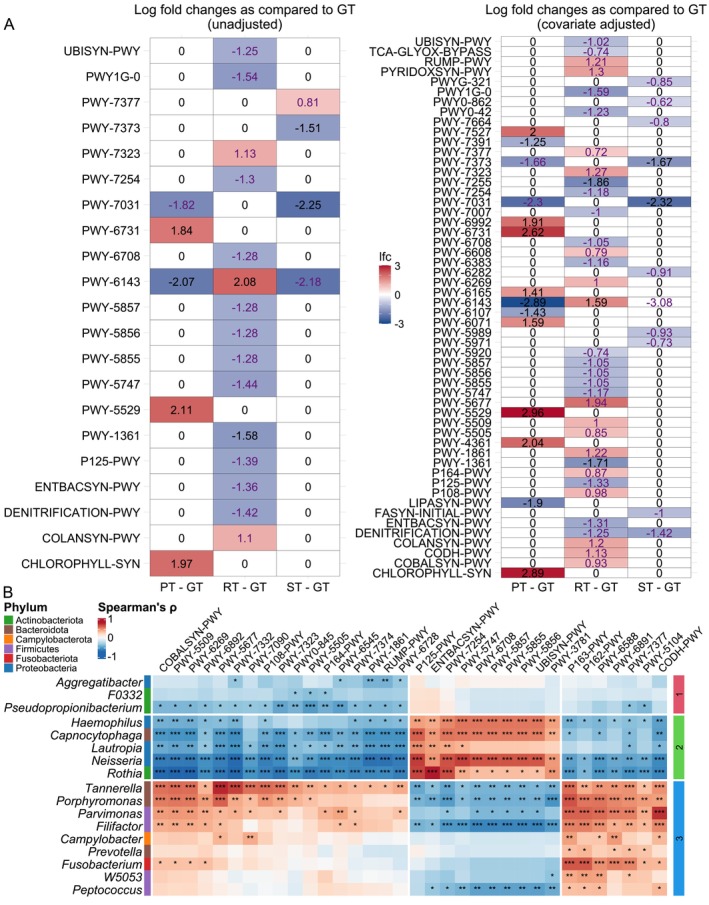
Differentially abundant microbial functions among different tissue types. (A) PICRUSt2‐predictioned MetaCyc pathway abundance was analysed using ANCOM‐BC2. Multiple pairwise comparisons against the reference GT were tested using Dunnett's type of test with control of mixed directional FDR (mdFDR) using Holm–Bonferroni correction. The heatmaps are coloured to the natural log of fold change (lfc). For clarity, lfc is reset to zero if *q* > 0.2 and the label of lfc is coloured in purple if passed the sensitivity analysis for pseudo‐count addition. GT, osseous granulation tissue; PT, periodontal tissue; RT, root granulation tissue; ST, socket granulation tissue. (B) Heatmap of Spearman's correlation between *clr* of Maaslin2‐selected genera and counts of ANCOMBC2‐selected PICRUSt2‐predicted MetaCyc pathways. Heatmap is split into three *k*‐means clusters for both genera and pathways. The corresponding phyla are shown. The *k*‐means clusters of genera are coloured and numbered. *q < 0.05; **q < 0.01; ***q < 0.001.

We employed PICRUSt2‐integrated ANCOM‐BC2 analysis to elucidate microbial function shifts and their links to defects. Notably, MetaCyc pathways for ubiquinol biosynthesis and TCA cycles were attenuated and butanoate biosynthesis was enhanced in RT/ST with reference to GT (*q* < 0.05, Figure 3A, Tables S15 and S16). Elevated short‐chain fatty acid (SCFA) fermentation and the concurrent weakened propanoate degradation aligned with periodontopathogens (*q* < 0.05, Figure 3B, Table S17), which might imply the detrimental nature of the extraction socket niche given the deleterious impact of butyrate on the periodontium (Zhao et al. 2020).

KEGG enrichment highlighted extensive pathway variation between RT/ST and GT (Figure S10 and Table S18) and the underlying microbe–KEGG Orthology (KO) association (Table S19). RT and ST shared similar enrichment profiles (sugar, butanoate and propanoate metabolism and citrate cycle), favouring periodontitis‐linked pathways, for example, porphyrin metabolism and O‐antigen nucleotide sugar biosynthesis for virulence factor lipopolysaccharide. Collectively, osseous GT contains less periodontally dysbiotic bacteriome and related periodontium‐deleterious bacterial functions, indicating that its cellular composition may also differ with the socket niche.

### 3.5 Osseous GT Is MSC–Rich, While Socket GT Is Natural Killer Cell (NKC)‐Abundant)

**FIGURE 4:**
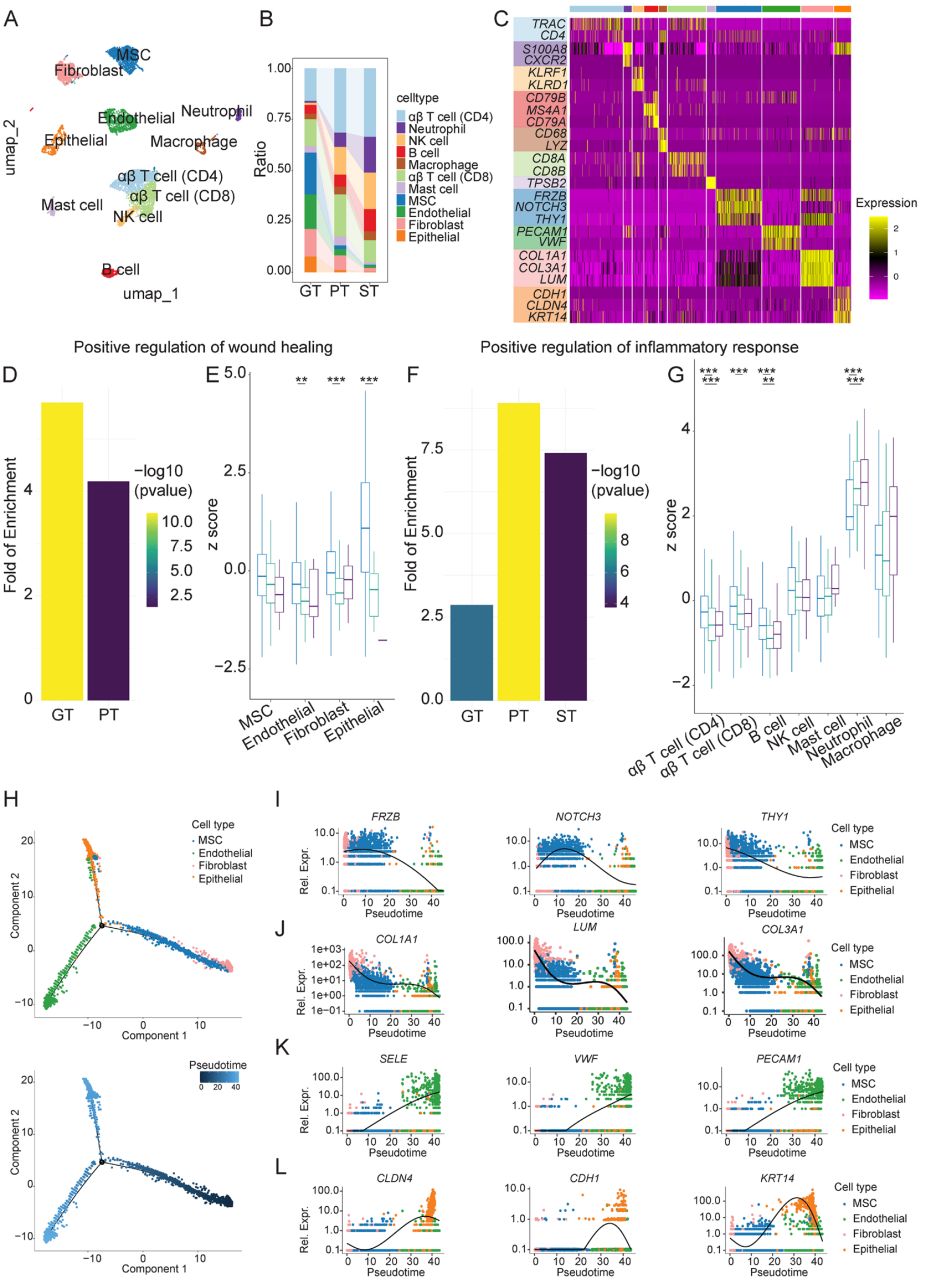
Single‐cell RNA sequencing of GT, PT and ST. (A) UMAP of major cell types by scRNA‐seq (16,100 cells, GT, PT and ST). (B) Bar plot of relative cell proportions in GT, PT and ST. (C) Heatmap of the average expression of marker genes for each identified cell type in sampled tissues. Fold enrichment of GO term ‘Positive regulation of wound healing’ (D) and scaled expression of genes associated with wound healing (z‐score of involved genes) (E) in MSC, fibroblast, endothelial and epithelial cells of GT, PT and ST. Fold enrichment of GO term ‘Positive regulation of inflammatory response’ (F) and scaled expression of genes associated with inflammatory response (z‐scores of involved genes) (G) of immune cells in GT, PT and ST. Fold enrichment is defined as the percentage of identified genes in a GO term, divided by the corresponding percentage in the background. The inter‐tissue difference was assessed using one‐way ANOVA and post hoc Tukey HSD test. *p < 0.05; **p < 0.01; ***p < 0.001. (H) Pseudotime analysis demonstrating the progression of cell clusters of MSC, fibroblast, endothelial and epithelial cells (upper panel) along the pseudotime trajectories (lower panel). The expression dynamics along pseudotime coordinates of selected marker genes from MSCs (I) and fibroblast (J) into two branches of endothelial (K) and epithelial cells (L) bifurcately. GT, osseous granulation tissue; MSC, mesenchymal stem cell; PT, periodontal tissue; RT, root granulation tissue; ST, socket granulation tissue.

We further explored cellular profiles of different types of tissues by utilising scRNA‐seq, that is, one GT, one PT and one ST from three male subjects (one tissue per subject) with similar backgrounds (age: 40s, BMI > 24, smoking: never or former, without uncontrolled systemic disease and use of medication; Table S20). After appropriate data processing, quality control and integration (Figure S11A, Tables S21−S24), an integrated single‐cell transcriptome of 16,100 cells was obtained, including 8100 (GT), 4000 (PT) and 4000 (ST) cells, which resolved into 14 distinct clusters in the Uniform Manifold Approximation and Projection (UMAP) space with distinguishable transcriptomic signatures (Figure S11B,C) and annotated as 11 major cell types (Figure 4A). GT included a higher proportion (20.5%) of MSCs (*FRZB, NOTCH3, THY1*) and fewer (1.2%) NK cells (*KLRF1*, *KLRD1*) as well as neutrophils (0.9%, *S1008A*, *CXCR2*) than PT (1.7% MSCs, 13.5% NK cells and 7.0% neutrophils) and ST (0.3% MSCs, 17.9% NK cells and 17.6% neutrophils) (Figure 4B) with distinct cell type–specific gene expression profiles (Figures 4C, and S12−S14).

KEGG pathway enrichment of scRNA‐seq displayed cytokine−cytokine receptor interaction (hsa04060) among all three tissues, reflecting their inflammatory nature. Nevertheless, GT highlighted PI3K−Akt signalling (hsa04151), focal adhesion (hsa04510) and ECM−receptor interaction (hsa04512) pathways, aligning with GO functional terms, for example, wound healing, extracellular matrix organisation and epithelial cell adhesion (Figure S15; Tables S25 and S26). Contrastingly, PT and ST revealed immune activation and regulation pathways. GT was also enriched with functions of osteoblast differentiation and bone mineralisation, while PT and ST showed significant enrichment with osteoclast differentiation. Corroboratively, GT manifested significantly higher expression of genes associated with wound healing among tissue and cell types but lower expression of inflammatory response–associated genes (Figure 4D−G). To explore the regenerative potential of GT, we constructed a pseudotime trajectory model starting from a less differentiated state shared by MSCs and fibroblasts. The trajectory diverged into two specialised branches, terminating in endothelial and epithelial lineages, suggesting differentiation tendencies from mesenchymal progenitors (MSCs and fibroblasts) towards these fates (Figure 4H). Consistently, MSC and fibroblast marker genes declined along the trajectory, whereas endothelial and epithelial markers progressively increased (Figure 4I−L). These results highlight higher propensity of osseous GT for wound healing and periodontal regeneration.

**FIGURE 5:**
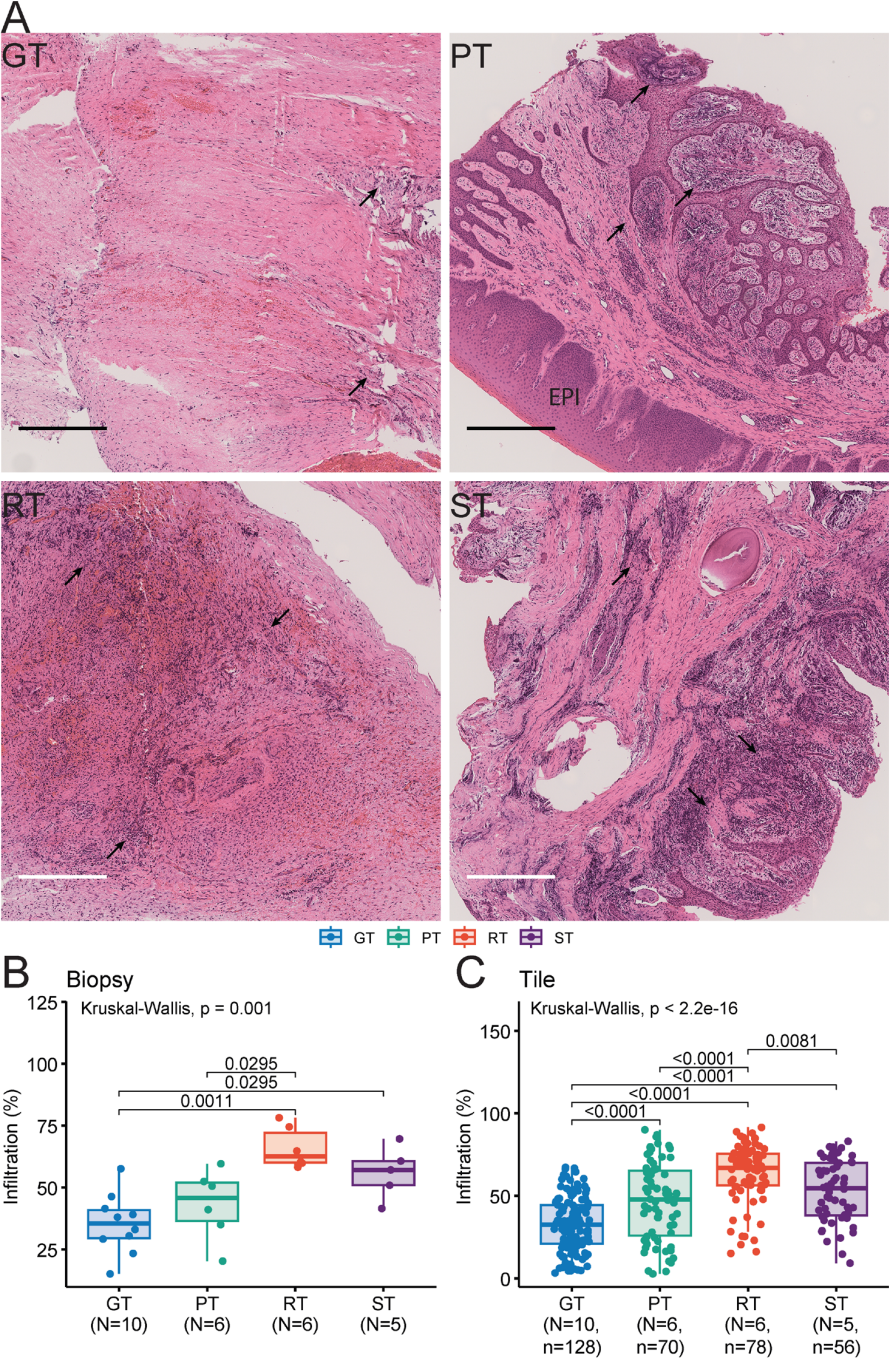
Histology staining of different types of tissue. (A) Representative images of H&E staining of four different types of tissues. Extensive inflammatory infiltrate is present among RT, ST and lamina propria of PT. Infiltrate (indicated by arrows) is less extensive among GT and epithelium of PT. Each slide of specimen or biopsy was automatically segmented into sequential square tiles (Figure S16). Inflammatory infiltrate of tiles of each slide was semi‐quantified with an object classifier (artificial neural network, ANN_MLP). The percentage of infiltration was calculated by dividing the number of positively infiltrate‐classified cells by the total number of detected cells in each specimen (B) and each square tile (C). The difference of Infiltration (%) among tissue types was statistically assessed by Krusakl−Wallis test (*p*) with post hoc Dunn's test controlling false discovery rate using Benjamini−Hochberg method (*q*). Significant *q* values (*q* < 0.05) are shown. GT, osseous granulation tissue; *n*, number of square tiles; *N*, number of tissue samples (biopsies); PT, periodontal tissue; RT, root granulation tissue; ST, socket granulation tissue; Scale bar = 400 μm.

Histology (total *N* = 27) revealed extensive inflammatory infiltrates among lamina propria (LP) and sulcular epithelium (SE) of PT (*N* = 6) as well as RT (*N* = 6) and ST (*N* = 5), whereas GT (*N* = 10) exhibited markedly less infiltration at both specimen and tile levels (Figure 5A−C, Dunn's test *q* < 0.05). GT had significantly less infiltrate than LP and SE of PT, but more than keratinised oral epithelium (EPI) of PT (Figure S17). Densely packed collagen fibre bundles were observed in GT. Immunohistochemistry further demonstrated reduced H‐score and immunoreactivity score (IRS) for NKG2D (NK biomarker) and CD4 in GT (*N* = 6) and PT (*N* = 5) compared to RT (*N* = 4) and ST (*N* = 4) (Figures S18 and S19; Dunn's test *q* < 0.05), corroborating cytotoxic features in socket GT identified by scRNA‐seq. These findings indicate distinct cellular profiles of GT from PT despite comparable bacteriome profiles in a similar niche.

## 4 Discussion

PGT plays a pivotal role in periodontal wound healing, yet it is routinely discarded in clinical practice due to its perceived negative impact on treatment outcomes (Sam et al. 2024). In this study, we have analysed three hypothetically classified types of PGT (GT, RT and ST), and the inflamed periodontal tissue (PT) as the non‐granulation diseased tissue reference. As such, GT and PT were collected post NSPT, while RT and ST were derived during extraction of teeth with periodontally hopeless prognosis. Given that periodontal healing is influenced by microbial and cellular microenvironments (Ahn and Shin 2008), we have demonstrated a distinct bacterial signature and cellular composition among different PGTs, in line with tooth‐related prognosis in periodontitis patients.

Omic studies on the biology of PGT remain limited (Chowdhry et al. 2018), with only two recent scRNA‐seq studies (Li et al. 2024; Zhu et al. 2026) and a bulk transcriptomic study (Sam et al. 2025) published to date. Moreover, the ‘Anna Karenina principle’ of periodontitis (Altabtbaei et al. 2021), marked by high heterogeneity among periodontitis‐associated microbiota, further makes the bacteriome profiling within PGT intricate. Thus, our pioneering analysis of the intra‐tissue bacteriome demonstrates the underlying severe intra‐tissue dysbiosis (depletion of oral commensals while enrichment of pathobionts) and bacterial functions (butyrate biosynthesis) favouring periodontal disruption (Zhao et al. 2020) in extraction socket RT/ST, corroborating clinically evident evidence of hopeless prognosis. As a periodontal dysbiosis signature (Boyer et al. 2020), the P/R ratio may guide clinical decisions on appropriately managing PGT on the basis of their biological attributes.

scRNA‐seq underscores the cellular subtype heterogeneity in oral mucosa, encompassing stromal, mesenchymal, epithelial and immune cells (Behm et al. 2024; Caetano et al. 2021; Chen et al. 2022; Fan et al. 2023; Williams et al. 2021). Our data indicate that osseous GT exhibits a reparative phenotype, enriched in MSCs and fibroblasts with reduced inflammatory infiltration, supported by KEGG‐enriched pathways in GT involving collagen synthesis, extracellular matrix organisation, and osteogenesis, consistent with bulk transcriptomic findings (Sam et al. 2025).

Contrastingly, socket‐derived ST and inflamed gingival/supracrestal PT enriches periodontally disruptive features especially with a increasing NK proportion, consistent with recent report of increased neutrophils and macrophages infiltrate and reduced dendritic cells in gingiva of periodontitis patients (Mo et al. 2024), as well as distinct cellular profiles (Li et al. 2024). Severe periodontitis disrupts epithelium−stroma signalling, favouring immune‐cell‐originated signalling (Caetano et al. 2021).

MSCs are crucial for periodontal regeneration and bone homeostasis within the osteoimmunology microenvironment (Behm et al. 2024; Chen et al. 2022; Fan et al. 2023; Liao et al. 2020), with diverse mesodermal lineages in healthy gingiva (Kim et al. 2021). Our pseudotime‐predicted two‐branched trajectories of mesenchymal progenitors are based on the gene‐expression dynamics, requiring future validation of lineage transition. Clinical trials using a‐BMMSC biocomplexes exhibit promise for periodontal regeneration (Apatzidou et al. 2021; Apatzidou et al. 2024), with future adjunctive autologous fibrin/platelet lysate (Calciolari et al. 2025). However, MSCs, pre‐osteoblasts and osteoblasts decrease significantly following NSPT (Chen et al. 2022), suggesting the limits of NSPT for osteoblast‐mediated bone regeneration (Sculean et al. 2015). Depletion of MSCs and enrichment of osteoclastogenesis‐related pathways in ST further suggest a deleterious hindrance to regeneration, consistent with previous studies (Ahn and Shin 2008). Moreover, ambiguous outcomes in PGT preservation (Adam et al. 2022; Moreno Rodriguez and Ortiz Ruiz 2022; Sam et al. 2024) may reflect the presence of detrimental cellular and microbial components. Considering the accessibility to GT compared to alveolar bone marrow (Apatzidou et al. 2021; Apatzidou et al. 2024), isolating MSCs from osseous GT combined with microbial decontamination may represent a feasible adjunctive strategy. Minimal differences between RT and ST support the combined consideration as socket‐derived PGT in future studies.

There are limitations of the study to be addressed for future investigations and clinical translation. First, ideally, paired PGT samples are collected from both osseous defects and extraction sockets of the same individual subject. However, it is rather challenging in clinical practice because the volume, composition and viable cell yield of the tissues may vary substantially among individuals, thereby making it difficult to obtain sufficient high‐quality cells for simultaneous microbiomics and single‐cell sequencing. Second, healthy PT could have been obtained for reference from subjects receiving gingivectomy for aesthetic crown lengthening or orthodontic treatment purposes. Third, 16S rRNA‐seq cannot provide microbiomics profiles at the species level. Additionally, our scRNA‐seq analysis was performed on one representative sample per tissue type due to the limited size of PGT, thereby limiting the resolution for detecting rare sub‐populations such as MSC‐like pericytes (Chen et al. 2022) (Supporting Information Discussion). Consequently, the scRNA‐seq findings provide a preliminary overview of intra‐tissue cellular heterogeneity rather than definitive statistical inference. Moreover, our study did not focus on dynamic profiles. Considering the heterogeneous nature and inter‐individual variation of PGT, future investigations with a large sample size and confounder controlling are needed.

The present multi‐omics study highlights the ‘Dr Jekyll and Mr Hyde’ features of different types of PGT, and shows that the osseous GT contains more periodontal health–associated intra‐tissue bacteriome and cellular composition. It is worthwhile to further explore the translational potential of the MSCs from osseous GT through pre‐clinical and clinical investigations for enhancing therapeutic outcomes of periodontitis patients.

## Author Contributions

T.C. contributed to conception and design, acquisition, analysis and interpretation, and drafted and critically revised the manuscript; T.L. contributed to analysis and interpretation, and drafted the manuscript; T.Y. Wong contributed to design, acquisition and analysis, and drafted the manuscript; B.C., C.L. contributed to analysis, and critically revised manuscript; X.D. contributed to the design, and critically revised manuscript; H.C. and W.Q. contributed to the interpretation, and critically revised manuscript; G.P. and L.J. contributed to conception, design and interpretation, and critically revised manuscript. All authors have their final approval and agree to be accountable for all aspects of work.

## Funding

This work was supported by the Innovation Fund of Faculty of Dentistry, The University of Hong Kong (to T.C.) and the Modern Dental Laboratory/The University of Hong Kong (HKU) Endowment Fund (to L.J.).

## Conflicts of Interest

The authors declare no conflicts of interest.

## Supporting information


**Data S1:** Supporting Information.

**Data S2:** Detailed anonymous anthropological, clinical and periodontal information for all participants.

## Data Availability

The raw 16S rRNA gene amplicon sequencing data and scRNA‐seq data reported in this study have been deposited in NCBI SRA (https://www.ncbi.nlm.nih.gov/sra) with BioProject ID: PRJNA1177177. The scRNA‐seq data set generated in this study has been uploaded to the GEO database (GSE299738). Other data in the figures are provided in the Supplementary Tables. The R codes for analysis and figures are available at: https://github.com/tfccheng/Microbe‐cell‐profiles‐periodontal‐granulation‐tissues.
