# Supplementary material for: Intra‐Tissue Bacteriome and Cellular Profiles in Periodontal Granulation Tissue From Osseous Defects and Extraction Sockets
Source: J Clin Periodontol. 2026 Feb 24;53(5):806–20. doi: 10.1111/jcpe.70108 (PMC13086548; doi:10.1111/jcpe.70108)
Supplement: Supplementary file 1 — Data S1: Supporting Information. [file JCPE-53-806-s001.pdf]

# Supplementary Materials

**Title: Intra-tissue bacteriome and cellular profiles in periodontal granulation tissue from osseous defects and extraction sockets**

**Authors:** Tianfan Cheng, Tianle Li, Tsz Yung Wong, Beibei Chen, Chongshan Liao, Xun Ding, Hui Chen, Wei Qiao, George Pelekos, Lijian Jin

This file includes additional detailed descriptions of Materials and Methods (Section 2) and discussion of pericytes and MSCs (Section 4). It also includes additional supporting figures, anonymous patient information (a separate Excel file), and tables for the main text.

## 2 Supplementary Materials and Methods

### 2.1 Questionnaire

Questionnaires were filled in by the participants to obtain background information, including educational level, BMI, systemic conditions (presence of diabetic mellitus, immune disease, genetic disease, cardiovascular disease and infectious disease), history of medication, dietary habits, cigarette smoking and alcohol drinking, lifestyles, oral hygiene habits, and exposure to radioactive and harmful substances. Subjects were also classified into 'Normal' and 'Overweight/Obese' based on BMI values using the cutoff of BMI $\geq$ 23.0 kg/m$^2$ for Asian populations as recommended by WHO (WHO Expert Consultation, 2004).

### 2.2 Inclusion and Exclusion Criteria

Inclusion criteria: i) Chinese adults aged >18 years old; ii) diagnosed with Stages III−IV periodontitis following the 2018 classification of periodontal disease (Tonetti et al. 2018), scheduled for periodontal surgery and/or extraction of periodontally involved teeth; iii) normal dietary habits (non-vegetarians/vegans or dieters).

Exclusion criteria: i) currently uncontrolled systemic diseases; ii) infectious diseases such as AIDS, hepatitis B and COVID-19; iii) cancer; iv) use of antibiotics within the past three months; v) continuous use of immunosuppressant and bisphosphonate medications, steroids/other hormone drugs, and anti-inflammatory agents; vi) pregnant or lactating; and vii) taking prescribed or over-the-counter probiotics (no limit on the consumption of cheese and yogurt).

### 2.3 Periodontal Examination

FMPS, FMBS, number of teeth, full-mouth mean PD, full-mouth mean CAL, and percentage of sites with PD ≥ 4 mm and ≥ 6 mm were calculated for subject-level analysis. Local periodontal parameters from each sampled tooth were presented as mean PD and CAL of the six-site charting of sampled teeth. The local percentage of bleeding on probing (BOP) was calculated as a percentage of sites with BOP on the six-site dichotomous records of sampled teeth. Using these data, the periodontal inflamed surface area (PISA) was calculated to quantify each sample's inflamed periodontal tissue and assess the extent of inflammation (Miki et al., 2021; Nesse et al., 2008).

### 2.4 Periodontal Therapy

Our study did not change the normal treatment protocol for collecting these samples. Non-surgical periodontal treatments (NSPT) were performed by the same dentist, according to steps 1 and 2 therapy specified in the EFP S3 level clinical practice guideline (Herrera et al., 2022; Sanz et al., 2020). Non-surgical treatment includes oral hygiene instruction using the Goal setting, Planning and Self-monitoring (GPS) approach (Tonetti et al., 2015), root surface debridement, and restorative treatment. Occlusal adjustment and periodontal splinting were performed when necessary.

Extraction of periodontally involved teeth with hopeless prognosis (Cortellini et al., 2011) was performed according to the operator's treatment plan. Teeth with a hopeless periodontal prognosis are defined as having 360-degree bone loss to or beyond the apex due to periodontal reasons, with a negative or inconclusive response on pulp tests. The timing of extraction depends on the operator's treatment plan. A tooth deemed hopeless is extracted before NSPT. For teeth extracted after surgical treatment, they present with no acute symptoms at baseline. Delayed extraction occurs due to the patient's preference, such as during periodontal surgery. Those teeth with delayed extraction still received NSPT attempting to save them. Granulation tissues on the root surface and in the tooth extraction socket were collected as RT and ST, respectively.

A full-mouth periodontal examination was performed three months after NSPT. Sites with residual PD ≥ 6mm after NSPT were subjected to periodontal surgeries (Sanz et al., 2020). Patients' systemic condition, oral hygiene condition, compliance, and tooth strategic value were assessed before periodontal surgeries were planned. GT and PT were collected during periodontal surgeries.

Timeline of periodontal therapy (Fig. 1):

The extraction was performed either (1) during Steps 1 and 2 therapy before periodontal re-evaluation or (2) during surgical phase. All extracted teeth were deemed periodontally hopeless in the baseline examination; Extraction was performed together with periodontal surgeries to improve patient morbidity. Our periodontal treatments follow the current EFP S3 guidelines. Steps 1 and 2 periodontal therapies include oral hygiene instructions, scaling, and root surface debridement as indicated in EFP S3 guidelines for treatment of Stages I-III and IV periodontitis. Root surface debridement was performed in a quadrant approach, minimally invasive to avoid unnecessary soft tissue damage. Periodontal re-evaluations were performed 12 weeks after completion of root surface debridement. Bi-weekly oral hygiene review and professional mechanical plaque removal (PMPR) were performed during the 12 weeks of periodontal healing after NSPT.

All periodontal surgeries and sampling of GT and PT were performed after periodontal re-evaluation. Periodontal surgeries and sampling of GT and PT were performed within 3 months after periodontal re-evaluation to ensure the accuracy of the periodontal parameters used in our study, and to ensure minimal re-contamination of the root surface.

Bi-weekly oral hygiene review and professional mechanical plaque removal (PMPR) were performed after periodontal re-evaluation and before the commencement of periodontal surgeries.

**2.5 Collection of Tissue Samples**

We hypothetically classified three types of periodontal granulation tissue (PGT) according to its sampling site. Four types of samples were collected, including i) inflamed gingival tissues (PT); ii) PGT in periodontal osseous defects (GT); iii) PGT on the root surface of extracted teeth (RT); and iv) PGT in extraction sockets of periodontally involved teeth (ST). PT was treated as the non-granulation tissue control.

PT collection:

During resective periodontal surgery, gingival tissue was excised as part of the surgical aim for apical repositioning of the gingival margin and achieving pocket depth reduction when adequate keratinized tissue was present. The tissue excision was achieved by a primary interval beveled scalloped paramarginal incision and a subsequent secondary intrasulcular incision through the bottom of the periodontal pocket (Carnevale & Kaldahl, 2000). The distance between the paramarginal incision and the gingival margin depended on the probing depth (Fig. S1). The excised gingival/supracrestal tissue was collected as the inflamed gingival tissue/supracrestal sample (PT). In addition, PT samples were also collected in distal wedge

procedures. Buccal and lingual internal beveling incisions were performed at the retromolar pad of a distal molar. The triangular-shaped tissue was then removed from the underlying bone (Lang et al., 2008). PT includes inflamed gingival tissue, e.g., pocket epithelium, connective tissue, and gingival epithelium.

GT collection:

GT was collected from the granulation tissue of periodontal osseous defects during periodontal surgery, e.g., open flap debridement, periodontal regeneration surgery, periodontal resective surgery (Fig. 1). Herein, GT was further subclassified into i) granulation tissue from infrabony defect (Fig. S2A); ii) granulation tissue from combined infrabony and furcation defect (Fig. S2B). Full-thickness mucoperiosteal flaps were raised to expose the osseous defect. The surgical type and flap design were chosen by the operator, based on the aim of surgery, the morphology of osseous defect, and periodontal parameters. In general, papilla preservation flap with minimally invasive surgical technique (MIST) or modified minimally invasive surgical technique (M-MIST) was performed for periodontal regeneration, exposing a 1-mm margin of healthy bone (Cortellini & Tonetti, 2007, 2009). Resective surgeries were performed using intrasulcular incision through the bottom of the periodontal pocket, and secondary beveled paramarginal incisions if necessary, also with distal wedge procedures for distal molars when needed (Carnevale & Kaldahl, 2000). Extension of the flap was determined by the bone exposure required for osseous surgery and/or root resection. Afterward, granulation tissue was carefully removed with a Gracey curette or diamond file, followed by root debridement with ultrasonic and hand instruments. GT sample was carefully removed by a Gracey curette or diamond file and was immediately transferred to Hank's Balanced Salt Solution (HBSS). An ultrasonic scaler was also used to assist in the removal of GT.

RT and ST collection:

Granulation tissue that was attached to the root surface of extracted tooth was collected by being scrapped away from the root surface and labelled as the root granulation tissue (RT) (Fig. S3A). Granulation tissue was also routinely removed during extraction of periodontally involved teeth for better wound healing and socket grafting in preparation for future implant bed. For granulation tissue remaining in the extraction socket (the socket granulation tissue, ST) sample was collected by curetting against the bony wall of the extraction socket, with the use of Lucas bone curettes and Lucas surgical curettes, after extraction had been performed (Fig. S3B).

To be qualified for downstream 16S rRNA-seq and scRNA-seq, the size of collected tissue samples has to be a minimum of $2\times2\times2$ $mm^3$.

### 2.6 Microbiome DNA Extraction and 16S rRNA Amplicon Metagenomic Sequencing

Total microbiome genomic DNA was extracted from freshly excised granulation tissue or other tissue using the Molzym DNA isolation (Ultra-Deep Microbiome prep) kit. Briefly, fresh tissues were washed with ice-cold Hanks' Balanced Salt Solution (HBSS) and cut into small pieces of about 1 $mm^3$ with further HBSS wash, to remove blood and non-intra-tissue microorganisms. The tissues were then dissociated and lysed with Proteinase K and corresponding lysis buffer to release human DNA, which was further digested by DNase for human DNA depletion, and enrichment of the bacteria cells. Then, bacterial DNA was extracted from the pellet of enriched bacteria and sent for NGS 16S rRNA amplicon metagenomic sequencing (Novogene, China). Amplicon library of the 16S rRNA V3–V4 regions was amplified with the barcoded primer pair of 341F (5'-CCTAYGGGRBGCASCAG-3') and 806R (5'-GGACTACNNGGGTATCTAAT-3'), with inclusion of two blank controls for each sample for verifying absence of contamination. PE250 paired-end sequencing was performed on the NovaSeq 6000 Systems (Illumina, USA).

### 2.7 Data Processing and Bacteriome Analysis

The raw sequencing data were preprocessed with DADA2 v1.22.0 (Callahan et al., 2016) pipeline, which produced the datasets of Amplicon Sequence Variant (ASV) sequences and counts. The ASVs were assigned with taxonomy with a DADA2-curated train set for SILVA database v138.1 (Quast et al., 2013). Singletons were filtered out from the DADA2-generated dataset, producing a minimally filtered dataset (MFD) with 3,913 ASVs remaining. Batch effects (BE) were removed using Tune_ConQuR function of ConQuR v2.0 (Ling et al., 2022), including covariates of tissue sample types, sex, age, BMI, overweight/obesity, full diagnosis, smoking status, and DM. The resultant BE-removed dataset was applied for downstream analyses. Bacteriome community profiles, core microbiome, alpha diversity, and beta diversity were evaluated with *microbiome* v1.20.0, *mia* v1.6.0 and *vegan* v2.6-4 packages. Bray–Curtis dissimilarity of principal coordinate analysis (PCoA) of different tissues was tested using Permutational Multivariate Analysis of Variance (PERMANOVA) *adonis2* of *vegan* and *pairwiseAdonis* v0.4.1.

### 2.8 Selection of Microbial Features for Tissue Types

Differentially abundant taxa among PT, RT, and ST versus GT were analyzed using MaAsLin2 v1.16.0 (Mallick et al., 2021) for a mixed-effect modelling applying compound Poisson linear model (CPLM) and total sum scaling (TSS) normalization at levels of family, genus, and

species. The fixed effects included tissue sample types only or adjustment with covariates of sex, age, obesity, smoking status, and DM. Among families, "Mitochondria" was removed. Taxon features with large variation were selected using sPLS-DA for tissue type classification using mixOmics v6.22.0 (Rohart et al., 2017) with cross-validation setting of folds/repeats (8×50), applying filtering minima of relative abundance of 0.05% and prevalence of 5%, and robust centered log-ratio (rclr) transformation of relative abundance. PCA was performed using mixOmics.

**2.9 Microbial functional analysis for Tissue Types**

Unstratified functional prediction was performed using PICRUSt2 v2.5.0 (Douglas et al., 2020), and the predicted results of KEGG orthologs (KOs) and MetaCyc pathways (PWs) were further examined for tissue association using ANCOM-BC2 (Lin & Peddada, 2024) with or without adjustment of covariates of sex, age, overweight/obesity, smoking status, and DM. *P* values were adjusted with Benjamini–Hochberg procedure controlling false discovery rate (FDR < 0.2). Multiple pairwise comparisons against the reference GT were tested using Dunnett's type of test with control of mixed directional FDR (mdFDR) using Holm–Bonferroni correction. ANCOM-BC2-selected KOs (with or without covariate adjustment) were enriched with KEGG pathways using MicrobiomeProfiler v1.8.0.

**2.10 Single-Cell Preparation and scRNA-seq Data Acquisition**

Freshly excised tissues were washed with HBSS, kept in MACS® Tissue Storage Solution (Miltenyi Biotec, Germany) at 4 °C overnight as in previous studies (Schütz et al., 2023) and shipped on the next day of surgery to BGI (Hong Kong) for single-cell preparation and library construction. Single cell suspensions were generated using the Tumor Dissociation Kit, human (Miltenyi Biotec) on a gentleMACS™ Dissociator (Miltenyi Biotec). For PT, the 'h_skin_01' program was selected and performed at 37℃, 45min, while for other tissues, the 'h_tumor_01' was selected with the same temperature and time settings. A total of three samples for GT, PT, and ST (one sample/tissue) from different patients were included in the study. The single-cell suspensions were filtered through a Falcon® 40 μm Cell Strainer (Corning, USA), counted and examined for viability (>80%) using a hemocytometer (Table S20).

Single cells were encapsulated into GEMs (Gel Bead in Emulsion) on a Chromium™ Controller using the Chromium Next GEM Single Cell 3' Reagent Kits v3.1 (10× Genomics, USA), with the following cell lysis and reverse transcription to 10×-barcoded cDNA inside. After post-GEM-RT cleanup, cDNAs were purified using SPRIselect Bead-Based Reagent (Beckman Coulter, USA) and amplified on an Applied Biosystems SimpliAmp Thermal Cycler

(Thermo Fisher, USA). The products were further fragmented, end-repaired, A-tailed, ligated with adapters, amplified with sample index PCR and SPRIselect-purified. The final libraries, containing barcode, UMI and cDNA, were sequenced on the DNBSEQ-G400 (BGI, China). Raw data were preprocessed with Cell Ranger v5.0.1 analysis pipeline (Zheng et al., 2017) (https://support.10xgenomics.com/single-cell-gene-expression/software/overview/welcome) for demultiplexing, barcode processing, genome alignment (GRCh38 v2020-A) and single-cell 3' gene counting (UMI counts). The sequencing was achieved with >340 million reads per sample. A total of 8,100, 4,000, and 4,000 cells were obtained in GT, PT, and ST samples, respectively. RTs failed to be sequenced due to their limitation of sample volume size.

The aligned reads were processed to generate a single-cell expression matrix, which was then imported using the *read10X* function for analysis by the *Seurat* package v5.0 (Hao et al., 2024). Cells expressing genes in fewer than three cells were removed, as well as cells characterized by mitochondrial gene expression above 25% or fewer than 100 detected genes. The top 2,000 variable genes were identified using *FindVariableGenes* function of *Seurat*, followed by PCA. Dimensionality reduction and visualization were achieved using the Uniform Manifold Approximation and Projection (UMAP), with identification of cell types based on known marker genes for oral mucosa (Williams et al., 2021) and previous studies (Behm et al., 2024; Liu et al., 2023; Morgan & Tergaonkar, 2022; Shi et al., 2023; Xie et al., 2020).

**2.11 Differential Expression and Pathway Analysis**

Differential expression analysis was carried out using the *FindMarkers* function in *Seurat*, targeting only genes that were detected in at least 25% of cells from each of the analyzed populations. The analysis was specifically limited to genes exhibiting an average log-fold change of at least 0.25. To delve into the biological implications of the differentially expressed genes (DEGs), Gene Ontology (GO) and KEGG pathway enrichment analyses were conducted using the *clusterProfiler* R package v4.10.1 (Wu et al., 2021) with significance defined by an adjusted $P$-value < 0.05.

**2.12 Pseudotime Trajectory Analysis**

Using the Monocle3 v3.0.2 package (Cao et al., 2019; Trapnell et al., 2014), pseudotime analysis was conducted on trajectories. A *Seurat* object, containing subsets of MSCs, endothelial cells, fibroblasts, and epithelial cells, was imported into Monocle. The analysis was performed on the normalized counts matrix, with cell clustering and dimensional reduction facilitated by UMAP. The principal trajectory graph was constructed using the *learn_graph*

function, and key genes were visualized over pseudotime in a heatmap using the *plot_pseudotime_heatmap* function.

**2.13 Histology and Immunohistochemistry Analysis**

Freshly excised tissues were washed with HBSS and fixed in 10% neutral buffer formalin solution at 4 °C overnight, followed by dehydration using a series of ethanol until three times of 100% ethanol. Ethanol within tissues was then exchanged with xylene three times, and xylene was further exchanged with paraffin twice for embedding. The paraffin blocks were sectioned at a thickness of 4 μm, de-paraffinized, and rehydrated. For histology, the slides were stained with Gill's Hematoxylin and 1% Eosin Y solution. For IHC, antigen retrieval of the section was achieved with EDTA buffer, pH 8, for 5 min using a pressure heat-induced method. The slides were incubated with the corresponding primary antibody at 4 °C overnight and detected using UltraVision Quanto Detection System HRP DAB (Thermo Fisher) at room temperature. Primary antibodies: CD4 (#NBP1-19371, 1:500, Novus Biologicals), and NKG2D/CD314 (#NB100-65956, 1:500, Novus Biologicals). H&E and IHC images were analyzed with the open-source digital pathology suite QuPath v0.5.1 (Bankhead et al., 2017). For cell detection, each slide was automatically added with square annotation areas at a tileSize of 4196 by running the official script (https://qupath.readthedocs.io/), which could generate thousands of cell detections.

For H&E images, cell detection was automatically performed by QuPath using the default setting of Hematoxylin OD thresholding and Nucleus Eosin OD mean. Inflammatory infiltrate of tiles in each slide was semi-quantified using QuPath with an object classifier (artificial neural network, ANN_MLP) trained on self-defined regions of interest (ROIs), both with and without infiltrate. The percentage of infiltration was calculated by dividing the number of positively classified cells by the total number of detected cells. The overall correctness of classification was confirmed visually.

For cell detection of IHC, positive cells were detected using "Positive cell detection" function with the setting of the default three-level threshold in "Intensity threshold parameter" at "Score compartment" of "Cell: DAB OD mean" for markers. The positiveness was further semi-quantified using both immunoreactivity score (IRS) and H-score. The staining intensity score (*I*) was scored on a scale of 0–3 (0, negative; 1+, weak; 2+, moderate; and 3+, strong) based on the default three-level threshold of QuPath. The positive cell proportion score (*P*) was

scored as follows: 0, no staining; 1, 1–25%; 2, 26–50%; 3, 51–75%; and 4, 76–100% stained cells.

$$IRS = \sum_{i=1}^{3} I_i \times P_i$$

H-score of individual annotation area was calculated automatically by QuPath, adding the multiplication of the different staining intensities *I* (0–3) with the percentage of positive cells, i.e., H-score (0–300 scale) = 3 × (% at 3 +) + 2 × (% at 2+) + 1 × (% at 1+).

### 2.14 Statistical analysis

The statistical tests implemented in 16S rRNA seq and scRNA-seq were performed within the corresponding R packages. The inter-tissue difference of taxon relative abundance was assessed using Kruskal-Wallis test. The inter-tissue difference of histological analysis was examined using Krusakl−Wallis test with *post hoc* Dunn's test controlling false discovery rate using Benjamini−Hochberg method.

### 2.15 Sample size (*a priori* and *post hoc*)

There was limited published intra-tissue study for periodontal tissue available. Most studies investigated oral bacteriomes of the subgingival plaque, saliva, and surface swabs of periodontitis patients. Referring to a previous 16S rRNA seq study comparing periodontally healthy and diseased gingivae from five patients with Stage III periodontitis (Bao et al., 2020), the relevant abundance (mean and SD) of two top OTUs are: *Streptococcus vestibularis* (Healthy sites 0.417 ± 0.029; Diseased sites 0.169 ± 0.114) and *Treponema* sp. HMT_253 (Healthy sites 0.033 ± 0.035; Diseased sites 0.301 ± 0.176). An *a priori* calculation using t-test was performed utilizing G*Power v3.1.9.7 (Heinrich Heine University Düsseldorf, Germany) (Faul et al., 2009). Based on the results of these two OTUs, 4 and 6 samples per group are needed to achieve a 5% level of significance and 95% detection power for *S. vestibularis* and *Treponema* sp. HMT_253, respectively.

A *post hoc* calculation was performed. For 16S rRNA seq (27 GTs, 13 PTs, 8 RTs, and 11 STs), the sample sizes were estimated based on the x-axis data of Bray–Curtis dissimilarity and P/R ratio, respectively (table below). Effect size f for F test of one-way fixed effects ANOVA was calculated using mean and n of each type of tissue and maxima of SD. A power of 75% and 95% was achieved for Bray–Curtis dissimilarity and P/R ratio, respectively.

Summary of parameters of 16S rRNA seq for *post hoc* sample size calculation

| Characteristic | GT<br>N = 27[1] | PT<br>N = 13[1] | RT<br>N = 8[1] | ST<br>N = 11[1] | p-value[2] |
|---|---|---|---|---|---|
| Bray–Curtis (X) | 0.06 (0.26) | 0.12 (0.20) | -0.22 (0.17) | -0.12 (0.28) | 0.010 |
| PR_ratio (log2) | -5.7 (5.2) | -7.1 (4.5) | 0.7 (4.9) | -0.8 (4.0) | 0.003 |

[1] Mean (SD); [2] Kruskal-Wallis rank sum test

There was no previously published scRNA-seq study specific to periodontal granulation tissue available. Only one preprint study of scRNA-seq of granulation tissue pooled from three periodontitis patients (doi:10.1101/2025.01.13.632112) was noticed with 6,729 sequenced cells, but without available data. Thus, a prospective sample size of cells was calculated using SCOPIT (https://alexdavisscs.shinyapps.io/scs_power_multinomial/) (Davis et al., 2019) based on the lowest proportion of cells reported in the previously reported human oral mucosa cell atlas (Williams et al., 2021). The lowest proportion of cell type is average of 0.004. We set 'Frequency of rarest subpopulation' of 0.002 and '# of subpopulations with the lowest frequency' of 3 with other parameters of defaults. Totally, 3,863 cells were required for 0.95 probability of success. We finally sequenced 8,100 (GT), 4,000 (PT), and 4,000 (ST) cells.

The rarest cell type in GT is neutrophil with cell number of 59. The second rarest is NK cell with cell number of 75. Thus, a retrospective calculation presented 903 (95% CI: 1,091) cells required for 0.95 probability of success.

The rarest cell type in PT is epithelial cell with cell number of 11. The second rarest is macrophage with cell number of 42. Thus, a retrospective calculation presented 2,323 (95% CI: 4,442) cells required for 0.95 probability of success.

The rarest cell type in ST is epithelial cell with cell number of 7. The second rarest is macrophage with cell number of 8. Thus, a retrospective calculation presented 3,922 (95% CI: 8,904) cells required for 0.95 probability of success.

Hitherto, majority of scRNA-seq studies have primarily focused on understanding cellular heterogeneity, often using limited samples (Agrafioti et al., 2022). In the current study, we explored the sample collection and preparation for scRNA-seq as proof-of-concept. As the volume of most granulation tissue was relatively small, the availability of granulation tissue with qualified volume for scRNA-seq was uncertain. We will expand the sample size of biopsies in future study of larger sample cohorts.

## 4 Supplementary Discussion

Studies have demonstrated that pericytes serve as the primary source of MSCs *in vivo*, particularly during tissue injury and repair, where they become activated and transition into functionally reparative MSCs (Yianni & Sharpe, 2019). Given that granulation tissue forming within periodontal defects during proliferative phrase in an attempt to repair defect site, our identified MSC population may contain activated pericyte population. We investigated pericyte markers such as *CXCL12* (Chen et al., 2022), but its expression was observed across multiple cell types, including clusters of endothelial cells, fibroblasts, and MSCs (Figs. S14−S15). This lack of a distinct expression pattern, limited by sample size and capture efficiency, made it difficult to computationally resolve a distinct and stable pericyte cluster.

## Supplementary Information

Detailed anonymous anthropological, clinical and periodontal information for all participants for all participants is provided in an additional Excel file.

## References


Agrafioti, P., Morin-Baxter, J., Tanagala, K. K. K., Dubey, S., Sims, P., Lalla, E., & Momen-Heravi, F. (2022). Decoding the role of macrophages in periodontitis and type 2 diabetes using single-cell RNA-sequencing. *FASEB Journal, 36*(2), e22136. doi:10.1096/fj.202101198R

Bankhead, P., Loughrey, M. B., Fernandez, J. A., Dombrowski, Y., McArt, D. G., Dunne, P. D., . . . Hamilton, P. W. (2017). QuPath: Open source software for digital pathology image analysis. *Scientific Reports, 7*(1), 16878. doi:10.1038/s41598-017-17204-5

Bao, K., Li, X., Poveda, L., Qi, W., Selevsek, N., Gumus, P., . . . Belibasakis, G. N. (2020). Proteome and microbiome mapping of human gingival tissue in health and disease. *Front Cell Infect Microbiol, 10*, 588155. doi:10.3389/fcimb.2020.588155

Behm, C., Milek, O., Schwarz, K., Kovar, A., Derdak, S., Rausch-Fan, X., . . . Andrukhov, O. (2024). Heterogeneity in dental tissue-derived MSCs revealed by single-cell RNA-seq. *Journal of Dental Research, 103*(11), 1141–1152. doi:10.1177/00220345241271997

Callahan, B. J., McMurdie, P. J., Rosen, M. J., Han, A. W., Johnson, A. J., & Holmes, S. P. (2016). DADA2: High-resolution sample inference from Illumina amplicon data. *Nature Methods, 13*(7), 581–583. doi:10.1038/nmeth.3869

Cao, J., Spielmann, M., Qiu, X., Huang, X., Ibrahim, D. M., Hill, A. J., . . . Shendure, J. (2019). The single-cell transcriptional landscape of mammalian organogenesis. *Nature, 566*(7745), 496–502. doi:10.1038/s41586-019-0969-x

Carnevale, G., & Kaldahl, W. B. (2000). Osseous resective surgery. *Periodontology 2000, 22*, 59–87. doi:10.1034/j.1600-0757.2000.2220106.x

Chen, Y., Wang, H., Yang, Q., Zhao, W., Chen, Y., Ni, Q., . . . Sun, W. (2022). Single-cell RNA landscape of the osteoimmunology microenvironment in periodontitis. *Theranostics, 12*(3), 1074–1096. doi:10.7150/thno.65694

Cortellini, P., Stalpers, G., Mollo, A., & Tonetti, M. S. (2011). Periodontal regeneration versus extraction and prosthetic replacement of teeth severely compromised by attachment loss to the apex: 5-year results of an ongoing randomized clinical trial. *Journal of Clinical Periodontology, 38*(10), 915–924. doi:10.1111/j.1600-051X.2011.01768.x

Cortellini, P., & Tonetti, M. S. (2007). A minimally invasive surgical technique with an enamel matrix derivative in the regenerative treatment of intra-bony defects: a novel approach to limit morbidity. *J Clin Periodontol, 34*(1), 87–93. doi:10.1111/j.1600-051X.2006.01020.x

Cortellini, P., & Tonetti, M. S. (2009). Improved wound stability with a modified minimally invasive surgical technique in the regenerative treatment of isolated interdental intrabony defects. *J Clin Periodontol, 36*(2), 157–163. doi:10.1111/j.1600-051X.2008.01352.x

Davis, A., Gao, R., & Navin, N. E. (2019). SCOPIT: sample size calculations for single-cell sequencing experiments. *BMC Bioinformatics, 20*(1), 566. doi:10.1186/s12859-019-3167-9

Douglas, G. M., Maffei, V. J., Zaneveld, J. R., Yurgel, S. N., Brown, J. R., Taylor, C. M., . . . Langille, M. G. I. (2020). PICRUSt2 for prediction of metagenome functions. *Nature Biotechnology, 38*(6), 685–688. doi:10.1038/s41587-020-0548-6

Faul, F., Erdfelder, E., Buchner, A., & Lang, A. G. (2009). Statistical power analyses using G*Power 3.1: tests for correlation and regression analyses. *Behavior Research Methods, 41*(4), 1149–1160. doi:10.3758/BRM.41.4.1149

Hao, Y., Stuart, T., Kowalski, M. H., Choudhary, S., Hoffman, P., Hartman, A., . . . Satija, R. (2024). Dictionary learning for integrative, multimodal and scalable single-cell analysis. *Nature Biotechnology, 42*(2), 293–304. doi:10.1038/s41587-023-01767-y

Herrera, D., Sanz, M., Kebschull, M., Jepsen, S., Sculean, A., Berglundh, T., . . . EFP Workshop Participants Methodological, Consultant. (2022). Treatment of stage IV periodontitis: The EFP S3 level clinical practice guideline. *Journal of Clinical Periodontology, 49*(Suppl 24), 4–71. doi:10.1111/jcpe.13639

Lang, N. P., Tan, W. C., Krahenmann, M. A., & Zwahlen, M. (2008). A systematic review of the effects of full-mouth debridement with and without antiseptics in patients with chronic periodontitis. *Journal of Clinical Periodontology, 35*(8 Suppl), 8–21. doi:10.1111/j.1600-051X.2008.01257.x

Lin, H., & Peddada, S. D. (2024). Multigroup analysis of compositions of microbiomes with covariate adjustments and repeated measures. *Nature Methods, 21*(1), 83–91. doi:10.1038/s41592-023-02092-7

Ling, W., Lu, J., Zhao, N., Lulla, A., Plantinga, A. M., Fu, W., . . . Wu, M. C. (2022). Batch effects removal for microbiome data via conditional quantile regression. *Nature Communications, 13*(1), 5418. doi:10.1038/s41467-022-33071-9

Liu, J., Li, T., Zhang, S., Lu, E., Qiao, W., Chen, H., . . . Chen, H. (2023). Proteomic and single-cell analysis shed new light on the anti-inflammatory role of interferonβ in chronic periodontitis. *Frontiers in Pharmacology, 14*, 1232539. doi:10.3389/fphar.2023.1232539

Mallick, H., Rahnavard, A., McIver, L. J., Ma, S., Zhang, Y., Nguyen, L. H., . . . Huttenhower, C. (2021). Multivariable association discovery in population-scale meta-omics studies. *PLoS Computational Biology, 17*(11), e1009442. doi:10.1371/journal.pcbi.1009442

Miki, K., Kitamura, M., Hatta, K., Kamide, K., Gondo, Y., Yamashita, M., . . . Murakami, S. (2021). Periodontal inflamed surface area is associated with hs-CRP in septuagenarian Japanese adults in cross-sectional findings from the SONIC study. *Scientific Reports, 11*(1), 14436. doi:10.1038/s41598-021-93872-8

Morgan, D., & Tergaonkar, V. (2022). Unraveling B cell trajectories at single cell resolution. *Trends in Immunology, 43*(3), 210–229. doi:10.1016/j.it.2022.01.003

Nesse, W., Abbas, F., van der Ploeg, I., Spijkervet, F. K., Dijkstra, P. U., & Vissink, A. (2008). Periodontal inflamed surface area: quantifying inflammatory burden. *Journal of Clinical Periodontology, 35*(8), 668–673. doi:10.1111/j.1600-051X.2008.01249.x

Quast, C., Pruesse, E., Yilmaz, P., Gerken, J., Schweer, T., Yarza, P., . . . Glockner, F. O. (2013). The SILVA ribosomal RNA gene database project: improved data processing and web-based tools. *Nucleic Acids Research, 41*(Database issue), D590–596. doi:10.1093/nar/gks1219

Rohart, F., Gautier, B., Singh, A., & Lê Cao, K. A. (2017). mixOmics: An R package for 'omics feature selection and multiple data integration. *PLoS Computational Biology, 13*(11), e1005752. doi:10.1371/journal.pcbi.1005752

Sanz, M., Herrera, D., Kebschull, M., Chapple, I., Jepsen, S., Beglundh, T., . . . EFP Workshop Participants Methodological, Consultants. (2020). Treatment of stage I–III periodontitis—The EFP S3 level clinical practice guideline. *Journal of Clinical Periodontology, 47*(Suppl 22), 4–60. doi:10.1111/jcpe.13290

Schütz, S., Solé-Boldo, L., Lucena-Porcel, C., Hoffmann, J., Brobeil, A., Lonsdorf, A. S., . . . Lyko, F. (2023). Functionally distinct cancer-associated fibroblast subpopulations establish a tumor promoting environment in squamous cell carcinoma. *Nature Communications, 14*(1), 5413. doi:10.1038/s41467-023-41141-9

Shi, G., Yang, C., Zhou, L., Zong, M., Guan, Q., da Roza, G., . . . Du, C. (2023). Comprehensive cell surface protein profiling of human mesenchymal stromal cells from peritoneal dialysis effluent and comparison with those from human bone marrow and adipose tissue. *Human Cell, 36*(6), 2259–2269. doi:10.1007/s13577-023-00971-x

Tonetti, M. S., Eickholz, P., Loos, B. G., Papapanou, P., van der Velden, U., Armitage, G., . . . Suvan, J. E. (2015). Principles in prevention of periodontal diseases: Consensus report of group 1 of the 11th European Workshop on Periodontology on effective prevention of periodontal and peri-implant diseases. *Journal of Clinical Periodontology, 42 Suppl 16*, S5–S11. doi:10.1111/jcpe.12368

Trapnell, C., Cacchiarelli, D., Grimsby, J., Pokharel, P., Li, S., Morse, M., . . . Rinn, J. L. (2014). The dynamics and regulators of cell fate decisions are revealed by pseudotemporal ordering of single cells. *Nature Biotechnology, 32*(4), 381–386. doi:10.1038/nbt.2859

WHO Expert Consultation. (2004). Appropriate body-mass index for Asian populations and its implications for policy and intervention strategies. *Lancet, 363*(9403), 157–163. doi:10.1016/S0140-6736(03)15268-3

Williams, D. W., Greenwell-Wild, T., Brenchley, L., Dutzan, N., Overmiller, A., Sawaya, A. P., . . . Moutsopoulos, N. M. (2021). Human oral mucosa cell atlas reveals a stromal-neutrophil axis regulating tissue immunity. *Cell, 184*(15), 4090–4104.e4015. doi:10.1016/j.cell.2021.05.013

Wu, T., Hu, E., Xu, S., Chen, M., Guo, P., Dai, Z., . . . Yu, G. (2021). clusterProfiler 4.0: A universal enrichment tool for interpreting omics data. *Innovation, 2*(3), 100141. doi:10.1016/j.xinn.2021.100141

Xie, X., Shi, Q., Wu, P., Zhang, X., Kambara, H., Su, J., . . . Luo, H. R. (2020). Single-cell transcriptome profiling reveals neutrophil heterogeneity in homeostasis and infection. *Nature Immunology, 21*(9), 1119–1133. doi:10.1038/s41590-020-0736-z

Yianni, V., & Sharpe, P. T. (2019). Perivascular-derived mesenchymal stem cells. *Journal of Dental Research, 98*(10), 1066–1072. doi:10.1177/0022034519862258

Zheng, G. X., Terry, J. M., Belgrader, P., Ryvkin, P., Bent, Z. W., Wilson, R., . . . Bielas, J. H. (2017). Massively parallel digital transcriptional profiling of single cells. *Nature Communications, 8*, 14049. doi:10.1038/ncomms14049

## Supplementary Figures

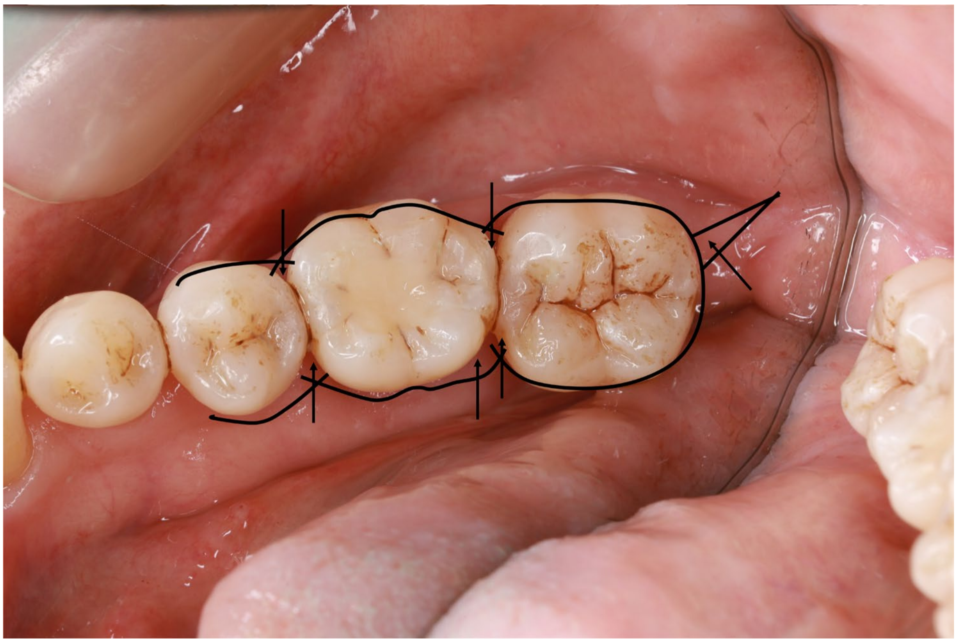

**Supplementary Figure 1. Representative photo illustrations of the locations of collecting inflamed gingival tissue (PT).** Incision made when collecting PT samples during periodontal resective surgeries (black lines). The arrowed parts were the soft tissue being excised and collected as PT sample.

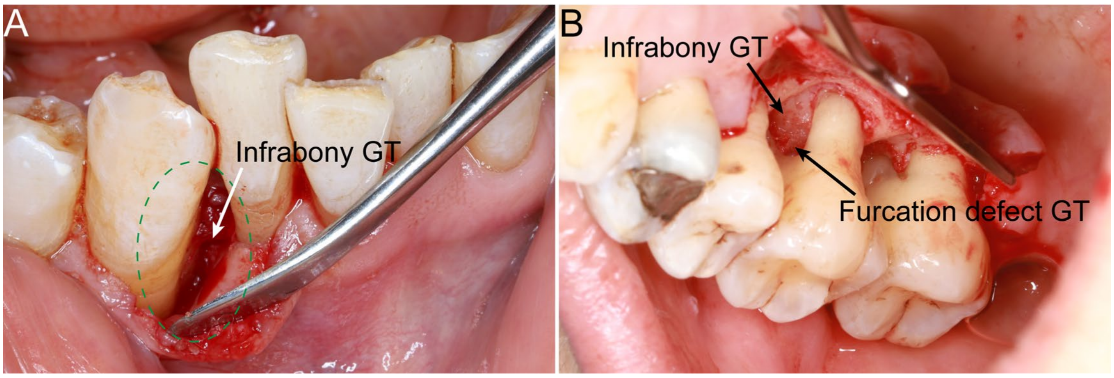


**Supplementary Figure 2. Representative photo illustrations of the locations of collecting osseous defect granulation tissue (GT).** (A) Infrabony granulation tissue. (B) Combined granulation tissue from infrabony and furcation osseous defects.

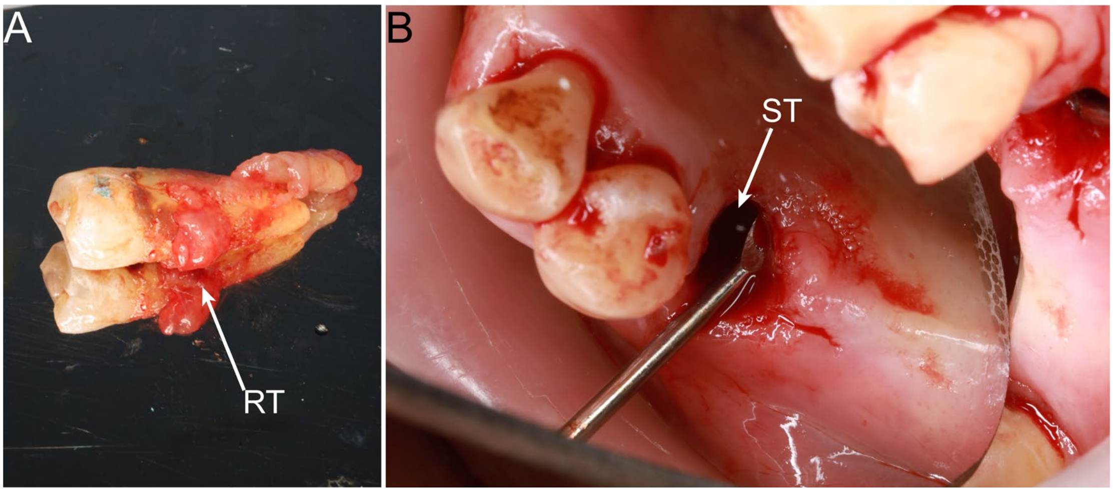


**Supplementary Figure 3. Representative photo illustrations of the locations of collecting RT and ST.** (A) Granulation tissue attached to root surface of extracted tooth (RT). (B) Granulation tissue from extraction socket (ST).

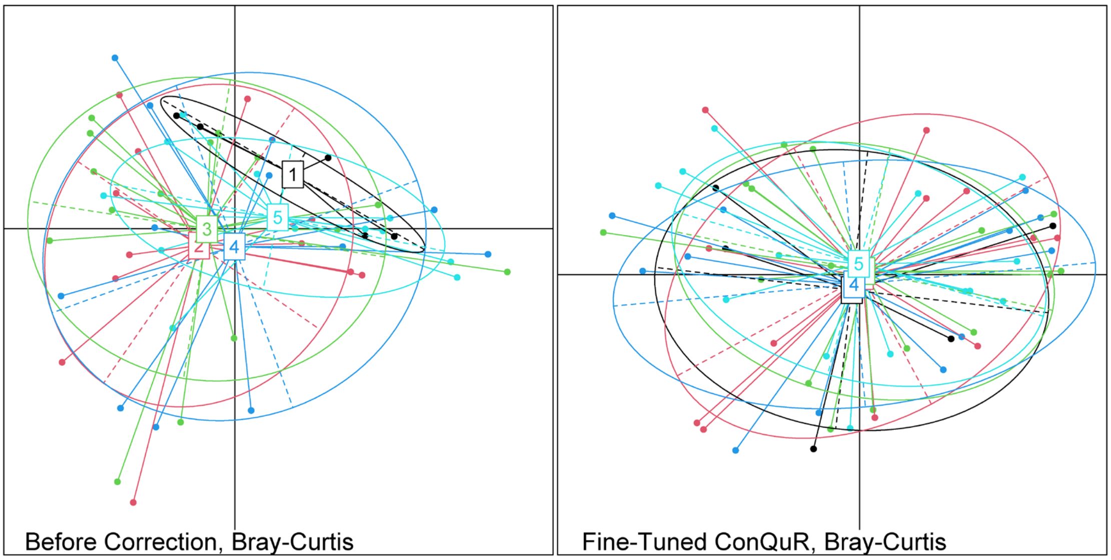


**Supplementary Figure 4. Batch effect removal.** The comparison of PCoA (Bray-Curtis dissimilarity) before (left panel) and after (right panel) batch effect removal using fine-tuned ConQuR with covariates.

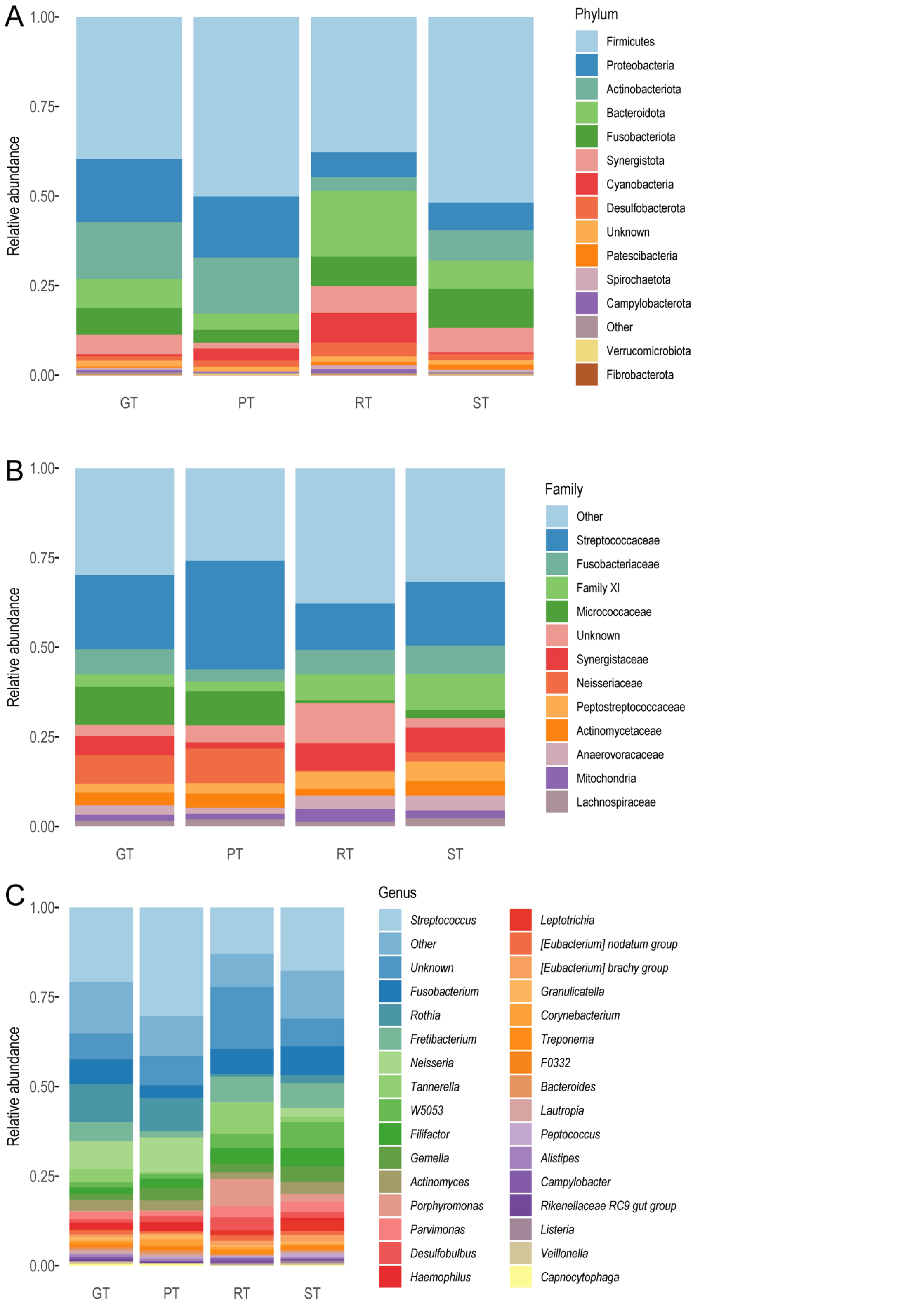


**Supplementary Figure 5. Relative abundance of microbiota from different types of tissues.** Average relative abundance of phyla (A), families (B) and genera (C). Detection threshold: 0.01; prevalence threshold: 10%. GT: osseous granulation tissue, PT: periodontal tissue; RT: root granulation tissue; ST: socket granulation tissue.

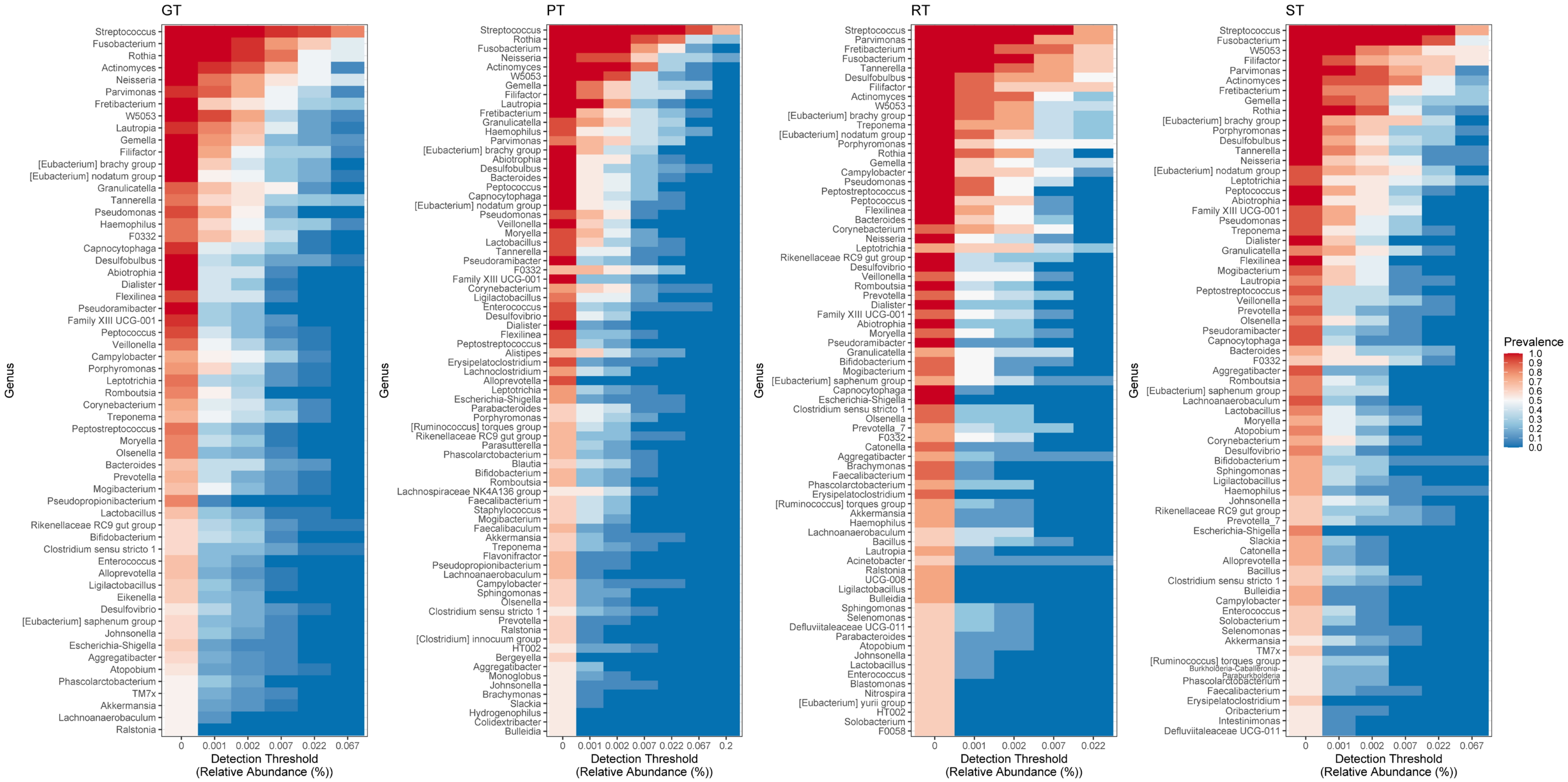


**Supplementary Figure 6. Core microbiome genera of different types of tissue.** GT: osseous granulation tissue, PT: periodontal tissue; RT: root granulation tissue; ST: socket granulation tissue.

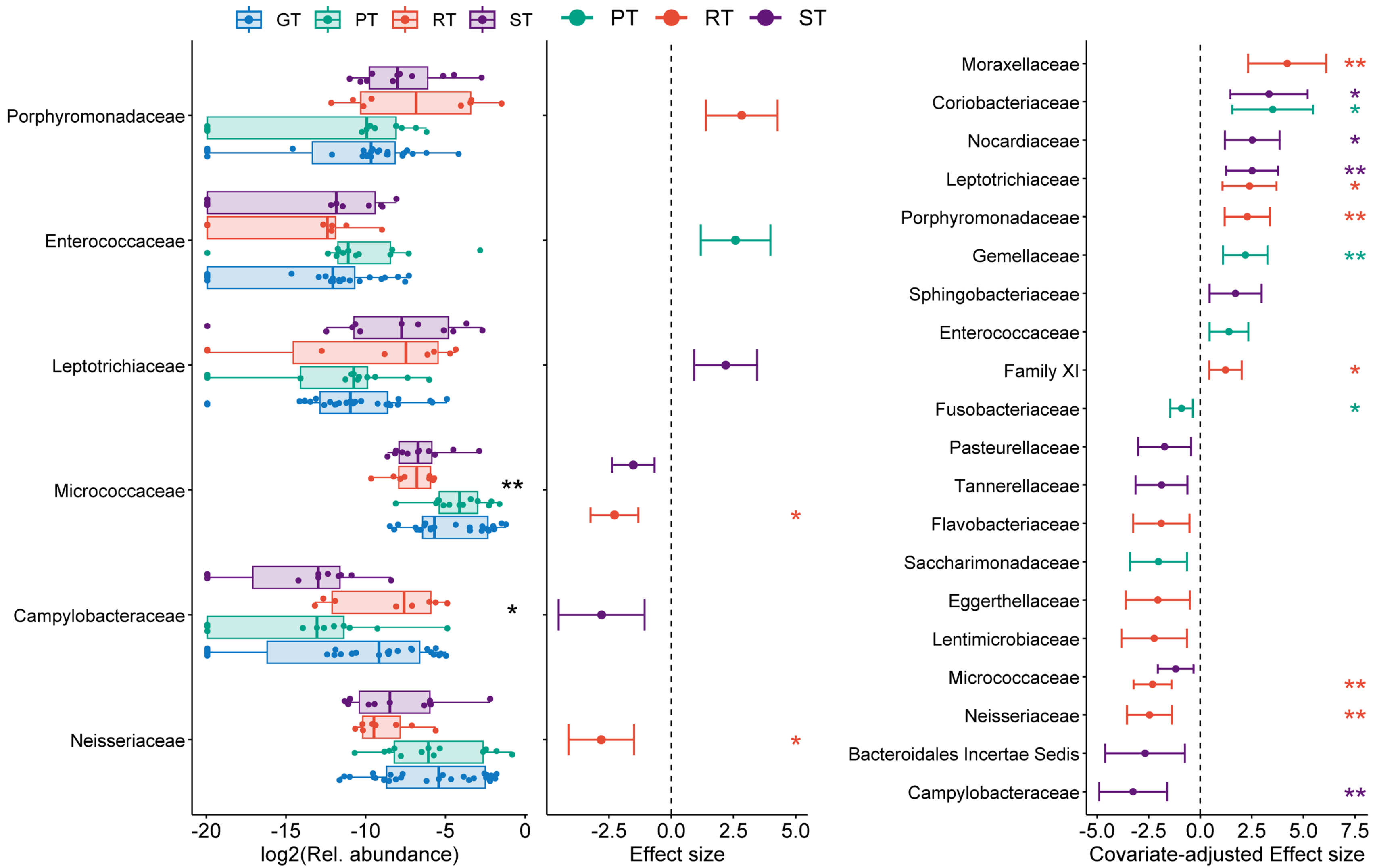


**Supplementary Figure 7. Multivariable statistical analysis to identify taxa associated with tissue types at family level.** MaAsLin2 was utilized to analyze the dataset with or without the adjustment of covariates (sex, age, obese, smoker and DM status) using GT as the reference. GT: osseous granulation tissue, PT: periodontal tissue; RT: root granulation tissue; ST: socket granulation tissue. The taxa with $q < 0.25$ or $q < 0.1$ (for covariate adjustment) are shown. *, $q < 0.05$; **, $q < 0.01$; ***, $q < 0.001$. MaAsLin2 effect sizes are shown in dot plot with error bars representing 95% confidence intervals. The $log_2$-transformed relative abundance of selected covariate-unadjusted families are also demonstrated in box plot, with significance of Kruskal-Wallis test shown. *, $P < 0.05$; **, $P < 0.01$; ***, $P < 0.001$. The families with more than 7 non-zero count samples were shown.

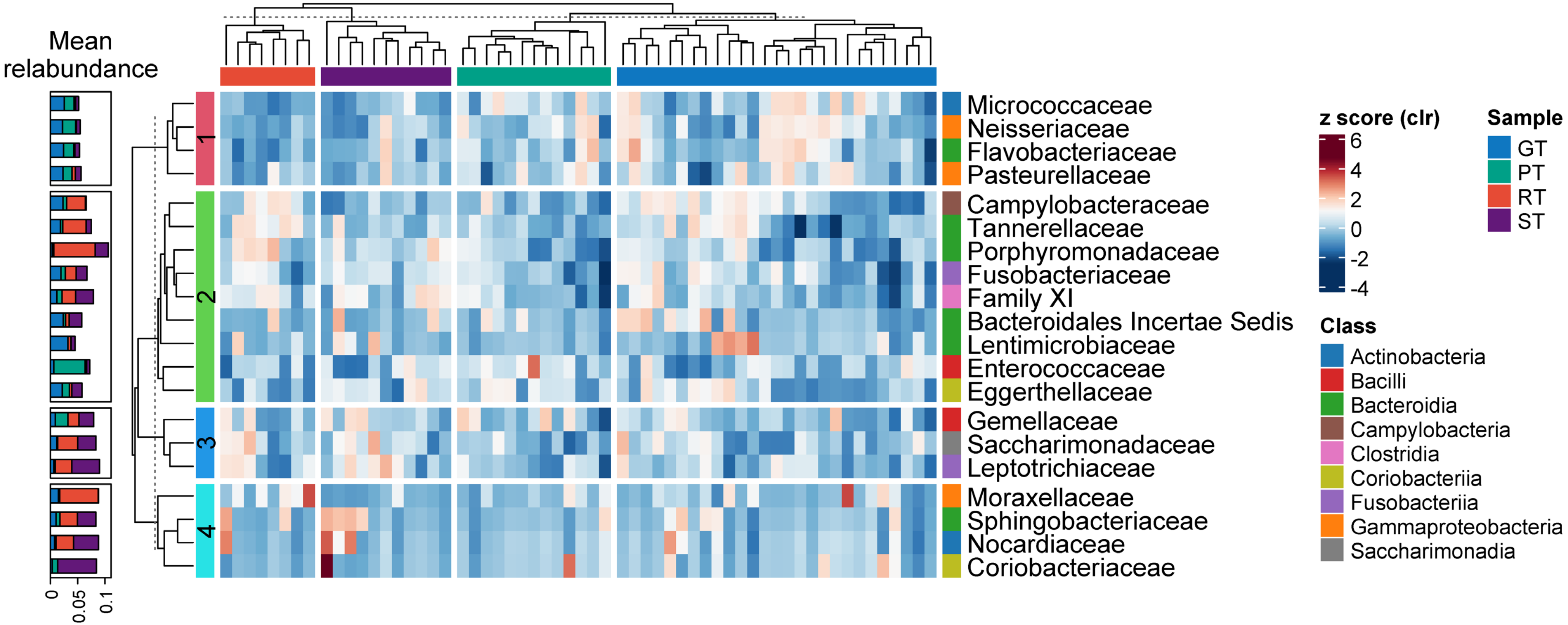


**Supplementary Figure 8. Heatmap and clustering of MaAsLin2-selected families.** Both with ($q < 0.1$) and without ($q < 0.25$) covariate-adjusted selected families are included in plotting the heatmap using z-score of clr. Families were clustered into four *k*-means clusters. The mean relative abundance of each family for each type of tissue is also shown in the bar plot. The corresponding class of each family is also shown. The families with more than 7 non-zero count samples were shown. GT: osseous granulation tissue, PT: periodontal tissue; RT: root granulation tissue; ST: socket granulation tissue.

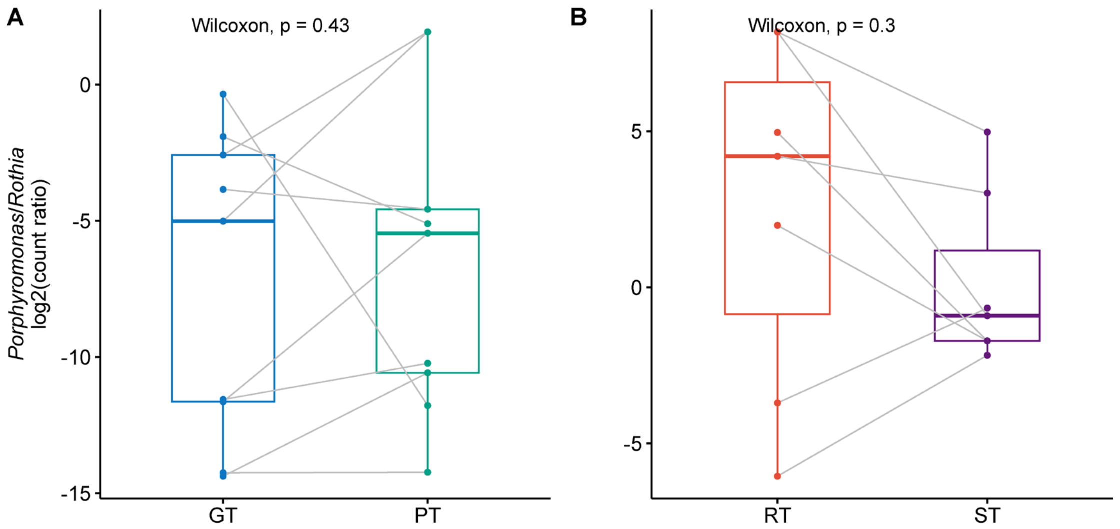


**Supplementary Figure 9. Subject-paired signature of microbial dysbiosis of GT/PT and RT/ST pairs.** Ratios ($log_2$) of the abundance (counts) of *Porphyromonas* to *Rothia* (A) between subjected paired GT and PT and (B) between subjected paired RT and ST. Paired Wilcoxon rank-sum test. GT: osseous granulation tissue, PT: periodontal tissue; RT: root granulation tissue; ST: socket granulation tissue.

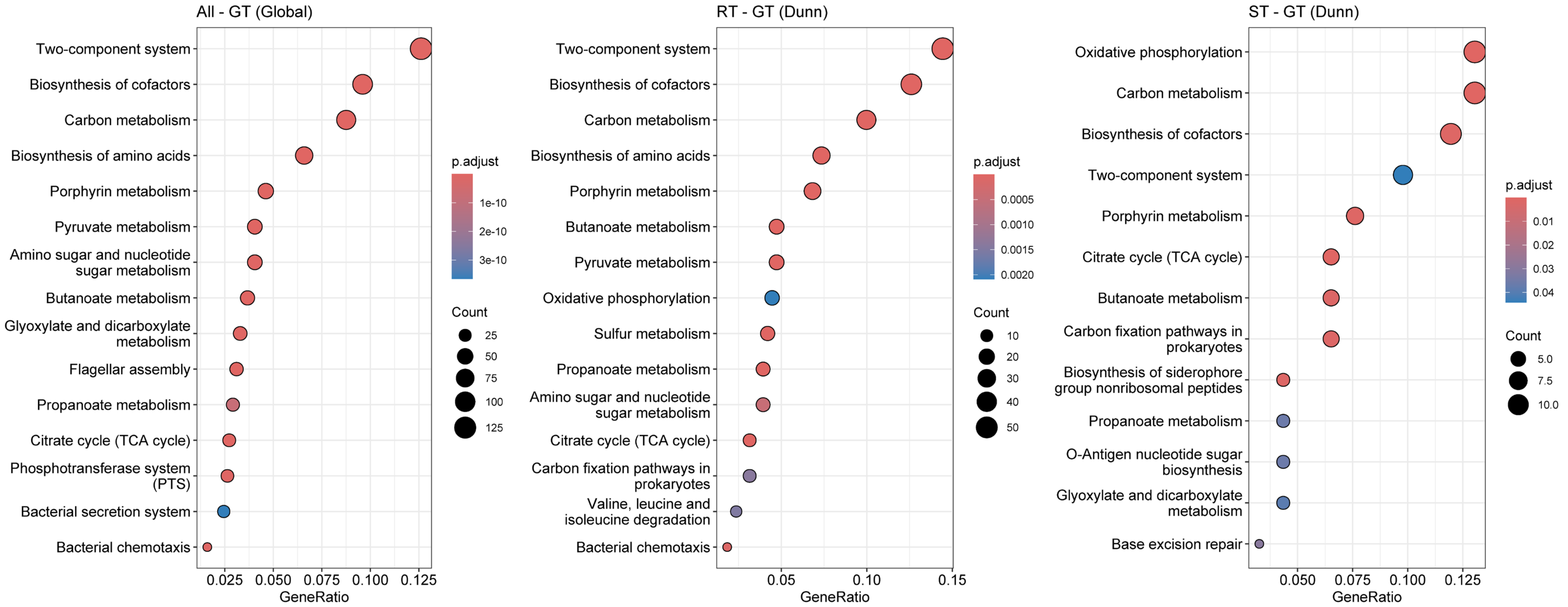


**Supplementary Figure 10. KEGG enrichment of ANCOM-BC2 selected KO terms.** The KO terms passed the sensitivity analysis for pseudo-count addition in ANCOM-BC2 global test results of PICRUSt2-prediction were enriched to KEGG pathways. Additionally, the KO terms, which passed the sensitivity analysis for pseudo-count addition in ANCOM-BC2 multiple pairwise comparisons against the reference GT of PICRUSt2-prediction with Dunnett's type (Dunn) of test with control of mixed directional FDR (mdFDR) using Holm–Bonferroni correction, were enriched to KEGG pathways. GT: osseous granulation tissue, PT: periodontal tissue; RT: root granulation tissue; ST: socket granulation tissue.

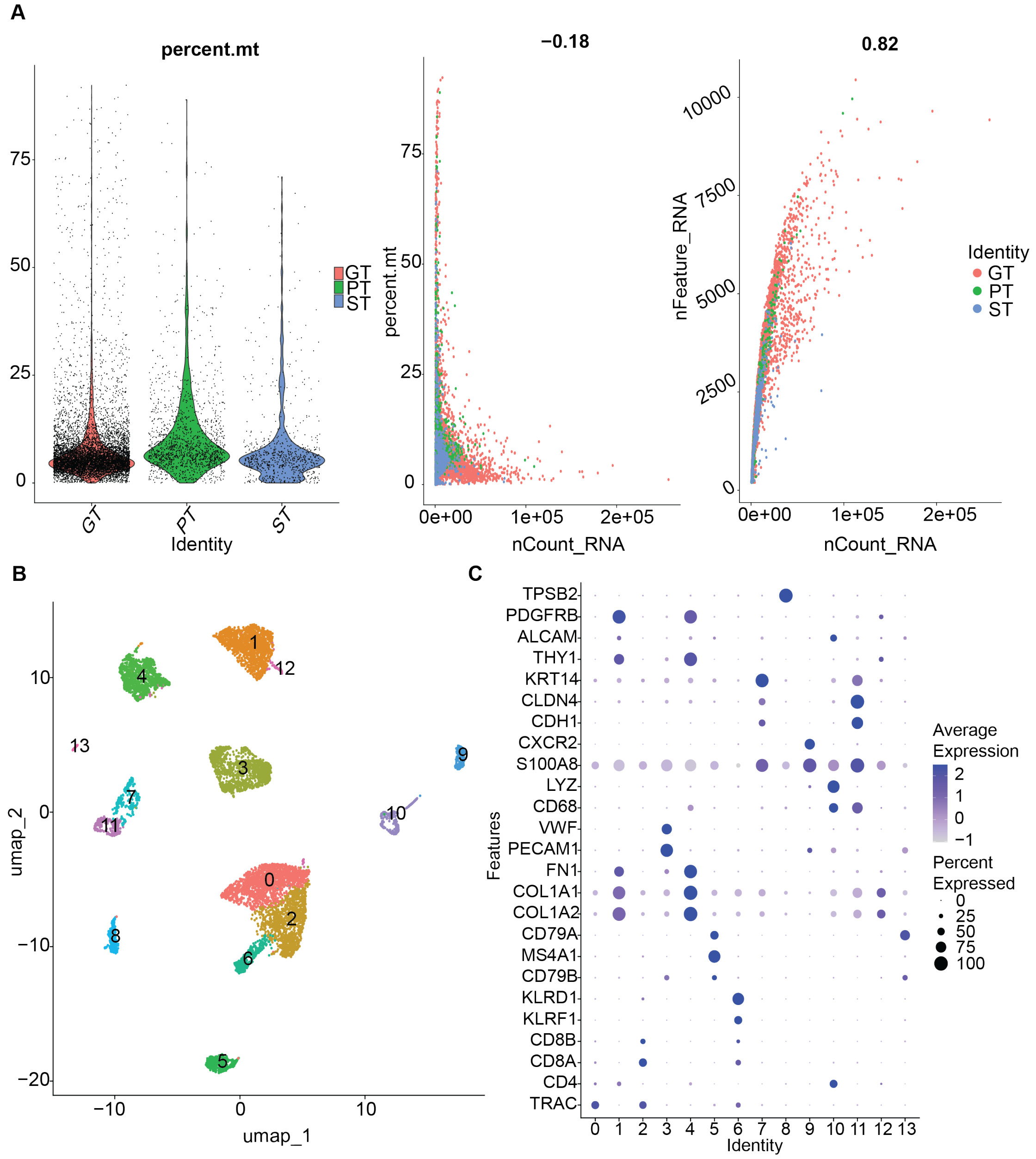


**Supplementary Figure 11. Quality control and cell annotation of scRNA-seq.** (A) Quality control of single-cell RNA sequencing for GT, PT and ST sub-populations. Parameters include the number of detected genes (nFeature_RNA), mitochondrial gene percentages (percent.mt) and total UMI counts (nCount_RNA) per cell after QC filtering. (B) UMAP visualization of clustered cells, which are color-coded. (C) The dot map depicting the average expression level (normalized and scaled averages) of marker (cluster-defining) genes and percentage of cells expressing each gene for cell clusters. GT: osseous granulation tissue, PT: periodontal tissue; RT: root granulation tissue; ST: socket granulation tissue.

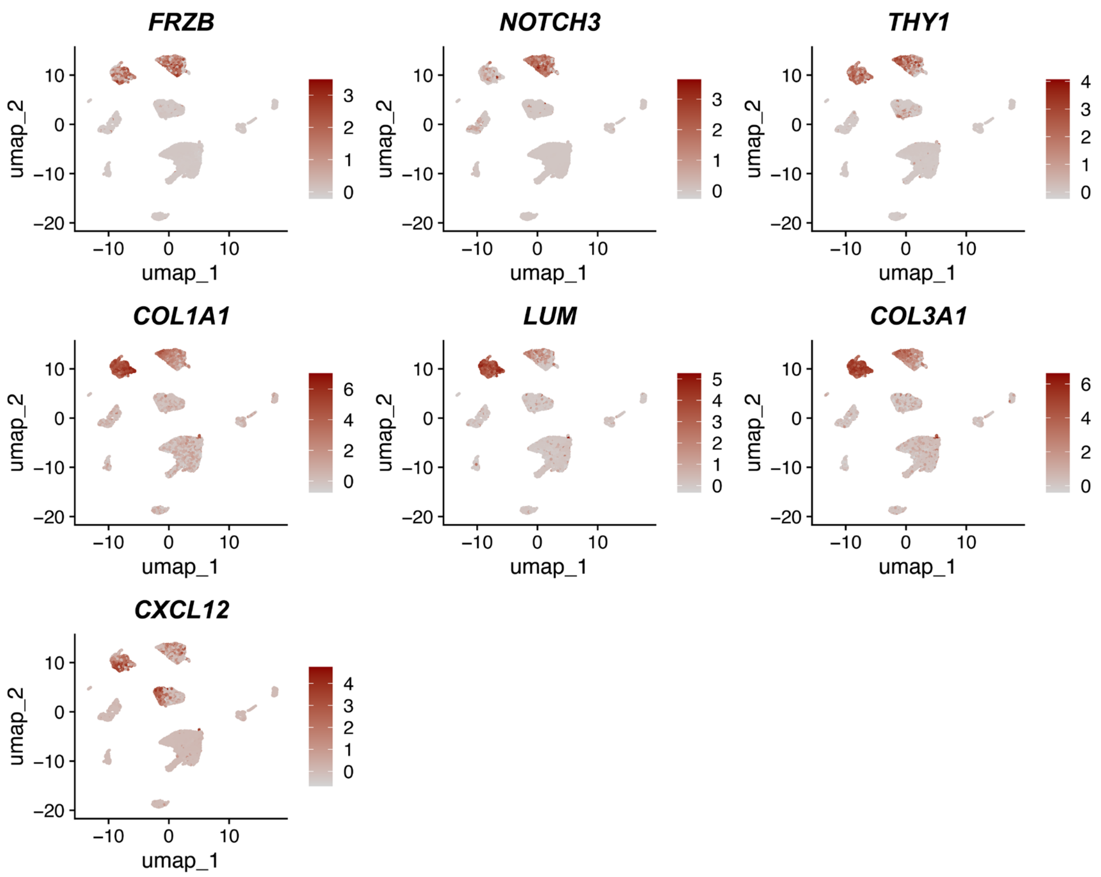


**Supplementary Figure 12. UMAP feature plots of the distribution of common marker genes.** Each cell is colored by its log-normalization of expression of corresponding gene. MSC markers: *FRZB*, *NOTCH3*, *THY1*. Fibroblast markers: *COL1A1*, *LUM*, *COL3A1*. Pericyte: *CXCL12*.

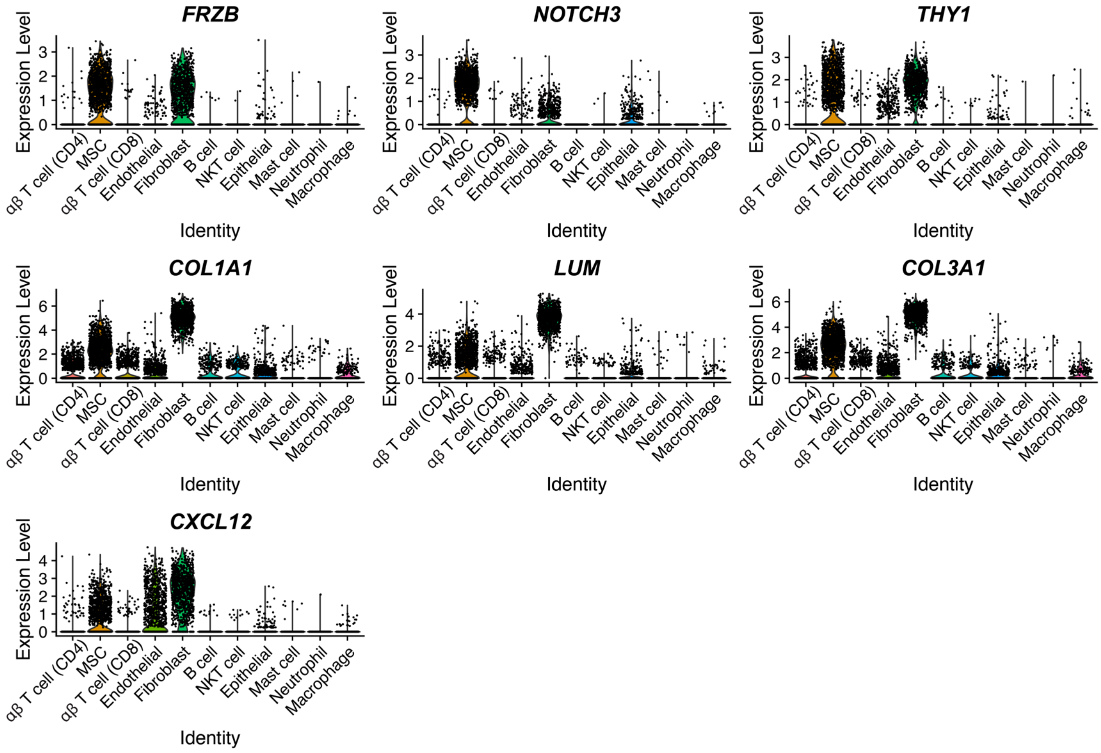


**Supplementary Figure 13. Expression levels of the distribution of common marker genes across cell types.** Expression levels are presented in log-normalization. MSC markers: *FRZB*, *NOTCH3*, *THY1*. Fibroblast markers: *COL1A1*, *LUM*, *COL3A1*. Pericyte: *CXCL12*.

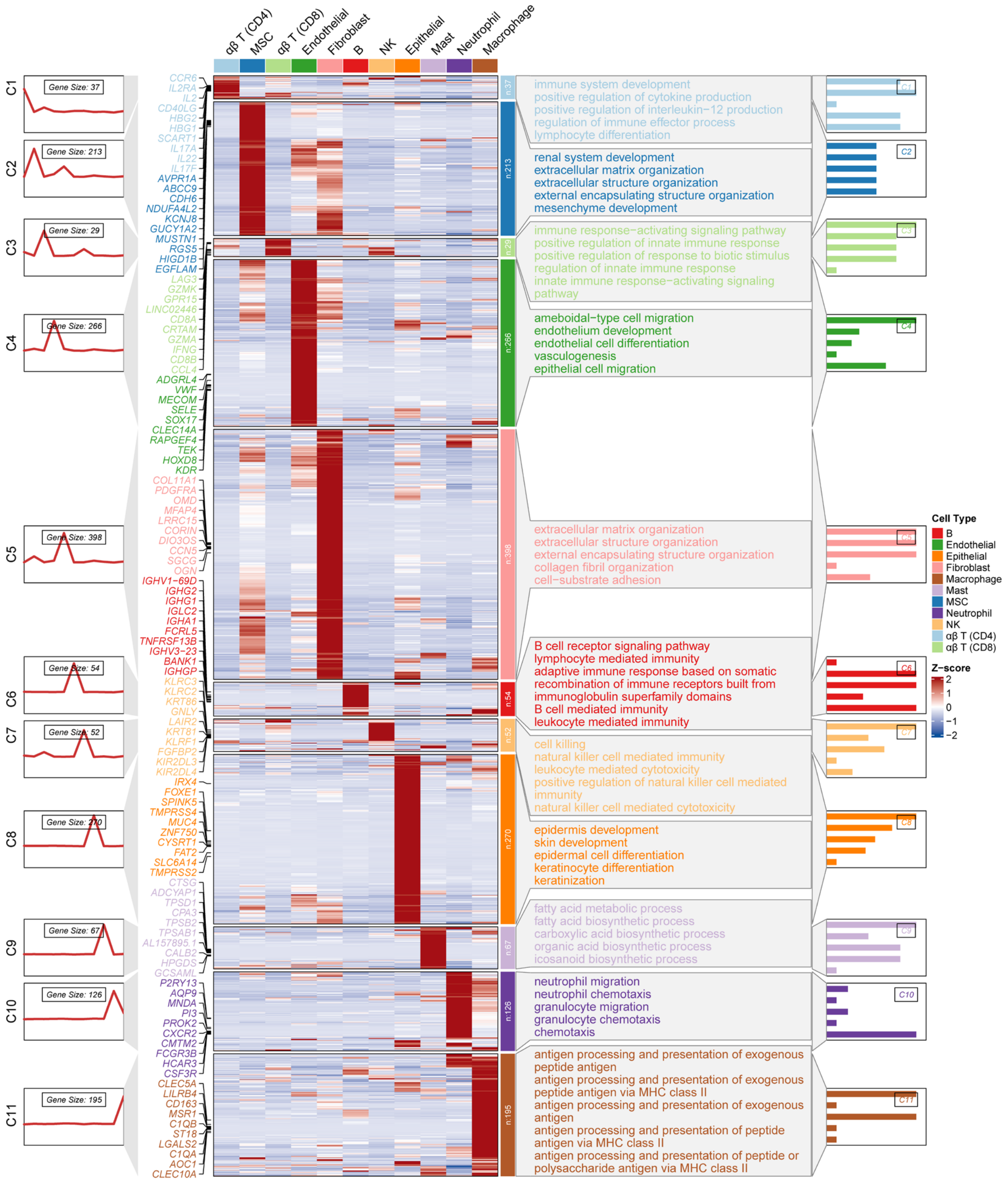


**Supplementary Figure 14. Heatmap of gene expression and GO enrichment of cell clusters of scRNA-seq.** Cells from GT, PT, and ST were further subdivided into 11 transcriptionally distinct clusters (C1–C11). The left panel presents a heatmap showing the top 10 signature genes per cluster (z score scaled). The right panel lists the top five GO biological processes significantly enriched in each cluster. n: gene size of each cell cluster. GT: osseous granulation tissue, PT: periodontal tissue; RT: root granulation tissue; ST: socket granulation tissue.

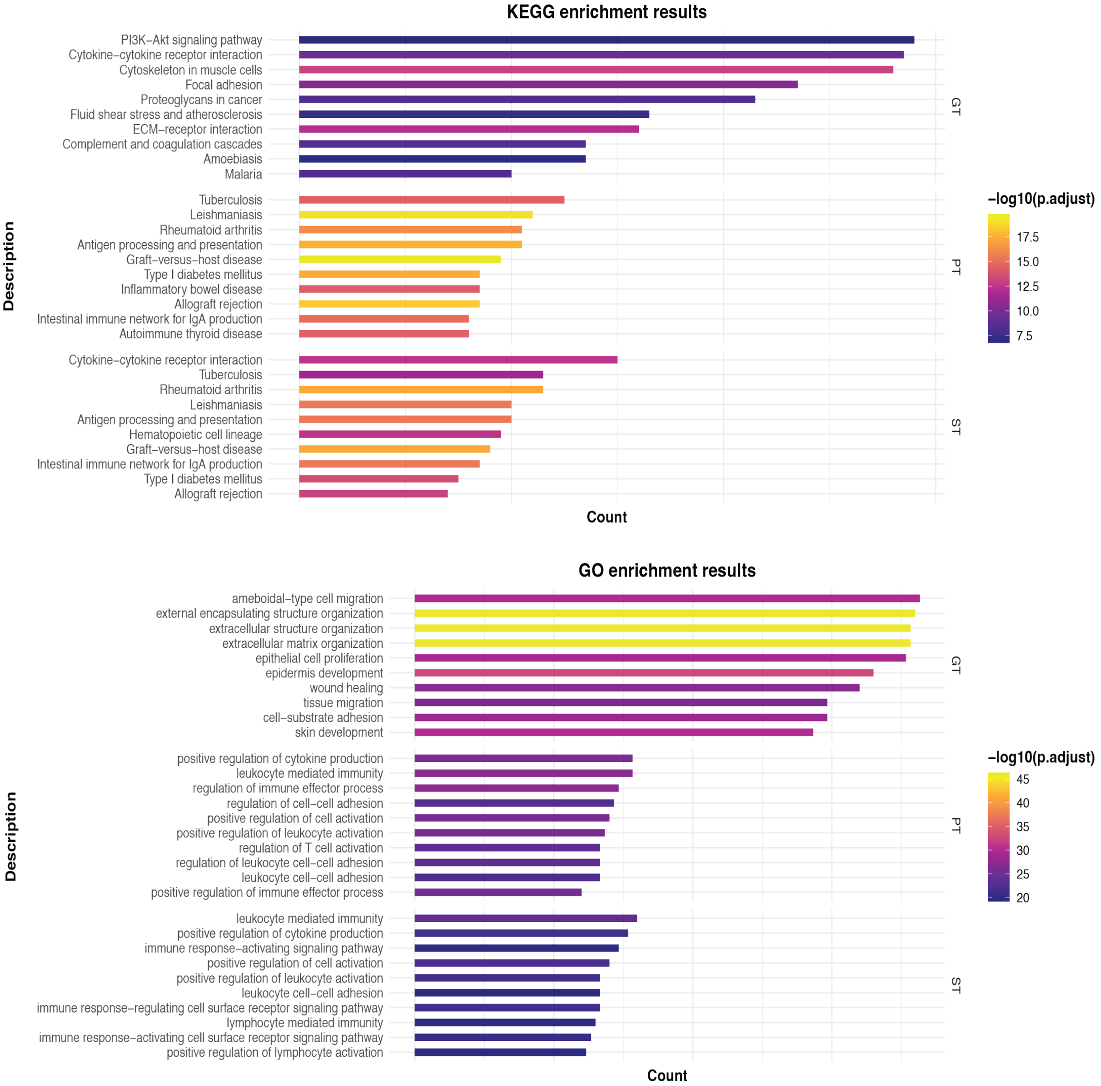


**Supplementary Figure 15. KEGG and GO enrichment of different groups of scRNA-seq.** The top 10 significantly enriched KEGG pathways and GO terms identified for each group in the single-cell sequencing dataset. GT: osseous granulation tissue, PT: periodontal tissue; RT: root granulation tissue; ST: socket granulation tissue.

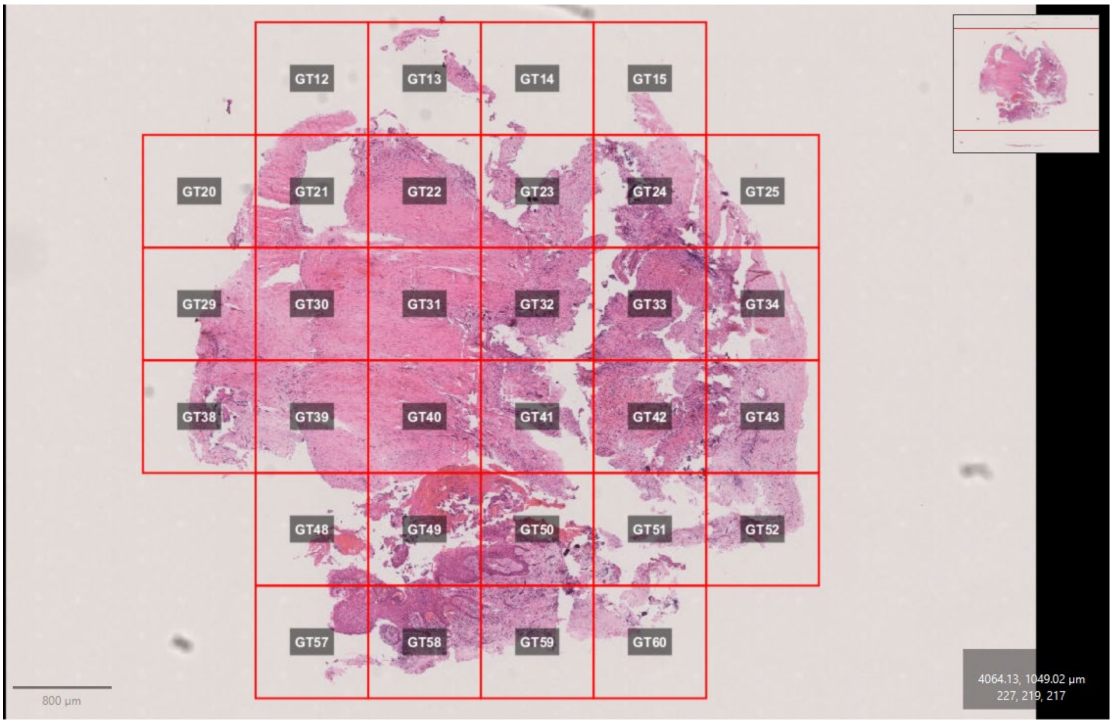


**Supplementary Figure 16. Representative segmentation of sequential square tiles using QuPath software.** The image shown is a representative slide of a GT. Each slide of specimen was automatically segmented into sequential square tiles using a custom script. Inflammatory infiltrate of tiles of each slide was semi-quantified with object classifier (artificial neural network, ANN_MLP). The percentage of infiltration was calculated by dividing the number of positively infiltrate-classified cells by the total number of detected cells in each tile and whole slide of specimen, respectively. The segmentation of both H&E and IHC used the identical script.

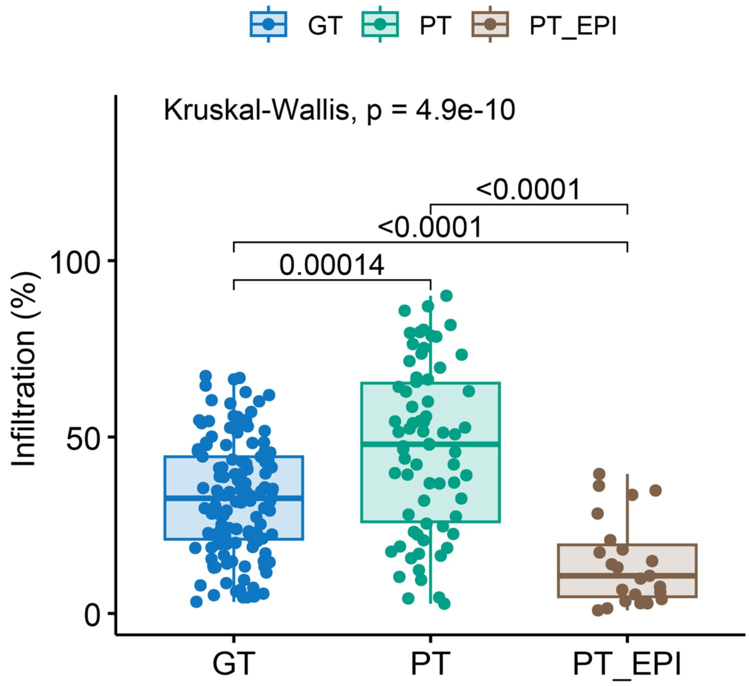


**Supplementary Figure 17. Histology staining of GT and PT.** PT showed distinct infiltration extent between epithelium (PT_EPI) and lamina propria. Manually drawn polygon tiles of PT_EPI were semi-quantified. The difference of Infiltration (%) was statistically assessed by Krusakl−Wallis test ($p$) with *post hoc* Dunn's test controlling false discovery rate using Benjamini−Hochberg method ($q$). Significant $q$ values ($q < 0.05$) are shown. GT: osseous granulation tissue; PT: periodontal tissue; PT_EPI: epithelium of PT.

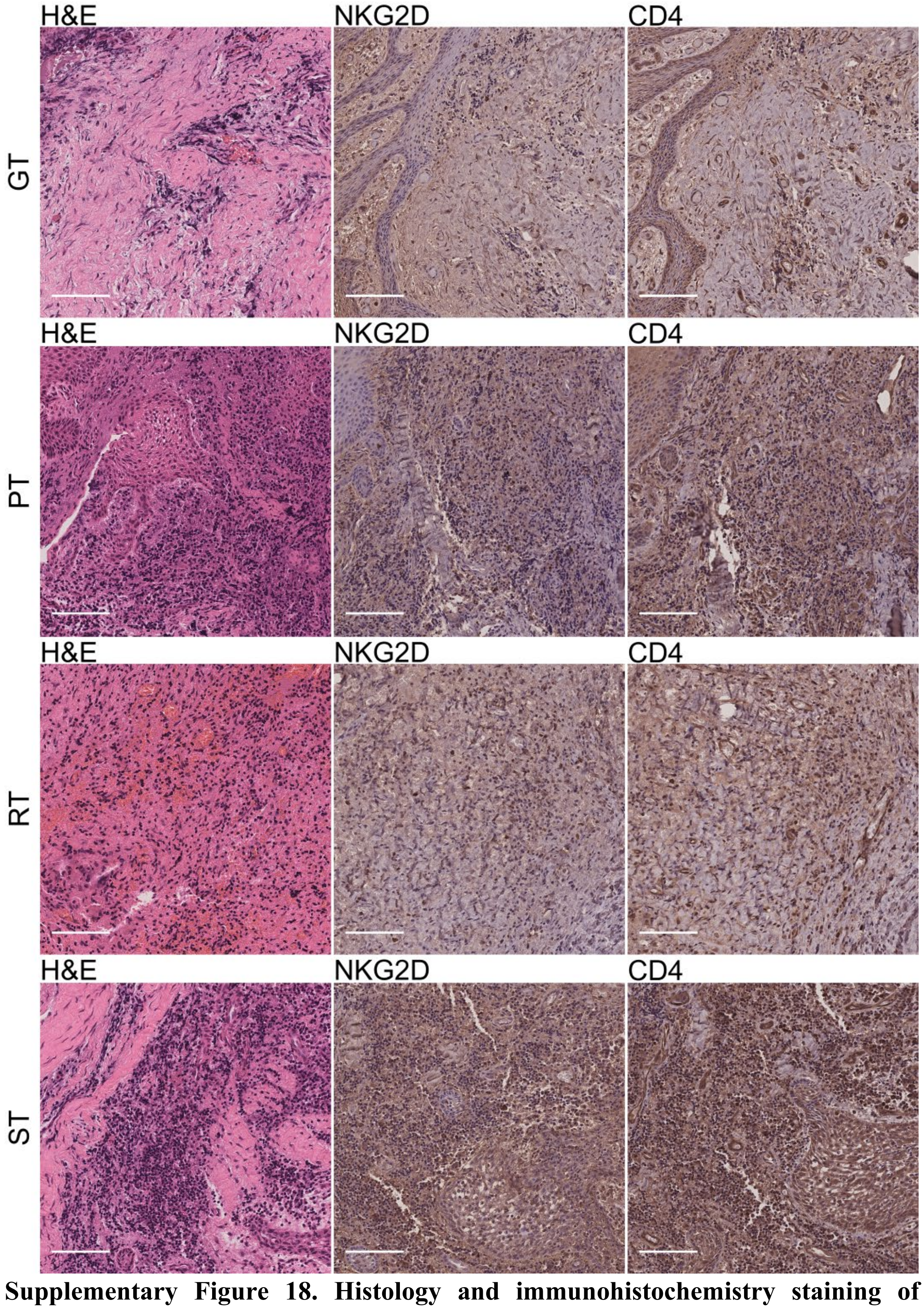


**Supplementary Figure 18. Histology and immunohistochemistry staining of different tissue types.** Representative images of H&E staining and IHC staining of NKG2D and CD4. Scale bar: 100 μm.

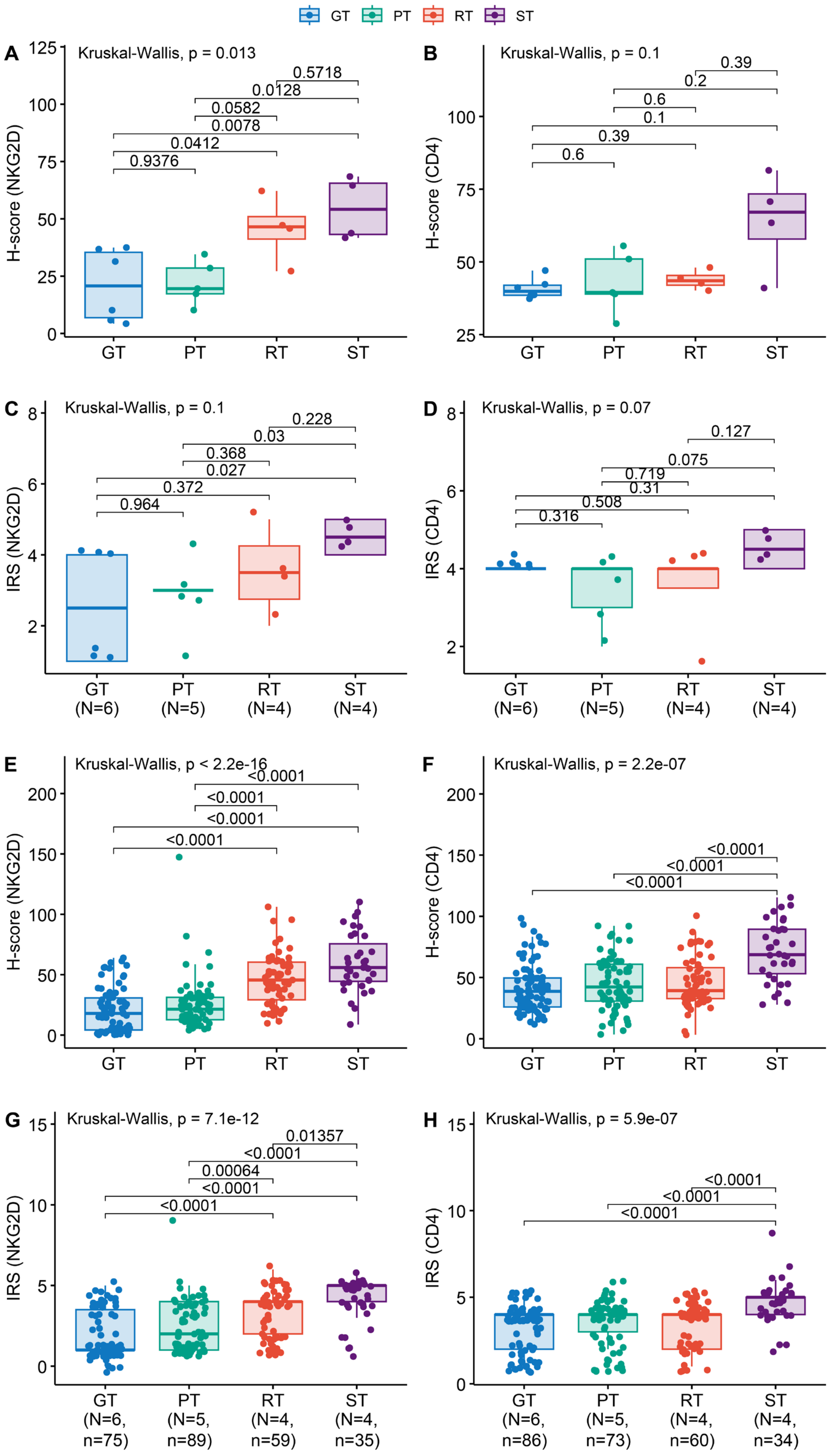

GT PT RT ST
A
Kruskal-Wallis, p = 0.013
H-score (NKG2D)
0.5718
0.0128
0.0582
0.0078
0.0412
0.9376
B
Kruskal-Wallis, p = 0.1
H-score (CD4)
0.39
0.2
0.6
0.1
0.39
0.6
C
Kruskal-Wallis, p = 0.1
IRS (NKG2D)
0.228
0.03
0.368
0.027
0.372
0.964
GT (N=6)
PT (N=5)
RT (N=4)
ST (N=4)
D
Kruskal-Wallis, p = 0.07
IRS (CD4)
0.127
0.075
0.719
0.31
0.508
0.316
E
Kruskal-Wallis, p < 2.2e-16
H-score (NKG2D)
<0.0001
F
Kruskal-Wallis, p = 2.2e-07
H-score (CD4)
<0.0001
G
Kruskal-Wallis, p = 7.1e-12
IRS (NKG2D)
0.01357
<0.0001
0.00064
GT (N=6, n=75)
PT (N=5, n=89)
RT (N=4, n=59)
ST (N=4, n=35)
H
Kruskal-Wallis, p = 5.9e-07
IRS (CD4)
GT (N=6, n=86)
PT (N=5, n=73)
RT (N=4, n=60)
ST (N=4, n=34)

**Supplementary Figure 19. Semi-quantification of immunohistochemistry of staining of different tissue types.** Each slide of specimen or biopsy was automatically segmented into sequential square tiles. For cell detection, positive cells were detected using "Positive cell detection" function with the setting of the default three-leveled threshold in "Intensity threshold parameter" at "Score compartment" of "Cell: DAB OD mean" for markers. The positiveness was further semi-quantified using both H-score and immunoreactivity score (IRS). (A−D) H-score and IRS of each specimen among tissue types with all *q* values shown. (E−F) H-score and IRS of each specimen among tissue types with significant *q* values ($q < 0.05$) shown. The difference of H-score and IRS among tissue types was statistically assessed by Krusakl−Wallis test (*p*) with *post hoc* Dunn's test controlling false discovery rate using Benjamini−Hochberg method (*q*). GT: osseous granulation tissue, PT: periodontal tissue; RT: root granulation tissue; ST: socket granulation tissue; N: number of tissue samples; n: number of square tiles.

# Supplementary Tables

## Table 1. Baseline periodontal parameters

| Variables | Total (n=49) | Stage III (n=31) | Stage IV (n=18) | *P* value |
|---|---|---|---|---|
| **Number of teeth** | 28 (25.0, 29.5) | 28 (26.0, 30.0) | 25.5 (23.0, 28.0) | **0.048** |
| **FMPS (%)** | 82 (66.0, 91.5) | 78 (60.0, 88.0) | 88.5 (74.0, 94.0) | **0.026** |
| **FMBS (%)** | 76.8±17.2 | 74.6±17.9 | 80.7±15.7 | 0.23 |
| **Mean PD (mm)** | 3.9 (3.2, 5.0) | 3.5(3.1, 4.2) | 4.5 (3.6, 5.7) | **0.005** |
| **PD ≥ 4mm (%)** | 35.0±5 | 38.2±22.8 | 45.0±25.7 | **0.013** |
| **PD ≥ 6mm (%)** | 19.4 (9.0, 32.7) | 16.0 (8.0, 23.5) | 28.3(15.2, 51.1) | **0.004** |
| **Mean CAL (mm)** | 5.0±1.5 | 4.35±1.0 | 6.2 ±1.5 | **<0.001** |
| **FI ≥ II (%)** | 10.7 (3.2, 19.7) | 7.1 (3.1, 15.1) | 13.4 (6.5, 19.4) | 0.077 |
| **Mobility ≥ II (%)** | 7.7 (0.0, 17.4) | 3.4 (0.0, 9.7) | 16.1 (9.4, 24.0) | **<0.001** |

FMPS: full mouth plaque score; FMBS: full mouth bleeding score; PD: probing depth; CAL: clinical attachment loss, FI ≥ II: horizontal furcation involvement grade II or above (Hamp's index), Mobility ≥ II (Miller's degree); mean PD: full-mouth mean of PD; mean CAL: full-mouth mean of CAL.

FMBS, mean CAL, FI ≥ II, MI ≥ II, PD ≥ 4mm and PD ≥ 6mm are presented in median and interquartile range (IQR) and assessed with Mann−Whitney U test. Others are presented in mean ± SD and assessed with independent two-side *t*-test**.**

## Table 2. Periodontal parameters after non-surgical periodontal treatment in 38 subjects

| Variables | Total (n=38) | Stage III (n=26) | Stage IV (n=12) | *P* value |
|---|---|---|---|---|
| **Number of teeth** | 25.0±3.8 | 26.44±3.0 | 21.92±3.6 | **<0.001** |
| **FMPS (%)** | 16.0±6.5 | 15.0±7.0 | 17.0±5.7 | 0.524 |
| **FMBS (%)** | 23.0 (16.0, 26.0) | 23.0 (14.0, 26.0) | 23.0 (17.0, 30.0) | 0.491 |
| **Mean PD (mm)** | 2.7±5.2 | 2.6±0.4 | 3.0±0.7 | **0.019** |
| **PD ≥ 4mm (%)** | 12.0 (7.0, 20.0) | 12.0 (5.0, 15.0) | 19.0 (12.0, 23.0) | **0.027** |
| **PD ≥ 6mm (%)** | 4.0 (1.0, 6.0) | 3.0 (1.0, 6.0) | 5.0 (3.0, 7.0) | 0.083 |
| **Mean CAL (mm)** | 3.83 (3.3, 4.6) | 3.6 (3.2, 4.1) | 4.7 (3.6, 7.3) | **0.008** |
| **FI ≥ II (%)** | 4.0 (0.0, 13.0) | 4.0 (0.0, 12.0) | 5.0 (1.0, 17.0) | 0.28 |
| **Mobility ≥ II (%)** | 0.0 (0.0, 0.0) | 0.0 (0.0, 0.0) | 0.0 (0.0, 0.0) | 0.67 |

**Table 3. Change of clinical parameters after non-surgical periodontal treatment in 38 subjects**

| Reduction | Total (n=38) | Stage III (n=26) | Stage IV (n=12) | *P* value |
|---|---|---|---|---|
| **FMPS (%)** | 61.2 (43.0, 72.0) | 54.0 (41.0, 68.0) | 68.0 (61.2, 72.5) | 0.8 |
| **FMBS (%)** | 56.5 (38.8, 65.6) | 57.0 (33.0, 65.0) | 56.0 (49.0, 66.0) | 0.79 |
| **PD ≥ 4mm (%)** | 27.6 (13.8, 44.8) | 25.7 (14.1, 36.4) | 36.7 (13.5, 53.8) | 0.42 |
| **PD ≥ 6mm (%)** | 15.0 (6.1, 31.4) | 11.7 (5.9, 20.7) | 29.0 (9.1, 47.4) | 0.08 |
| **M ≥ II (%)** | 6.9 (0.0, 11.9) | 0.0 (0.0, 9.4) | 0.0 (10.0, 19.1) | **0.001** |
| **FI ≥ II (%)** | 3.4 (0.0, 6.7) | 3.1 (0.0, 5.1) | 3.5 (0.0, 12.6) | 0.396 |
| **Mean PD (mm)** | 1.1 (0.7, 2.1) | 0.9 (0.7, 1.7) | 1.8 (0.9, 2.6) | 0.124 |
| **Mean CAL (mm)** | 0.7±0.7 | 0.70±0.6 | 0.7±0.9 | 0.948 |

## Table 4. Summary of collected samples

| Subj ID | Stage | GT | Tooth | Osseous defect | PT | Tooth | RT | Tooth | ST | Tooth | 16S | scRNA seq | Histology |
|---|---|---|---|---|---|---|---|---|---|---|---|---|---|
| P001 | III | | | | | | + | 16 | + | 16 | | | + ST |
| P002 | III | | | | | | | | | | | | |
| P003 | III | + | 44,45, 46 | Infrabony | + | 44,45, 46 | | | | | + GT, PT | | |
| P004 | IV | | | | | | + | 14*, 15 | + | 13,22, 23,25 | + RT, ST | | + ST, RT |
| P005 | IV | + | 25 | Infrabony | | | | | | | | | + GT |
| P006 | IV | | | | | | + | 36 | + | 36 | + RT, ST | | |
| P007 | III | + | 37,47 | Infrabony | | | + | 48 | | | + GT, RT | | + GT, RT |
| P008 | III | + | 16,27, 37 | Infrabony | + | 27 | | | | | + GT, PT | | + GT, PT |
| P009 | III | + | 36, 24, 25, 26 | 24: Infrabony 26,36: Combined | + | 24, 25, 26, 36 | | | | | + GT, PT | | + GT, PT |
| P010 | III | + | 12,13, 15,16, 17 | Infrabony | + | 13,15, 16,17 | | | | | + GT, PT | + PT | |
| P011 | III | + | 36 | | + | 36 | + | 37 | + | 37 | + PT, ST | + ST | + PT |
| P012 | IV | | | | | | + | 32, 31,41 | + | 41, 32,31 | + RT, ST | | + ST |
| P013 | III | + | 47 | Infrabony | + | 46, 47 | | | | | | | + PT |
| P014 | IV | + | 16 | Combined | + | 16 | | | | | + GT, PT | | + GT, PT |
| P015 | IV | + | 33 | Infrabony | + | 33 | | | | | + GT, PT | | |
| P016 | III | + | 33, 34 | Infrabony | | | | | | | | | |
| P017 | IV | + | 14 | Infrabony | | | | | | | + GT | | |
| P018 | III | + | 45 | Infrabony | | | | | | | | | |
| P019 | III | + | 36 | Combined | + | 36 | | | | | + GT, PT | | |
| P020 | IV | + | 46 | Combined | + | 35,36, 37; 46 | | | | | + PT | + GT | + GT |
| P021 | IV | | | | | | + | 27 | + | 17,27 | + ST | | |
| P022 | IV | | | | | | + | 36 | | | + RT | | |
| P023 | III | | | | | | + | 35 | + | 35 | | | |
| P024 | III | + | 37* | Infrabony | | | | | | | + GT | | |
| P025 | III | | | | | | + | 17 | + | 17 | + ST | | + ST |
| P026 | III | + | 37 | Combined | | | | | | | + GT | | + GT |
| P027 | III | + | 46 | Infrabony | | | | | | | + GT | | |
| P028 | IV | | | | | | | | + | 11, 24 | + ST | | |
| P029 | III | | | | | | + | 17 | | | | | |
| P030 | III | + | 16 | Combined | | | | | | | + GT | | + GT |

| Subj ID | Stage | GT | Tooth | Osseous defect | PT | Tooth | RT | Tooth | ST | Tooth | 16S | scRNA seq | Histology |
|---|---|---|---|---|---|---|---|---|---|---|---|---|---|
| **P031** | III | + | 34,36, 45 | Infrabony | | | | | | | + GT | | |
| **P032** | III | + | 24 | Infrabony | | | | | | | + GT | | |
| **P033** | III | + | 16 | Combined | | | | | | | + GT | | |
| **P034** | IV | | | | | + | 18 | | + | 17 | + RT, ST | | + ST |
| **P035** | III | + | 14 | Infrabony | | | | | | | + GT | | |
| **P036** | III | + | 46 | Combined | | | | | | | | | |
| **P037** | III | + | 25,26 | Combined | + | 25, 26 | | | | | + GT, PT | | |
| **P038** | IV | + | 44, 45, 46, 47 | 44, 45: Infrabony 46, 47: Combined | + | 45,46, 47 | | | | | + GT | | |
| **P039** | III | + | 14,16 | Combined | | | | | | | + GT | | |
| **P040** | IV | + | 14 | Infrabony | | | | | | | | | |
| **P041** | III | | | | | + | 16 | | + | 16, 27 | + RT, ST | | |
| **P042** | IV | + | 44 | Infrabony | | | | | | | + GT | | |
| **P043** | III | + | 27 | | + | 27 | | | | | + PT | | |
| **P044** | IV | | | | | | + | 31 | + | 31 | | | + RT, ST |
| **P045** | III | + | 15, 16 | Combined | | | | | | | + GT | | |
| **P046** | IV | + | 14 | Infrabony | | | | | | | + GT | | |
| **P047** | IV | + | 24,26, 27 | Combined | | | | | | | + GT | | |
| **P048** | III | + | 37 | Infrabony | | | | | | | | | |
| **P049** | III | + | 26 | | + | 26 | | | | | + PT | | |

GT: Osseous defect granulation tissues; PT: Inflamed gingival/supracrestal tissue; RT: Granulation tissues attached on root surface of extracted tooth; ST: Granulation tissue of tooth extraction socket; Infrabony: Granulation tissue from an infrabony defect; Combined: Granulation tissue from a combined infrabony and furcation osseous defect. *: Tissue samples that were divided into two due to their large size. +: Tissue samples collected and/or analyzed by 16S rRNA-seq, scRNA-seq and histology.

Totally, 55 GTs, 16 PTs, 18 RTs and 20 STs were collected. Some tissue samples were not subjected for further analyses due to small tissue size, but their local periodontal parameters were still analyzed.

**Table 5. Periodontal parameters of tooth sites for all collected tissue samples**

| Parameter | Total (n=112) | GT (n=30) | PT (n=20) | RT (n=7) | ST (n=12) | *P*-value |
|---|---|---|---|---|---|---|
| **Mean PD (mm)** | 4.4 (3.5, 5.5) | 3.8 (3.3, 4.5) | 3.6 (3.3, 4.4) | 6.7 (6.3, 7.5) | 6.6 (5.2, 7.8) | **<0.001** |
| | | | | | | GT–PT: 0.993 |
| | | | | | | GT–RT: <**0.0001** |
| | | | | | | GT–ST: <**0.0001** |
| | | | | | | PT–RT: <**0.0001** |
| | | | | | | PT–ST: <**0.0001** |
| | | | | | | RT–ST: 0.993 |
| **Largest PD (mm)** | 7.0 (6.0, 8.0) | 6.0 (6.0, 7.0) | 6.0 (6.0, 7.0) | 10.0 (8.0, 11.0) | 9.5 (7.0, 11.0) | **<0.001** |
| | | | | | | GT–PT: 0.918 |
| | | | | | | GT–RT: <**0.0001** |
| | | | | | | GT–ST: <**0.0001** |
| | | | | | | PT–RT: <**0.0001** |
| | | | | | | PT– ST: <**0.0001** |
| | | | | | | RT–ST: 0.918 |
| **Mean CAL (mm)** | 6.0 (5.0, 8.4) | 5.5 (4.5, 6.2) | 5.5 (4.9, 6.9) | 9.2 (7.3, 11.0) | 9.9 (7.8, 11.9) | **<0.001** |
| | | | | | | GT–PT: 0.600 |
| | | | | | | GT–RT: <**0.0001** |
| | | | | | | GT–ST: <**0.0001** |
| | | | | | | PT–RT: <**0.001** |
| | | | | | | PT–ST: <**0.001** |
| | | | | | | RT–ST: 0.949 |
| **BOP (%)** | 66.7 (33.3, 100.0) | 50.0 (33.3, 66.7) | 47.2 (33.3, 52.8) | 100.0 (100.0, 100.0) | 100.0 (91.7, 100.0) | **<0.001** |
| | | | | | | GT–PT: 0.966 |
| | | | | | | GT–RT: **<0.0001** |
| | | | | | | GT–ST: **<0.0001** |
| | | | | | | PT–RT: **<0.0001** |
| | | | | | | PT–ST: **<0.0001** |
| | | | | | | RT–ST: 0.889 |
| **PISA ($mm^2$)** | 71.8 (36.9, 151.3) | 45.8 (21.9, 104.0) | 69.1 (39.7, 94.9) | 165.0 (90.2, 303.9) | 134.6 (73.7, 278.0) | **<0.001** |
| | | | | | | GT–PT: 0.489 |
| | | | | | | GT–RT: <**0.0001** |

| |
|---|
| GT–ST: **<0.001** |
| PT–RT: **0.010** |
| PT–ST: **0.028** |
| RT–ST: 0.542 |

PISA: periodontal inflamed surface area. Mean PD and mean CAL were calculated using the average six-site PD/CAL of the sampled teeth. Largest PD is the maximum of six-site PD of the sampled teeth. BOP% was calculated by the ratio of BOP sites on six sites of the sampled teeth. Mean PD, Largest PD, Mean CAL, BOP% and PISA are presented with median (IQR). Difference was assessed using Kruskal-Wallis tests with post hoc Dunn's test and Benjamini-Hochberg procedure to adjust p values for multiple comparisons. For pairwise test, adjusted p values are shown. Third molars were excluded in analysis of PISA. n: Number of teeth involved in collected tissue sample.

**Table 6. PERMANOVA (Bray–Curtis dissimilarity) of bacteriome data**

Overall PERMANOVA (adonis2)

| | Df | SumOfSqs | R2 | F | Pr(>F) | |
|---|---|---|---|---|---|---|
| Sample | 3 | 1.527233 | 0.084248 | 1.686647 | 0.014 | * |
| Residual | 55 | 16.60055 | 0.915752 | | | |
| Total | 58 | 18.12779 | 1 | | | |

Signif. codes: 0 '***' 0.001 '**' 0.01 '*' 0.05 '.' 0.1 ' ' 1

PERMANOVA with 999 permutations

Pairwise PERMANOVA (adonis2)

| Df | SumOfSqs | R2 | F | Pr(>F) | |
|---|---|---|---|---|---|
| **ST_vs_RT** | | | | | |
| **Df** | **SumOfSqs** | **R2** | **F** | **Pr(>F)** | |
| **1** | 0.24667 | 0.047502 | 0.847806 | 0.567 | |
| **17** | 4.946176 | 0.952498 | | | |
| **18** | 5.192846 | 1 | | | |
| **ST_vs_GT** | | | | | |
| **Df** | **SumOfSqs** | **R2** | **F** | **Pr(>F)** | |
| **1** | 0.555768 | 0.048017 | 1.815819 | 0.038 | * |
| **36** | 11.01853 | 0.951983 | | | |
| **37** | 11.57429 | 1 | | | |
| **ST_vs_PT** | | | | | |
| **Df** | **SumOfSqs** | **R2** | **F** | **Pr(>F)** | |
| **1** | 0.50171 | 0.072192 | 1.711811 | 0.044 | * |
| **22** | 6.447912 | 0.927808 | | | |
| **23** | 6.949622 | 1 | | | |
| **RT_vs_GT** | | | | | |
| **Df** | **SumOfSqs** | **R2** | **F** | **Pr(>F)** | |
| **1** | 0.67441 | 0.062289 | 2.192094 | 0.017 | * |
| **33** | 10.15264 | 0.937711 | | | |
| **34** | 10.82705 | 1 | | | |
| **RT_vs_PT** | | | | | |
| **Df** | **SumOfSqs** | **R2** | **F** | **Pr(>F)** | |
| **1** | 0.754284 | 0.119042 | 2.567419 | 0.002 | ** |
| **19** | 5.582027 | 0.880958 | | | |
| **20** | 6.336312 | 1 | | | |
| **GT_vs_PT** | | | | | |
| **Df** | **SumOfSqs** | **R2** | **F** | **Pr(>F)** | |
| **1** | 0.320387 | 0.026755 | 1.044647 | 0.394 | |
| **38** | 11.65438 | 0.973245 | | | |
| **39** | 11.97476 | 1 | | | |

**Table 7. Maaslin2 analysis of taxa associated with tissue types with or without covariate adjustment**

Taxa with more than 7 non-zero count samples at each level are shown. Taxa with $q < 0.05$ are colored in green. For reasonable interpretations, only taxa with ≥ 30 non-zero count samples can be meaningful.

7.1 Family

| Family | value | coef | stderr | pval | qval | N.not.zero | Sig. | upper_ci | lower_ci | Phylum | Class | Order |
|---|---|---|---|---|---|---|---|---|---|---|---|---|
| Micrococcaceae | RT | -2.28 | 0.49 | <0.001 | 0.011 | 59 | * | -1.32 | -3.24 | Actinobacteriota | Actinobacteria | Micrococcales |
| Neisseriaceae | RT | -2.81 | 0.67 | <0.001 | 0.027 | 59 | * | -1.50 | -4.13 | Proteobacteria | Gammaproteobacteria | Burkholderiales |
| Porphyromonadaceae | RT | 2.83 | 0.73 | <0.001 | 0.058 | 48 | | 4.27 | 1.39 | Bacteroidota | Bacteroidia | Bacteroidales |
| Enterococcaceae | PT | 2.58 | 0.71 | 0.001 | 0.090 | 42 | | 3.98 | 1.18 | Firmicutes | Bacilli | Lactobacillales |
| Micrococcaceae | ST | -1.52 | 0.43 | 0.001 | 0.098 | 59 | | -0.67 | -2.37 | Actinobacteriota | Actinobacteria | Micrococcales |
| Leptotrichiaceae | ST | 2.19 | 0.64 | 0.001 | 0.116 | 50 | | 3.45 | 0.93 | Fusobacteriota | Fusobacteriia | Fusobacteriales |
| Campylobacteraceae | ST | -2.80 | 0.88 | 0.002 | 0.191 | 44 | | -1.08 | -4.52 | Campylobacterota | Campylobacteria | Campylobacterales |

7.2 Adjusted Family

| Family | value | coef | stderr | pval | qval | N.not.zero | sig. | upper_ci | lower_ci | Phylum | Class | Order |
|---|---|---|---|---|---|---|---|---|---|---|---|---|
| Micrococcaceae | RT | -2.31 | 0.47 | <0.001 | 0.001 | 59 | ** | -1.39 | -3.23 | Actinobacteriota | Actinobacteria | Micrococcales |
| Neisseriaceae | RT | -2.46 | 0.56 | <0.001 | 0.003 | 59 | ** | -1.37 | -3.55 | Proteobacteria | Gammaproteobacteria | Burkholderiales |
| Porphyromonadaceae | RT | 2.28 | 0.56 | <0.001 | 0.007 | 48 | ** | 3.37 | 1.18 | Bacteroidota | Bacteroidia | Bacteroidales |
| Gemellaceae | PT | 2.18 | 0.55 | <0.001 | 0.008 | 59 | ** | 3.26 | 1.11 | Firmicutes | Bacilli | Staphylococcales |
| Leptotrichiaceae | ST | 2.52 | 0.64 | <0.001 | 0.009 | 50 | ** | 3.77 | 1.26 | Fusobacteriota | Fusobacteriia | Fusobacteriales |
| Campylobacteraceae | ST | -3.26 | 0.84 | <0.001 | 0.009 | 44 | ** | -1.61 | -4.90 | Campylobacterota | Campylobacteria | Campylobacterales |
| Leptotrichiaceae | RT | 2.39 | 0.67 | 0.001 | 0.017 | 50 | * | 3.70 | 1.08 | Fusobacteriota | Fusobacteriia | Fusobacteriales |
| Fusobacteriaceae | PT | -0.91 | 0.28 | 0.002 | 0.034 | 59 | * | -0.35 | -1.46 | Fusobacteriota | Fusobacteriia | Fusobacteriales |
| Family XI | RT | 1.22 | 0.40 | 0.004 | 0.046 | 59 | * | 2.01 | 0.43 | Firmicutes | Clostridia | Peptostreptococcales-Tissierellales |
| Tannerellaceae | ST | -1.88 | 0.64 | 0.005 | 0.054 | 57 | | -0.62 | -3.13 | Bacteroidota | Bacteroidia | Bacteroidales |
| Enterococcaceae | PT | 1.39 | 0.48 | 0.005 | 0.056 | 42 | | 2.33 | 0.45 | Firmicutes | Bacilli | Lactobacillales |
| Saccharimonadaceae | PT | -2.03 | 0.71 | 0.006 | 0.059 | 48 | | -0.64 | -3.41 | Patescibacteria | Saccharimonadia | Saccharimonadales |
| Flavobacteriaceae | RT | -1.89 | 0.70 | 0.009 | 0.077 | 58 | | -0.52 | -3.25 | Bacteroidota | Bacteroidia | Flavobacteriales |
| Micrococcaceae | ST | -1.19 | 0.44 | 0.009 | 0.077 | 59 | | -0.33 | -2.05 | Actinobacteriota | Actinobacteria | Micrococcales |
| Pasteurellaceae | ST | -1.73 | 0.65 | 0.011 | 0.085 | 55 | | -0.45 | -3.01 | Proteobacteria | Gammaproteobacteria | Enterobacterales |
| Eggerthellaceae | RT | -2.06 | 0.79 | 0.012 | 0.093 | 39 | | -0.50 | -3.61 | Actinobacteriota | Coriobacteriia | Coriobacteriales |

| | | | | | | | | | | | | |
|---|---|---|---|---|---|---|---|---|---|---|---|---|
| Xanthomonadaceae | PT | 1.84 | 0.77 | 0.021 | 0.128 | 30 | | 3.35 | 0.33 | Proteobacteria | Gammaproteobacteria | Xanthomonadales |
| Peptococcaceae | ST | 1.62 | 0.72 | 0.028 | 0.157 | 57 | | 3.02 | 0.21 | Firmicutes | Clostridia | Peptococcales |
| Campylobacteraceae | PT | -1.38 | 0.62 | 0.032 | 0.171 | 44 | | -0.15 | -2.60 | Campylobacterota | Campylobacteria | Campylobacterales |
| Family XI | ST | 0.82 | 0.38 | 0.035 | 0.178 | 59 | | 1.56 | 0.08 | Firmicutes | Clostridia | Peptostreptococcales-Tissierellales |
| Gemellaceae | ST | 1.32 | 0.61 | 0.036 | 0.182 | 59 | | 2.52 | 0.12 | Firmicutes | Bacilli | Staphylococcales |

7.3 Genus

| Genus | value | coef | stderr | pval | qval | N. not. zero | sig. | upper ci | lower ci | Phylum | Class | Order | Family |
|---|---|---|---|---|---|---|---|---|---|---|---|---|---|
| Rothia | RT | -2.57 | 0.50 | <0.001 | 0.004 | 59 | ** | -1.59 | -3.55 | Actinobacteriota | Actinobacteria | Micrococcales | Micrococcaceae |
| Neisseria | RT | -2.80 | 0.67 | <0.001 | 0.033 | 59 | * | -1.48 | -4.13 | Proteobacteria | Gammaproteobacteria | Burkholderiales | Neisseriaceae |
| Porphyromonas | RT | 2.83 | 0.73 | <0.001 | 0.062 | 48 | | 4.25 | 1.40 | Bacteroidota | Bacteroidia | Bacteroidales | Porphyromonadaceae |
| Lautropia | RT | -2.70 | 0.79 | 0.001 | 0.146 | 54 | | -1.15 | -4.26 | Proteobacteria | Gammaproteobacteria | Burkholderiales | Burkholderiaceae |
| Rothia | ST | -1.54 | 0.44 | 0.001 | 0.146 | 59 | | -0.67 | -2.40 | Actinobacteriota | Actinobacteria | Micrococcales | Micrococcaceae |
| Leptotrichia | ST | 2.20 | 0.65 | 0.001 | 0.146 | 49 | | 3.47 | 0.93 | Fusobacteriota | Fusobacteriia | Fusobacteriales | Leptotrichiaceae |
| Enterococcus | PT | 2.49 | 0.72 | 0.001 | 0.146 | 42 | | 3.90 | 1.09 | Firmicutes | Bacilli | Lactobacillales | Enterococcaceae |
| Lachnoanaerobaculum | RT | 2.01 | 0.60 | 0.002 | 0.151 | 38 | | 3.19 | 0.83 | Firmicutes | Clostridia | Lachnospirales | Lachnospiraceae |
| Campylobacter | ST | -2.71 | 0.88 | 0.003 | 0.237 | 44 | | -0.99 | -4.43 | Campylobacterota | Campylobacteria | Campylobacterales | Campylobacteraceae |
| W5053 | ST | 1.62 | 0.53 | 0.003 | 0.237 | 59 | | 2.64 | 0.59 | Firmicutes | Clostridia | Peptostreptococcales-Tissierellales | Family XI |

7.4 Adjusted Genus

| Genus | value | coef | stderr | pval | qval | N. not. zero | sig. | upper ci | lower ci | Phylum | Class | Order | Family |
|---|---|---|---|---|---|---|---|---|---|---|---|---|---|
| Rothia | RT | -2.56 | 0.49 | <0.001 | <0.001 | 59 | *** | -1.60 | -3.52 | Actinobacteriota | Actinobacteria | Micrococcales | Micrococcaceae |
| Neisseria | RT | -2.52 | 0.56 | <0.001 | 0.002 | 59 | ** | -1.42 | -3.61 | Proteobacteria | Gammaproteobacteria | Burkholderiales | Neisseriaceae |
| Porphyromonas | RT | 2.26 | 0.56 | <0.001 | 0.008 | 48 | ** | 3.36 | 1.16 | Bacteroidota | Bacteroidia | Bacteroidales | Porphyromonadaceae |
| Lautropia | RT | -2.25 | 0.57 | <0.001 | 0.009 | 54 | ** | -1.13 | -3.37 | Proteobacteria | Gammaproteobacteria | Burkholderiales | Burkholderiaceae |
| Gemella | PT | 2.17 | 0.55 | <0.001 | 0.010 | 59 | ** | 3.25 | 1.09 | Firmicutes | Bacilli | Staphylococcales | Gemellaceae |
| Leptotrichia | ST | 2.55 | 0.65 | <0.001 | 0.010 | 49 | ** | 3.82 | 1.28 | Fusobacteriota | Fusobacteriia | Fusobacteriales | Leptotrichiaceae |
| Campylobacter | ST | -3.19 | 0.84 | <0.001 | 0.012 | 44 | * | -1.55 | -4.83 | Campylobacterota | Campylobacteria | Campylobacterales | Campylobacteraceae |

| | | | | | | | | | | | | | |
|---|---|---|---|---|---|---|---|---|---|---|---|---|---|
| W5053 | ST | 1.66 | 0.46 | 0.001 | 0.019 | 59 | * | 2.57 | 0.76 | Firmicutes | Clostridia | Peptostreptococcales-Tissierellales | Family XI |
| Leptotrichia | RT | 2.39 | 0.67 | 0.001 | 0.021 | 49 | * | 3.71 | 1.07 | Fusobacteriota | Fusobacteriia | Fusobacteriales | Leptotrichiaceae |
| TM7x | PT | -2.53 | 0.78 | 0.002 | 0.040 | 30 | * | -1.00 | -4.07 | Patescibacteria | Saccharimonadia | Saccharimonadales | Saccharimonadaceae |
| Fusobacterium | PT | -0.90 | 0.28 | 0.003 | 0.044 | 59 | * | -0.35 | -1.45 | Fusobacteriota | Fusobacteriia | Fusobacteriales | Fusobacteriaceae |
| Lachno-anaerobaculum | RT | 1.87 | 0.61 | 0.003 | 0.052 | 38 | | 3.05 | 0.68 | Firmicutes | Clostridia | Lachnospirales | Lachnospiraceae |
| Tannerella | ST | -2.24 | 0.75 | 0.004 | 0.058 | 54 | | -0.78 | -3.70 | Bacteroidota | Bacteroidia | Bacteroidales | Tannerellaceae |
| Rothia | ST | -1.28 | 0.46 | 0.008 | 0.087 | 59 | | -0.38 | -2.18 | Actinobacteriota | Actinobacteria | Micrococcales | Micrococcaceae |
| Enterococcus | PT | 1.34 | 0.49 | 0.009 | 0.093 | 42 | | 2.29 | 0.38 | Firmicutes | Bacilli | Lactobacillales | Enterococcaceae |
| Erysipelato-clostridium | PT | 1.95 | 0.72 | 0.009 | 0.099 | 39 | | 3.37 | 0.54 | Firmicutes | Bacilli | Erysipelotrichales | Erysipelatoclostridiaceae |
| Capnocytophaga | RT | -1.99 | 0.75 | 0.010 | 0.104 | 57 | | -0.53 | -3.45 | Bacteroidota | Bacteroidia | Flavobacteriales | Flavobacteriaceae |
| Lachnoclostridium | PT | 1.29 | 0.50 | 0.013 | 0.120 | 31 | | 2.27 | 0.31 | Firmicutes | Clostridia | Lachnospirales | Lachnospiraceae |
| Faecalibacterium | PT | 1.52 | 0.59 | 0.013 | 0.120 | 33 | | 2.68 | 0.37 | Firmicutes | Clostridia | Oscillospirales | Ruminococcaceae |
| Parvimonas | RT | 1.20 | 0.47 | 0.013 | 0.120 | 56 | | 2.11 | 0.29 | Firmicutes | Clostridia | Peptostreptococcales-Tissierellales | Family XI |
| Prevotella | PT | -1.74 | 0.68 | 0.014 | 0.130 | 44 | | -0.40 | -3.08 | Bacteroidota | Bacteroidia | Bacteroidales | Prevotellaceae |
| Faecalibacterium | ST | 1.64 | 0.65 | 0.015 | 0.135 | 33 | | 2.93 | 0.36 | Firmicutes | Clostridia | Oscillospirales | Ruminococcaceae |
| Haemophilus | ST | -1.77 | 0.73 | 0.019 | 0.154 | 49 | | -0.34 | -3.20 | Proteobacteria | Gammaproteobacteria | Enterobacterales | Pasteurellaceae |
| Aggregatibacter | PT | -1.53 | 0.64 | 0.020 | 0.159 | 39 | | -0.28 | -2.78 | Proteobacteria | Gammaproteobacteria | Enterobacterales | Pasteurellaceae |
| Peptococcus | ST | 1.72 | 0.74 | 0.024 | 0.175 | 57 | | 3.16 | 0.27 | Firmicutes | Clostridia | Peptococcales | Peptococcaceae |
| Filifactor | ST | 1.20 | 0.54 | 0.031 | 0.201 | 59 | | 2.26 | 0.14 | Firmicutes | Clostridia | Peptostreptococcales-Tissierellales | Peptostreptococcaceae |
| Lautropia | PT | -0.74 | 0.33 | 0.031 | 0.203 | 54 | | -0.09 | -1.39 | Proteobacteria | Gammaproteobacteria | Burkholderiales | Burkholderiaceae |
| Campylobacter | PT | -1.37 | 0.63 | 0.034 | 0.215 | 44 | | -0.14 | -2.60 | Campylobacterota | Campylobacteria | Campylobacterales | Campylobacteraceae |
| Peptostreptococcus | RT | 1.02 | 0.47 | 0.035 | 0.220 | 53 | | 1.94 | 0.10 | Firmicutes | Clostridia | Peptostreptococcales-Tissierellales | Peptostreptococcaceae |
| W5053 | RT | 1.07 | 0.49 | 0.035 | 0.220 | 59 | | 2.04 | 0.10 | Firmicutes | Clostridia | Peptostreptococcales-Tissierellales | Family XI |
| Pseudo-propionibacterium | RT | -2.16 | 1.00 | 0.035 | 0.221 | 40 | | -0.20 | -4.11 | Actinobacteriota | Actinobacteria | Propionibacteriales | Propionibacteriaceae |
| Bulleidia | PT | -1.80 | 0.83 | 0.036 | 0.222 | 34 | | -0.17 | -3.44 | Firmicutes | Bacilli | Erysipelotrichales | Erysipelotrichaceae |
| F0332 | PT | 0.91 | 0.43 | 0.038 | 0.231 | 45 | | 1.75 | 0.07 | Actinobacteriota | Actinobacteria | Actinomycetales | Actinomycetaceae |
| Gemella | ST | 1.31 | 0.62 | 0.038 | 0.231 | 59 | | 2.52 | 0.10 | Firmicutes | Bacilli | Staphylococcales | Gemellaceae |

7.5 Species

| Species | value | coef | stderr | pval | qval | N. not. zero | sig. | upper ci | lower ci | Phylum | Class | Order | Family |
|---|---|---|---|---|---|---|---|---|---|---|---|---|---|
| Rothia_aeria | RT | -3.19 | 0.68 | <0.001 | 0.004 | 59 | ** | -1.85 | -4.53 | Actinobacteriota | Actinobacteria | Micrococcales | Micrococcaceae |
| Neisseria_mucosa | RT | -3.08 | 0.69 | <0.001 | 0.005 | 59 | ** | -1.73 | -4.42 | Proteobacteria | Gammaproteobacteria | Burkholderiales | Neisseriaceae |
| Porphyromonas_gingivalis | RT | 2.99 | 0.76 | <0.001 | 0.015 | 44 | * | 4.47 | 1.51 | Bacteroidota | Bacteroidia | Bacteroidales | Porphyromonadaceae |
| Actinomyces_oris | ST | 2.11 | 0.61 | 0.001 | 0.032 | 30 | * | 3.31 | 0.92 | Actinobacteriota | Actinobacteria | Actinomycetales | Actinomycetaceae |
| Rothia_aeria | ST | -1.78 | 0.60 | 0.005 | 0.116 | 59 | | -0.59 | -2.96 | Actinobacteriota | Actinobacteria | Micrococcales | Micrococcaceae |
| Streptococcus_constellatus | ST | 0.96 | 0.33 | 0.005 | 0.116 | 58 | | 1.59 | 0.32 | Firmicutes | Bacilli | Lactobacillales | Streptococcaceae |
| Capnocytophaga_sputigena | RT | -1.74 | 0.65 | 0.010 | 0.196 | 57 | | -0.46 | -3.02 | Bacteroidota | Bacteroidia | Flavobacteriales | Flavobacteriaceae |
| Haemophilus_parainfluenzae | RT | -2.60 | 1.01 | 0.013 | 0.214 | 49 | | -0.62 | -4.57 | Proteobacteria | Gammaproteobacteria | Enterobacterales | Pasteurellaceae |
| Porphyromonas_gingivalis | ST | 1.92 | 0.74 | 0.013 | 0.214 | 44 | | 3.37 | 0.46 | Bacteroidota | Bacteroidia | Bacteroidales | Porphyromonadaceae |
| Rothia_mucilaginosa | PT | 1.48 | 0.58 | 0.014 | 0.225 | 41 | | 2.63 | 0.34 | Actinobacteriota | Actinobacteria | Micrococcales | Micrococcaceae |
| Capnocytophaga_gingivalis | RT | -3.29 | 1.32 | 0.016 | 0.236 | 33 | | -0.70 | -5.87 | Bacteroidota | Bacteroidia | Flavobacteriales | Flavobacteriaceae |
| Streptococcus_sanguinis | RT | -1.07 | 0.43 | 0.016 | 0.236 | 59 | | -0.23 | -1.91 | Firmicutes | Bacilli | Lactobacillales | Streptococcaceae |

7.6 Adjusted Species

| Species | value | coef | stderr | pval | qval | N. not. zero | sig. | upper ci | lower ci | Phylum | Class | Order | Family |
|---|---|---|---|---|---|---|---|---|---|---|---|---|---|
| Rothia_aeria | RT | -3.60 | 0.66 | <0.001 | <0.001 | 59 | *** | -2.30 | -4.89 | Actinobacteriota | Actinobacteria | Micrococcales | Micrococcaceae |
| Neisseria_mucosa | RT | -2.61 | 0.56 | <0.001 | 0.002 | 59 | ** | -1.51 | -3.71 | Proteobacteria | Gamma-proteobacteria | Burkholderiales | Neisseriaceae |
| Porphyromonas_gingivalis | RT | 2.41 | 0.64 | <0.001 | 0.012 | 44 | * | 3.67 | 1.15 | Bacteroidota | Bacteroidia | Bacteroidales | Porphyromonadaceae |
| Fusobacterium_nucleatum | PT | -0.86 | 0.30 | 0.007 | 0.073 | 59 | | -0.27 | -1.46 | Fusobacteriota | Fusobacteriia | Fusobacteriales | Fusobacteriaceae |
| Haemophilus_parainfluenzae | ST | -1.96 | 0.73 | 0.010 | 0.097 | 49 | | -0.54 | -3.39 | Proteobacteria | Gamma-proteobacteria | Enterobacterales | Pasteurellaceae |
| Rothia_mucilaginosa | PT | 1.43 | 0.54 | 0.011 | 0.108 | 41 | | 2.50 | 0.36 | Actinobacteriota | Actinobacteria | Micrococcales | Micrococcaceae |
| [Eubacterium] saphenum_saphenum | ST | 1.91 | 0.77 | 0.017 | 0.141 | 34 | | 3.43 | 0.39 | Firmicutes | Clostridia | Peptostreptococcales-Tissierellales | Anaerovoracaceae |
| Parvimonas_micra | RT | 1.19 | 0.48 | 0.018 | 0.144 | 52 | | 2.14 | 0.24 | Firmicutes | Clostridia | Peptostreptococcales-Tissierellales | Family XI |
| Capnocytophaga_sputigena | RT | -1.38 | 0.57 | 0.019 | 0.151 | 57 | | -0.26 | -2.50 | Bacteroidota | Bacteroidia | Flavobacteriales | Flavobacteriaceae |
| [Eubacterium] brachy_brachy | ST | 1.18 | 0.50 | 0.022 | 0.162 | 59 | | 2.15 | 0.20 | Firmicutes | Clostridia | Peptostreptococcales-Tissierellales | Anaerovoracaceae |
| Streptococcus_constellatus | RT | 0.92 | 0.39 | 0.023 | 0.166 | 58 | | 1.68 | 0.15 | Firmicutes | Bacilli | Lactobacillal | Streptococcaceae |

| | | | | | | | | | | | | | |
|---|---|---|---|---|---|---|---|---|---|---|---|---|---|
| Actinomyces_oris | ST | 1.32 | 0.58 | 0.027 | 0.185 | 30 | | 2.45 | 0.18 | Actinobacteriota | Actinobacteria | Actinomycetales | Actinomycetaceae |
| Filifactor_alocis | ST | 1.25 | 0.56 | 0.030 | 0.196 | 59 | | 2.35 | 0.15 | Firmicutes | Clostridia | Peptostreptococcales-Tissierellales | Peptostreptococcaceae |
| Streptococcus_constellatus | ST | 0.82 | 0.37 | 0.030 | 0.198 | 58 | | 1.55 | 0.10 | Firmicutes | Bacilli | Lactobacillales | Streptococcaceae |
| Bulleidia_extructa | PT | -1.86 | 0.84 | 0.031 | 0.199 | 34 | | -0.22 | -3.50 | Firmicutes | Bacilli | Erysipelotrichales | Erysipelotrichaceae |
| Rothia_aeria | ST | -1.36 | 0.61 | 0.031 | 0.200 | 59 | | -0.16 | -2.57 | Actinobacteriota | Actinobacteria | Micrococcales | Micrococcaceae |
| Actinomyces_gerencseriae | RT | -2.22 | 1.01 | 0.033 | 0.204 | 33 | | -0.24 | -4.19 | Actinobacteriota | Actinobacteria | Actinomycetales | Actinomycetaceae |
| Capnocytophaga_gingivalis | RT | -2.52 | 1.15 | 0.033 | 0.207 | 33 | | -0.27 | -4.77 | Bacteroidota | Bacteroidia | Flavobacteriales | Flavobacteriaceae |
| Actinomyces_gerencseriae | ST | -1.97 | 0.91 | 0.035 | 0.213 | 33 | | -0.19 | -3.75 | Actinobacteriota | Actinobacteria | Actinomycetales | Actinomycetaceae |
| Streptococcus_sanguinis | RT | -1.02 | 0.49 | 0.042 | 0.234 | 59 | | -0.06 | -1.97 | Firmicutes | Bacilli | Lactobacillales | Streptococcaceae |

Sig.: significant; * $q < 0.05$, ** $q < 0.01$, *** $q < 0.001$.

**Table 8. Maaslin2 analysis of taxa associated with age, sex, overweight/obesity, DM and smoking status**

Taxa with more than mean number of non-zero count samples at each level were shown. Taxa with $q < 0.05$ are colored in green. For reasonable interpretations, only taxa with more than 30 non-zero count samples can be meaningful.

8.1 Family

| Family | meta | value | coef | stderr | pval | qval | N. not. zero | sig. | upper ci | lower ci | Phylum | Class | Order |
|---|---|---|---|---|---|---|---|---|---|---|---|---|---|
| Eubacteriaceae | Age | Age | -0.62 | 0.18 | 0.002 | 0.026 | 58 | * | -0.26 | -0.98 | Firmicutes | Clostridia | Eubacteriales |
| Rikenellaceae | Age | Age | 0.86 | 0.27 | 0.003 | 0.038 | 44 | * | 1.40 | 0.33 | Bacteroidota | Bacteroidia | Bacteroidales |
| Enterococcaceae | Age | Age | 0.65 | 0.21 | 0.004 | 0.045 | 42 | * | 1.07 | 0.23 | Firmicutes | Bacilli | Lactobacillales |
| Pasteurellaceae | DM | Yes | -3.62 | 0.93 | <0.001 | 0.009 | 55 | ** | -1.80 | -5.44 | Proteobacteria | Gammaproteobacteria | Enterobacterales |
| Muribaculaceae | DM | Yes | -5.44 | 1.43 | <0.001 | 0.011 | 44 | * | -2.65 | -8.24 | Bacteroidota | Bacteroidia | Bacteroidales |
| Rikenellaceae | DM | Yes | -5.08 | 1.45 | 0.001 | 0.019 | 44 | * | -2.24 | -7.93 | Bacteroidota | Bacteroidia | Bacteroidales |
| Oscillospiraceae | DM | Yes | -4.21 | 1.27 | 0.002 | 0.028 | 43 | * | -1.72 | -6.69 | Firmicutes | Clostridia | Oscillospirales |
| Fusobacteriaceae | DM | Yes | 1.07 | 0.36 | 0.004 | 0.049 | 59 | * | 1.76 | 0.37 | Fusobacteriota | Fusobacteriia | Fusobacteriales |
| Fusobacteriaceae | Obese | Obese | 1.39 | 0.24 | <0.001 | <0.001 | 59 | *** | 1.87 | 0.92 | Fusobacteriota | Fusobacteriia | Fusobacteriales |
| Porphyromonadaceae | Obese | Obese | 3.05 | 0.53 | <0.001 | <0.001 | 48 | *** | 4.10 | 2.01 | Bacteroidota | Bacteroidia | Bacteroidales |
| Family XI | Obese | Obese | 1.21 | 0.29 | <0.001 | 0.007 | 59 | ** | 1.78 | 0.63 | Firmicutes | Clostridia | Peptostreptococcales-Tissierellales |
| Enterococcaceae | Obese | Obese | -1.68 | 0.45 | 0.001 | 0.012 | 42 | * | -0.79 | -2.56 | Firmicutes | Bacilli | Lactobacillales |
| Desulfovibrionaceae | Obese | Obese | -1.65 | 0.45 | 0.001 | 0.014 | 48 | * | -0.77 | -2.53 | Desulfobacterota | Desulfovibrionia | Desulfovibrionales |
| Oscillospiraceae | Obese | Obese | -2.04 | 0.56 | 0.001 | 0.015 | 43 | * | -0.93 | -3.14 | Firmicutes | Clostridia | Oscillospirales |
| Ruminococcaceae | Obese | Obese | -1.87 | 0.54 | 0.001 | 0.020 | 43 | * | -0.81 | -2.92 | Firmicutes | Clostridia | Oscillospirales |
| Eubacteriaceae | Obese | Obese | 1.30 | 0.40 | 0.002 | 0.033 | 58 | * | 2.09 | 0.51 | Firmicutes | Clostridia | Eubacteriales |
| Enterococcaceae | Sex | M | 2.19 | 0.49 | <0.001 | 0.003 | 42 | ** | 3.14 | 1.23 | Firmicutes | Bacilli | Lactobacillales |
| Flavobacteriaceae | Age | Age | 0.64 | 0.22 | 0.005 | 0.053 | 58 | | 1.07 | 0.22 | Bacteroidota | Bacteroidia | Flavobacteriales |
| Neisseriaceae | Age | Age | 0.54 | 0.19 | 0.005 | 0.053 | 59 | | 0.91 | 0.18 | Proteobacteria | Gammaproteobacteria | Burkholderiales |
| Tannerellaceae | Age | Age | -0.64 | 0.22 | 0.006 | 0.056 | 57 | | -0.21 | -1.07 | Bacteroidota | Bacteroidia | Bacteroidales |
| Oscillospiraceae | Age | Age | 0.76 | 0.27 | 0.006 | 0.060 | 43 | | 1.28 | 0.24 | Firmicutes | Clostridia | Oscillospirales |
| Ruminococcaceae | Age | Age | 0.71 | 0.25 | 0.008 | 0.068 | 43 | | 1.20 | 0.21 | Firmicutes | Clostridia | Oscillospirales |
| Atopobiaceae | Age | Age | -0.53 | 0.21 | 0.015 | 0.105 | 54 | | -0.12 | -0.94 | Actinobacteriota | Coriobacteriia | Coriobacteriales |
| Pasteurellaceae | Age | Age | -0.53 | 0.22 | 0.020 | 0.126 | 55 | | -0.10 | -0.97 | Proteobacteria | Gammaproteobacteria | Enterobacterales |
| Erysipelatoclostridiaceae | Age | Age | 0.73 | 0.31 | 0.021 | 0.128 | 46 | | 1.34 | 0.13 | Firmicutes | Bacilli | Erysipelotrichales |

| | | | | | | | | | | | | | |
|---|---|---|---|---|---|---|---|---|---|---|---|---|---|
| Campylobacteraceae | Age | Age | -0.54 | 0.25 | 0.032 | 0.171 | 44 | | -0.06 | -1.02 | Campylobacterota | Campylobacteria | Campylobacterales |
| Anaerovoracaceae | Age | Age | -0.37 | 0.18 | 0.041 | 0.197 | 59 | | -0.03 | -0.72 | Firmicutes | Clostridia | Peptostreptococcales-Tissierellales |
| Muribaculaceae | Age | Age | 0.58 | 0.28 | 0.041 | 0.197 | 44 | | 1.12 | 0.04 | Bacteroidota | Bacteroidia | Bacteroidales |
| Anaerolineaceae | Age | Age | -0.39 | 0.19 | 0.049 | 0.225 | 56 | | -0.01 | -0.76 | Chloroflexi | Anaerolineae | Anaerolineales |
| Desulfovibrionaceae | DM | Yes | -2.63 | 0.93 | 0.007 | 0.064 | 48 | | -0.80 | -4.46 | Desulfobacterota | Desulfovibrionia | Desulfovibrionales |
| Gemellaceae | DM | Yes | 2.05 | 0.76 | 0.010 | 0.080 | 59 | | 3.55 | 0.55 | Firmicutes | Bacilli | Staphylococcales |
| Bacteroidaceae | DM | Yes | -2.77 | 1.07 | 0.012 | 0.092 | 47 | | -0.69 | -4.86 | Bacteroidota | Bacteroidia | Bacteroidales |
| Atopobiaceae | DM | Yes | 1.74 | 0.68 | 0.013 | 0.097 | 54 | | 3.07 | 0.42 | Actinobacteriota | Coriobacteriia | Coriobacteriales |
| Ruminococcaceae | DM | Yes | -2.68 | 1.13 | 0.022 | 0.131 | 43 | | -0.47 | -4.90 | Firmicutes | Clostridia | Oscillospirales |
| Selenomonadaceae | DM | Yes | 1.37 | 0.60 | 0.026 | 0.149 | 41 | | 2.55 | 0.20 | Firmicutes | Negativicutes | Veillonellales-Selenomonadales |
| Propionibacteriaceae | DM | Yes | -1.80 | 0.84 | 0.038 | 0.188 | 46 | | -0.15 | -3.46 | Actinobacteriota | Actinobacteria | Propionibacteriales |
| Saccharimonadaceae | DM | Yes | 1.59 | 0.75 | 0.038 | 0.188 | 48 | | 3.06 | 0.13 | Patescibacteria | Saccharimonadia | Saccharimonadales |
| Erysipelatoclostridiaceae | Obese | Obese | -1.90 | 0.64 | 0.005 | 0.053 | 46 | | -0.64 | -3.16 | Firmicutes | Bacilli | Erysipelotrichales |
| Clostridiaceae | Obese | Obese | -1.94 | 0.67 | 0.006 | 0.057 | 38 | | -0.62 | -3.26 | Firmicutes | Clostridia | Clostridiales |
| Muribaculaceae | Obese | Obese | -1.63 | 0.59 | 0.008 | 0.069 | 44 | | -0.48 | -2.78 | Bacteroidota | Bacteroidia | Bacteroidales |
| Pseudomonadaceae | Obese | Obese | -0.92 | 0.33 | 0.008 | 0.070 | 55 | | -0.27 | -1.57 | Proteobacteria | Gammaproteobacteria | Pseudomonadales |
| Gemellaceae | Obese | Obese | -1.28 | 0.47 | 0.010 | 0.079 | 59 | | -0.35 | -2.21 | Firmicutes | Bacilli | Staphylococcales |
| Bacteroidaceae | Obese | Obese | -1.28 | 0.50 | 0.014 | 0.100 | 47 | | -0.30 | -2.27 | Bacteroidota | Bacteroidia | Bacteroidales |
| Anaerolineaceae | Obese | Obese | 1.08 | 0.42 | 0.014 | 0.100 | 56 | | 1.91 | 0.25 | Chloroflexi | Anaerolineae | Anaerolineales |
| Xanthomonadaceae | Obese | Obese | -1.72 | 0.68 | 0.015 | 0.103 | 30 | | -0.39 | -3.05 | Proteobacteria | Gammaproteobacteria | Xanthomonadales |
| Akkermansiaceae | Obese | Obese | -1.49 | 0.65 | 0.026 | 0.148 | 34 | | -0.22 | -2.76 | Verrucomicrobiota | Verrucomicrobiae | Verrucomicrobiales |
| Rikenellaceae | Obese | Obese | -1.27 | 0.58 | 0.033 | 0.172 | 44 | | -0.14 | -2.41 | Bacteroidota | Bacteroidia | Bacteroidales |
| Pasteurellaceae | Sex | M | 1.22 | 0.49 | 0.015 | 0.106 | 55 | | 2.17 | 0.27 | Proteobacteria | Gammaproteobacteria | Enterobacterales |
| Erysipelotrichaceae | Sex | M | -1.05 | 0.42 | 0.016 | 0.106 | 54 | | -0.23 | -1.87 | Firmicutes | Bacilli | Erysipelotrichales |
| Eubacteriaceae | Sex | M | -1.00 | 0.42 | 0.020 | 0.126 | 58 | | -0.18 | -1.82 | Firmicutes | Clostridia | Eubacteriales |
| Comamonadaceae | Sex | M | -1.51 | 0.64 | 0.022 | 0.133 | 47 | | -0.26 | -2.76 | Proteobacteria | Gammaproteobacteria | Burkholderiales |
| Atopobiaceae | Sex | M | -1.00 | 0.49 | 0.048 | 0.222 | 54 | | -0.03 | -1.97 | Actinobacteriota | Coriobacteriia | Coriobacteriales |
| Neisseriaceae | Smoker | Former | -2.31 | 0.78 | 0.005 | 0.052 | 59 | | -0.79 | -3.83 | Proteobacteria | Gammaproteobacteria | Burkholderiales |
| Propionibacteriaceae | Smoker | Former | 2.55 | 0.93 | 0.009 | 0.074 | 46 | | 4.37 | 0.72 | Actinobacteriota | Actinobacteria | Propionibacteriales |
| Clostridiaceae | Smoker | Never | -2.46 | 1.01 | 0.018 | 0.115 | 38 | | -0.49 | -4.44 | Firmicutes | Clostridia | Clostridiales |
| Selenomonadaceae | Smoker | Former | 2.17 | 0.93 | 0.024 | 0.139 | 41 | | 4.00 | 0.35 | Firmicutes | Negativicutes | Veillonellales-Selenomonadales |
| Leptotrichiaceae | Smoker | Former | -2.20 | 0.95 | 0.025 | 0.143 | 50 | | -0.34 | -4.05 | Fusobacteriota | Fusobacteriia | Fusobacteriales |

| | | | | | | | | | | | | | |
|---|---|---|---|---|---|---|---|---|---|---|---|---|---|
| Actinomycetaceae | Smoker | Former | 1.36 | 0.59 | 0.026 | 0.149 | 59 | | 2.53 | 0.20 | Actinobacteriota | Actinobacteria | Actinomycetales |
| Tannerellaceae | Smoker | Never | -1.80 | 0.84 | 0.037 | 0.186 | 57 | | -0.15 | -3.44 | Bacteroidota | Bacteroidia | Bacteroidales |
| Eggerthellaceae | Smoker | Former | 1.75 | 0.87 | 0.050 | 0.227 | 39 | | 3.45 | 0.05 | Actinobacteriota | Coriobacteriia | Coriobacteriales |

8.2 Genus

| **Genus** | **meta** | **value** | **coef** | **stderr** | **pval** | **qval** | **N. not. zero** | **sig.** | **upper ci** | **lower ci** | **Phylum** | **Class** | **Order** | **Family** |
|---|---|---|---|---|---|---|---|---|---|---|---|---|---|---|
| Sphingomonas | Age | Age | -0.97 | 0.21 | <0.001 | 0.002 | 34 | ** | -0.56 | -1.39 | Proteobacteria | Alpha-proteobacteria | Sphingomonadales | Sphingomonadaceae |
| Lautropia | Age | Age | 0.58 | 0.14 | <0.001 | 0.007 | 54 | ** | 0.85 | 0.30 | Proteobacteria | Gamma-proteobacteria | Burkholderiales | Burkholderiaceae |
| F0332 | Age | Age | -0.69 | 0.20 | 0.001 | 0.024 | 45 | * | -0.30 | -1.08 | Actinobacteriota | Actinobacteria | Actinomycetales | Actinomycetaceae |
| Pseudoramibacter | Age | Age | -0.60 | 0.18 | 0.002 | 0.037 | 58 | * | -0.24 | -0.97 | Firmicutes | Clostridia | Eubacteriales | Eubacteriaceae |
| TM7x | Age | Age | 0.85 | 0.26 | 0.002 | 0.042 | 30 | * | 1.37 | 0.33 | Patescibacteria | Saccharimonadia | Saccharimonadales | Saccharimonadaceae |
| Tannerella | Age | Age | -0.79 | 0.25 | 0.003 | 0.046 | 54 | * | -0.30 | -1.28 | Bacteroidota | Bacteroidia | Bacteroidales | Tannerellaceae |
| Prevotella | DM | Yes | 2.68 | 0.72 | <0.001 | 0.015 | 44 | * | 4.09 | 1.28 | Bacteroidota | Bacteroidia | Bacteroidales | Prevotellaceae |
| Haemophilus | DM | Yes | -3.83 | 1.08 | 0.001 | 0.021 | 49 | * | -1.72 | -5.95 | Proteobacteria | Gamma-proteobacteria | Enterobacterales | Pasteurellaceae |
| Sphingomonas | Obese | Obese | -3.33 | 0.51 | <0.001 | <0.001 | 34 | *** | -2.33 | -4.33 | Proteobacteria | Alpha-proteobacteria | Sphingomonadales | Sphingomonadaceae |
| Fusobacterium | Obese | Obese | 1.40 | 0.24 | <0.001 | <0.001 | 59 | *** | 1.87 | 0.92 | Fusobacteriota | Fusobacteriia | Fusobacteriales | Fusobacteriaceae |
| Porphyromonas | Obese | Obese | 2.98 | 0.53 | <0.001 | <0.001 | 48 | *** | 4.02 | 1.94 | Bacteroidota | Bacteroidia | Bacteroidales | Porphyromonadaceae |
| Moryella | Obese | Obese | 1.76 | 0.42 | <0.001 | 0.005 | 49 | ** | 2.59 | 0.94 | Firmicutes | Clostridia | Lachnospirales | Lachnospiraceae |
| W5053 | Obese | Obese | 1.42 | 0.36 | <0.001 | 0.009 | 59 | ** | 2.12 | 0.71 | Firmicutes | Clostridia | Peptostreptococcales-Tissierellales | Family XI |
| Enterococcus | Obese | Obese | -1.72 | 0.46 | <0.001 | 0.015 | 42 | * | -0.82 | -2.62 | Firmicutes | Bacilli | Lactobacillales | Enterococcaceae |
| Desulfovibrio | Obese | Obese | -1.58 | 0.47 | 0.001 | 0.031 | 44 | * | -0.66 | -2.49 | Desulfobacterota | Desulfovibrionia | Desulfovibrionales | Desulfovibrionaceae |
| Aggregatibacter | Obese | Obese | 1.79 | 0.54 | 0.002 | 0.034 | 39 | * | 2.85 | 0.73 | Proteobacteria | Gamma-proteobacteria | Enterobacterales | Pasteurellaceae |
| Pseudoramibacter | Obese | Obese | 1.27 | 0.40 | 0.003 | 0.046 | 58 | * | 2.05 | 0.48 | Firmicutes | Clostridia | Eubacteriales | Eubacteriaceae |
| Enterococcus | Sex | M | 2.13 | 0.49 | <0.001 | 0.004 | 42 | ** | 3.10 | 1.16 | Firmicutes | Bacilli | Lactobacillales | Enterococcaceae |
| [Eubacterium] saphenum group | Sex | M | 2.37 | 0.62 | <0.001 | 0.012 | 37 | * | 3.59 | 1.16 | Firmicutes | Clostridia | Peptostreptococcales-Tissierellales | Anaerovoracaceae |

| | | | | | | | | | | | | | | |
|---|---|---|---|---|---|---|---|---|---|---|---|---|---|---|
| Lautropia | Smoker | Never | 2.19 | 0.69 | 0.003 | 0.044 | 54 | * | 3.53 | 0.84 | Proteobacteria | Gamma-proteobacteria | Burkholderiales | Burkholderiaceae |
| Actinomyces | Smoker | Former | 2.02 | 0.64 | 0.003 | 0.046 | 59 | * | 3.27 | 0.76 | Actinobacteriota | Actinobacteria | Actinomycetales | Actinomycetaceae |
| Solobacterium | Age | Age | -0.78 | 0.32 | 0.017 | 0.141 | 31 | | -0.16 | -1.40 | Firmicutes | Bacilli | Erysipelotrichales | Erysipelotrichaceae |
| [Eubacterium] nodatum group | Age | Age | -0.68 | 0.22 | 0.004 | 0.056 | 58 | | -0.24 | -1.12 | Firmicutes | Clostridia | Peptostreptococcales-Tissierellales | Anaerovoracaceae |
| Campylobacter | Age | Age | -0.54 | 0.25 | 0.034 | 0.218 | 44 | | -0.05 | -1.02 | Campylobacterota | Campylobacteria | Campylobacterales | Campylobacteraceae |
| Olsenella | Age | Age | -0.52 | 0.23 | 0.030 | 0.199 | 46 | | -0.06 | -0.97 | Actinobacteriota | Coriobacteriia | Coriobacteriales | Atopobiaceae |
| Fusobacterium | Age | Age | 0.22 | 0.11 | 0.042 | 0.244 | 59 | | 0.43 | 0.01 | Fusobacteriota | Fusobacteriia | Fusobacteriales | Fusobacteriaceae |
| Neisseria | Age | Age | 0.56 | 0.19 | 0.004 | 0.060 | 59 | | 0.92 | 0.19 | Proteobacteria | Gamma-proteobacteria | Burkholderiales | Neisseriaceae |
| [Ruminococcus] torques group | Age | Age | 0.60 | 0.24 | 0.018 | 0.146 | 34 | | 1.07 | 0.12 | Firmicutes | Clostridia | Lachnospirales | Lachnospiraceae |
| Enterococcus | Age | Age | 0.66 | 0.22 | 0.004 | 0.057 | 42 | | 1.08 | 0.23 | Firmicutes | Bacilli | Lactobacillales | Enterococcaceae |
| Capnocytophaga | Age | Age | 0.66 | 0.23 | 0.005 | 0.067 | 57 | | 1.11 | 0.22 | Bacteroidota | Bacteroidia | Flavobacteriales | Flavobacteriaceae |
| Eikenella | Age | Age | 0.72 | 0.25 | 0.007 | 0.081 | 31 | | 1.22 | 0.22 | Proteobacteria | Gamma-proteobacteria | Burkholderiales | Neisseriaceae |
| Veillonella | Age | Age | 0.72 | 0.27 | 0.009 | 0.098 | 52 | | 1.24 | 0.20 | Firmicutes | Negativicutes | Veillonellales-Selenomonadales | Veillonellaceae |
| Rikenellaceae RC9 gut group | Age | Age | 0.89 | 0.32 | 0.007 | 0.082 | 40 | | 1.51 | 0.27 | Bacteroidota | Bacteroidia | Bacteroidales | Rikenellaceae |
| Rikenellaceae RC9 gut group | DM | Yes | -4.45 | 1.62 | 0.008 | 0.092 | 40 | | -1.27 | -7.63 | Bacteroidota | Bacteroidia | Bacteroidales | Rikenellaceae |
| Bacteroides | DM | Yes | -2.80 | 1.09 | 0.014 | 0.124 | 47 | | -0.66 | -4.93 | Bacteroidota | Bacteroidia | Bacteroidales | Bacteroidaceae |
| Aggregatibacter | DM | Yes | -2.66 | 1.10 | 0.019 | 0.154 | 39 | | -0.51 | -4.81 | Proteobacteria | Gamma-proteobacteria | Enterobacterales | Pasteurellaceae |
| Desulfovibrio | DM | Yes | -2.47 | 0.98 | 0.015 | 0.133 | 44 | | -0.55 | -4.40 | Desulfobacterota | Desulfovibrionia | Desulfovibrionales | Desulfovibrionaceae |
| Fusobacterium | DM | Yes | 1.09 | 0.36 | 0.004 | 0.054 | 59 | | 1.79 | 0.39 | Fusobacteriota | Fusobacteriia | Fusobacteriales | Fusobacteriaceae |
| Moryella | DM | Yes | 1.53 | 0.56 | 0.009 | 0.095 | 49 | | 2.63 | 0.43 | Firmicutes | Clostridia | Lachnospirales | Lachnospiraceae |
| [Eubacterium] saphenum group | DM | Yes | 1.96 | 0.86 | 0.026 | 0.184 | 37 | | 3.64 | 0.28 | Firmicutes | Clostridia | Peptostreptococcales-Tissierellales | Anaerovoracaceae |
| Olsenella | DM | Yes | 2.02 | 0.72 | 0.007 | 0.082 | 46 | | 3.42 | 0.61 | Actinobacteriota | Coriobacteriia | Coriobacteriales | Atopobiaceae |
| Gemella | DM | Yes | 2.07 | 0.77 | 0.010 | 0.101 | 59 | | 3.58 | 0.56 | Firmicutes | Bacilli | Staphylococcales | Gemellaceae |
| Lachnoanaero-baculum | DM | Yes | 2.13 | 0.72 | 0.005 | 0.066 | 38 | | 3.55 | 0.71 | Firmicutes | Clostridia | Lachnospirales | Lachnospiraceae |

| | | | | | | | | | | | | | | |
|---|---|---|---|---|---|---|---|---|---|---|---|---|---|---|
| Clostridium sensu stricto 1 | Obese | Obese | -1.94 | 0.67 | 0.006 | 0.070 | 37 | | -0.63 | -3.26 | Firmicutes | Clostridia | Clostridiales | Clostridiaceae |
| Akkermansia | Obese | Obese | -1.60 | 0.66 | 0.019 | 0.154 | 34 | | -0.31 | -2.90 | Verruco-microbiota | Verrucomicrobiae | Verrucomicrobiales | Akkermansiaceae |
| Lactobacillus | Obese | Obese | -1.59 | 0.56 | 0.006 | 0.076 | 44 | | -0.50 | -2.68 | Firmicutes | Bacilli | Lactobacillales | Lactobacillaceae |
| Rikenellaceae RC9 gut group | Obese | Obese | -1.51 | 0.67 | 0.029 | 0.195 | 40 | | -0.20 | -2.83 | Bacteroidota | Bacteroidia | Bacteroidales | Rikenellaceae |
| Faecalibacterium | Obese | Obese | -1.51 | 0.52 | 0.006 | 0.070 | 33 | | -0.49 | -2.53 | Firmicutes | Clostridia | Oscillospirales | Ruminococcaceae |
| Bacteroides | Obese | Obese | -1.39 | 0.52 | 0.010 | 0.104 | 47 | | -0.37 | -2.40 | Bacteroidota | Bacteroidia | Bacteroidales | Bacteroidaceae |
| Gemella | Obese | Obese | -1.31 | 0.48 | 0.008 | 0.092 | 59 | | -0.37 | -2.25 | Firmicutes | Bacilli | Staphylococcales | Gemellaceae |
| [Ruminococcus] torques group | Obese | Obese | -1.06 | 0.51 | 0.041 | 0.242 | 34 | | -0.07 | -2.06 | Firmicutes | Clostridia | Lachnospirales | Lachnospiraceae |
| Pseudomonas | Obese | Obese | -0.95 | 0.33 | 0.006 | 0.073 | 55 | | -0.30 | -1.60 | Proteobacteria | Gamma-proteobacteria | Pseudomonadales | Pseudomonadaceae |
| Filifactor | Obese | Obese | 0.94 | 0.42 | 0.030 | 0.199 | 59 | | 1.76 | 0.12 | Firmicutes | Clostridia | Peptostreptococcales-Tissierellales | Peptostrepto-coccaceae |
| Parvimonas | Obese | Obese | 0.97 | 0.37 | 0.011 | 0.108 | 56 | | 1.69 | 0.25 | Firmicutes | Clostridia | Peptostreptococcales-Tissierellales | Family XI |
| Flexilinea | Obese | Obese | 1.08 | 0.42 | 0.014 | 0.125 | 56 | | 1.91 | 0.25 | Chloroflexi | Anaerolineae | Anaerolineales | Anaerolineaceae |
| TM7x | Obese | Obese | 1.59 | 0.61 | 0.012 | 0.114 | 30 | | 2.78 | 0.40 | Patescibacteria | Saccharimonadia | Saccharimonadales | Saccharimonadaceae |
| Prevotella | Obese | Obese | 1.63 | 0.56 | 0.005 | 0.067 | 44 | | 2.73 | 0.54 | Bacteroidota | Bacteroidia | Bacteroidales | Prevotellaceae |
| Bulleidia | Sex | M | -2.19 | 0.72 | 0.004 | 0.057 | 34 | | -0.78 | -3.60 | Firmicutes | Bacilli | Erysipelotrichales | Erysipelotrichaceae |
| Atopobium | Sex | M | -1.53 | 0.64 | 0.020 | 0.159 | 35 | | -0.28 | -2.77 | Actinobacteriota | Coriobacteriia | Coriobacteriales | Atopobiaceae |
| [Eubacterium] brachy group | Sex | M | -1.26 | 0.42 | 0.004 | 0.057 | 59 | | -0.44 | -2.07 | Firmicutes | Clostridia | Peptostreptococcales-Tissierellales | Anaerovoracaceae |
| Pseudoramibacter | Sex | M | -0.96 | 0.42 | 0.026 | 0.183 | 58 | | -0.14 | -1.78 | Firmicutes | Clostridia | Eubacteriales | Eubacteriaceae |
| Lautropia | Sex | M | 0.91 | 0.31 | 0.004 | 0.060 | 54 | | 1.51 | 0.32 | Proteobacteria | Gamma-proteobacteria | Burkholderiales | Burkholderiaceae |
| Ligilactobacillus | Sex | M | 1.25 | 0.54 | 0.023 | 0.174 | 42 | | 2.30 | 0.20 | Firmicutes | Bacilli | Lactobacillales | Lactobacillaceae |
| Haemophilus | Sex | M | 1.60 | 0.53 | 0.004 | 0.057 | 49 | | 2.63 | 0.56 | Proteobacteria | Gamma-proteobacteria | Enterobacterales | Pasteurellaceae |
| Clostridium sensu stricto 1 | Smoker | Never | -2.47 | 1.00 | 0.017 | 0.144 | 37 | | -0.51 | -4.43 | Firmicutes | Clostridia | Clostridiales | Clostridiaceae |
| Bulleidia | Smoker | Never | -2.44 | 1.05 | 0.024 | 0.176 | 34 | | -0.38 | -4.49 | Firmicutes | Bacilli | Erysipelotrichales | Erysipelotrichaceae |

| | | | | | | | | | | | | | | |
|---|---|---|---|---|---|---|---|---|---|---|---|---|---|---|
| Eikenella | Smoker | Former | -2.37 | 1.07 | 0.031 | 0.203 | 31 | | -0.28 | -4.46 | Proteobacteria | Gamma-proteobacteria | Burkholderiales | Neisseriaceae |
| Leptotrichia | Smoker | Former | -2.35 | 0.96 | 0.018 | 0.148 | 49 | | -0.47 | -4.22 | Fusobacteriota | Fusobacteriia | Fusobacteriales | Leptotrichiaceae |
| Neisseria | Smoker | Former | -2.27 | 0.78 | 0.005 | 0.069 | 59 | | -0.74 | -3.81 | Proteobacteria | Gamma-proteobacteria | Burkholderiales | Neisseriaceae |
| Tannerella | Smoker | Never | -2.20 | 0.95 | 0.024 | 0.178 | 54 | | -0.34 | -4.07 | Bacteroidota | Bacteroidia | Bacteroidales | Tannerellaceae |
| Lautropia | Smoker | Former | 1.95 | 0.72 | 0.009 | 0.098 | 54 | | 3.35 | 0.54 | Proteobacteria | Gamma-proteobacteria | Burkholderiales | Burkholderiaceae |
| [Eubacterium] saphenum group | Smoker | Never | 2.22 | 1.03 | 0.036 | 0.225 | 37 | | 4.25 | 0.20 | Firmicutes | Clostridia | Peptostreptococcales-Tissierellales | Anaerovoracaceae |
| Pseudopropioni-bacterium | Smoker | Former | 2.83 | 1.06 | 0.010 | 0.102 | 40 | | 4.90 | 0.76 | Actinobacteriota | Actinobacteria | Propionibacteriales | Propionibacteriaceae |

8.3 Species

| Species | meta | value | coef | stderr | pval | qval | N. not. zero | sig. | upper ci | lower ci | Phylum | Class | Order | Family |
|---|---|---|---|---|---|---|---|---|---|---|---|---|---|---|
| [Eubacterium] nodatum_minutum | Age | Age | -1.07 | 0.26 | <0.001 | 0.006 | 56 | ** | -0.56 | -1.59 | Firmicutes | Clostridia | Peptostreptococcales-Tissierellales | Anaerovoracaceae |
| Actinomyces_oris | Age | Age | -0.95 | 0.25 | <0.001 | 0.011 | 30 | * | -0.46 | -1.44 | Actinobacteriota | Actinobacteria | Actinomycetales | Actinomycetaceae |
| Gemella_ morbillorum | Age | Age | 0.40 | 0.12 | 0.002 | 0.028 | 58 | * | 0.64 | 0.17 | Firmicutes | Bacilli | Staphylococcales | Gemellaceae |
| Actinomyces_ naeslundii | Age | Age | -0.63 | 0.20 | 0.003 | 0.041 | 44 | * | -0.24 | -1.02 | Actinobacteriota | Actinobacteria | Actinomycetales | Actinomycetaceae |
| Actinomyces_ gerencseriae | Age | Age | -0.96 | 0.31 | 0.003 | 0.047 | 33 | * | -0.35 | -1.57 | Actinobacteriota | Actinobacteria | Actinomycetales | Actinomycetaceae |
| Haemophilus_ parainfluenzae | DM | Yes | -3.57 | 1.05 | 0.001 | 0.025 | 49 | * | -1.52 | -5.63 | Proteobacteria | Gamma-proteobacteria | Enterobacterales | Pasteurellaceae |
| Fusobacterium_ nucleatum | Obese | Obese | 1.42 | 0.26 | <0.001 | <0.001 | 59 | *** | 1.94 | 0.91 | Fusobacteriota | Fusobacteriia | Fusobacteriales | Fusobacteriaceae |
| Porphyromonas_ gingivalis | Obese | Obese | 2.85 | 0.60 | <0.001 | 0.001 | 44 | ** | 4.02 | 1.67 | Bacteroidota | Bacteroidia | Bacteroidales | Porphyromonadaceae |
| [Eubacterium] saphenum_saphenum | Sex | M | 3.05 | 0.67 | <0.001 | 0.002 | 34 | ** | 4.36 | 1.73 | Firmicutes | Clostridia | Peptostreptococcales-Tissierellales | Anaerovoracaceae |
| Streptococcus_ cristatus | Sex | M | -1.77 | 0.51 | 0.001 | 0.020 | 51 | * | -0.78 | -2.77 | Firmicutes | Bacilli | Lactobacillales | Streptococcaceae |
| Neisseria_mucosa | Smoker | Former | -2.65 | 0.78 | 0.001 | 0.025 | 59 | * | -1.12 | -4.19 | Proteobacteria | Gamma-proteobacteria | Burkholderiales | Neisseriaceae |

| | | | | | | | | | | | | | | |
|---|---|---|---|---|---|---|---|---|---|---|---|---|---|---|
| Escherichia-Shigella_coli | Smoker | Former | 3.89 | 1.16 | 0.002 | 0.028 | 43 | * | 6.17 | 1.61 | Proteobacteria | Gamma-proteobacteria | Enterobacterales | Enterobacteriaceae |
| Solobacterium_moorei | Age | Age | -0.66 | 0.31 | 0.036 | 0.216 | 31 | | -0.06 | -1.27 | Firmicutes | Bacilli | Erysipelotrichales | Erysipelotrichaceae |
| Fusobacterium_nucleatum | Age | Age | 0.26 | 0.12 | 0.029 | 0.193 | 59 | | 0.49 | 0.03 | Fusobacteriota | Fusobacteriia | Fusobacteriales | Fusobacteriaceae |
| Capnocytophaga_sputigena | Age | Age | 0.42 | 0.17 | 0.020 | 0.154 | 57 | | 0.76 | 0.08 | Bacteroidota | Bacteroidia | Flavobacteriales | Flavobacteriaceae |
| Neisseria_mucosa | Age | Age | 0.47 | 0.19 | 0.016 | 0.136 | 59 | | 0.83 | 0.10 | Proteobacteria | Gamma-proteobacteria | Burkholderiales | Neisseriaceae |
| Veillonella_parvula | Age | Age | 0.67 | 0.26 | 0.014 | 0.122 | 51 | | 1.18 | 0.16 | Firmicutes | Negativicutes | Veillonellales-Selenomonadales | Veillonellaceae |
| Capnocytophaga_gingivalis | Age | Age | 0.73 | 0.28 | 0.012 | 0.109 | 33 | | 1.28 | 0.18 | Bacteroidota | Bacteroidia | Flavobacteriales | Flavobacteriaceae |
| Streptococcus_intermedius | DM | Yes | -2.74 | 0.98 | 0.007 | 0.078 | 54 | | -0.82 | -4.66 | Firmicutes | Bacilli | Lactobacillales | Streptococcaceae |
| Gemella_morbillorum | DM | Yes | -1.04 | 0.47 | 0.032 | 0.204 | 58 | | -0.12 | -1.97 | Firmicutes | Bacilli | Staphylococcales | Gemellaceae |
| Fusobacterium_nucleatum | DM | Yes | 0.99 | 0.39 | 0.015 | 0.132 | 59 | | 1.77 | 0.22 | Fusobacteriota | Fusobacteriia | Fusobacteriales | Fusobacteriaceae |
| Actinomyces_oris | DM | Yes | 1.53 | 0.70 | 0.033 | 0.207 | 30 | | 2.91 | 0.16 | Actinobacteriota | Actinobacteria | Actinomycetales | Actinomycetaceae |
| Escherichia-Shigella_coli | Obese | Obese | -1.28 | 0.55 | 0.023 | 0.168 | 43 | | -0.21 | -2.35 | Proteobacteria | Gamma-proteobacteria | Enterobacterales | Enterobacteriaceae |
| Rothia_aeria | Obese | Obese | -1.09 | 0.48 | 0.026 | 0.179 | 59 | | -0.16 | -2.02 | Actinobacteriota | Actinobacteria | Micrococcales | Micrococcaceae |
| Filifactor_alocis | Obese | Obese | 0.97 | 0.43 | 0.031 | 0.199 | 59 | | 1.82 | 0.11 | Firmicutes | Clostridia | Peptostreptococcales-Tissierellales | Peptostreptococcaceae |
| Bulleidia_extructa | Sex | M | -2.18 | 0.72 | 0.004 | 0.053 | 34 | | -0.76 | -3.59 | Firmicutes | Bacilli | Erysipelotrichales | Erysipelotrichaceae |
| Akkermansia_muciniphila | Sex | M | -1.49 | 0.67 | 0.031 | 0.199 | 32 | | -0.18 | -2.81 | Verruco-microbiota | Verruco-microbiae | Verrucomicrobiales | Akkermansiaceae |
| [Eubacterium] brachy_brachy | Sex | M | -1.19 | 0.41 | 0.006 | 0.067 | 59 | | -0.38 | -1.99 | Firmicutes | Clostridia | Peptostreptococcales-Tissierellales | Anaerovoracaceae |
| Streptococcus_oralis | Sex | M | -0.89 | 0.40 | 0.029 | 0.191 | 59 | | -0.11 | -1.67 | Firmicutes | Bacilli | Lactobacillales | Streptococcaceae |
| Haemophilus_parainfluenzae | Sex | M | 1.26 | 0.52 | 0.019 | 0.151 | 49 | | 2.28 | 0.24 | Proteobacteria | Gamma-proteobacteria | Enterobacterales | Pasteurellaceae |
| Streptococcus_intermedius | Sex | M | 1.35 | 0.51 | 0.010 | 0.101 | 54 | | 2.34 | 0.36 | Firmicutes | Bacilli | Lactobacillales | Streptococcaceae |
| Enterococcus_faecium | Sex | M | 1.39 | 0.59 | 0.022 | 0.164 | 36 | | 2.55 | 0.24 | Firmicutes | Bacilli | Lactobacillales | Enterococcaceae |
| Neisseria_elongata | Smoker | Former | -2.22 | 0.91 | 0.019 | 0.149 | 49 | | -0.43 | -4.02 | Proteobacteria | Gamma-proteobacteria | Burkholderiales | Neisseriaceae |
| Capnocytophaga_sputigena | Smoker | Former | -1.77 | 0.76 | 0.025 | 0.173 | 57 | | -0.27 | -3.27 | Bacteroidota | Bacteroidia | Flavobacteriales | Flavobacteriaceae |

| | | | | | | | | | | | | | | |
|---|---|---|---|---|---|---|---|---|---|---|---|---|---|---|
| Filifactor_alocis | Smoker | Never | 1.84 | 0.80 | 0.026 | 0.179 | 59 | | 3.42 | 0.27 | Firmicutes | Clostridia | Peptostreptococcales-Tissierellales | Peptostreptococcaceae |
| Rothia_aeria | Smoker | Former | 2.11 | 0.93 | 0.027 | 0.185 | 59 | | 3.92 | 0.29 | Actinobacteriota | Actinobacteria | Micrococcales | Micrococcaceae |
| [Eubacterium] saphenum_saphenum | Smoker | Never | 2.15 | 1.05 | 0.046 | 0.248 | 34 | | 4.20 | 0.09 | Firmicutes | Clostridia | Peptostreptococcales-Tissierellales | Anaerovoracaceae |
| Streptococcus_ intermedius | Smoker | Never | 2.21 | 0.97 | 0.027 | 0.185 | 54 | | 4.11 | 0.30 | Firmicutes | Bacilli | Lactobacillales | Streptococcaceae |
| Rothia_aeria | Smoker | Never | 2.23 | 0.88 | 0.014 | 0.125 | 59 | | 3.95 | 0.51 | Actinobacteriota | Actinobacteria | Micrococcales | Micrococcaceae |
| Actinomyces_oris | Smoker | Former | 2.48 | 1.08 | 0.026 | 0.178 | 30 | | 4.59 | 0.37 | Actinobacteriota | Actinobacteria | Actinomycetales | Actinomycetaceae |
| Actinomyces_oris | Smoker | Never | 2.82 | 1.17 | 0.020 | 0.154 | 30 | | 5.11 | 0.52 | Actinobacteriota | Actinobacteria | Actinomycetales | Actinomycetaceae |
| Actinomyces_ gerencseriae | Smoker | Former | 3.74 | 1.42 | 0.011 | 0.106 | 33 | | 6.52 | 0.97 | Actinobacteriota | Actinobacteria | Actinomycetales | Actinomycetaceae |

Sig.: significant; * q < 0.05, ** q < 0.01, *** q < 0.001.

## Table 9. Table sPLS-DA loading of importance of genera to types of tissue

Ordered by the absolute values of importance (loading)

| Genus | Osseous (GT) | Periodontium (PT) | Root (RT) | Socket (ST) | GroupContrib | importance |
|---|---|---|---|---|---|---|
| Rothia | 0.02 | 0.62 | -0.91 | -0.89 | Periodontium | 0.16 |
| Family XIII UCG-001 | -0.47 | -0.41 | 0.40 | 0.96 | Socket | -0.15 |
| Leptotrichia | -0.27 | -0.27 | 0.67 | 0.76 | Socket | -0.15 |
| Lautropia | 0.02 | -0.01 | -1.01 | -0.49 | Osseous | 0.12 |
| Porphyromonas | -0.23 | -0.49 | 0.98 | 0.28 | Root | -0.12 |
| Capnocytophaga | 0.11 | 0.29 | -0.74 | -0.20 | Periodontium | 0.12 |
| Parvimonas | -0.22 | -0.33 | 0.91 | 0.67 | Root | -0.11 |
| Peptococcus | -0.38 | 0.09 | 0.61 | 0.32 | Root | -0.11 |
| [Eubacterium] brachy group | -0.68 | -0.72 | 0.72 | 0.60 | Root | -0.11 |
| W5053 | -0.36 | -0.03 | -0.47 | 1.58 | Socket | -0.11 |
| Peptostreptococcus | -0.11 | -0.19 | 0.97 | -0.11 | Root | -0.10 |
| Filifactor | -0.54 | -0.37 | 1.07 | 1.10 | Socket | -0.10 |
| [Eubacterium] nodatum group | -0.49 | -0.28 | 0.47 | 0.77 | Socket | -0.10 |
| Haemophilus | 0.14 | -0.03 | -0.31 | -0.42 | Osseous | 0.10 |
| Neisseria | 0.45 | 0.22 | -0.84 | -0.51 | Osseous | 0.10 |
| Desulfobulbus | -0.37 | -0.20 | 1.21 | 0.23 | Root | -0.10 |
| Fretibacterium | -0.23 | -0.26 | 0.77 | 0.43 | Root | -0.08 |
| Fusobacterium | 0.26 | -0.39 | 0.49 | 0.63 | Socket | -0.07 |
| Tannerella | -0.20 | -0.52 | 1.39 | -0.19 | Root | -0.07 |
| Flexilinea | -0.37 | -0.57 | 0.76 | 0.67 | Root | -0.06 |
| Campylobacter | -0.15 | -0.24 | 0.90 | -0.68 | Root | 0.06 |
| Dialister | -0.17 | -0.27 | -0.16 | 0.26 | Socket | -0.05 |
| Escherichia-Shigella | 0.57 | -0.69 | -0.01 | 0.25 | Osseous | 0.05 |
| Olsenella | 0.12 | 0.12 | 0.12 | 0.61 | Socket | -0.05 |
| Bulleidia | 0.65 | -0.25 | -0.28 | -0.60 | Osseous | 0.05 |
| Lachnospiraceae NK4A136 group | -0.38 | 0.27 | -0.38 | -0.38 | Periodontium | 0.05 |
| Akkermansia | 0.02 | 0.02 | 0.11 | 0.02 | Root | -0.05 |
| Erysipelatoclostridium | 0.79 | -0.19 | -0.86 | 0.49 | Osseous | 0.05 |
| Gemella | -0.29 | -0.14 | -0.31 | -0.05 | Socket | -0.04 |
| Pseudopropionibacterium | -0.02 | 0.07 | 0.21 | 0.73 | Socket | -0.04 |
| Ralstonia | 0.36 | -0.07 | -1.22 | 0.97 | Socket | 0.04 |
| Treponema | -0.34 | -0.50 | 0.85 | -0.49 | Root | -0.04 |
| Mogibacterium | -0.54 | -0.54 | -0.04 | 0.29 | Socket | -0.04 |
| Lactobacillus | -0.08 | 0.53 | -0.08 | 0.18 | Periodontium | 0.03 |
| Ligilactobacillus | -0.25 | 0.40 | -0.44 | -0.25 | Periodontium | 0.03 |
| [Eubacterium] saphenum group | 0.16 | 0.16 | 0.43 | 0.32 | Root | -0.03 |
| Granulicatella | -0.12 | -0.26 | -0.66 | -0.40 | Osseous | 0.03 |
| Lachnoanaerobaculum | 0.41 | -0.84 | 0.75 | 0.21 | Root | -0.03 |
| Streptococcus | -0.07 | 0.84 | 0.15 | 0.08 | Periodontium | 0.02 |
| Parabacteroides | -0.22 | -0.06 | -0.27 | -0.22 | Periodontium | 0.02 |

| | | | | | | |
|---|---|---|---|---|---|---|
| Moryella | -0.23 | 0.53 | 0.15 | 0.03 | Periodontium | -0.02 |
| F0332 | 0.00 | 0.44 | -0.42 | -0.19 | Periodontium | 0.02 |
| Prevotella | -0.11 | -0.11 | 0.33 | -0.11 | Root | -0.02 |
| Pseudomonas | 0.09 | -0.22 | -0.30 | -0.41 | Osseous | -0.02 |
| Blautia | -0.03 | 0.06 | -0.03 | -0.03 | Periodontium | -0.01 |
| Bifidobacterium | -0.03 | -0.03 | 0.21 | -0.03 | Root | 0.01 |
| Veillonella | -0.12 | -0.06 | 0.32 | -0.12 | Root | 0.00 |
| Atopobium | 0.01 | 0.01 | 0.22 | 0.01 | Root | 0.00 |
| Bacteroides | -0.56 | -0.03 | 0.23 | -0.32 | Root | 0.00 |

**Table 10. Periodontal parameters at tooth sites of paired GT and PT samples**

| Subject | GT Tooth | GT Mean PD | GT Mean CAL | PT Tooth | PT Mean PD | PT Mean CAL | GT PR ratio | PT PR ratio | *P*-value PD GT−PT | *P*-value CAL GT−PT | *P*-value *PR* ratio GT−PT |
|---|---|---|---|---|---|---|---|---|---|---|---|
| **P008** | 27 | 4.0 | 4.7 | 27 | 4.0 | 4.7 | −5.0 | 1.9 | 0.485 | 0.847 | 0.934 |
| **P008** | 37 | 4.5 | 4.8 | | | | −2.6 | | | | |
| **P009** | 36 | 3.0 | 4.0 | 36 | 3.0 | 4.0 | −3.8 | −4.6 | | | |
| **P014** | 16 | 4.5 | 6.7 | 16 | 4.5 | 6.7 | −1.9 | −5.1 | | | |
| **P015** | 33 | 4.8 | 10.8 | 33 | 4.8 | 10.8 | −11.6 | −10.2 | | | |
| **P019** | 36 | 2.8 | 6.0 | 36 | 2.8 | 6.0 | −14.3 | −14.2 | | | |
| **P003** | 45 | 3.8 | 5.5 | 44, 45, 46 | 3.5 | 5.7 | −14.4 | −10.6 | | | |
| **P010** | 17 | 4.2 | 4.2 | 13, 15, 16, 17 | 3.4 | 3.5 | −11.6 | −5.5 | | | |
| **P037** | 26 | 3.7 | 6.2 | 25, 26 | 3.5 | 5.8 | −0.4 | −11.8 | | | |
| **Total** | | 3.9 ± 0.7 | 5.5 (4.7, 6.2) | | 3.7 ± 0.7 | 5.4 (4.4, 6.4) | −7.5 ± 5.2 | −7.5 ± 5.2 | | | |

GT tooth involved mean PD, PT tooth involved mean PD, GT *PR* ratio, and PT *PR* ratio are presented as mean ± SD and were assessed by t-test.

GT tooth involved mean CAL, and PT tooth involved mean CAL are presented as median (IQR) and were assessed by Wilcoxon rank sum test.

**All paired GT and PT**

| | **GT** n = 28 | **PT** n = 16 | ***p*-value** |
|---|---|---|---|
| **PD** | 3.6 ± 0.7 | 3.9 ± 0.8 | 0.283 |
| **CAL** | 5.4 (4.1, 6.4) | 5.5 (4.9, 6.9) | 0.299 |

**Table 11. Local periodontal parameters at tooth sites of paired RT and ST samples**

| Subject | RT Tooth | RT Mean PD | RT Mean CAL | ST Tooth | ST Mean PD | ST Mean CAL | RT *PR* ratio | ST *PR* ratio | *P*-value PD RT−ST | *P*-value CAL RT−ST | *P*-value *PR* ratio RT-ST |
|---|---|---|---|---|---|---|---|---|---|---|---|
| **P004** | 14 | 6.7 | 9.8 | 13 | 4.7 | 9.5 | 5.0 | −1.7 | 0.730 | 0.464 | 0.654 |
| **P006** | 36 | 5.0 | 10.2 | 36 | 5.0 | 10.2 | −3.7 | −0.7 | | | |
| **P041** | 16 | 6.5 | 7.3 | 16 | 6.5 | 7.3 | 8.2 | −0.9 | | | |
| **P041** | | | | 27 | 8.8 | 9.0 | | 5.0 | | | |
| **P012** | 32 | 4.8 | 9.2 | 41 | 6.7 | 12.5 | −6.1 | −2.2 | | | |
| **P034** | 18 | 6.3 | 7.3 | 17 | 5.8 | 10.2 | 4.2 | 3.0 | | | |
| **Total** | | 6.0±0.9 | 9.5 (7.7, 9.8) | | 6.3±1.5 | 9.9 (9.1, 10.2) | 1.6 ± 5.4 | 0.4 ± 2.9 | | | |

RT tooth involved mean PD, ST tooth involved mean PD, RT *PR* ratio, and ST *PR* ratio are presented as mean ± SD and were assessed by t-test.

RT tooth involved mean CAL, and ST tooth involved mean CAL, are presented as median (IQR) and were assessed by Wilcoxon rank sum test.

**All paired RT and ST**

| | **RT** n = 15 | **ST** n = 18 | ***p*-value** |
|---|---|---|---|
| **PD** | 6.6 ± 1.4 | 6.5 ± 1.6 | 0.856 |
| **CAL** | 9.1 ± 2.2 | 9.3 ± 2.6 | 0.813 |

**Table 12. Local periodontal parameters at the GT samples from osseous defects**

**All GT samples**

| | **Total (n=59)** | **Infrabony (n=40)** | **Combined (n=19)** | ***p*-value** |
|---|---|---|---|---|
| **Mean PD (mm)** | 3.9 ± 0.9 | 3.9 ± 0.8 | 4.0 ± 0.9 | 0.524 |
| **Mean CAL (mm)** | 5.5 (4.5, 6.2) | 5.1 (4.3, 5.8) | 6.0 (5.5, 6.5) | **0.008** |
| **BOP (%)** | 50.0 (33.3, 66.7) | 50.0 (29.1, 66.7) | 50.0 (33.3, 66,7) | 0.440 |
| **PISA ($mm^2$)** | 45.8 (21.9, 104.0) | 35.3 (19.8, 57.9) | 101.0 (43.7, 151.3) | **<0.001** |

**Table 13. Local periodontal parameters in RT before and after completion of NSPT**

**All RT samples**

| **Parameter** | **Before (n=11)** | **After (n=8)** | ***p*-value** |
|---|---|---|---|
| **Mean PD (mm)** | 6.7 ± 1.6 | 6.7 ± 1.0 | 0.914 |
| **Mean CAL (mm)** | 9.7 ± 2.8 | 8.9 ± 1.6 | 0.466 |
| **BOP (%)** | 100.0 (100, 100.0) | 100.0 (100.0, 100.0) | 0.654 |
| **PISA ($mm^2$)** | 178.2 (63.7, 324.4) | 165.0 (134.6, 279.4) | 0.871 |

Teeth with delayed extraction still received non-surgical periodontal treatment (NSPT).

**Table 14. Local periodontal parameters in ST before and after completion of NSPT**

**All ST samples**

| **Parameter** | **Before (n=14)** | **After (n=6)** | ***p*-value** |
|---|---|---|---|
| **Mean PD (mm)** | 6.9 ± 1.8 | 6.5 ± 1.7 | 0.625 |
| **Mean CAL (mm)** | 9.7 ± 2.8 | 9.8 ± 2.8 | 0.900 |
| **BOP (%)** | 100.0 (95.8, 100.0) | 91.7 (70.8, 100.0) | 0.073 |
| **PISA ($mm^2$)** | 173.0 (79.3, 316.6) | 80.0 (71.7, 190.1) | 0.274 |

Teeth with delayed extraction still received non-surgical periodontal treatment (NSPT).

**Table 15. Globally significant MetaCyc ontology against the reference GT**

$q < 0.05$, passed the sensitivity analysis

**(A) Covariate-unadjusted**

| pathway | description | W | p_val | q_val |
|---|---|---|---|---|
| PWY-5529 | superpathway of bacteriochlorophyll a biosynthesis | 43.0444 | 2.58E-10 | 4.95E-08 |
| ENTBACSYN-PWY | enterobactin biosynthesis | 22.85593 | 1.95E-09 | 1.87E-07 |
| PWY-7031 | protein N-glycosylation (bacterial) | 26.57367 | 2.08E-09 | 1.87E-07 |
| PWY1G-0 | mycothiol biosynthesis | 22.5222 | 2.43E-09 | 1.87E-07 |
| PWY-7254 | TCA cycle VII (acetate-producers) | 22.08216 | 3.26E-09 | 2.09E-07 |
| CHLOROPHYLL-SYN | chlorophyllide a biosynthesis I (aerobic, light-dependent) | 30.71028 | 7.72E-09 | 3.29E-07 |
| PWY-7323 | superpathway of GDP-mannose-derived O-antigen building blocks biosynthesis | 19.55423 | 1.87E-08 | 7.2E-07 |
| PWY-5855 | ubiquinol-7 biosynthesis (prokaryotic) | 18.52906 | 3.94E-08 | 1.08E-06 |
| PWY-5856 | ubiquinol-9 biosynthesis (prokaryotic) | 18.52906 | 3.94E-08 | 1.08E-06 |
| PWY-5857 | ubiquinol-10 biosynthesis (prokaryotic) | 18.52906 | 3.94E-08 | 1.08E-06 |
| PWY-6708 | ubiquinol-8 biosynthesis (prokaryotic) | 18.52906 | 3.94E-08 | 1.08E-06 |
| UBISYN-PWY | superpathway of ubiquinol-8 biosynthesis (prokaryotic) | 17.72704 | 7.13E-08 | 1.83E-06 |
| PWY-5747 | 2-methylcitrate cycle II | 17.60887 | 7.79E-08 | 1.87E-06 |
| PWY-5509 | adenosylcobalamin biosynthesis from cobyrinate a,c-diamide I | 17.1134 | 1.13E-07 | 2.42E-06 |
| PWY-6269 | adenosylcobalamin salvage from cobinamide II | 17.12844 | 1.12E-07 | 2.42E-06 |
| COBALSYN-PWY | adenosylcobalamin salvage from cobinamide I | 16.45716 | 1.88E-07 | 3.61E-06 |
| PWY-7377 | cob(II)yrinate a,c-diamide biosynthesis I (early cobalt insertion) | 15.78683 | 3.17E-07 | 5.8E-06 |
| DENITRIFICATION-PWY | nitrate reduction I (denitrification) | 15.03547 | 5.78E-07 | 9.66E-06 |
| PWY-3781 | aerobic respiration I (cytochrome c) | 14.97478 | 6.07E-07 | 9.72E-06 |
| PWY-5505 | L-glutamate and L-glutamine biosynthesis | 14.80067 | 6.99E-07 | 1.07E-05 |
| COLANSYN-PWY | colanic acid building blocks biosynthesis | 14.39561 | 9.74E-07 | 1.44E-05 |
| PWY0-42 | 2-methylcitrate cycle I | 13.88256 | 1.49E-06 | 2.12E-05 |
| PWY-5677 | succinate fermentation to butanoate | 13.90378 | 1.56E-06 | 2.14E-05 |
| PWY-7373 | superpathway of demethylmenaquinol-6 biosynthesis II | 14.60323 | 1.94E-06 | 2.57E-05 |
| P125-PWY | superpathway of (R,R)-butanediol biosynthesis | 13.38958 | 2.26E-06 | 2.89E-05 |
| CODH-PWY | reductive acetyl coenzyme A pathway | 11.98889 | 7.97E-06 | 9.57E-05 |
| P108-PWY | pyruvate fermentation to propanoate I | 10.19057 | 3.89E-05 | 0.000426 |
| PWY-7332 | superpathway of UDP-N-acetylglucosamine-derived O-antigen building blocks biosynthesis | 10.05533 | 4.98E-05 | 0.000516 |
| PWY-1861 | formaldehyde assimilation II (RuMP Cycle) | 9.63085 | 6.59E-05 | 0.000666 |
| PWY-5304 | superpathway of sulfur oxidation (Acidianus ambivalens) | 9.487963 | 7.55E-05 | 0.000715 |

| | | | | |
|---|---|---|---|---|
| RUMP-PWY | formaldehyde oxidation I | 9.477045 | 7.63E-05 | 0.000715 |
| PWY-6728 | methylaspartate cycle | 9.274651 | 9.6E-05 | 0.000857 |
| PWY-6588 | pyruvate fermentation to acetone | 9.081616 | 0.000112 | 0.000975 |
| PWY-5920 | superpathway of heme biosynthesis from glycine | 8.942502 | 0.000128 | 0.001092 |
| PWY-7374 | 1,4-dihydroxy-6-naphthoate biosynthesis I | 8.744399 | 0.000155 | 0.001242 |
| PYRIDOXSYN-PWY | pyridoxal 5'-phosphate biosynthesis I | 8.745683 | 0.000155 | 0.001242 |
| P105-PWY | TCA cycle IV (2-oxoglutarate decarboxylase) | 8.46888 | 0.000204 | 0.001504 |
| PWY-6545 | pyrimidine deoxyribonucleotides de novo biosynthesis III | 8.480287 | 0.000201 | 0.001504 |
| SULFATE-CYS-PWY | superpathway of sulfate assimilation and cysteine biosynthesis | 8.492821 | 0.000199 | 0.001504 |
| P163-PWY | L-lysine fermentation to acetate and butanoate | 8.346169 | 0.00023 | 0.001667 |
| PWY-5177 | glutaryl-CoA degradation | 8.216151 | 0.000262 | 0.001862 |
| PWY-6383 | mono-trans, poly-cis decaprenyl phosphate biosynthesis | 7.90733 | 0.000357 | 0.002447 |
| PWY-7013 | L-1,2-propanediol degradation | 7.801514 | 0.000397 | 0.002676 |
| PWY-7211 | superpathway of pyrimidine deoxyribonucleotides de novo biosynthesis | 7.715995 | 0.000433 | 0.002868 |
| PWY-7007 | methyl ketone biosynthesis | 7.650141 | 0.000463 | 0.003014 |
| TCA-GLYOX-BYPASS | superpathway of glyoxylate bypass and TCA | 7.558954 | 0.000508 | 0.003199 |
| GLYCOL-GLYOXDEG-PWY | superpathway of glycol metabolism and degradation | 7.500695 | 0.000539 | 0.003341 |
| P164-PWY | purine nucleobases degradation I (anaerobic) | 7.202781 | 0.000733 | 0.004395 |
| PWY-5345 | superpathway of L-methionine biosynthesis (by sulfhydrylation) | 6.902196 | 0.001001 | 0.005822 |
| GLYCOLYSIS-TCA-GLYOX-BYPASS | superpathway of glycolysis, pyruvate dehydrogenase, TCA, and glyoxylate bypass | 6.833114 | 0.001076 | 0.006164 |
| PWY-6901 | superpathway of glucose and xylose degradation | 6.520962 | 0.001493 | 0.008432 |
| PWY-6608 | guanosine nucleotides degradation III | 6.405895 | 0.001687 | 0.009386 |
| SO4ASSIM-PWY | sulfate reduction I (assimilatory) | 6.209978 | 0.002078 | 0.011397 |
| PWY-5154 | L-arginine biosynthesis III (via N-acetyl-L-citrulline) | 6.189677 | 0.002123 | 0.011482 |
| PWY0-1061 | superpathway of L-alanine biosynthesis | 6.157414 | 0.002198 | 0.01172 |
| PWY-5104 | L-isoleucine biosynthesis IV | 6.140285 | 0.002238 | 0.011773 |
| PWY0-1415 | superpathway of heme biosynthesis from uroporphyrinogen-III | 6.079018 | 0.00239 | 0.012237 |
| TCA | TCA cycle I (prokaryotic) | 6.082291 | 0.002382 | 0.012237 |
| POLYAMINSYN3-PWY | superpathway of polyamine biosynthesis II | 6.004666 | 0.002589 | 0.012909 |
| PWY-7090 | UDP-2,3-diacetamido-2,3-dideoxy-α-D-mannuronate biosynthesis | 5.814138 | 0.003179 | 0.015259 |
| PWY-7184 | pyrimidine deoxyribonucleotides de novo biosynthesis I | 5.688662 | 0.003642 | 0.016851 |
| FASYN-INITIAL-PWY | superpathway of fatty acid biosynthesis initiation (E. coli) | 5.306816 | 0.005529 | 0.024404 |
| GLYOXYLATE-BYPASS | glyoxylate cycle | 5.238798 | 0.005959 | 0.026003 |
| P461-PWY | hexitol fermentation to lactate, formate, ethanol and acetate | 5.081798 | 0.007088 | 0.029911 |

| | | | | |
|---|---|---|---|---|
| PWY-6396 | superpathway of 2,3-butanediol biosynthesis | 5.015686 | 0.007628 | 0.031159 |
| PWY-5989 | stearate biosynthesis II (bacteria and plants) | 4.773532 | 0.009991 | 0.038753 |
| PWY0-845 | superpathway of pyridoxal 5'-phosphate biosynthesis and salvage | 4.6309 | 0.011724 | 0.044574 |

**(B) Covariate-adjusted**

| pathway | description | W | p_val | q_val |
|---|---|---|---|---|
| PWY-5529 | superpathway of bacteriochlorophyll a biosynthesis | 72.44679 | 2.93E-11 | 5.63E-09 |
| CHLOROPHYLL-SYN | chlorophyllide a biosynthesis I (aerobic, light-dependent) | 62.33683 | 6.74E-11 | 8.63E-09 |
| PWY-7031 | protein N-glycosylation (bacterial) | 30.26919 | 1.56E-09 | 1.2E-07 |
| PWY-7323 | superpathway of GDP-mannose-derived O-antigen building blocks biosynthesis | 23.83267 | 2.36E-09 | 1.51E-07 |
| PWY-5509 | adenosylcobalamin biosynthesis from cobyrinate a,c-diamide I | 23.06106 | 3.77E-09 | 1.86E-07 |
| PWY-6269 | adenosylcobalamin salvage from cobinamide II | 23.02022 | 3.87E-09 | 1.86E-07 |
| COBALSYN-PWY | adenosylcobalamin salvage from cobinamide I | 22.72006 | 4.65E-09 | 1.99E-07 |
| PWY-5505 | L-glutamate and L-glutamine biosynthesis | 21.77819 | 8.39E-09 | 3.22E-07 |
| PWY1G-0 | mycothiol biosynthesis | 20.97022 | 1.41E-08 | 4.91E-07 |
| COLANSYN-PWY | colanic acid building blocks biosynthesis | 18.26982 | 8.62E-08 | 2.76E-06 |
| ENTBACSYN-PWY | enterobactin biosynthesis | 17.61764 | 1.36E-07 | 4.02E-06 |
| PWY-7373 | superpathway of demethylmenaquinol-6 biosynthesis II | 19.59976 | 1.53E-07 | 4.19E-06 |
| PWY-5677 | succinate fermentation to butanoate | 17.06087 | 2.24E-07 | 5.73E-06 |
| PWY-7254 | TCA cycle VII (acetate-producers) | 16.50922 | 3.03E-07 | 6.84E-06 |
| PWY-7377 | cob(II)yrinate a,c-diamide biosynthesis I (early cobalt insertion) | 16.50974 | 3.03E-07 | 6.84E-06 |
| CODH-PWY | reductive acetyl coenzyme A pathway | 16.55812 | 3.21E-07 | 6.85E-06 |
| P108-PWY | pyruvate fermentation to propanoate I | 15.38072 | 7.01E-07 | 1.4E-05 |
| PYRIDOXSYN-PWY | pyridoxal 5'-phosphate biosynthesis I | 14.97132 | 9.58E-07 | 1.67E-05 |
| PWY-5989 | stearate biosynthesis II (bacteria and plants) | 13.95682 | 2.11E-06 | 3.38E-05 |
| FASYN-INITIAL-PWY | superpathway of fatty acid biosynthesis initiation (E. coli) | 13.86682 | 2.27E-06 | 3.48E-05 |
| PWY-6282 | palmitoleate biosynthesis I (from (5Z)-dodec-5-enoate) | 13.73235 | 2.52E-06 | 3.73E-05 |
| P163-PWY | L-lysine fermentation to acetate and butanoate | 13.27286 | 3.65E-06 | 5E-05 |
| PWY0-42 | 2-methylcitrate cycle I | 13.21617 | 3.82E-06 | 5.06E-05 |
| PWY-6588 | pyruvate fermentation to acetone | 13.08595 | 4.24E-06 | 5.43E-05 |
| PWYG-321 | mycolate biosynthesis | 12.76424 | 5.52E-06 | 6.84E-05 |
| PWY-7664 | oleate biosynthesis IV (anaerobic) | 12.58674 | 6.39E-06 | 7.44E-05 |
| DENITRIFICATION-PWY | nitrate reduction I (denitrification) | 12.53221 | 6.69E-06 | 7.55E-05 |
| PWY-5177 | glutaryl-CoA degradation | 11.8713 | 1.16E-05 | 0.000121 |
| PWY-6383 | mono-trans, poly-cis decaprenyl phosphate biosynthesis | 11.83961 | 1.19E-05 | 0.000121 |

| | | | | |
|---|---|---|---|---|
| P164-PWY | purine nucleobases degradation I (anaerobic) | 11.5516 | 1.53E-05 | 0.00015 |
| PWY-5855 | ubiquinol-7 biosynthesis (prokaryotic) | 11.41551 | 1.72E-05 | 0.000153 |
| PWY-5856 | ubiquinol-9 biosynthesis (prokaryotic) | 11.41551 | 1.72E-05 | 0.000153 |
| PWY-5857 | ubiquinol-10 biosynthesis (prokaryotic) | 11.41551 | 1.72E-05 | 0.000153 |
| PWY-6708 | ubiquinol-8 biosynthesis (prokaryotic) | 11.41551 | 1.72E-05 | 0.000153 |
| PWY-7332 | superpathway of UDP-N-acetylglucosamine-derived O-antigen building blocks biosynthesis | 11.49413 | 1.91E-05 | 0.000167 |
| PWY-5747 | 2-methylcitrate cycle II | 11.04748 | 2.36E-05 | 0.000197 |
| PWY-6608 | guanosine nucleotides degradation III | 10.91788 | 2.64E-05 | 0.000216 |
| PWY-5971 | palmitate biosynthesis II (bacteria and plants) | 10.68231 | 3.25E-05 | 0.00026 |
| UBISYN-PWY | superpathway of ubiquinol-8 biosynthesis (prokaryotic) | 10.64803 | 3.35E-05 | 0.000262 |
| P125-PWY | superpathway of (R,R)-butanediol biosynthesis | 10.50166 | 3.81E-05 | 0.000292 |
| PWY0-845 | superpathway of pyridoxal 5'-phosphate biosynthesis and salvage | 10.05339 | 5.68E-05 | 0.000428 |
| PWY-6545 | pyrimidine deoxyribonucleotides de novo biosynthesis III | 9.399951 | 0.000103 | 0.000747 |
| PWY-5304 | superpathway of sulfur oxidation (Acidianus ambivalens) | 9.309433 | 0.000112 | 0.000783 |
| PWY-5920 | superpathway of heme biosynthesis from glycine | 9.311999 | 0.000112 | 0.000783 |
| PWY0-862 | (5Z)-dodec-5-enoate biosynthesis | 9.006122 | 0.000149 | 0.000951 |
| PWY-7007 | methyl ketone biosynthesis | 8.923115 | 0.000161 | 0.000998 |
| PWY-3781 | aerobic respiration I (cytochrome c) | 8.579309 | 0.000222 | 0.001312 |
| PWY-6901 | superpathway of glucose and xylose degradation | 8.448345 | 0.000252 | 0.001464 |
| PWY-1861 | formaldehyde assimilation II (RuMP Cycle) | 7.947545 | 0.000407 | 0.002267 |
| RUMP-PWY | formaldehyde oxidation I | 7.787313 | 0.000476 | 0.002612 |
| PWY-5104 | L-isoleucine biosynthesis IV | 7.533472 | 0.000611 | 0.003214 |
| TCA-GLYOX-BYPASS | superpathway of glyoxylate bypass and TCA | 7.540409 | 0.000607 | 0.003214 |
| PWY-6891 | thiazole biosynthesis II (Bacillus) | 7.305324 | 0.000766 | 0.003921 |
| PWY-7090 | UDP-2,3-diacetamido-2,3-dideoxy-α-D-mannuronate biosynthesis | 7.250735 | 0.000809 | 0.004086 |
| METHANOGENESIS-PWY | methanogenesis from H2 and CO2 | 9.909519 | 0.000884 | 0.004351 |
| PWY-6895 | superpathway of thiamin diphosphate biosynthesis II | 6.849088 | 0.00121 | 0.005737 |
| PWY-6892 | thiazole biosynthesis I (E. coli) | 6.826137 | 0.001239 | 0.005801 |
| PWY-7374 | 1,4-dihydroxy-6-naphthoate biosynthesis I | 6.778246 | 0.0013 | 0.005944 |
| GLYCOLYSIS-TCA-GLYOX-BYPASS | superpathway of glycolysis, pyruvate dehydrogenase, TCA, and glyoxylate bypass | 6.520534 | 0.001691 | 0.007378 |
| P162-PWY | L-glutamate degradation V (via hydroxyglutarate) | 6.531514 | 0.001672 | 0.007378 |
| P105-PWY | TCA cycle IV (2-oxoglutarate decarboxylase) | 6.346031 | 0.002023 | 0.00863 |
| PWY-5154 | L-arginine biosynthesis III (via N-acetyl-L-citrulline) | 6.169555 | 0.002428 | 0.010023 |

| | | | | |
|---|---|---|---|---|
| ASPASN-PWY | superpathway of L-aspartate and L-asparagine biosynthesis | 6.007381 | 0.002874 | 0.01126 |
| PWY-6703 | preQ0 biosynthesis | 5.958655 | 0.003024 | 0.011728 |
| NAGLIPASYN-PWY | lipid IVA biosynthesis | 5.936959 | 0.003093 | 0.011878 |
| PWY0-1241 | ADP-L-glycero-β-D-manno-heptose biosynthesis | 5.879918 | 0.003284 | 0.012362 |
| GLYOXYLATE-BYPASS | glyoxylate cycle | 5.612892 | 0.00435 | 0.015809 |
| THISYN-PWY | superpathway of thiamin diphosphate biosynthesis I | 5.60112 | 0.004405 | 0.015809 |
| PWY-6700 | queuosine biosynthesis | 5.335741 | 0.005843 | 0.020397 |
| PWY-6467 | Kdo transfer to lipid IVA III (Chlamydia) | 5.311734 | 0.005995 | 0.020739 |
| FASYN-ELONG-PWY | fatty acid elongation -- saturated | 4.984528 | 0.008528 | 0.028475 |
| PWY-1269 | CMP-3-deoxy-D-manno-octulosonate biosynthesis I | 4.892568 | 0.009423 | 0.031059 |
| SULFATE-CYS-PWY | superpathway of sulfate assimilation and cysteine biosynthesis | 4.747037 | 0.011042 | 0.035631 |
| PWY-7211 | superpathway of pyrimidine deoxyribonucleotides de novo biosynthesis | 4.646819 | 0.012322 | 0.039431 |
| PWY-5941 | glycogen degradation II (eukaryotic) | 5.610863 | 0.012564 | 0.039873 |
| GLYCOGENSYNTH-PWY | glycogen biosynthesis I (from ADP-D-Glucose) | 4.620826 | 0.012678 | 0.039906 |
| GLYCOL-GLYOXDEG-PWY | superpathway of glycol metabolism and degradation | 4.52032 | 0.01416 | 0.04385 |
| PWY-6507 | 4-deoxy-L-threo-hex-4-enopyranuronate degradation | 4.484835 | 0.014724 | 0.045233 |
| PWY0-1415 | superpathway of heme biosynthesis from uroporphyrinogen-III | 4.448615 | 0.015325 | 0.046704 |
| TCA | TCA cycle I (prokaryotic) | 4.414682 | 0.01591 | 0.047721 |

MetaCyc terms positively associated with Maaslin2-selected periodontopathogen genera are colored in red, while those terms positively associated with commensal genera are colored in green (Fig.3B).

**Table 16. Multiple pairwise comparisons of MetaCyc ontology against the reference GT using Dunnett's type of test**

**(A) Covariate-unadjusted**

| pathway | description | lfc_PT | lfc_RT | lfc_ST | se_PT | se_RT | se_ST | p_PT | p_RT | p_ST | q_PT | q_RT | q_ST | passed_ss_PT | passed_ss_RT | passed_ss_ST |
|---|---|---|---|---|---|---|---|---|---|---|---|---|---|---|---|---|
| PWY-6143 | CMP-pseudaminate biosynthesis | -2.07 | 2.08 | -2.18 | 0.63 | 0.55 | 0.50 | 0.0028 | 0.0008 | 0.0001 | 0.1296 | 0.0375 | 0.0067 | FALSE | FALSE | TRUE |
| PWY-7323 | superpathway of GDP-mannose-derived O-antigen building blocks biosynthesis | -0.35 | 1.13 | 0.39 | 0.34 | 0.31 | 0.32 | 0.3040 | 0.0006 | 0.2259 | 1.0000 | 0.0294 | 1.0000 | TRUE | TRUE | TRUE |
| COLANSYN-PWY | colanic acid building blocks biosynthesis | -0.24 | 1.10 | 0.34 | 0.28 | 0.33 | 0.29 | 0.4064 | 0.0017 | 0.2451 | 1.0000 | 0.0828 | 1.0000 | TRUE | TRUE | TRUE |
| PWY-7377 | cob(II)yrinate a,c-diamide biosynthesis I (early cobalt insertion) | -0.24 | 0.64 | 0.81 | 0.33 | 0.35 | 0.25 | 0.4638 | 0.0704 | 0.0021 | 1.0000 | 1.0000 | 0.0990 | TRUE | FALSE | TRUE |
| CHLOROPHYLL-SYN | chlorophyllide a biosynthesis I (aerobic, light-dependent) | 1.97 | 0.35 | -0.05 | 0.43 | 0.82 | 0.59 | 0.0001 | 0.6690 | 0.9292 | 0.0040 | 1.0000 | 1.0000 | FALSE | FALSE | FALSE |
| PWY-7373 | superpathway of demethylmenaquinol-6 biosynthesis II | -1.32 | 0.22 | -1.51 | 0.51 | 0.88 | 0.46 | 0.0127 | 0.8076 | 0.0019 | 0.5956 | 1.0000 | 0.0910 | FALSE | FALSE | FALSE |
| PWY-5529 | superpathway of bacteriochlorophyll a biosynthesis | 2.11 | -0.02 | 0.08 | 0.42 | 0.59 | 0.54 | 0.0000 | 0.9728 | 0.8816 | 0.0012 | 1.0000 | 1.0000 | FALSE | FALSE | FALSE |
| PWY-7031 | protein N-glycosylation (bacterial) | -1.82 | -0.23 | -2.25 | 0.56 | 0.95 | 0.47 | 0.0021 | 0.8086 | 0.0000 | 0.1000 | 1.0000 | 0.0010 | TRUE | FALSE | FALSE |
| PWY-6731 | starch degradation III | 1.84 | -0.50 | -0.20 | 0.40 | 0.59 | 0.43 | 0.0001 | 0.4060 | 0.6485 | 0.0061 | 1.0000 | 1.0000 | FALSE | TRUE | TRUE |
| UBISYN-PWY | superpathway of ubiquinol-8 biosynthesis (prokaryotic) | 0.00 | -1.25 | -0.72 | 0.44 | 0.32 | 0.36 | 0.9974 | 0.0003 | 0.0521 | 1.0000 | 0.0133 | 1.0000 | TRUE | TRUE | FALSE |
| PWY-5855 | ubiquinol-7 biosynthesis (prokaryotic) | 0.01 | -1.28 | -0.73 | 0.44 | 0.32 | 0.37 | 0.9892 | 0.0002 | 0.0513 | 1.0000 | 0.0102 | 1.0000 | TRUE | TRUE | FALSE |
| PWY-5856 | ubiquinol-9 biosynthesis (prokaryotic) | 0.01 | -1.28 | -0.73 | 0.44 | 0.32 | 0.37 | 0.9892 | 0.0002 | 0.0513 | 1.0000 | 0.0102 | 1.0000 | TRUE | TRUE | FALSE |
| PWY-5857 | ubiquinol-10 biosynthesis (prokaryotic) | 0.01 | -1.28 | -0.73 | 0.44 | 0.32 | 0.37 | 0.9892 | 0.0002 | 0.0513 | 1.0000 | 0.0102 | 1.0000 | TRUE | TRUE | FALSE |
| PWY-6708 | ubiquinol-8 biosynthesis (prokaryotic) | 0.01 | -1.28 | -0.73 | 0.44 | 0.32 | 0.37 | 0.9892 | 0.0002 | 0.0513 | 1.0000 | 0.0102 | 1.0000 | TRUE | TRUE | FALSE |

| | | | | | | | | | | | | | | | | |
|---|---|---|---|---|---|---|---|---|---|---|---|---|---|---|---|---|
| PWY-7254 | TCA cycle VII (acetate-producers) | 0.29 | -1.30 | -0.91 | 0.37 | 0.35 | 0.37 | 0.4388 | 0.0005 | 0.0163 | 1.0000 | 0.0219 | 0.7672 | TRUE | TRUE | FALSE |
| ENTBACSYN-PWY | enterobactin biosynthesis | 0.28 | -1.36 | -1.03 | 0.39 | 0.36 | 0.39 | 0.4790 | 0.0004 | 0.0119 | 1.0000 | 0.0207 | 0.5584 | TRUE | TRUE | FALSE |
| P125-PWY | superpathway of (R,R)-butanediol biosynthesis | 0.29 | -1.39 | -0.78 | 0.42 | 0.46 | 0.45 | 0.4963 | 0.0039 | 0.0874 | 1.0000 | 0.1884 | 1.0000 | TRUE | TRUE | FALSE |
| DENITRIFICATION-PWY | nitrate reduction I (denitrification) | -0.16 | -1.42 | -1.33 | 0.58 | 0.41 | 0.52 | 0.7805 | 0.0011 | 0.0136 | 1.0000 | 0.0542 | 0.6382 | TRUE | TRUE | FALSE |
| PWY-5747 | 2-methylcitrate cycle II | 0.01 | -1.44 | -0.93 | 0.51 | 0.38 | 0.45 | 0.9834 | 0.0004 | 0.0452 | 1.0000 | 0.0177 | 1.0000 | TRUE | TRUE | FALSE |
| PWY1G-0 | mycothiol biosynthesis | 0.27 | -1.54 | -1.23 | 0.49 | 0.41 | 0.45 | 0.5859 | 0.0004 | 0.0079 | 1.0000 | 0.0182 | 0.3736 | TRUE | TRUE | FALSE |
| PWY-1361 | benzoyl-CoA degradation I (aerobic) | 0.10 | -1.58 | -1.16 | 0.38 | 0.47 | 0.43 | 0.7900 | 0.0036 | 0.0151 | 1.0000 | 0.1749 | 0.7106 | TRUE | FALSE | TRUE |

## (B) Covariate-adjusted

| pathway | description | lfc_PT | lfc_RT | lfc_ST | se_PT | se_RT | se_ST | p_PT | p_RT | p_ST | q_PT | q_RT | q_ST | passed_ss_PT | passed_ss_RT | passed_ss_ST |
|---|---|---|---|---|---|---|---|---|---|---|---|---|---|---|---|---|
| PWY-5677 | succinate fermentation to butanoate | -0.59 | 1.94 | -0.35 | 0.47 | 0.61 | 0.54 | 0.2073 | 0.0027 | 0.5209 | 1.0000 | 0.0519 | 1.0000 | TRUE | TRUE | TRUE |
| PWY-6143 | CMP-pseudaminate biosynthesis | -2.89 | 1.59 | -3.08 | 0.56 | 0.49 | 0.62 | 0.0000 | 0.0039 | 0.0001 | 0.0007 | 0.0673 | 0.0010 | FALSE | FALSE | TRUE |
| PYRIDOXSYN-PWY | pyridoxal 5'-phosphate biosynthesis I | -0.21 | 1.30 | -0.14 | 0.29 | 0.41 | 0.36 | 0.4783 | 0.0026 | 0.7063 | 1.0000 | 0.0493 | 1.0000 | TRUE | TRUE | TRUE |
| PWY-7323 | superpathway of GDP-mannose-derived O-antigen building blocks biosynthesis | -0.45 | 1.27 | 0.12 | 0.29 | 0.33 | 0.34 | 0.1192 | 0.0003 | 0.7191 | 1.0000 | 0.0062 | 1.0000 | FALSE | TRUE | TRUE |
| PWY-1861 | formaldehyde assimilation II (RuMP Cycle) | 0.67 | 1.22 | 1.02 | 0.55 | 0.44 | 0.53 | 0.2316 | 0.0076 | 0.0599 | 1.0000 | 0.1460 | 1.0000 | TRUE | TRUE | FALSE |
| RUMP-PWY | formaldehyde oxidation I | 0.67 | 1.21 | 1.01 | 0.55 | 0.44 | 0.52 | 0.2254 | 0.0082 | 0.0599 | 1.0000 | 0.1580 | 1.0000 | TRUE | TRUE | FALSE |
| COLANSYN-PWY | colanic acid building blocks biosynthesis | -0.30 | 1.20 | 0.08 | 0.24 | 0.33 | 0.30 | 0.2258 | 0.0007 | 0.7995 | 1.0000 | 0.0130 | 1.0000 | TRUE | TRUE | TRUE |
| CODH-PWY | reductive acetyl coenzyme A pathway | -0.56 | 1.13 | 0.18 | 0.48 | 0.35 | 0.43 | 0.2434 | 0.0024 | 0.6765 | 1.0000 | 0.0456 | 1.0000 | TRUE | TRUE | TRUE |
| CHLOROPHYLL-SYN | chlorophyllide a biosynthesis I (aerobic, light-dependent) | 2.89 | 1.04 | 0.48 | 0.41 | 0.70 | 0.60 | 0.0000 | 0.1498 | 0.4320 | 0.0000 | 1.0000 | 1.0000 | FALSE | FALSE | TRUE |

| | | | | | | | | | | | | | | | | |
|---|---|---|---|---|---|---|---|---|---|---|---|---|---|---|---|---|
| PWY-6269 | adenosylcobalamin salvage from cobinamide II | -0.56 | 1.00 | 0.38 | 0.28 | 0.29 | 0.27 | 0.0477 | 0.0012 | 0.1664 | 0.8683 | 0.0239 | 1.0000 | FALSE | TRUE | FALSE |
| PWY-5509 | adenosylcobalamin biosynthesis from cobyrinate a,c-diamide I | -0.56 | 1.00 | 0.38 | 0.27 | 0.29 | 0.27 | 0.0482 | 0.0012 | 0.1661 | 0.8773 | 0.0233 | 1.0000 | FALSE | TRUE | FALSE |
| P108-PWY | pyruvate fermentation to propanoate I | -0.37 | 0.98 | -0.14 | 0.33 | 0.33 | 0.30 | 0.2713 | 0.0046 | 0.6364 | 1.0000 | 0.0878 | 1.0000 | TRUE | TRUE | TRUE |
| COBALSYN-PWY | adenosylcobalamin salvage from cobinamide I | -0.57 | 0.93 | 0.37 | 0.27 | 0.28 | 0.27 | 0.0417 | 0.0017 | 0.1655 | 0.7598 | 0.0330 | 1.0000 | FALSE | TRUE | FALSE |
| P164-PWY | purine nucleobases degradation I (anaerobic) | -0.36 | 0.87 | 0.26 | 0.34 | 0.31 | 0.32 | 0.2878 | 0.0083 | 0.4200 | 1.0000 | 0.1600 | 1.0000 | TRUE | TRUE | TRUE |
| PWY-5505 | L-glutamate and L-glutamine biosynthesis | -0.73 | 0.85 | 0.37 | 0.29 | 0.29 | 0.27 | 0.0140 | 0.0046 | 0.1776 | 0.2551 | 0.0890 | 1.0000 | FALSE | TRUE | FALSE |
| PWY-6608 | guanosine nucleotides degradation III | -0.35 | 0.79 | 0.19 | 0.35 | 0.29 | 0.31 | 0.3199 | 0.0091 | 0.5383 | 1.0000 | 0.1741 | 1.0000 | TRUE | TRUE | TRUE |
| PWY-7377 | cob(II)yrinate a,c-diamide biosynthesis I (early cobalt insertion) | -0.40 | 0.72 | 0.49 | 0.29 | 0.25 | 0.26 | 0.1654 | 0.0052 | 0.0646 | 1.0000 | 0.1007 | 1.0000 | FALSE | TRUE | FALSE |
| PWY-5529 | superpathway of bacteriochlorophyll a biosynthesis | 2.96 | 0.50 | 0.55 | 0.40 | 0.46 | 0.54 | 0.0000 | 0.2849 | 0.3197 | 0.0000 | 1.0000 | 1.0000 | FALSE | FALSE | TRUE |
| PWY-7527 | L-methionine salvage cycle III | 2.00 | 0.38 | 0.24 | 0.43 | 0.54 | 0.51 | 0.0002 | 0.4971 | 0.6380 | 0.0040 | 1.0000 | 1.0000 | FALSE | TRUE | TRUE |
| PWY-4361 | S-methyl-5-thio-α-D-ribose 1-phosphate degradation | 2.04 | 0.36 | 0.25 | 0.43 | 0.55 | 0.51 | 0.0002 | 0.5160 | 0.6372 | 0.0035 | 1.0000 | 1.0000 | FALSE | TRUE | TRUE |
| PWY-6071 | superpathway of phenylethylamine degradation | 1.59 | 0.26 | 0.47 | 0.42 | 0.39 | 0.45 | 0.0009 | 0.5078 | 0.3071 | 0.0174 | 1.0000 | 1.0000 | FALSE | TRUE | TRUE |
| PWY-7373 | superpathway of demethylmenaquinol-6 biosynthesis II | -1.66 | 0.18 | -1.67 | 0.48 | 0.81 | 0.47 | 0.0014 | 0.8295 | 0.0011 | 0.0252 | 1.0000 | 0.0209 | TRUE | FALSE | FALSE |
| PWY-7664 | oleate biosynthesis IV (anaerobic) | -0.25 | 0.00 | -0.80 | 0.33 | 0.33 | 0.24 | 0.4592 | 0.9944 | 0.0015 | 1.0000 | 1.0000 | 0.0279 | TRUE | TRUE | TRUE |
| PWY0-862 | (5Z)-dodec-5-enoate biosynthesis | -0.04 | 0.00 | -0.62 | 0.30 | 0.29 | 0.22 | 0.8886 | 0.9995 | 0.0061 | 1.0000 | 1.0000 | 0.1165 | TRUE | TRUE | TRUE |
| PWY-5971 | palmitate biosynthesis II (bacteria and plants) | -0.25 | -0.01 | -0.73 | 0.32 | 0.31 | 0.23 | 0.4434 | 0.9768 | 0.0029 | 1.0000 | 1.0000 | 0.0554 | TRUE | TRUE | TRUE |

| | | | | | | | | | | | | | | | | |
|---|---|---|---|---|---|---|---|---|---|---|---|---|---|---|---|---|
| PWYG-321 | mycolate biosynthesis | -0.27 | -0.03 | -0.85 | 0.34 | 0.34 | 0.25 | 0.4325 | 0.9387 | 0.0013 | 1.0000 | 1.0000 | 0.0240 | TRUE | TRUE | TRUE |
| PWY-6282 | palmitoleate biosynthesis I (from (5Z)-dodec-5-enoate) | -0.29 | -0.03 | -0.91 | 0.36 | 0.36 | 0.26 | 0.4169 | 0.9384 | 0.0009 | 1.0000 | 1.0000 | 0.0171 | TRUE | TRUE | TRUE |
| PWY-5989 | stearate biosynthesis II (bacteria and plants) | -0.31 | -0.05 | -0.93 | 0.36 | 0.36 | 0.26 | 0.3896 | 0.8865 | 0.0008 | 1.0000 | 1.0000 | 0.0148 | TRUE | TRUE | TRUE |
| PWY-7031 | protein N-glycosylation (bacterial) | -2.30 | -0.06 | -2.32 | 0.51 | 0.87 | 0.52 | 0.0001 | 0.9448 | 0.0001 | 0.0015 | 1.0000 | 0.0015 | TRUE | FALSE | FALSE |
| PWY-6107 | chlorosalicylate degradation | -1.43 | -0.09 | 0.49 | 0.40 | 0.38 | 0.50 | 0.0038 | 0.8123 | 0.3471 | 0.0722 | 1.0000 | 1.0000 | FALSE | TRUE | TRUE |
| PWY-6165 | chorismate biosynthesis II (archaea) | 1.41 | -0.09 | 0.05 | 0.40 | 0.38 | 0.49 | 0.0065 | 0.8153 | 0.9139 | 0.1247 | 1.0000 | 1.0000 | FALSE | TRUE | TRUE |
| FASYN-INITIAL-PWY | superpathway of fatty acid biosynthesis initiation (E. coli) | -0.30 | -0.17 | -1.00 | 0.39 | 0.37 | 0.28 | 0.4458 | 0.6514 | 0.0007 | 1.0000 | 1.0000 | 0.0129 | TRUE | TRUE | TRUE |
| LIPASYN-PWY | phospholipases | -1.90 | -0.19 | -0.33 | 0.43 | 0.49 | 0.53 | 0.0010 | 0.7050 | 0.5475 | 0.0194 | 1.0000 | 1.0000 | FALSE | TRUE | TRUE |
| PWY-6731 | starch degradation III | 2.62 | -0.26 | 0.22 | 0.38 | 0.37 | 0.48 | 0.0000 | 0.4893 | 0.6544 | 0.0001 | 1.0000 | 1.0000 | FALSE | TRUE | TRUE |
| PWY-7391 | isoprene biosynthesis II (engineered) | -1.25 | -0.43 | -0.05 | 0.44 | 0.46 | 0.44 | 0.0086 | 0.3527 | 0.9115 | 0.1652 | 1.0000 | 1.0000 | FALSE | TRUE | TRUE |
| PWY-5920 | superpathway of heme biosynthesis from glycine | -0.07 | -0.74 | -0.13 | 0.31 | 0.26 | 0.32 | 0.8209 | 0.0059 | 0.6806 | 1.0000 | 0.1136 | 1.0000 | TRUE | TRUE | TRUE |
| TCA-GLYOX-BYPASS | superpathway of glyoxylate bypass and TCA | -0.22 | -0.74 | -0.30 | 0.28 | 0.27 | 0.33 | 0.4293 | 0.0085 | 0.3712 | 1.0000 | 0.1631 | 1.0000 | TRUE | TRUE | TRUE |
| PWY-6992 | 1,5-anhydrofructose degradation | 1.91 | -0.84 | 0.78 | 0.42 | 0.45 | 0.51 | 0.0010 | 0.0907 | 0.1605 | 0.0194 | 1.0000 | 1.0000 | FALSE | TRUE | TRUE |
| PWY-7007 | methyl ketone biosynthesis | -0.14 | -1.00 | -0.38 | 0.39 | 0.35 | 0.47 | 0.7142 | 0.0060 | 0.4198 | 1.0000 | 0.1155 | 1.0000 | TRUE | TRUE | TRUE |
| UBISYN-PWY | superpathway of ubiquinol-8 biosynthesis (prokaryotic) | 0.02 | -1.02 | -0.58 | 0.43 | 0.33 | 0.41 | 0.9535 | 0.0032 | 0.1617 | 1.0000 | 0.0606 | 1.0000 | TRUE | TRUE | FALSE |
| PWY-5855 | ubiquinol-7 biosynthesis (prokaryotic) | 0.03 | -1.05 | -0.57 | 0.42 | 0.33 | 0.41 | 0.9505 | 0.0023 | 0.1718 | 1.0000 | 0.0439 | 1.0000 | TRUE | TRUE | FALSE |
| PWY-5856 | ubiquinol-9 biosynthesis (prokaryotic) | 0.03 | -1.05 | -0.57 | 0.42 | 0.33 | 0.41 | 0.9505 | 0.0023 | 0.1718 | 1.0000 | 0.0439 | 1.0000 | TRUE | TRUE | FALSE |
| PWY-5857 | ubiquinol-10 biosynthesis (prokaryotic) | 0.03 | -1.05 | -0.57 | 0.42 | 0.33 | 0.41 | 0.9505 | 0.0023 | 0.1718 | 1.0000 | 0.0439 | 1.0000 | TRUE | TRUE | FALSE |

| | | | | | | | | | | | | | | | | |
|---|---|---|---|---|---|---|---|---|---|---|---|---|---|---|---|---|
| PWY-6708 | ubiquinol-8 biosynthesis (prokaryotic) | 0.03 | -1.05 | -0.57 | 0.42 | 0.33 | 0.41 | 0.9505 | 0.0023 | 0.1718 | 1.0000 | 0.0439 | 1.0000 | TRUE | TRUE | FALSE |
| PWY-6383 | mono-trans, poly-cis decaprenyl phosphate biosynthesis | -0.13 | -1.16 | -0.39 | 0.43 | 0.35 | 0.36 | 0.7663 | 0.0016 | 0.2784 | 1.0000 | 0.0305 | 1.0000 | TRUE | TRUE | TRUE |
| PWY-5747 | 2-methylcitrate cycle II | 0.02 | -1.17 | -0.79 | 0.51 | 0.37 | 0.51 | 0.9641 | 0.0029 | 0.1307 | 1.0000 | 0.0559 | 1.0000 | TRUE | TRUE | FALSE |
| PWY-7254 | TCA cycle VII (acetate-producers) | 0.24 | -1.18 | -0.79 | 0.39 | 0.34 | 0.38 | 0.5478 | 0.0010 | 0.0405 | 1.0000 | 0.0187 | 0.7370 | TRUE | TRUE | FALSE |
| PWY0-42 | 2-methylcitrate cycle I | -0.42 | -1.23 | -0.81 | 0.46 | 0.35 | 0.45 | 0.3678 | 0.0008 | 0.0761 | 1.0000 | 0.0163 | 1.0000 | TRUE | TRUE | FALSE |
| DENITRIFICATION-PWY | nitrate reduction I (denitrification) | -0.11 | -1.25 | -1.42 | 0.60 | 0.43 | 0.53 | 0.8569 | 0.0057 | 0.0097 | 1.0000 | 0.1101 | 0.1766 | TRUE | TRUE | TRUE |
| ENTBACSYN-PWY | enterobactin biosynthesis | 0.12 | -1.31 | -0.85 | 0.39 | 0.35 | 0.40 | 0.7513 | 0.0005 | 0.0380 | 1.0000 | 0.0102 | 0.6917 | TRUE | TRUE | FALSE |
| P125-PWY | superpathway of (R,R)-butanediol biosynthesis | 0.16 | -1.33 | -0.71 | 0.42 | 0.45 | 0.49 | 0.7014 | 0.0051 | 0.1504 | 1.0000 | 0.0971 | 1.0000 | TRUE | TRUE | FALSE |
| PWY1G-0 | mycothiol biosynthesis | 0.13 | -1.59 | -1.08 | 0.51 | 0.39 | 0.46 | 0.7912 | 0.0002 | 0.0239 | 1.0000 | 0.0038 | 0.4358 | TRUE | TRUE | FALSE |
| PWY-1361 | benzoyl-CoA degradation I (aerobic) | 0.35 | -1.71 | -1.32 | 0.38 | 0.37 | 0.52 | 0.3689 | 0.0008 | 0.0283 | 1.0000 | 0.0144 | 0.5143 | FALSE | FALSE | TRUE |
| PWY-7255 | ergothioneine biosynthesis I (bacteria) | -0.12 | -1.86 | -0.36 | 0.45 | 0.51 | 0.46 | 0.7905 | 0.0023 | 0.4441 | 1.0000 | 0.0446 | 1.0000 | TRUE | FALSE | TRUE |

**passed_ss:** passed the sensitivity analysis for pseudo-count addition

**Table 17. Spearman's correlation between Maaslin2-identified taxa and ANCOM-BC2-selected PICRUSt2-predicted MetaCyc ontology**

17.1

| Genus | p2PW | description | Spearman_rho | p_val | p_adj |
|---|---|---|---|---|---|
| Campylobacter | PWY-7031 | protein N-glycosylation (bacterial) | 0.87 | 8.06E-19 | 1.40E-15 |
| Rothia | PWY1G-0 | mycothiol biosynthesis | 0.84 | 9.68E-17 | 8.44E-14 |
| Rothia | ENTBACSYN-PWY | enterobactin biosynthesis | 0.82 | 1.51E-15 | 8.77E-13 |
| Tannerella | PWY-5677 | succinate fermentation to butanoate | 0.80 | 3.93E-14 | 1.71E-11 |
| Parvimonas | CODH-PWY | reductive acetyl coenzyme A pathway | 0.77 | 1.00E-12 | 3.49E-10 |
| Campylobacter | PWY-7373 | superpathway of demethylmenaquinol-6 biosynthesis II | 0.76 | 2.39E-12 | 6.95E-10 |
| Neisseria | P125-PWY | superpathway of (R,R)-butanediol biosynthesis | 0.74 | 2.13E-11 | 5.30E-09 |
| Neisseria | PWY-5747 | 2-methylcitrate cycle II | 0.73 | 6.11E-11 | 1.28E-08 |
| Tannerella | PWY-7332 | superpathway of UDP-N-acetylglucosamine-derived O-antigen building blocks biosynthesis | 0.73 | 6.61E-11 | 1.28E-08 |
| Rothia | PWY-5509 | adenosylcobalamin biosynthesis from cobyrinate a,c-diamide I | -0.72 | 1.26E-10 | 2.19E-08 |
| Rothia | PWY-6269 | adenosylcobalamin salvage from cobinamide II | -0.72 | 1.41E-10 | 2.23E-08 |
| Rothia | P125-PWY | superpathway of (R,R)-butanediol biosynthesis | 0.72 | 1.67E-10 | 2.42E-08 |
| Rothia | PWY-7332 | superpathway of UDP-N-acetylglucosamine-derived O-antigen building blocks biosynthesis | -0.71 | 3.92E-10 | 5.26E-08 |
| Rothia | COBALSYN-PWY | adenosylcobalamin salvage from cobinamide I | -0.70 | 5.34E-10 | 6.65E-08 |
| Neisseria | PWY-7332 | superpathway of UDP-N-acetylglucosamine-derived O-antigen building blocks biosynthesis | -0.70 | 7.20E-10 | 8.37E-08 |
| Neisseria | DENITRIFICATION-PWY | nitrate reduction I (denitrification) | 0.69 | 1.28E-09 | 1.39E-07 |
| Rothia | PWY-7254 | TCA cycle VII (acetate-producers) | 0.69 | 1.61E-09 | 1.65E-07 |
| Neisseria | PWY-7254 | TCA cycle VII (acetate-producers) | 0.68 | 3.52E-09 | 3.40E-07 |
| Porphyromonas | P163-PWY | L-lysine fermentation to acetate and butanoate | 0.67 | 5.16E-09 | 4.73E-07 |
| Neisseria | PWY-1861 | formaldehyde assimilation II (RuMP Cycle) | -0.67 | 6.76E-09 | 5.89E-07 |
| Rothia | COLANSYN-PWY | colanic acid building blocks biosynthesis | -0.67 | 8.20E-09 | 6.24E-07 |
| Neisseria | PWY-5855 | ubiquinol-7 biosynthesis (prokaryotic) | 0.67 | 8.96E-09 | 6.24E-07 |
| Neisseria | PWY-5856 | ubiquinol-9 biosynthesis (prokaryotic) | 0.67 | 8.96E-09 | 6.24E-07 |
| Neisseria | PWY-5857 | ubiquinol-10 biosynthesis (prokaryotic) | 0.67 | 8.96E-09 | 6.24E-07 |
| Neisseria | PWY-6708 | ubiquinol-8 biosynthesis (prokaryotic) | 0.67 | 8.96E-09 | 6.24E-07 |
| Neisseria | RUMP-PWY | formaldehyde oxidation I | -0.66 | 1.05E-08 | 7.00E-07 |
| Rothia | PWY-7323 | superpathway of GDP-mannose-derived O-antigen building blocks biosynthesis | -0.66 | 1.32E-08 | 8.46E-07 |

| | | | | | |
|---|---|---|---|---|---|
| Neisseria | UBISYN-PWY | superpathway of ubiquinol-8 biosynthesis (prokaryotic) | 0.66 | 1.36E-08 | 8.46E-07 |
| Fusobacterium | P163-PWY | L-lysine fermentation to acetate and butanoate | 0.66 | 1.43E-08 | 8.60E-07 |
| Fusobacterium | P162-PWY | L-glutamate degradation V (via hydroxyglutarate) | 0.66 | 1.72E-08 | 9.97E-07 |
| Neisseria | PWY-6901 | superpathway of glucose and xylose degradation | -0.65 | 2.36E-08 | 1.33E-06 |
| Rothia | PWY-5677 | succinate fermentation to butanoate | -0.65 | 2.94E-08 | 1.60E-06 |
| Neisseria | PWY-6269 | adenosylcobalamin salvage from cobinamide II | -0.63 | 6.65E-08 | 3.51E-06 |
| Rothia | PWY-6383 | mono-trans, poly-cis decaprenyl phosphate biosynthesis | 0.63 | 7.17E-08 | 3.67E-06 |
| Neisseria | PWY-5509 | adenosylcobalamin biosynthesis from cobyrinate a,c-diamide I | -0.63 | 7.76E-08 | 3.86E-06 |
| Capnocytophaga | P125-PWY | superpathway of (R,R)-butanediol biosynthesis | 0.63 | 8.52E-08 | 4.12E-06 |
| Rothia | PWY-5505 | L-glutamate and L-glutamine biosynthesis | -0.63 | 9.02E-08 | 4.25E-06 |
| Neisseria | COBALSYN-PWY | adenosylcobalamin salvage from cobinamide I | -0.63 | 1.18E-07 | 5.27E-06 |
| Porphyromonas | PWY-5677 | succinate fermentation to butanoate | 0.63 | 1.17E-07 | 5.27E-06 |
| Parvimonas | P163-PWY | L-lysine fermentation to acetate and butanoate | 0.62 | 1.38E-07 | 5.99E-06 |
| Parvimonas | P162-PWY | L-glutamate degradation V (via hydroxyglutarate) | 0.62 | 1.41E-07 | 5.99E-06 |
| Neisseria | PWY-6728 | methylaspartate cycle | -0.62 | 1.53E-07 | 6.36E-06 |
| Neisseria | PWY-5677 | succinate fermentation to butanoate | -0.61 | 2.47E-07 | 1.00E-05 |
| Rothia | PWY-6728 | methylaspartate cycle | -0.61 | 2.66E-07 | 1.05E-05 |
| Neisseria | P108-PWY | pyruvate fermentation to propanoate I | -0.61 | 2.89E-07 | 1.12E-05 |
| Rothia | PWY-6901 | superpathway of glucose and xylose degradation | -0.61 | 3.22E-07 | 1.22E-05 |
| Rothia | PYRIDOXSYN-PWY | pyridoxal 5'-phosphate biosynthesis I | -0.60 | 5.46E-07 | 1.97E-05 |
| Tannerella | PWY-5509 | adenosylcobalamin biosynthesis from cobyrinate a,c-diamide I | 0.60 | 5.53E-07 | 1.97E-05 |
| Parvimonas | PWY-6588 | pyruvate fermentation to acetone | 0.60 | 5.42E-07 | 1.97E-05 |
| Tannerella | P108-PWY | pyruvate fermentation to propanoate I | 0.60 | 5.76E-07 | 2.01E-05 |
| Rothia | P108-PWY | pyruvate fermentation to propanoate I | -0.60 | 5.91E-07 | 2.02E-05 |
| Tannerella | P163-PWY | L-lysine fermentation to acetate and butanoate | 0.60 | 6.38E-07 | 2.14E-05 |
| Tannerella | PWY-6269 | adenosylcobalamin salvage from cobinamide II | 0.59 | 6.97E-07 | 2.29E-05 |
| Rothia | P164-PWY | purine nucleobases degradation I (anaerobic) | -0.59 | 7.54E-07 | 2.43E-05 |
| Filifactor | UBISYN-PWY | superpathway of ubiquinol-8 biosynthesis (prokaryotic) | -0.59 | 7.93E-07 | 2.51E-05 |
| Lautropia | CHLOROPHYLL-SYN | chlorophyllide a biosynthesis I (aerobic, light-dependent) | -0.59 | 8.88E-07 | 2.76E-05 |
| Filifactor | PWY-5855 | ubiquinol-7 biosynthesis (prokaryotic) | -0.59 | 9.77E-07 | 2.84E-05 |
| Filifactor | PWY-5856 | ubiquinol-9 biosynthesis (prokaryotic) | -0.59 | 9.77E-07 | 2.84E-05 |
| Filifactor | PWY-5857 | ubiquinol-10 biosynthesis (prokaryotic) | -0.59 | 9.77E-07 | 2.84E-05 |
| Filifactor | PWY-6708 | ubiquinol-8 biosynthesis (prokaryotic) | -0.59 | 9.77E-07 | 2.84E-05 |
| Rothia | PWY-7374 | 1,4-dihydroxy-6-naphthoate biosynthesis I | -0.59 | 1.01E-06 | 2.88E-05 |

| | | | | | |
|---|---|---|---|---|---|
| Tannerella | PWY-7090 | UDP-2,3-diacetamido-2,3-dideoxy-α-D-mannuronate biosynthesis | 0.59 | 1.08E-06 | 3.04E-05 |
| Tannerella | COBALSYN-PWY | adenosylcobalamin salvage from cobinamide I | 0.58 | 1.29E-06 | 3.56E-05 |
| Filifactor | SULFATE-CYS-PWY | superpathway of sulfate assimilation and cysteine biosynthesis | -0.58 | 1.41E-06 | 3.83E-05 |
| Tannerella | COLANSYN-PWY | colanic acid building blocks biosynthesis | 0.58 | 1.44E-06 | 3.85E-05 |
| Rothia | PWY-6608 | guanosine nucleotides degradation III | -0.58 | 1.49E-06 | 3.87E-05 |
| Tannerella | PWY-7323 | superpathway of GDP-mannose-derived O-antigen building blocks biosynthesis | 0.58 | 1.47E-06 | 3.87E-05 |
| Rothia | CODH-PWY | reductive acetyl coenzyme A pathway | -0.58 | 1.51E-06 | 3.87E-05 |
| Lautropia | COLANSYN-PWY | colanic acid building blocks biosynthesis | -0.58 | 1.54E-06 | 3.89E-05 |
| Rothia | PWY-1861 | formaldehyde assimilation II (RuMP Cycle) | -0.57 | 1.95E-06 | 4.85E-05 |
| Porphyromonas | PWY-6588 | pyruvate fermentation to acetone | 0.57 | 1.98E-06 | 4.85E-05 |
| Haemophilus | PWY-5747 | 2-methylcitrate cycle II | 0.57 | 2.15E-06 | 5.21E-05 |
| Capnocytophaga | PWY-7254 | TCA cycle VII (acetate-producers) | 0.57 | 2.20E-06 | 5.24E-05 |
| Neisseria | PWY-7374 | 1,4-dihydroxy-6-naphthoate biosynthesis I | -0.57 | 2.33E-06 | 5.49E-05 |
| Haemophilus | PWY-5855 | ubiquinol-7 biosynthesis (prokaryotic) | 0.57 | 2.58E-06 | 5.62E-05 |
| Haemophilus | PWY-5856 | ubiquinol-9 biosynthesis (prokaryotic) | 0.57 | 2.58E-06 | 5.62E-05 |
| Haemophilus | PWY-5857 | ubiquinol-10 biosynthesis (prokaryotic) | 0.57 | 2.58E-06 | 5.62E-05 |
| Haemophilus | PWY-6708 | ubiquinol-8 biosynthesis (prokaryotic) | 0.57 | 2.58E-06 | 5.62E-05 |
| Haemophilus | SULFATE-CYS-PWY | superpathway of sulfate assimilation and cysteine biosynthesis | 0.57 | 2.55E-06 | 5.62E-05 |
| Haemophilus | UBISYN-PWY | superpathway of ubiquinol-8 biosynthesis (prokaryotic) | 0.57 | 2.46E-06 | 5.62E-05 |
| Porphyromonas | P162-PWY | L-glutamate degradation V (via hydroxyglutarate) | 0.57 | 2.62E-06 | 5.63E-05 |
| Neisseria | COLANSYN-PWY | colanic acid building blocks biosynthesis | -0.57 | 2.68E-06 | 5.69E-05 |
| Rothia | RUMP-PWY | formaldehyde oxidation I | -0.57 | 2.86E-06 | 6.00E-05 |
| Neisseria | CODH-PWY | reductive acetyl coenzyme A pathway | -0.57 | 3.02E-06 | 6.27E-05 |
| Porphyromonas | PWY-5509 | adenosylcobalamin biosynthesis from cobyrinate a,c-diamide I | 0.57 | 3.12E-06 | 6.39E-05 |
| Rothia | METHANOGENESIS-PWY | methanogenesis from H2 and CO2 | -0.56 | 3.33E-06 | 6.75E-05 |
| Capnocytophaga | PWY-5747 | 2-methylcitrate cycle II | 0.56 | 3.47E-06 | 6.96E-05 |
| Lautropia | METHANOGENESIS-PWY | methanogenesis from H2 and CO2 | -0.56 | 3.87E-06 | 7.66E-05 |
| Neisseria | P164-PWY | purine nucleobases degradation I (anaerobic) | -0.56 | 4.33E-06 | 8.47E-05 |
| Porphyromonas | PWY-6269 | adenosylcobalamin salvage from cobinamide II | 0.56 | 4.38E-06 | 8.47E-05 |
| Neisseria | PWY-7323 | superpathway of GDP-mannose-derived O-antigen building blocks biosynthesis | -0.56 | 4.63E-06 | 8.86E-05 |
| Neisseria | METHANOGENESIS-PWY | methanogenesis from H2 and CO2 | -0.56 | 4.84E-06 | 9.17E-05 |
| Capnocytophaga | PWY-5855 | ubiquinol-7 biosynthesis (prokaryotic) | 0.55 | 5.15E-06 | 9.34E-05 |
| Capnocytophaga | PWY-5856 | ubiquinol-9 biosynthesis (prokaryotic) | 0.55 | 5.15E-06 | 9.34E-05 |

| | | | | | |
|---|---|---|---|---|---|
| Capnocytophaga | PWY-5857 | ubiquinol-10 biosynthesis (prokaryotic) | 0.55 | 5.15E-06 | 9.34E-05 |
| Capnocytophaga | PWY-6708 | ubiquinol-8 biosynthesis (prokaryotic) | 0.55 | 5.15E-06 | 9.34E-05 |
| Capnocytophaga | DENITRIFICATION-PWY | nitrate reduction I (denitrification) | 0.55 | 6.07E-06 | 0.000108923 |
| Neisseria | SULFATE-CYS-PWY | superpathway of sulfate assimilation and cysteine biosynthesis | 0.55 | 6.22E-06 | 0.000110495 |
| Rothia | PWY-6545 | pyrimidine deoxyribonucleotides de novo biosynthesis III | -0.55 | 6.56E-06 | 0.000115455 |
| Rothia | PWY-6892 | thiazole biosynthesis I (E. coli) | -0.55 | 6.67E-06 | 0.000115455 |
| Neisseria | PWY-6545 | pyrimidine deoxyribonucleotides de novo biosynthesis III | -0.55 | 6.89E-06 | 0.000115455 |
| Porphyromonas | COBALSYN-PWY | adenosylcobalamin salvage from cobinamide I | 0.55 | 6.80E-06 | 0.000115455 |
| Parvimonas | PWY-6891 | thiazole biosynthesis II (Bacillus) | 0.55 | 6.84E-06 | 0.000115455 |
| Pseudo-propionibacterium | PWY-5177 | glutaryl-CoA degradation | -0.55 | 6.73E-06 | 0.000115455 |
| Fusobacterium | PWY-6891 | thiazole biosynthesis II (Bacillus) | 0.55 | 7.04E-06 | 0.00011607 |
| Capnocytophaga | UBISYN-PWY | superpathway of ubiquinol-8 biosynthesis (prokaryotic) | 0.55 | 7.06E-06 | 0.00011607 |
| Capnocytophaga | PWY-7332 | superpathway of UDP-N-acetylglucosamine-derived O-antigen building blocks biosynthesis | -0.55 | 7.28E-06 | 0.000118454 |
| Parvimonas | PWY-7377 | cob(II)yrinate a,c-diamide biosynthesis I (early cobalt insertion) | 0.54 | 8.10E-06 | 0.000130685 |
| Filifactor | P162-PWY | L-glutamate degradation V (via hydroxyglutarate) | 0.54 | 8.34E-06 | 0.000133355 |
| Filifactor | PWY-5345 | superpathway of L-methionine biosynthesis (by sulfhydrylation) | -0.54 | 9.41E-06 | 0.000148962 |
| Filifactor | P163-PWY | L-lysine fermentation to acetate and butanoate | 0.54 | 1.10E-05 | 0.000172467 |
| Neisseria | PWY-6892 | thiazole biosynthesis I (E. coli) | -0.53 | 1.29E-05 | 0.000199874 |
| Rothia | PWY0-845 | superpathway of pyridoxal 5'-phosphate biosynthesis and salvage | -0.53 | 1.37E-05 | 0.000209487 |
| Pseudo-propionibacterium | METHANOGENESIS-PWY | methanogenesis from H2 and CO2 | -0.53 | 1.37E-05 | 0.000209487 |
| Tannerella | PYRIDOXSYN-PWY | pyridoxal 5'-phosphate biosynthesis I | 0.53 | 1.40E-05 | 0.000211995 |
| Neisseria | PWY-6608 | guanosine nucleotides degradation III | -0.53 | 1.44E-05 | 0.000215388 |
| Lautropia | PWY-5304 | superpathway of sulfur oxidation (Acidianus ambivalens) | -0.53 | 1.45E-05 | 0.000215388 |
| Rothia | PWY-7377 | cob(II)yrinate a,c-diamide biosynthesis I (early cobalt insertion) | -0.53 | 1.54E-05 | 0.000227202 |
| Capnocytophaga | PWY-1861 | formaldehyde assimilation II (RuMP Cycle) | -0.53 | 1.60E-05 | 0.000234696 |
| Fusobacterium | PWY-7377 | cob(II)yrinate a,c-diamide biosynthesis I (early cobalt insertion) | 0.53 | 1.67E-05 | 0.000242425 |
| Rothia | PWY-7090 | UDP-2,3-diacetamido-2,3-dideoxy-α-D-mannuronate biosynthesis | -0.53 | 1.70E-05 | 0.000244968 |
| Lautropia | PWY-7332 | superpathway of UDP-N-acetylglucosamine-derived O-antigen building blocks biosynthesis | -0.53 | 1.80E-05 | 0.000256661 |
| Capnocytophaga | PWY-6728 | methylaspartate cycle | -0.53 | 1.86E-05 | 0.000261617 |
| Capnocytophaga | RUMP-PWY | formaldehyde oxidation I | -0.53 | 1.85E-05 | 0.000261617 |
| Capnocytophaga | PWY-6269 | adenosylcobalamin salvage from cobinamide II | -0.53 | 1.93E-05 | 0.000268847 |
| Lautropia | PWY-5529 | superpathway of bacteriochlorophyll a biosynthesis | -0.52 | 2.16E-05 | 0.000298125 |

| | | | | | |
|---|---|---|---|---|---|
| Capnocytophaga | PWY-5509 | adenosylcobalamin biosynthesis from cobyrinate a,c-diamide I | -0.52 | 2.20E-05 | 0.00029983 |
| Capnocytophaga | PWY-6901 | superpathway of glucose and xylose degradation | -0.52 | 2.19E-05 | 0.00029983 |
| Porphyromonas | PWY-6891 | thiazole biosynthesis II (Bacillus) | 0.52 | 2.25E-05 | 0.000303495 |
| Porphyromonas | PWY-6895 | superpathway of thiamin diphosphate biosynthesis II | 0.52 | 2.34E-05 | 0.000314159 |
| Haemophilus | PWY-7254 | TCA cycle VII (acetate-producers) | 0.52 | 2.53E-05 | 0.000336511 |
| Porphyromonas | PWY-3781 | aerobic respiration I (cytochrome c) | -0.52 | 2.62E-05 | 0.000345796 |
| Filifactor | CODH-PWY | reductive acetyl coenzyme A pathway | 0.52 | 2.71E-05 | 0.000354342 |
| Rothia | PWY-6588 | pyruvate fermentation to acetone | -0.51 | 3.14E-05 | 0.000405161 |
| Filifactor | PWY-5920 | superpathway of heme biosynthesis from glycine | -0.51 | 3.12E-05 | 0.000405161 |
| Lautropia | PWY-6728 | methylaspartate cycle | -0.51 | 3.35E-05 | 0.000426228 |
| Capnocytophaga | COBALSYN-PWY | adenosylcobalamin salvage from cobinamide I | -0.51 | 3.34E-05 | 0.000426228 |
| Filifactor | PWY-5747 | 2-methylcitrate cycle II | -0.51 | 3.47E-05 | 0.000437678 |
| Lautropia | P125-PWY | superpathway of (R,R)-butanediol biosynthesis | 0.51 | 3.51E-05 | 0.000439795 |
| Haemophilus | PWY-5345 | superpathway of L-methionine biosynthesis (by sulfhydrylation) | 0.51 | 4.04E-05 | 0.000502921 |
| Filifactor | PWY-7254 | TCA cycle VII (acetate-producers) | -0.51 | 4.09E-05 | 0.000505783 |
| Capnocytophaga | PWY-7323 | superpathway of GDP-mannose-derived O-antigen building blocks biosynthesis | -0.51 | 4.18E-05 | 0.00051263 |
| Filifactor | GLYCOL-GLYOXDEG-PWY | superpathway of glycol metabolism and degradation | -0.50 | 4.68E-05 | 0.000570252 |
| Lautropia | PWY-7323 | superpathway of GDP-mannose-derived O-antigen building blocks biosynthesis | -0.50 | 4.90E-05 | 0.000592162 |
| Rothia | P163-PWY | L-lysine fermentation to acetate and butanoate | -0.50 | 5.08E-05 | 0.000610548 |
| Neisseria | PWY-6588 | pyruvate fermentation to acetone | -0.50 | 5.50E-05 | 0.000656422 |

positive, rho > 0.5 negative, rho < -0.5 p_adj<=0.05

## 17.2 Family

| **Family** | **p2PW** | **description** | **Spearman_rho** | **p_val** | **p_adj** |
|---|---|---|---|---|---|
| Campylobacteraceae | PWY-7031 | protein N-glycosylation (bacterial) | 0.86 | 4.54E-18 | 5.17E-15 |
| Tannerellaceae | PWY-5677 | succinate fermentation to butanoate | 0.82 | 2.38E-15 | 1.36E-12 |
| Micrococcaceae | ENTBACSYN-PWY | enterobactin biosynthesis | 0.81 | 5.48E-15 | 2.08E-12 |
| Micrococcaceae | PWY1G-0 | mycothiol biosynthesis | 0.81 | 1.36E-14 | 3.87E-12 |
| Tannerellaceae | PWY-7332 | superpathway of UDP-N-acetylglucosamine-derived O-antigen building blocks biosynthesis | 0.76 | 2.40E-12 | 5.47E-10 |
| Campylobacteraceae | PWY-7373 | superpathway of demethylmenaquinol-6 biosynthesis II | 0.74 | 2.07E-11 | 3.90E-09 |
| Micrococcaceae | PWY-5509 | adenosylcobalamin biosynthesis from cobyrinate a,c-diamide I | -0.74 | 2.58E-11 | 3.90E-09 |
| Micrococcaceae | PWY-6269 | adenosylcobalamin salvage from cobinamide II | -0.74 | 2.74E-11 | 3.90E-09 |

| | | | | | |
|---|---|---|---|---|---|
| Micrococcaceae | PWY-7332 | superpathway of UDP-N-acetylglucosamine-derived O-antigen building blocks biosynthesis | -0.73 | 5.74E-11 | 7.26E-09 |
| Neisseriaceae | P125-PWY | superpathway of (R,R)-butanediol biosynthesis | 0.72 | 8.89E-11 | 1.01E-08 |
| Micrococcaceae | COBALSYN-PWY | adenosylcobalamin salvage from cobinamide I | -0.72 | 1.24E-10 | 1.28E-08 |
| Micrococcaceae | P125-PWY | superpathway of (R,R)-butanediol biosynthesis | 0.71 | 4.34E-10 | 4.12E-08 |
| Neisseriaceae | PWY-7332 | superpathway of UDP-N-acetylglucosamine-derived O-antigen building blocks biosynthesis | -0.70 | 4.75E-10 | 4.16E-08 |
| Micrococcaceae | PWY-7254 | TCA cycle VII (acetate-producers) | 0.70 | 6.16E-10 | 5.01E-08 |
| Neisseriaceae | PWY-1861 | formaldehyde assimilation II (RuMP Cycle) | -0.70 | 8.39E-10 | 6.37E-08 |
| Neisseriaceae | PWY-5747 | 2-methylcitrate cycle II | 0.70 | 9.81E-10 | 6.98E-08 |
| Neisseriaceae | RUMP-PWY | formaldehyde oxidation I | -0.69 | 1.22E-09 | 8.21E-08 |
| Tannerellaceae | P108-PWY | pyruvate fermentation to propanoate I | 0.69 | 1.45E-09 | 9.19E-08 |
| Micrococcaceae | PWY-6728 | methylaspartate cycle | -0.67 | 4.85E-09 | 2.90E-07 |
| Neisseriaceae | DENITRIFICATION-PWY | nitrate reduction I (denitrification) | 0.67 | 5.18E-09 | 2.95E-07 |
| Micrococcaceae | COLANSYN-PWY | colanic acid building blocks biosynthesis | -0.67 | 6.04E-09 | 3.17E-07 |
| Micrococcaceae | PWY-5677 | succinate fermentation to butanoate | -0.67 | 6.12E-09 | 3.17E-07 |
| Family XI | P163-PWY | L-lysine fermentation to acetate and butanoate | 0.66 | 1.21E-08 | 5.75E-07 |
| Flavobacteriaceae | P125-PWY | superpathway of (R,R)-butanediol biosynthesis | 0.66 | 1.21E-08 | 5.75E-07 |
| Micrococcaceae | PWY-7323 | superpathway of GDP-mannose-derived O-antigen building blocks biosynthesis | -0.66 | 1.54E-08 | 7.01E-07 |
| Neisseriaceae | PWY-6901 | superpathway of glucose and xylose degradation | -0.66 | 1.62E-08 | 7.09E-07 |
| Family XI | P162-PWY | L-glutamate degradation V (via hydroxyglutarate) | 0.66 | 1.73E-08 | 7.30E-07 |
| Tannerellaceae | COLANSYN-PWY | colanic acid building blocks biosynthesis | 0.65 | 2.01E-08 | 8.19E-07 |
| Micrococcaceae | PWY-7374 | 1,4-dihydroxy-6-naphthoate biosynthesis I | -0.65 | 2.33E-08 | 9.14E-07 |
| Family XI | PWY-6588 | pyruvate fermentation to acetone | 0.65 | 2.56E-08 | 9.71E-07 |
| Neisseriaceae | PWY-6728 | methylaspartate cycle | -0.65 | 2.97E-08 | 1.06E-06 |
| Neisseriaceae | PWY-7254 | TCA cycle VII (acetate-producers) | 0.65 | 2.92E-08 | 1.06E-06 |
| Porphyromonadaceae | P163-PWY | L-lysine fermentation to acetate and butanoate | 0.64 | 3.64E-08 | 1.26E-06 |
| Micrococcaceae | PWY-6901 | superpathway of glucose and xylose degradation | -0.64 | 3.79E-08 | 1.27E-06 |
| Micrococcaceae | P108-PWY | pyruvate fermentation to propanoate I | -0.64 | 4.85E-08 | 1.58E-06 |
| Neisseriaceae | PWY-5855 | ubiquinol-7 biosynthesis (prokaryotic) | 0.64 | 6.42E-08 | 1.87E-06 |
| Neisseriaceae | PWY-5856 | ubiquinol-9 biosynthesis (prokaryotic) | 0.64 | 6.42E-08 | 1.87E-06 |
| Neisseriaceae | PWY-5857 | ubiquinol-10 biosynthesis (prokaryotic) | 0.64 | 6.42E-08 | 1.87E-06 |
| Neisseriaceae | PWY-6708 | ubiquinol-8 biosynthesis (prokaryotic) | 0.64 | 6.42E-08 | 1.87E-06 |
| Neisseriaceae | UBISYN-PWY | superpathway of ubiquinol-8 biosynthesis (prokaryotic) | 0.63 | 9.79E-08 | 2.79E-06 |
| Micrococcaceae | PYRIDOXSYN-PWY | pyridoxal 5'-phosphate biosynthesis I | -0.62 | 1.51E-07 | 4.20E-06 |

| | | | | | |
|---|---|---|---|---|---|
| Neisseriaceae | PWY-6269 | adenosylcobalamin salvage from cobinamide II | -0.62 | 1.63E-07 | 4.41E-06 |
| Neisseriaceae | PWY-5677 | succinate fermentation to butanoate | -0.62 | 1.81E-07 | 4.79E-06 |
| Neisseriaceae | PWY-5509 | adenosylcobalamin biosynthesis from cobyrinate a,c-diamide I | -0.62 | 1.92E-07 | 4.85E-06 |
| Tannerellaceae | PYRIDOXSYN-PWY | pyridoxal 5'-phosphate biosynthesis I | 0.62 | 1.88E-07 | 4.85E-06 |
| Fusobacteriaceae | P163-PWY | L-lysine fermentation to acetate and butanoate | 0.61 | 2.23E-07 | 5.53E-06 |
| Micrococcaceae | PWY-5505 | L-glutamate and L-glutamine biosynthesis | -0.61 | 2.53E-07 | 6.13E-06 |
| Tannerellaceae | PWY-6269 | adenosylcobalamin salvage from cobinamide II | 0.61 | 2.69E-07 | 6.39E-06 |
| Tannerellaceae | PWY-5509 | adenosylcobalamin biosynthesis from cobyrinate a,c-diamide I | 0.61 | 2.79E-07 | 6.47E-06 |
| Neisseriaceae | COBALSYN-PWY | adenosylcobalamin salvage from cobinamide I | -0.61 | 2.96E-07 | 6.74E-06 |
| Neisseriaceae | P108-PWY | pyruvate fermentation to propanoate I | -0.61 | 3.17E-07 | 7.08E-06 |
| Fusobacteriaceae | P162-PWY | L-glutamate degradation V (via hydroxyglutarate) | 0.60 | 3.99E-07 | 8.74E-06 |
| Family XI | CODH-PWY | reductive acetyl coenzyme A pathway | 0.60 | 4.18E-07 | 8.81E-06 |
| Flavobacteriaceae | PWY-7332 | superpathway of UDP-N-acetylglucosamine-derived O-antigen building blocks biosynthesis | -0.60 | 4.11E-07 | 8.81E-06 |
| Micrococcaceae | METHANOGENESIS-PWY | methanogenesis from H2 and CO2 | -0.60 | 4.28E-07 | 8.87E-06 |
| Tannerellaceae | PWY-7323 | superpathway of GDP-mannose-derived O-antigen building blocks biosynthesis | 0.60 | 4.78E-07 | 9.73E-06 |
| Porphyromonadaceae | PWY-5677 | succinate fermentation to butanoate | 0.60 | 5.40E-07 | 1.08E-05 |
| Micrococcaceae | P164-PWY | purine nucleobases degradation I (anaerobic) | -0.60 | 6.65E-07 | 1.31E-05 |
| Micrococcaceae | CODH-PWY | reductive acetyl coenzyme A pathway | -0.59 | 6.88E-07 | 1.33E-05 |
| Micrococcaceae | PWY-6383 | mono-trans, poly-cis decaprenyl phosphate biosynthesis | 0.59 | 7.61E-07 | 1.44E-05 |
| Tannerellaceae | COBALSYN-PWY | adenosylcobalamin salvage from cobinamide I | 0.59 | 7.90E-07 | 1.48E-05 |
| Micrococcaceae | PWY-1861 | formaldehyde assimilation II (RuMP Cycle) | -0.59 | 8.81E-07 | 1.62E-05 |
| Micrococcaceae | PWY-6608 | guanosine nucleotides degradation III | -0.59 | 1.07E-06 | 1.94E-05 |
| Micrococcaceae | RUMP-PWY | formaldehyde oxidation I | -0.58 | 1.30E-06 | 2.31E-05 |
| Tannerellaceae | PWY-7090 | UDP-2,3-diacetamido-2,3-dideoxy-α-D-mannuronate biosynthesis | 0.58 | 1.70E-06 | 2.99E-05 |
| Neisseriaceae | PWY-7374 | 1,4-dihydroxy-6-naphthoate biosynthesis I | -0.58 | 1.78E-06 | 3.07E-05 |
| Neisseriaceae | METHANOGENESIS-PWY | methanogenesis from H2 and CO2 | -0.58 | 1.86E-06 | 3.16E-05 |
| Tannerellaceae | PWY-6901 | superpathway of glucose and xylose degradation | 0.57 | 2.14E-06 | 3.58E-05 |
| Neisseriaceae | CODH-PWY | reductive acetyl coenzyme A pathway | -0.57 | 2.24E-06 | 3.60E-05 |
| Neisseriaceae | COLANSYN-PWY | colanic acid building blocks biosynthesis | -0.57 | 2.19E-06 | 3.60E-05 |
| Flavobacteriaceae | PWY-7254 | TCA cycle VII (acetate-producers) | 0.57 | 2.23E-06 | 3.60E-05 |
| Tannerellaceae | PWY-7373 | superpathway of demethylmenaquinol-6 biosynthesis II | 0.57 | 2.31E-06 | 3.65E-05 |
| Micrococcaceae | PWY-6892 | thiazole biosynthesis I (E. coli) | -0.57 | 2.48E-06 | 3.86E-05 |
| Neisseriaceae | P164-PWY | purine nucleobases degradation I (anaerobic) | -0.57 | 2.99E-06 | 4.60E-05 |

| | | | | | |
|---|---|---|---|---|---|
| Micrococcaceae | PWY-7090 | UDP-2,3-diacetamido-2,3-dideoxy-α-D-mannuronate biosynthesis | -0.56 | 3.46E-06 | 5.21E-05 |
| Flavobacteriaceae | PWY-6901 | superpathway of glucose and xylose degradation | -0.56 | 3.47E-06 | 5.21E-05 |
| Tannerellaceae | PWY-7254 | TCA cycle VII (acetate-producers) | -0.56 | 4.02E-06 | 5.95E-05 |
| Flavobacteriaceae | PWY-5747 | 2-methylcitrate cycle II | 0.56 | 4.46E-06 | 6.52E-05 |
| Neisseriaceae | PWY-7323 | superpathway of GDP-mannose-derived O-antigen building blocks biosynthesis | -0.56 | 4.56E-06 | 6.58E-05 |
| Micrococcaceae | PWY0-845 | superpathway of pyridoxal 5'-phosphate biosynthesis and salvage | -0.56 | 4.84E-06 | 6.89E-05 |
| Flavobacteriaceae | PWY-7323 | superpathway of GDP-mannose-derived O-antigen building blocks biosynthesis | -0.56 | 5.06E-06 | 7.11E-05 |
| Flavobacteriaceae | PWY-5855 | ubiquinol-7 biosynthesis (prokaryotic) | 0.55 | 5.82E-06 | 7.62E-05 |
| Flavobacteriaceae | PWY-5856 | ubiquinol-9 biosynthesis (prokaryotic) | 0.55 | 5.82E-06 | 7.62E-05 |
| Flavobacteriaceae | PWY-5857 | ubiquinol-10 biosynthesis (prokaryotic) | 0.55 | 5.82E-06 | 7.62E-05 |
| Flavobacteriaceae | PWY-6269 | adenosylcobalamin salvage from cobinamide II | -0.55 | 5.49E-06 | 7.62E-05 |
| Flavobacteriaceae | PWY-6708 | ubiquinol-8 biosynthesis (prokaryotic) | 0.55 | 5.82E-06 | 7.62E-05 |
| Flavobacteriaceae | PWY-6728 | methylaspartate cycle | -0.55 | 5.74E-06 | 7.62E-05 |
| Flavobacteriaceae | PWY-5509 | adenosylcobalamin biosynthesis from cobyrinate a,c-diamide I | -0.55 | 5.90E-06 | 7.64E-05 |
| Flavobacteriaceae | COLANSYN-PWY | colanic acid building blocks biosynthesis | -0.55 | 6.13E-06 | 7.85E-05 |
| Micrococcaceae | PWY-6545 | pyrimidine deoxyribonucleotides de novo biosynthesis III | -0.55 | 6.48E-06 | 8.20E-05 |
| Tannerellaceae | P163-PWY | L-lysine fermentation to acetate and butanoate | 0.55 | 6.73E-06 | 8.42E-05 |
| Tannerellaceae | P125-PWY | superpathway of (R,R)-butanediol biosynthesis | -0.55 | 7.24E-06 | 8.96E-05 |
| Neisseriaceae | PWY-6545 | pyrimidine deoxyribonucleotides de novo biosynthesis III | -0.54 | 8.92E-06 | 0.000109242 |
| Neisseriaceae | PWY-6608 | guanosine nucleotides degradation III | -0.54 | 9.11E-06 | 0.000110404 |
| Flavobacteriaceae | UBISYN-PWY | superpathway of ubiquinol-8 biosynthesis (prokaryotic) | 0.54 | 9.48E-06 | 0.000113677 |
| Flavobacteriaceae | PWY-5677 | succinate fermentation to butanoate | -0.54 | 9.80E-06 | 0.000116235 |
| Porphyromonadaceae | PWY-6588 | pyruvate fermentation to acetone | 0.54 | 1.05E-05 | 0.000120597 |
| Tannerellaceae | PWY0-845 | superpathway of pyridoxal 5'-phosphate biosynthesis and salvage | 0.54 | 1.04E-05 | 0.000120597 |
| Flavobacteriaceae | COBALSYN-PWY | adenosylcobalamin salvage from cobinamide I | -0.54 | 1.03E-05 | 0.000120597 |
| Micrococcaceae | PWY-6588 | pyruvate fermentation to acetone | -0.54 | 1.10E-05 | 0.0001255 |
| Micrococcaceae | PWY-7377 | cob(II)yrinate a,c-diamide biosynthesis I (early cobalt insertion) | -0.54 | 1.18E-05 | 0.000133 |
| Porphyromonadaceae | PWY-5509 | adenosylcobalamin biosynthesis from cobyrinate a,c-diamide I | 0.54 | 1.22E-05 | 0.000136234 |
| Flavobacteriaceae | PWY-1861 | formaldehyde assimilation II (RuMP Cycle) | -0.54 | 1.27E-05 | 0.000140275 |
| Family XI | PWY-7377 | cob(II)yrinate a,c-diamide biosynthesis I (early cobalt insertion) | 0.53 | 1.31E-05 | 0.000143316 |
| Flavobacteriaceae | DENITRIFICATION-PWY | nitrate reduction I (denitrification) | 0.53 | 1.41E-05 | 0.000153386 |
| Flavobacteriaceae | RUMP-PWY | formaldehyde oxidation I | -0.53 | 1.44E-05 | 0.000154045 |
| Pasteurellaceae | PWY-5747 | 2-methylcitrate cycle II | 0.53 | 1.45E-05 | 0.000154045 |
| Porphyromonadaceae | P162-PWY | L-glutamate degradation V (via hydroxyglutarate) | 0.53 | 1.48E-05 | 0.00015659 |

| | | | | | |
|---|---|---|---|---|---|
| Porphyromonadaceae | PWY-6269 | adenosylcobalamin salvage from cobinamide II | 0.53 | 1.64E-05 | 0.00017186 |
| Neisseriaceae | SULFATE-CYS-PWY | superpathway of sulfate assimilation and cysteine biosynthesis | 0.53 | 1.78E-05 | 0.000184249 |
| Tannerellaceae | PWY-7031 | protein N-glycosylation (bacterial) | 0.53 | 1.80E-05 | 0.000184409 |
| Micrococcaceae | P163-PWY | L-lysine fermentation to acetate and butanoate | -0.53 | 1.86E-05 | 0.000189384 |
| Micrococcaceae | PWY-5104 | L-isoleucine biosynthesis IV | -0.52 | 2.28E-05 | 0.000229944 |
| Porphyromonadaceae | PWY-3781 | aerobic respiration I (cytochrome c) | -0.52 | 2.43E-05 | 0.000243113 |
| Porphyromonadaceae | COBALSYN-PWY | adenosylcobalamin salvage from cobinamide I | 0.52 | 2.58E-05 | 0.000255714 |
| Neisseriaceae | PWY-6892 | thiazole biosynthesis I (E. coli) | -0.52 | 2.70E-05 | 0.000264987 |
| Flavobacteriaceae | CODH-PWY | reductive acetyl coenzyme A pathway | -0.52 | 2.90E-05 | 0.000282063 |
| Flavobacteriaceae | P164-PWY | purine nucleobases degradation I (anaerobic) | -0.51 | 3.37E-05 | 0.000325128 |
| Flavobacteriaceae | PYRIDOXSYN-PWY | pyridoxal 5'-phosphate biosynthesis I | -0.51 | 3.65E-05 | 0.00034906 |
| Family XI | PWY-6891 | thiazole biosynthesis II (Bacillus) | 0.51 | 4.21E-05 | 0.000396166 |
| Pasteurellaceae | CODH-PWY | reductive acetyl coenzyme A pathway | -0.51 | 4.19E-05 | 0.000396166 |
| Pasteurellaceae | RUMP-PWY | formaldehyde oxidation I | -0.51 | 4.45E-05 | 0.00041338 |
| Peptococcaceae | PWY0-42 | 2-methylcitrate cycle I | -0.51 | 4.46E-05 | 0.00041338 |
| Micrococcaceae | PWY-3781 | aerobic respiration I (cytochrome c) | 0.50 | 4.57E-05 | 0.000419971 |
| Fusobacteriaceae | PWY-6891 | thiazole biosynthesis II (Bacillus) | 0.50 | 4.68E-05 | 0.000426548 |
| Pasteurellaceae | PWY-1861 | formaldehyde assimilation II (RuMP Cycle) | -0.50 | 4.85E-05 | 0.000438358 |
| Neisseriaceae | PYRIDOXSYN-PWY | pyridoxal 5'-phosphate biosynthesis I | -0.50 | 4.95E-05 | 0.00044419 |
| Porphyromonadaceae | PWY-6895 | superpathway of thiamin diphosphate biosynthesis II | 0.50 | 5.31E-05 | 0.000472748 |

positive, rho > 0.5 negative, rho < -0.5 p_adj<=0.05

## Table 18. KEGG enrichment of ANCOM-BC2 selected PICRUSt2 predicted KO terms

Global ($q < 0.2$, passed the sensitivity analysis)

| ID | Description | GeneRatio | BgRatio | pvalue | p.adjust | qvalue | Count |
|---|---|---|---|---|---|---|---|
| map02020 | Two-component system | 134/1062 | 499/13584 | 2.80011E-39 | 7.25228E-37 | 5.51179E-37 | 134 |
| map01240 | Biosynthesis of cofactors | 102/1062 | 375/13584 | 2.04625E-30 | 2.64989E-28 | 2.01394E-28 | 102 |
| map01200 | Carbon metabolism | 93/1062 | 365/13584 | 1.78769E-25 | 1.54337E-23 | 1.17298E-23 | 93 |
| map01230 | Biosynthesis of amino acids | 70/1062 | 238/13584 | 2.79667E-23 | 1.81084E-21 | 1.37625E-21 | 70 |
| map02040 | Flagellar assembly | 33/1062 | 55/13584 | 4.50478E-23 | 2.33348E-21 | 1.77346E-21 | 33 |
| map00860 | Porphyrin metabolism | 49/1062 | 139/13584 | 2.26646E-20 | 9.78357E-19 | 7.43559E-19 | 49 |
| map00620 | Pyruvate metabolism | 43/1062 | 133/13584 | 1.93973E-16 | 7.17701E-15 | 5.45459E-15 | 43 |
| map00650 | Butanoate metabolism | 39/1062 | 114/13584 | 5.70062E-16 | 1.84558E-14 | 1.40265E-14 | 39 |
| map00020 | Citrate cycle (TCA cycle) | 29/1062 | 67/13584 | 2.398E-15 | 6.90091E-14 | 5.24475E-14 | 29 |
| map00630 | Glyoxylate and dicarboxylate metabolism | 35/1062 | 104/13584 | 3.18033E-14 | 8.23706E-13 | 6.26023E-13 | 35 |
| map00520 | Amino sugar and nucleotide sugar metabolism | 43/1062 | 156/13584 | 1.11518E-13 | 2.62573E-12 | 1.99558E-12 | 43 |
| map02060 | Phosphotransferase system (PTS) | 28/1062 | 72/13584 | 1.90692E-13 | 4.11576E-12 | 3.12801E-12 | 28 |
| map02030 | Bacterial chemotaxis | 17/1062 | 26/13584 | 2.14232E-13 | 4.26816E-12 | 3.24384E-12 | 17 |
| map00640 | Propanoate metabolism | 31/1062 | 97/13584 | 4.44673E-12 | 8.22644E-11 | 6.25216E-11 | 31 |
| map03070 | Bacterial secretion system | 26/1062 | 74/13584 | 2.11625E-11 | 3.65405E-10 | 2.77711E-10 | 26 |
| map00720 | Carbon fixation pathways in prokaryotes | 33/1062 | 117/13584 | 4.15512E-11 | 6.72609E-10 | 5.11189E-10 | 33 |
| map00500 | Starch and sucrose metabolism | 30/1062 | 106/13584 | 2.90706E-10 | 4.429E-09 | 3.36607E-09 | 30 |
| map00010 | Glycolysis / Gluconeogenesis | 29/1062 | 106/13584 | 1.38101E-09 | 1.98712E-08 | 1.51022E-08 | 29 |
| map00920 | Sulfur metabolism | 29/1062 | 109/13584 | 2.80691E-09 | 3.82626E-08 | 2.90799E-08 | 29 |
| map00910 | Nitrogen metabolism | 22/1062 | 68/13584 | 4.41589E-09 | 5.71857E-08 | 4.34616E-08 | 22 |
| map00540 | Lipopolysaccharide biosynthesis | 20/1062 | 62/13584 | 2.35413E-08 | 2.90343E-07 | 2.20663E-07 | 20 |
| map00270 | Cysteine and methionine metabolism | 29/1062 | 124/13584 | 6.46501E-08 | 7.61109E-07 | 5.78449E-07 | 29 |
| map00130 | Ubiquinone and other terpenoid-quinone biosynthesis | 18/1062 | 59/13584 | 3.0609E-07 | 3.44684E-06 | 2.61963E-06 | 18 |
| map00300 | Lysine biosynthesis | 16/1062 | 48/13584 | 3.58238E-07 | 3.86599E-06 | 2.93818E-06 | 16 |
| map00541 | O-Antigen nucleotide sugar biosynthesis | 24/1062 | 99/13584 | 4.29986E-07 | 4.45465E-06 | 3.38557E-06 | 24 |
| map00400 | Phenylalanine, tyrosine and tryptophan biosynthesis | 20/1062 | 74/13584 | 6.09076E-07 | 6.06734E-06 | 4.61123E-06 | 20 |
| map00250 | Alanine, aspartate and glutamate metabolism | 19/1062 | 70/13584 | 1.07977E-06 | 1.03578E-05 | 7.872E-06 | 19 |
| map00051 | Fructose and mannose metabolism | 25/1062 | 112/13584 | 1.31018E-06 | 1.21191E-05 | 9.21064E-06 | 25 |
| map01250 | Biosynthesis of nucleotide sugars | 37/1062 | 211/13584 | 2.57096E-06 | 2.29614E-05 | 1.74508E-05 | 37 |
| map00330 | Arginine and proline metabolism | 22/1062 | 107/13584 | 2.23874E-05 | 0.000193277 | 0.000146892 | 22 |
| map00030 | Pentose phosphate pathway | 19/1062 | 88/13584 | 3.92778E-05 | 0.00032816 | 0.000249404 | 19 |

| | | | | | | | |
|---|---|---|---|---|---|---|---|
| map00280 | Valine, leucine and isoleucine degradation | 16/1062 | 69/13584 | 6.36056E-05 | 0.000514808 | 0.000391258 | 16 |
| map00260 | Glycine, serine and threonine metabolism | 21/1062 | 109/13584 | 9.2808E-05 | 0.000728402 | 0.000553591 | 21 |
| map00190 | Oxidative phosphorylation | 34/1062 | 223/13584 | 0.000125728 | 0.000957754 | 0.000727901 | 34 |
| map01210 | 2-Oxocarboxylic acid metabolism | 17/1062 | 82/13584 | 0.000167575 | 0.001207917 | 0.000918027 | 17 |
| map00220 | Arginine biosynthesis | 14/1062 | 60/13584 | 0.000167896 | 0.001207917 | 0.000918027 | 14 |
| map02025 | Biofilm formation - Pseudomonas aeruginosa | 18/1062 | 90/13584 | 0.00017625 | 0.001233747 | 0.000937658 | 18 |
| map00680 | Methane metabolism | 30/1062 | 195/13584 | 0.000261818 | 0.001784495 | 0.001356231 | 30 |
| map00071 | Fatty acid degradation | 13/1062 | 59/13584 | 0.00051696 | 0.003433147 | 0.00260922 | 13 |
| map00550 | Peptidoglycan biosynthesis | 12/1062 | 53/13584 | 0.000647885 | 0.004195055 | 0.003188276 | 12 |
| map00450 | Selenocompound metabolism | 9/1062 | 33/13584 | 0.000729463 | 0.004608068 | 0.003502169 | 9 |
| map00511 | Other glycan degradation | 7/1062 | 22/13584 | 0.001048894 | 0.00631776 | 0.004801549 | 7 |
| map01502 | Vancomycin resistance | 7/1062 | 22/13584 | 0.001048894 | 0.00631776 | 0.004801549 | 7 |
| map05111 | Biofilm formation - Vibrio cholerae | 18/1062 | 106/13584 | 0.001368517 | 0.00805559 | 0.006122314 | 18 |
| map00710 | Carbon fixation in photosynthetic organisms | 9/1062 | 36/13584 | 0.001439649 | 0.008285981 | 0.006297413 | 9 |
| map00350 | Tyrosine metabolism | 15/1062 | 84/13584 | 0.002001892 | 0.011271523 | 0.008566449 | 15 |
| map02026 | Biofilm formation - Escherichia coli | 12/1062 | 61/13584 | 0.00237128 | 0.013067266 | 0.009931229 | 12 |
| map01503 | Cationic antimicrobial peptide (CAMP) resistance | 11/1062 | 54/13584 | 0.002665971 | 0.014385133 | 0.010932818 | 11 |
| map00340 | Histidine metabolism | 10/1062 | 47/13584 | 0.002940525 | 0.015542774 | 0.011812634 | 10 |
| map00480 | Glutathione metabolism | 11/1062 | 57/13584 | 0.004145608 | 0.02147425 | 0.016320604 | 11 |
| map03410 | Base excision repair | 9/1062 | 43/13584 | 0.005264807 | 0.026484451 | 0.020128398 | 9 |
| map01212 | Fatty acid metabolism | 14/1062 | 84/13584 | 0.005317342 | 0.026484451 | 0.020128398 | 14 |
| map00430 | Taurine and hypotaurine metabolism | 7/1062 | 29/13584 | 0.005930311 | 0.028980201 | 0.022025189 | 7 |
| map01501 | beta-Lactam resistance | 17/1062 | 112/13584 | 0.006067217 | 0.029100173 | 0.022116368 | 17 |
| map00052 | Galactose metabolism | 13/1062 | 78/13584 | 0.007094976 | 0.033410885 | 0.025392544 | 13 |
| map00310 | Lysine degradation | 15/1062 | 98/13584 | 0.008894502 | 0.039792309 | 0.030242478 | 15 |
| map00040 | Pentose and glucuronate interconversions | 14/1062 | 89/13584 | 0.008901423 | 0.039792309 | 0.030242478 | 14 |
| map00780 | Biotin metabolism | 6/1062 | 24/13584 | 0.008911019 | 0.039792309 | 0.030242478 | 6 |
| map00061 | Fatty acid biosynthesis | 8/1062 | 39/13584 | 0.009444181 | 0.041458352 | 0.031508685 | 8 |
| map00360 | Phenylalanine metabolism | 12/1062 | 74/13584 | 0.0117439 | 0.050694503 | 0.038528234 | 12 |
| map00230 | Purine metabolism | 27/1062 | 220/13584 | 0.012885096 | 0.054630585 | 0.041519689 | 27 |
| map00790 | Folate biosynthesis | 13/1062 | 84/13584 | 0.013077592 | 0.054630585 | 0.041519689 | 13 |

RT−GT ($q < 0.2$, passed the sensitivity analysis)

| ID | Description | GeneRatio | BgRatio | pvalue | p.adjust | qvalue | Count |
|---|---|---|---|---|---|---|---|
| map01240 | Biosynthesis of cofactors | 48/381 | 375/13584 | 6.13E-19 | 1.03E-16 | 7.67E-17 | 48 |
| map02020 | Two-component system | 55/381 | 499/13584 | 1.47E-18 | 1.23E-16 | 9.19E-17 | 55 |
| map00860 | Porphyrin metabolism | 26/381 | 139/13584 | 1.12E-14 | 6.28E-13 | 4.68E-13 | 26 |
| map01200 | Carbon metabolism | 38/381 | 365/13584 | 2.61E-12 | 1.1E-10 | 8.18E-11 | 38 |
| map01230 | Biosynthesis of amino acids | 28/381 | 238/13584 | 1.33E-10 | 4.45E-09 | 3.32E-09 | 28 |
| map00650 | Butanoate metabolism | 18/381 | 114/13584 | 2.65E-09 | 7.41E-08 | 5.52E-08 | 18 |
| map00620 | Pyruvate metabolism | 18/381 | 133/13584 | 3.22E-08 | 7.73E-07 | 5.77E-07 | 18 |
| map00920 | Sulfur metabolism | 16/381 | 109/13584 | 5.93E-08 | 1.24E-06 | 9.28E-07 | 16 |
| map00640 | Propanoate metabolism | 15/381 | 97/13584 | 7.58E-08 | 1.41E-06 | 1.05E-06 | 15 |
| map00020 | Citrate cycle (TCA cycle) | 12/381 | 67/13584 | 2.97E-07 | 5E-06 | 3.72E-06 | 12 |
| map02030 | Bacterial chemotaxis | 7/381 | 26/13584 | 5.36E-06 | 8.19E-05 | 6.11E-05 | 7 |
| map00520 | Amino sugar and nucleotide sugar metabolism | 15/381 | 156/13584 | 3.28E-05 | 0.000459 | 0.000343 | 15 |
| map00720 | Carbon fixation pathways in prokaryotes | 12/381 | 117/13584 | 0.000108 | 0.001393 | 0.001039 | 12 |
| map00280 | Valine, leucine and isoleucine degradation | 9/381 | 69/13584 | 0.000125 | 0.001502 | 0.00112 | 9 |
| map00190 | Oxidative phosphorylation | 17/381 | 223/13584 | 0.000186 | 0.002088 | 0.001557 | 17 |
| map00680 | Methane metabolism | 15/381 | 195/13584 | 0.0004 | 0.004203 | 0.003134 | 15 |
| map00541 | O-Antigen nucleotide sugar biosynthesis | 10/381 | 99/13584 | 0.000453 | 0.004477 | 0.003338 | 10 |
| map00910 | Nitrogen metabolism | 8/381 | 68/13584 | 0.000605 | 0.005646 | 0.00421 | 8 |
| map00630 | Glyoxylate and dicarboxylate metabolism | 10/381 | 104/13584 | 0.000671 | 0.005931 | 0.004422 | 10 |
| map00270 | Cysteine and methionine metabolism | 11/381 | 124/13584 | 0.000727 | 0.006105 | 0.004552 | 11 |
| map00010 | Glycolysis / Gluconeogenesis | 10/381 | 106/13584 | 0.000779 | 0.006233 | 0.004647 | 10 |
| map03070 | Bacterial secretion system | 8/381 | 74/13584 | 0.001066 | 0.008142 | 0.006071 | 8 |
| map00660 | C5-Branched dibasic acid metabolism | 5/381 | 29/13584 | 0.001152 | 0.008415 | 0.006275 | 5 |
| map00130 | Ubiquinone and other terpenoid-quinone biosynthesis | 7/381 | 59/13584 | 0.001256 | 0.008791 | 0.006554 | 7 |
| map00220 | Arginine biosynthesis | 7/381 | 60/13584 | 0.001388 | 0.009328 | 0.006955 | 7 |
| map00450 | Selenocompound metabolism | 5/381 | 33/13584 | 0.002101 | 0.013208 | 0.009848 | 5 |
| map00300 | Lysine biosynthesis | 6/381 | 48/13584 | 0.002123 | 0.013208 | 0.009848 | 6 |
| map00350 | Tyrosine metabolism | 8/381 | 84/13584 | 0.002421 | 0.014528 | 0.010832 | 8 |
| map00330 | Arginine and proline metabolism | 9/381 | 107/13584 | 0.003122 | 0.018084 | 0.013483 | 9 |
| map00250 | Alanine, aspartate and glutamate metabolism | 7/381 | 70/13584 | 0.003394 | 0.019007 | 0.014172 | 7 |
| map02025 | Biofilm formation - Pseudomonas aeruginosa | 8/381 | 90/13584 | 0.003725 | 0.020188 | 0.015052 | 8 |
| map00071 | Fatty acid degradation | 6/381 | 59/13584 | 0.006026 | 0.031637 | 0.023589 | 6 |
| map01250 | Biosynthesis of nucleotide sugars | 13/381 | 211/13584 | 0.006631 | 0.033503 | 0.02498 | 13 |

| | | | | | | | |
|---|---|---|---|---|---|---|---|
| map03410 | Base excision repair | 5/381 | 43/13584 | 0.00678 | 0.033503 | 0.02498 | 5 |
| map01210 | 2-Oxocarboxylic acid metabolism | 7/381 | 82/13584 | 0.008096 | 0.038376 | 0.028614 | 7 |
| map00410 | beta-Alanine metabolism | 5/381 | 45/13584 | 0.008223 | 0.038376 | 0.028614 | 5 |
| map00790 | Folate biosynthesis | 7/381 | 84/13584 | 0.0092 | 0.040674 | 0.030327 | 7 |
| map01212 | Fatty acid metabolism | 7/381 | 84/13584 | 0.0092 | 0.040674 | 0.030327 | 7 |
| map00340 | Histidine metabolism | 5/381 | 47/13584 | 0.009867 | 0.042502 | 0.03169 | 5 |

ST−GT ($q < 0.2$, passed the sensitivity analysis)

| ID | Description | GeneRatio | BgRatio | pvalue | p.adjust | qvalue | Count |
|---|---|---|---|---|---|---|---|
| map00190 | Oxidative phosphorylation | 12/92 | 223/13584 | 3.26E-08 | 2.83E-06 | 2.23E-06 | 12 |
| map00020 | Citrate cycle (TCA cycle) | 6/92 | 67/13584 | 5.86E-06 | 0.00018 | 0.000141 | 6 |
| map01200 | Carbon metabolism | 12/92 | 365/13584 | 6.2E-06 | 0.00018 | 0.000141 | 12 |
| map00860 | Porphyrin metabolism | 7/92 | 139/13584 | 4.29E-05 | 0.000773 | 0.000608 | 7 |
| map01240 | Biosynthesis of cofactors | 11/92 | 375/13584 | 4.44E-05 | 0.000773 | 0.000608 | 11 |
| map01053 | Biosynthesis of siderophore group nonribosomal peptides | 4/92 | 36/13584 | 9.83E-05 | 0.001426 | 0.001121 | 4 |
| map00650 | Butanoate metabolism | 6/92 | 114/13584 | 0.000121 | 0.001509 | 0.001187 | 6 |
| map00720 | Carbon fixation pathways in prokaryotes | 6/92 | 117/13584 | 0.00014 | 0.001523 | 0.001198 | 6 |
| map03410 | Base excision repair | 3/92 | 43/13584 | 0.00305 | 0.029484 | 0.023187 | 3 |
| map00640 | Propanoate metabolism | 4/92 | 97/13584 | 0.004226 | 0.035941 | 0.028266 | 4 |
| map00541 | O-Antigen nucleotide sugar biosynthesis | 4/92 | 99/13584 | 0.004544 | 0.035941 | 0.028266 | 4 |
| map00630 | Glyoxylate and dicarboxylate metabolism | 4/92 | 104/13584 | 0.005411 | 0.039228 | 0.030851 | 4 |
| map02020 | Two-component system | 9/92 | 499/13584 | 0.006607 | 0.044213 | 0.034772 | 9 |

Enriched KEGG pathways with $q < 0.05$ are shown.

## Table 19. Spearman's correlation between Maaslin2-identified taxa and ANCOM-BC2-selected PICRUSt2-predicted KO terms

19.1 Genus

| Genus | p2KO | description | Spearman_rho | p_val | p_adj |
|---|---|---|---|---|---|
| Haemophilus | K01347 | iga; IgA-specific serine endopeptidase [EC:3.4.21.72] | 0.94 | 6.46E-28 | 3.06E-23 |
| Neisseria | K18133 | K18133, porB; major outer membrane protein P.IB | 0.93 | 3.92E-27 | 9.30E-23 |
| Neisseria | K07280 | K07280; outer membrane protein | 0.93 | 1.11E-26 | 1.76E-22 |
| Neisseria | K00025 | MDH1; malate dehydrogenase [EC:1.1.1.37] | 0.93 | 2.13E-26 | 2.52E-22 |
| Neisseria | K01947 | birA-coaX; biotin---[acetyl-CoA-carboxylase] ligase / type III pantothenate kinase [EC:6.3.4.15 2.7.1.33] | 0.93 | 9.69E-26 | 9.19E-22 |
| Haemophilus | K11909 | vasI; type VI secretion system protein VasI | 0.90 | 1.59E-22 | 1.26E-18 |
| Neisseria | K12875 | ACIN1, ACINUS; apoptotic chromatin condensation inducer in the nucleus | 0.90 | 1.89E-22 | 1.28E-18 |
| Neisseria | K03675 | grxB; glutaredoxin 2 | 0.90 | 6.25E-22 | 3.71E-18 |
| Rothia | K03343 | puo; putrescine oxidase [EC:1.4.3.10] | 0.90 | 8.30E-22 | 4.37E-18 |
| Neisseria | K16346 | xanQ; xanthine permease XanQ | 0.89 | 4.28E-21 | 2.03E-17 |
| Neisseria | K18148 | rtcB; release factor H-coupled RctB family protein | 0.89 | 5.50E-21 | 2.37E-17 |
| Tannerella | K15899 | pseF; pseudaminic acid cytidylyltransferase [EC:2.7.7.81] | 0.88 | 2.33E-20 | 9.19E-17 |
| Rothia | K15525 | mshB; N-acetyl-1-D-myo-inositol-2-amino-2-deoxy-alpha-D-glucopyranoside deacetylase [EC:3.5.1.103] | 0.88 | 3.23E-20 | 1.18E-16 |
| Rothia | K02364 | entF; enterobactin synthetase component F [EC:6.3.2.14] | 0.88 | 8.51E-20 | 2.69E-16 |
| Rothia | K15733 | E1.11.1.19; dye decolorizing peroxidase [EC:1.11.1.19] | 0.88 | 8.38E-20 | 2.69E-16 |
| Campylobacter | K16293 | psrB; polysulfide reductase chain B | 0.87 | 1.36E-19 | 4.03E-16 |
| Tannerella | K05286 | PIGB; phosphatidylinositol glycan, class B [EC:2.4.1.-] | 0.87 | 1.58E-19 | 4.40E-16 |
| Rothia | K16014 | cydCD; ATP-binding cassette, subfamily C, bacterial CydCD | 0.87 | 2.12E-19 | 5.58E-16 |
| Rothia | K00153 | E1.1.1.306; S-(hydroxymethyl)mycothiol dehydrogenase [EC:1.1.1.306] | 0.87 | 2.83E-19 | 6.23E-16 |
| Campylobacter | K15910 | pglE; UDP-N-acetylbacillosamine transaminase [EC:2.6.1.34] | 0.87 | 2.89E-19 | 6.23E-16 |
| Campylobacter | K15912 | pglF; UDP-N-acetyl-D-glucosamine 4,6-dehydratase [EC:4.2.1.135] | 0.87 | 2.89E-19 | 6.23E-16 |
| Campylobacter | K15914 | pglA; N,N'-diacetylbacillosaminyl-diphospho-undecaprenol alpha-1,3-N-acetylgalactosaminyltransferase [EC:2.4.1.290] | 0.87 | 2.89E-19 | 6.23E-16 |
| Neisseria | K11107 | yfaE; ferredoxin | 0.87 | 3.88E-19 | 8.01E-16 |
| Haemophilus | K07125 | K07125; uncharacterized protein | 0.87 | 4.72E-19 | 9.33E-16 |
| Campylobacter | K17249 | pglH; GalNAc-alpha-(1->4)-GalNAc-alpha-(1->3)-diNAcBac-PP-undecaprenol alpha-1,4-N-acetyl-D-galactosaminyltransferase [EC:2.4.1.292] | 0.87 | 4.98E-19 | 9.44E-16 |
| Haemophilus | K02681 | ppdC; prepilin peptidase dependent protein C | 0.87 | 5.42E-19 | 9.88E-16 |
| Neisseria | K02839 | prfH; peptide chain release factor | 0.87 | 6.31E-19 | 1.11E-15 |

| | | | | | |
|---|---|---|---|---|---|
| Campylobacter | K16294 | psrC; polysulfide reductase chain C | 0.87 | 7.26E-19 | 1.19E-15 |
| Haemophilus | K02679 | ppdA; prepilin peptidase dependent protein A | 0.87 | 7.20E-19 | 1.19E-15 |
| Haemophilus | K03674 | grxA; glutaredoxin 1 | 0.87 | 8.02E-19 | 1.22E-15 |
| Haemophilus | K09896 | K09896; uncharacterized protein | 0.87 | 8.22E-19 | 1.22E-15 |
| Haemophilus | K09910 | K09910; uncharacterized protein | 0.87 | 7.84E-19 | 1.22E-15 |
| Haemophilus | K09911 | K09911; uncharacterized protein | 0.87 | 8.87E-19 | 1.28E-15 |
| Rothia | K16234 | hutT; histidine transporter | 0.86 | 1.50E-18 | 2.08E-15 |
| Rothia | K17883 | mtr; mycothione reductase [EC:1.8.1.15] | 0.86 | 1.53E-18 | 2.08E-15 |
| Campylobacter | K17251 | pglB; undecaprenyl-diphosphooligosaccharide---protein glycotransferase [EC:2.4.99.19] | 0.86 | 2.35E-18 | 3.10E-15 |
| Neisseria | K19339 | nosR; NosR/NirI family transcriptional regulator, nitrous oxide reductase regulator | 0.86 | 3.62E-18 | 4.64E-15 |
| Rothia | K00216 | entA; 2,3-dihydro-2,3-dihydroxybenzoate dehydrogenase [EC:1.3.1.28] | 0.86 | 4.49E-18 | 5.61E-15 |
| Rothia | K13671 | K13671; alpha-1,2-mannosyltransferase [EC:2.4.1.-] | 0.86 | 4.95E-18 | 6.02E-15 |
| Rothia | K01252 | entB, dhbB, vibB, mxcF; bifunctional isochorismate lyase / aryl carrier protein [EC:3.3.2.1 6.3.2.14] | 0.86 | 5.40E-18 | 6.40E-15 |
| Rothia | K15521 | mshA; D-inositol-3-phosphate glycosyltransferase [EC:2.4.1.250] | 0.85 | 7.58E-18 | 8.77E-15 |
| Rothia | K02363 | entE, dhbE, vibE, mxcE; 2,3-dihydroxybenzoate-AMP ligase [EC:6.3.2.14 2.7.7.58] | 0.85 | 1.04E-17 | 1.15E-14 |
| Neisseria | K08324 | sad; succinate-semialdehyde dehydrogenase [EC:1.2.1.16 1.2.1.24] | 0.85 | 1.02E-17 | 1.15E-14 |
| Haemophilus | K09906 | epmC; elongation factor P hydroxylase [EC:1.14.-.-] | 0.85 | 1.10E-17 | 1.19E-14 |
| Tannerella | K15896 | pseH; UDP-4-amino-4,6-dideoxy-N-acetyl-beta-L-altrosamine N-acetyltransferase [EC:2.3.1.202] | 0.85 | 1.60E-17 | 1.69E-14 |
| Neisseria | K00484 | hpaC; flavin reductase (NADH) [EC:1.5.1.36] | 0.85 | 1.97E-17 | 2.03E-14 |
| Neisseria | K11089 | TROVE2, SSA2; 60 kDa SS-A/Ro ribonucleoprotein | 0.85 | 2.43E-17 | 2.45E-14 |
| Neisseria | K11904 | vgrG; type VI secretion system secreted protein VgrG | 0.85 | 2.91E-17 | 2.87E-14 |
| Tannerella | K18574 | ptpA; Xaa-Xaa-Pro tripeptidyl-peptidase [EC:3.4.14.12] | 0.85 | 3.06E-17 | 2.96E-14 |
| Neisseria | K09890 | arfA; alternative ribosome-rescue factor | 0.84 | 4.01E-17 | 3.80E-14 |
| Neisseria | K07131 | K07131; uncharacterized protein | 0.84 | 4.22E-17 | 3.93E-14 |
| Neisseria | K12500 | tesC; thioesterase III [EC:3.1.2.-] | 0.84 | 4.33E-17 | 3.95E-14 |
| Rothia | K18455 | mca; mycothiol S-conjugate amidase [EC:3.5.1.115] | 0.84 | 4.68E-17 | 4.19E-14 |
| Rothia | K15526 | mshC; L-cysteine:1D-myo-inositol 2-amino-2-deoxy-alpha-D-glucopyranoside ligase [EC:6.3.1.13] | 0.84 | 5.32E-17 | 4.67E-14 |
| Neisseria | K16088 | TC.FEV.OM1, fhuE, fpvA, fptA; outer-membrane receptor for ferric coprogen and ferric-rhodotorulic acid | 0.84 | 6.16E-17 | 5.31E-14 |
| Rothia | K15520 | mshD; mycothiol synthase [EC:2.3.1.189] | 0.84 | 6.77E-17 | 5.73E-14 |
| Rothia | K03890 | qcrA; ubiquinol-cytochrome c reductase iron-sulfur subunit | 0.84 | 6.94E-17 | 5.77E-14 |
| Rothia | K03891 | qcrB; ubiquinol-cytochrome c reductase cytochrome b subunit | 0.84 | 8.23E-17 | 6.73E-14 |
| Tannerella | K18119 | sucD; succinate-semialdehyde dehydrogenase [EC:1.2.1.76] | 0.84 | 9.19E-17 | 7.39E-14 |

| | | | | | |
|---|---|---|---|---|---|
| Neisseria | K11910 | vasJ; type VI secretion system protein VasJ | 0.84 | 9.41E-17 | 7.44E-14 |
| Rothia | K03889 | qcrC; ubiquinol-cytochrome c reductase cytochrome c subunit | 0.84 | 1.08E-16 | 8.36E-14 |
| Neisseria | K11905 | K11905; type VI secretion system protein | 0.84 | 1.47E-16 | 1.12E-13 |
| Neisseria | K18991 | mtrA; AraC family transcriptional regulator, activator of mtrCDE | 0.84 | 1.55E-16 | 1.17E-13 |
| Neisseria | K02655 | pilE; type IV pilus assembly protein PilE | 0.84 | 1.69E-16 | 1.23E-13 |
| Neisseria | K08159 | sotB; MFS transporter, DHA1 family, L-arabinose/isopropyl-beta-D-thiogalactopyranoside export protein | 0.84 | 1.69E-16 | 1.23E-13 |
| Neisseria | K10679 | nfnB, nfsB; nitroreductase / dihydropteridine reductase [EC:1.-.-.- 1.5.1.34] | 0.83 | 2.25E-16 | 1.62E-13 |
| Rothia | K09019 | rutE; 3-hydroxypropanoate dehydrogenase [EC:1.1.1.-] | 0.83 | 5.27E-16 | 3.73E-13 |
| Neisseria | K14414 | rtcR; transcriptional regulatory protein RtcR | 0.83 | 5.50E-16 | 3.84E-13 |
| Neisseria | K03777 | dld; D-lactate dehydrogenase (quinone) [EC:1.1.5.12] | 0.83 | 6.83E-16 | 4.70E-13 |
| Neisseria | K10010 | ABC.CYST.A; cystine transport system ATP-binding protein [EC:3.6.3.-] | 0.83 | 8.71E-16 | 5.90E-13 |
| Tannerella | K18120 | 4hbD, abfH; 4-hydroxybutyrate dehydrogenase [EC:1.1.1.61] | 0.82 | 1.09E-15 | 7.29E-13 |
| Neisseria | K15974 | emrR, mprA; MarR family transcriptional regulator, negative regulator of the multidrug operon emrRAB | 0.82 | 1.53E-15 | 1.01E-12 |
| Rothia | K16650 | glft2; galactofuranosylgalactofuranosylrhamnosyl-N-acetylglucosaminyl-diphospho-decaprenol beta-1,5/1,6-galactofuranosyltransferase [EC:2.4.1.288] | 0.82 | 1.63E-15 | 1.05E-12 |
| Neisseria | K09977 | K09977; uncharacterized protein | 0.82 | 1.64E-15 | 1.05E-12 |
| Capnocytophaga | K12585 | DIS3, RRP44; exosome complex exonuclease DIS3/RRP44 [EC:3.1.13.-] | 0.82 | 1.98E-15 | 1.25E-12 |
| Rothia | K11602 | mntB; manganese transport system permease protein | 0.82 | 2.08E-15 | 1.30E-12 |
| Neisseria | K05952 | K05952; uncharacterized protein | 0.82 | 2.12E-15 | 1.30E-12 |
| Neisseria | K09929 | K09929; uncharacterized protein | 0.82 | 2.38E-15 | 1.45E-12 |
| Fusobacterium | K01121 | CNP; 2',3'-cyclic-nucleotide 3'-phosphodiesterase [EC:3.1.4.37] | 0.82 | 2.51E-15 | 1.51E-12 |
| Neisseria | K03835 | mtr; tryptophan-specific transport protein | 0.82 | 2.56E-15 | 1.52E-12 |
| Fusobacterium | K00870 | E2.7.1.37; protein kinase [EC:2.7.1.37] | 0.82 | 2.66E-15 | 1.56E-12 |
| Neisseria | K06020 | E3.6.3.25; sulfate-transporting ATPase [EC:3.6.3.25] | 0.82 | 3.31E-15 | 1.92E-12 |
| Rothia | K11603 | mntA; manganese transport system ATP-binding protein | 0.82 | 3.37E-15 | 1.92E-12 |
| Tannerella | K18428 | hpdC; 4-hydroxyphenylacetate decarboxylase small subunit [EC:4.1.1.83] | 0.82 | 3.49E-15 | 1.97E-12 |
| Neisseria | K07645 | qseC; two-component system, OmpR family, sensor histidine kinase QseC [EC:2.7.13.3] | 0.82 | 3.60E-15 | 2.01E-12 |
| Tannerella | K08693 | yfkN; 2',3'-cyclic-nucleotide 2'-phosphodiesterase / 3'-nucleotidase / 5'-nucleotidase [EC:3.1.4.16 3.1.3.6 3.1.3.5] | 0.82 | 3.75E-15 | 2.07E-12 |
| Haemophilus | K11258 | ilvM; acetolactate synthase II small subunit [EC:2.2.1.6] | 0.81 | 4.27E-15 | 2.33E-12 |
| Neisseria | K07248 | aldA; lactaldehyde dehydrogenase / glycolaldehyde dehydrogenase [EC:1.2.1.22 1.2.1.21] | 0.81 | 4.37E-15 | 2.36E-12 |
| Porphyromonas | K08589 | rgpA_B; gingipain R [EC:3.4.22.37] | 0.81 | 4.97E-15 | 2.65E-12 |
| Neisseria | K00813 | aspC; aspartate aminotransferase [EC:2.6.1.1] | 0.81 | 6.69E-15 | 3.52E-12 |

| | | | | | |
|---|---|---|---|---|---|
| Neisseria | K11907 | vasG, clpV; type VI secretion system protein VasG | 0.81 | 7.51E-15 | 3.91E-12 |
| Campylobacter | K05922 | hydB; quinone-reactive Ni/Fe-hydrogenase large subunit [EC:1.12.5.1] | 0.81 | 1.04E-14 | 5.36E-12 |
| Campylobacter | K08077 | NUDT14; UDP-sugar diphosphatase [EC:3.6.1.45] | 0.81 | 1.16E-14 | 5.85E-12 |
| Campylobacter | K18284 | K18284; adenosylhomocysteine/aminodeoxyfutalosine nucleosidase [EC:3.2.2.9 3.2.2.30] | 0.81 | 1.16E-14 | 5.85E-12 |
| Neisseria | K11901 | impB; type VI secretion system protein ImpB | 0.81 | 1.22E-14 | 6.08E-12 |
| Neisseria | K11895 | impH, vasB; type VI secretion system protein ImpH | 0.81 | 1.41E-14 | 6.97E-12 |
| Neisseria | K08082 | algZ; two-component system, LytTR family, sensor histidine kinase AlgZ [EC:2.7.13.3] | 0.81 | 1.52E-14 | 7.23E-12 |
| Neisseria | K11893 | impJ, vasE; type VI secretion system protein ImpJ | 0.81 | 1.49E-14 | 7.23E-12 |
| Parvimonas | K05299 | fdhA; formate dehydrogenase (NADP+) alpha subunit [EC:1.17.1.10] | 0.81 | 1.52E-14 | 7.23E-12 |
| Peptococcus | K06027 | NSF, SEC18; vesicle-fusing ATPase [EC:3.6.4.6] | 0.81 | 1.52E-14 | 7.23E-12 |
| Rothia | K13950 | pabAB; para-aminobenzoate synthetase [EC:2.6.1.85] | 0.80 | 1.57E-14 | 7.38E-12 |
| Fusobacterium | K19268 | glmE, mutE, mamB; methylaspartate mutase epsilon subunit [EC:5.4.99.1] | 0.80 | 2.23E-14 | 1.04E-11 |
| Neisseria | K11900 | impC; type VI secretion system protein ImpC | 0.80 | 2.27E-14 | 1.04E-11 |
| Neisseria | K16868 | tehB; tellurite methyltransferase [EC:2.1.1.265] | 0.80 | 2.54E-14 | 1.16E-11 |
| Neisseria | K07673 | narX; two-component system, NarL family, nitrate/nitrite sensor histidine kinase NarX [EC:2.7.13.3] | 0.80 | 2.74E-14 | 1.24E-11 |
| Neisseria | K11896 | impG, vasA; type VI secretion system protein ImpG | 0.80 | 3.02E-14 | 1.35E-11 |
| Neisseria | K03181 | ubiC; chorismate--pyruvate lyase [EC:4.1.3.40] | 0.80 | 3.40E-14 | 1.51E-11 |
| Neisseria | K07215 | pigA, hemO; heme oxygenase (biliverdin-IX-beta and delta-forming) [EC:1.14.99.58] | 0.80 | 3.61E-14 | 1.59E-11 |
| Parvimonas | K19084 | braE, bceB; bacitracin transport system permease protein | 0.80 | 4.02E-14 | 1.75E-11 |
| Neisseria | K07074 | K07074; uncharacterized protein | 0.80 | 4.22E-14 | 1.82E-11 |
| Tannerella | K07480 | insB; insertion element IS1 protein InsB | 0.80 | 4.96E-14 | 2.12E-11 |
| Neisseria | K09941 | K09941; uncharacterized protein | 0.80 | 5.01E-14 | 2.12E-11 |
| Fusobacterium | K03653 | K03653; N-glycosylase/DNA lyase [EC:3.2.2.- 4.2.99.18] | 0.80 | 5.52E-14 | 2.31E-11 |

positive, rho > 0.8 negative, rho < -0.8 p_adj<=0.05

19.2 Family

| Family | p2KO | description | Spearman_rho | p_val | p_adj |
|---|---|---|---|---|---|
| Neisseriaceae | K18133 | K18133, porB; major outer membrane protein P.IB | 0.93 | 1.21E-25 | 3.75E-21 |
| Neisseriaceae | K01947 | birA-coaX; biotin---[acetyl-CoA-carboxylase] ligase / type III pantothenate kinase [EC:6.3.4.15 2.7.1.33] | 0.92 | 5.96E-25 | 9.25E-21 |
| Neisseriaceae | K07280 | K07280; outer membrane protein | 0.92 | 1.36E-24 | 1.40E-20 |
| Neisseriaceae | K00025 | MDH1; malate dehydrogenase [EC:1.1.1.37] | 0.92 | 2.16E-24 | 1.67E-20 |
| Neisseriaceae | K12875 | ACIN1, ACINUS; apoptotic chromatin condensation inducer in the nucleus | 0.89 | 3.14E-21 | 1.94E-17 |
| Neisseriaceae | K03675 | grxB; glutaredoxin 2 | 0.88 | 2.78E-20 | 1.44E-16 |
| Neisseriaceae | K16346 | xanQ; xanthine permease XanQ | 0.88 | 3.81E-20 | 1.69E-16 |
| Micrococcaceae | K03343 | puo; putrescine oxidase [EC:1.4.3.10] | 0.87 | 4.43E-19 | 1.72E-15 |
| Neisseriaceae | K18148 | rtcB; release factor H-coupled RctB family protein | 0.87 | 5.42E-19 | 1.87E-15 |
| Campylobacteraceae | K16293 | psrB; polysulfide reductase chain B | 0.87 | 9.90E-19 | 3.07E-15 |
| Campylobacteraceae | K15910 | pglE; UDP-N-acetylbacillosamine transaminase [EC:2.6.1.34] | 0.86 | 1.48E-18 | 3.54E-15 |
| Campylobacteraceae | K15912 | pglF; UDP-N-acetyl-D-glucosamine 4,6-dehydratase [EC:4.2.1.135] | 0.86 | 1.48E-18 | 3.54E-15 |
| Campylobacteraceae | K15914 | pglA; N,N'-diacetylbacillosaminyl-diphospho-undecaprenol alpha-1,3-N-acetylgalactosaminyltransferase [EC:2.4.1.290] | 0.86 | 1.48E-18 | 3.54E-15 |
| Campylobacteraceae | K17249 | pglH; GalNAc-alpha-(1->4)-GalNAc-alpha-(1->3)-diNAcBac-PP-undecaprenol alpha-1,4-N-acetyl-D-galactosaminyltransferase [EC:2.4.1.292] | 0.86 | 3.18E-18 | 7.04E-15 |
| Campylobacteraceae | K16294 | psrC; polysulfide reductase chain C | 0.86 | 4.35E-18 | 8.98E-15 |
| Bacteroidaceae | K18348 | vanT; serine/alanine racemase [EC:5.1.1.18 5.1.1.1] | 0.86 | 4.88E-18 | 9.47E-15 |
| Neisseriaceae | K02839 | prfH; peptide chain release factor | 0.85 | 8.72E-18 | 1.59E-14 |
| Micrococcaceae | K15733 | E1.11.1.19; dye decolorizing peroxidase [EC:1.11.1.19] | 0.85 | 1.05E-17 | 1.72E-14 |
| Micrococcaceae | K16014 | cydCD; ATP-binding cassette, subfamily C, bacterial CydCD | 0.85 | 1.01E-17 | 1.72E-14 |
| Micrococcaceae | K16234 | hutT; histidine transporter | 0.85 | 1.27E-17 | 1.98E-14 |
| Campylobacteraceae | K17251 | pglB; undecaprenyl-diphosphooligosaccharide---protein glycotransferase [EC:2.4.99.19] | 0.85 | 1.55E-17 | 2.29E-14 |
| Neisseriaceae | K11107 | yfaE; ferredoxin | 0.85 | 1.80E-17 | 2.54E-14 |
| Micrococcaceae | K00153 | E1.1.1.306; S-(hydroxymethyl)mycothiol dehydrogenase [EC:1.1.1.306] | 0.85 | 2.20E-17 | 2.96E-14 |
| Micrococcaceae | K15525 | mshB; N-acetyl-1-D-myo-inositol-2-amino-2-deoxy-alpha-D-glucopyranoside deacetylase [EC:3.5.1.103] | 0.84 | 4.88E-17 | 6.31E-14 |
| Micrococcaceae | K00216 | entA; 2,3-dihydro-2,3-dihydroxybenzoate dehydrogenase [EC:1.3.1.28] | 0.84 | 5.37E-17 | 6.66E-14 |
| Micrococcaceae | K01252 | entB, dhbB, vibB, mxcF; bifunctional isochorismate lyase / aryl carrier protein [EC:3.3.2.1 6.3.2.14] | 0.84 | 5.81E-17 | 6.93E-14 |
| Micrococcaceae | K02363 | entE, dhbE, vibE, mxcE; 2,3-dihydroxybenzoate-AMP ligase [EC:6.3.2.14 2.7.7.58] | 0.84 | 6.16E-17 | 7.07E-14 |
| Micrococcaceae | K02364 | entF; enterobactin synthetase component F [EC:6.3.2.14] | 0.84 | 1.08E-16 | 1.19E-13 |
| Micrococcaceae | K17883 | mtr; mycothione reductase [EC:1.8.1.15] | 0.84 | 1.13E-16 | 1.21E-13 |

| | | | | | |
|---|---|---|---|---|---|
| Neisseriaceae | K19339 | nosR; NosR/NirI family transcriptional regulator, nitrous oxide reductase regulator | 0.83 | 3.25E-16 | 3.36E-13 |
| Tannerellaceae | K18120 | 4hbD, abfH; 4-hydroxybutyrate dehydrogenase [EC:1.1.1.61] | 0.83 | 3.48E-16 | 3.48E-13 |
| Neisseriaceae | K08324 | sad; succinate-semialdehyde dehydrogenase [EC:1.2.1.16 1.2.1.24] | 0.83 | 4.07E-16 | 3.94E-13 |
| Neisseriaceae | K12500 | tesC; thioesterase III [EC:3.1.2.-] | 0.83 | 6.47E-16 | 6.08E-13 |
| Neisseriaceae | K00484 | hpaC; flavin reductase (NADH) [EC:1.5.1.36] | 0.83 | 6.74E-16 | 6.15E-13 |
| Neisseriaceae | K07131 | K07131; uncharacterized protein | 0.82 | 9.76E-16 | 8.64E-13 |
| Pasteurellaceae | K02681 | ppdC; prepilin peptidase dependent protein C | 0.82 | 1.02E-15 | 8.77E-13 |
| Pasteurellaceae | K02679 | ppdA; prepilin peptidase dependent protein A | 0.82 | 1.41E-15 | 1.18E-12 |
| Tannerellaceae | K15896 | pseH; UDP-4-amino-4,6-dideoxy-N-acetyl-beta-L-altrosamine N-acetyltransferase [EC:2.3.1.202] | 0.82 | 1.62E-15 | 1.32E-12 |
| Tannerellaceae | K14534 | abfD; 4-hydroxybutyryl-CoA dehydratase / vinylacetyl-CoA-Delta-isomerase [EC:4.2.1.120 5.3.3.3] | 0.82 | 1.87E-15 | 1.49E-12 |
| Neisseriaceae | K16088 | TC.FEV.OM1, fhuE, fpvA, fptA; outer-membrane receptor for ferric coprogen and ferric-rhodotorulic acid | 0.82 | 2.05E-15 | 1.59E-12 |
| Neisseriaceae | K11089 | TROVE2, SSA2; 60 kDa SS-A/Ro ribonucleoprotein | 0.82 | 2.16E-15 | 1.63E-12 |
| Micrococcaceae | K18455 | mca; mycothiol S-conjugate amidase [EC:3.5.1.115] | 0.82 | 2.59E-15 | 1.91E-12 |
| Micrococcaceae | K15526 | mshC; L-cysteine:1D-myo-inositol 2-amino-2-deoxy-alpha-D-glucopyranoside ligase [EC:6.3.1.13] | 0.82 | 2.98E-15 | 2.10E-12 |
| Pasteurellaceae | K09911 | K09911; uncharacterized protein | 0.82 | 2.95E-15 | 2.10E-12 |
| Neisseriaceae | K09890 | arfA; alternative ribosome-rescue factor | 0.82 | 3.19E-15 | 2.20E-12 |
| Micrococcaceae | K15521 | mshA; D-inositol-3-phosphate glycosyltransferase [EC:2.4.1.250] | 0.82 | 3.27E-15 | 2.20E-12 |
| Micrococcaceae | K15520 | mshD; mycothiol synthase [EC:2.3.1.189] | 0.82 | 3.62E-15 | 2.39E-12 |
| Micrococcaceae | K03890 | qcrA; ubiquinol-cytochrome c reductase iron-sulfur subunit | 0.82 | 3.91E-15 | 2.48E-12 |
| Peptococcaceae | K06027 | NSF, SEC18; vesicle-fusing ATPase [EC:3.6.4.6] | 0.82 | 3.86E-15 | 2.48E-12 |
| Neisseriaceae | K11904 | vgrG; type VI secretion system secreted protein VgrG | 0.81 | 4.13E-15 | 2.56E-12 |
| Micrococcaceae | K03891 | qcrB; ubiquinol-cytochrome c reductase cytochrome b subunit | 0.81 | 4.52E-15 | 2.75E-12 |
| Pasteurellaceae | K09896 | K09896; uncharacterized protein | 0.81 | 4.62E-15 | 2.76E-12 |
| Pasteurellaceae | K09910 | K09910; uncharacterized protein | 0.81 | 5.19E-15 | 3.03E-12 |
| Micrococcaceae | K03889 | qcrC; ubiquinol-cytochrome c reductase cytochrome c subunit | 0.81 | 5.44E-15 | 3.12E-12 |
| Micrococcaceae | K13671 | K13671; alpha-1,2-mannosyltransferase [EC:2.4.1.-] | 0.81 | 5.62E-15 | 3.17E-12 |
| Pasteurellaceae | K03674 | grxA; glutaredoxin 1 | 0.81 | 5.76E-15 | 3.19E-12 |
| Tannerellaceae | K15899 | pseF; pseudaminic acid cytidylyltransferase [EC:2.7.7.81] | 0.81 | 6.03E-15 | 3.28E-12 |
| Neisseriaceae | K08159 | sotB; MFS transporter, DHA1 family, L-arabinose/isopropyl-beta-D-thiogalactopyranoside export protein | 0.81 | 6.58E-15 | 3.52E-12 |
| Neisseriaceae | K02655 | pilE; type IV pilus assembly protein PilE | 0.81 | 6.86E-15 | 3.61E-12 |
| Neisseriaceae | K10679 | nfnB, nfsB; nitroreductase / dihydropteridine reductase [EC:1.-.-.- 1.5.1.34] | 0.81 | 7.25E-15 | 3.75E-12 |
| Neisseriaceae | K18991 | mtrA; AraC family transcriptional regulator, activator of mtrCDE | 0.81 | 8.04E-15 | 4.09E-12 |

| | | | | | |
|---|---|---|---|---|---|
| Tannerellaceae | K15898 | pseI, neuB3; pseudaminic acid synthase [EC:2.5.1.97] | 0.81 | 1.21E-14 | 6.06E-12 |
| Neisseriaceae | K11910 | vasJ; type VI secretion system protein VasJ | 0.81 | 1.26E-14 | 6.19E-12 |
| Neisseriaceae | K14414 | rtcR; transcriptional regulatory protein RtcR | 0.80 | 1.56E-14 | 7.58E-12 |
| Neisseriaceae | K11905 | K11905; type VI secretion system protein | 0.80 | 1.85E-14 | 8.81E-12 |
| Neisseriaceae | K03835 | mtr; tryptophan-specific transport protein | 0.80 | 2.48E-14 | 1.17E-11 |
| Neisseriaceae | K07248 | aldA; lactaldehyde dehydrogenase / glycolaldehyde dehydrogenase [EC:1.2.1.22 1.2.1.21] | 0.80 | 2.99E-14 | 1.39E-11 |
| Neisseriaceae | K03777 | dld; D-lactate dehydrogenase (quinone) [EC:1.1.5.12] | 0.80 | 3.06E-14 | 1.39E-11 |
| Neisseriaceae | K05952 | K05952; uncharacterized protein | 0.80 | 3.88E-14 | 1.74E-11 |
| Flavobacteriaceae | K15269 | pecM; probable blue pigment (indigoidine) exporter | 0.80 | 4.12E-14 | 1.82E-11 |
| Neisseriaceae | K06020 | E3.6.3.25; sulfate-transporting ATPase [EC:3.6.3.25] | 0.80 | 4.87E-14 | 2.13E-11 |
| Neisseriaceae | K15974 | emrR, mprA; MarR family transcriptional regulator, negative regulator of the multidrug operon emrRAB | 0.80 | 5.29E-14 | 2.28E-11 |

positive, rho > 0.8 negative, rho < -0.8 p_adj<=0.05

**Table 20. Subject information of tissue for scRNA-seq**

| Subj. ID | Sample | Age | BMI | Obese/ Overweight | Full Diagnosis | Smoker | DM | FMPS (%) | FMBS (%) | PD (mm) | CAL (mm) | PD≥4mm (%) | PD≥6mm (%) | M≥2 (%) | FI≥2 (%) |
|---|---|---|---|---|---|---|---|---|---|---|---|---|---|---|---|
| **P010** | PT | 41 | 24.1 | Yes | Generalised Stage III Grade C | Never | No | 41 | 57 | 3.04 | 3.23 | 0.16 | 0.05 | 0 | 0 |
| **P011** | ST | 48 | 25.4 | Yes | Generalised Stage III Grade C | Former (6 yr cessation) | No | 83 | 88 | 4.97 | 5.19 | 0.4 | 0.25 | 0.19 | 0.26 |
| **P020** | GT | 47 | 29.3 | Yes | Generalised Stage IV Grade C | Never | No | 94 | 99 | 7.54 | 7.59 | 0.85 | 0.64 | 0.31 | 0.24 |

**Table 21. Quality control of single-cell preparation**

| Tissue ID | Conc. of viable cells (cells/µL) | Viability (%) | Total no. of viable cells (cells) |
|---|---|---|---|
| P020-GT | 512 | 87 | >20,000 |
| P010-PT | 1,272 | 97 | >20,000 |
| P011-37ST | 1,080 | 97 | >20,000 |

**Table 22. Basic statistics of sequencing data**

| sample_name | number_of _reads | mean_reads _per_cell | valid_barcodes | q30_bases _in_barcode | q30_bases _in_rna_read | q30_bases _in_umi |
|---|---|---|---|---|---|---|
| **GT** | 409,068,196 | 50,502 | 96.50% | 93.00% | 94.50% | 92.10% |
| **PT** | 346,254,096 | 86,563 | 97.10% | 91.40% | 90.10% | 90.70% |
| **ST** | 351,452,734 | 87,863 | 97.40% | 91.40% | 90.30% | 90.70% |

number_of_reads: Total reads number
mean_reads_per_cell: Average number of sequencing reads per cell
valid_barcodes: The proportion of barcode in Reads in the known dataset
q30_bases_in_barcode: The ratio of bases with barcode sequencing quality above Q30
q30_bases_in_rna_read: The ratio of bases with an RNA fragment inserted above Q30
q30_bases_in umi: Base ratio of UMI sequencing quality above Q30.

**Table 23. Statistics of alignment**

| Sample _name | reads_mapped _confidently_to _transcriptome | reads_mapped _confidently_to _exonic_regions | reads_mapped _confidently_to _intronic_regions | reads_mapped _confidently_to _intergenic_regions | Sequencing _saturation |
|---|---|---|---|---|---|
| **GT** | 50.80% | 54.60% | 28.50% | 10.70% | 57.80% |
| **PT** | 46.60% | 50.00% | 34.90% | 9.10% | 93.60% |
| **ST** | 48.40% | 51.60% | 34.00% | 8.10% | 96.30% |

reads_mapped_confidently_to_transcriptome: The mapped reads ratio to the transcriptome
reads_mapped_confidently_to_exonic_regions: The mapped reads ratio to the exon region
reads_mapped_confidently_to_intronic_regions: The mapped reads ratio to the intron region
reads_mapped_confidently_to_intergenic_regions: The mapped reads ratio to the intergenic region
sequencing_saturation: Sequencing saturation estimation

**Table 24. Statistics of library**

| sample_name | estimated _number _of_cells | fraction _reads _in_cells | mean_reads _per_cell | median_genes _per_cell | total_genes _detected | median_umi _count_per_cell |
|---|---|---|---|---|---|---|
| **GT** | 8,100 | 90.10% | 50502 | 1664 | 28554 | 4638 |
| **PT** | 4,000 | 91.20% | 86563 | 55 | 23364 | 249 |
| **ST** | 4,000 | 93.10% | 87863 | 19 | 21666 | 35 |

estimated_number_of_cells: Cell number estimation

fraction_reads_in_cells: Proportion of reads in cells
mean_reads_per_cell: Average number of reads per cell
median_genes_per_cell: Median number of genes detected in each cell
total_genes_detected: Total number of genes detected
median_umi_counts_per_cell: Median number of UMI detected in each cell

**Table 25. KEGG enrichment of scRNA-seq for each type of tissue**

p.adjust < 0.05

| Cluster | ID | Description | Gene Ratio | Bg Ratio | pvalue | p.adjust | qvalue | geneID | Count |
|---|---|---|---|---|---|---|---|---|---|
| GT | hsa04820 | Cytoskeleton in muscle cells | 54/615 | 229/8773 | 6.52E-16 | 1.98E-13 | 1.57E-13 | ANK2/BGN/COL11A1/COL1A1/COL1A2/COL3A1/COL4A1/COL4A2/COL5A1/COL5A2/COL5A3/COL6A1/COL6A2/COL6A3/CSRP2/DCN/DMD/DSC2/DSP/FBN1/FHL1/FN1/HSPG2/ITGA1/ITGA2/ITGA6/ITGAV/ITGB1/ITGB4/ITGB5/ITGB8/JUP/LMNA/MYH11/MYL9/NID1/NID2/PDLIM1/PDLIM4/SDC1/SDC2/SDC3/SDC4/SGCB/SPTBN1/SSPN/SYNPO2/THBS1/THBS2/TPM1/TPM2/VCAN/VIM/XIRP1 | 54 |
| GT | hsa04512 | ECM-receptor interaction | 32/615 | 89/8773 | 1.84E-15 | 2.78E-13 | 2.20E-13 | CD36/COL1A1/COL1A2/COL4A1/COL4A2/COL6A1/COL6A2/COL6A3/FN1/HMMR/HSPG2/ITGA1/ITGA2/ITGA6/ITGAV/ITGB1/ITGB4/ITGB5/ITGB8/LAMA3/LAMA4/LAMB1/LAMB2/LAMB3/LAMC1/LAMC2/SDC1/SDC4/THBS1/THBS2/TNC/VWF | 32 |
| GT | hsa04510 | Focal adhesion | 47/615 | 202/8773 | 9.36E-14 | 9.45E-12 | 7.49E-12 | ACTN1/CAV2/CCND1/COL1A1/COL1A2/COL4A1/COL4A2/COL6A1/COL6A2/COL6A3/DOCK1/EGFR/EMP1/EMP2/FLT1/FN1/HGF/ITGA1/ITGA2/ITGA6/ITGAV/ITGB1/ITGB4/ITGB5/ITGB8/JUN/KDR/LAMA3/LAMA4/LAMB1/LAMB2/LAMB3/LAMC1/LAMC2/MYL9/MYLK/PARVA/PDGFA/PDGFD/PDGFRB/PGF/PTK2/THBS1/THBS2/TNC/VEGFA/VWF | 47 |
| GT | hsa04060 | Cytokine-cytokine receptor interaction | 57/615 | 297/8773 | 1.31E-12 | 9.90E-11 | 7.85E-11 | ACKR3/BMP2/BMP4/BMP8A/BMPR2/CCL14/CCL17/CCL19/CCL2/CCL20/CCL8/CD40/CSF3/CXCL1/CXCL10/CXCL12/CXCL13/CXCL14/CXCL17/CXCL2/CXCL3/CXCL6/CXCL9/EBI3/IL11/IL11RA/IL13/IL17A/IL17F/IL18/IL19/IL1A/IL1R1/IL1RL1/IL22/IL24/IL2RA/IL33/IL34/IL36A/IL36G/IL3RA/IL6/INHBA/LEPR/LIF/LIFR/OSMR/TGFB3/TGFBR2/TNFRSF11B/TNFRSF12A/TNFRSF1A/TNFRSF21/TNFRSF6B/TNFSF10/TNFSF15 | 57 |
| GT | hsa04610 | Complement and coagulation cascades | 27/615 | 86/8773 | 1.22E-11 | 7.42E-10 | 5.87E-10 | A2M/BDKRB2/C1QA/C1QB/C1QC/C1R/C1S/C3/CD59/CFB/CFD/CFH/CFI/CLU/F2R/F2RL2/F3/MASP1/PLAT/PLAU/SERPINB2/SERPINE1/SERPINE2/SERPING1/TFPI/THBD/VWF | 27 |

| | | | | | | | | | |
|---|---|---|---|---|---|---|---|---|---|
| GT | hsa05205 | Proteoglycans in cancer | 43/615 | 203/8773 | 3.10E-11 | 1.57E-09 | 1.24E-09 | ANK2/ARHGEF12/CAV2/CCND1/CD63/COL1A1/COL1A2/CTSL/CTTN/DCN/EGFR/FGFR1/FN1/FZD1/FZD4/FZD6/FZD7/HBEGF/HGF/HSPG2/IGF2/ITGA2/ITGAV/ITGB1/ITGB5/KDR/LUM/MMP2/MYC/PLAU/PTK2/RDX/SDC1/SDC2/SDC4/THBS1/TIMP3/TWIST1/TWIST2/VEGFA/WNT2/WNT4/WNT5A | 43 |
| GT | hsa05144 | Malaria | 20/615 | 50/8773 | 3.89E-11 | 1.69E-09 | 1.33E-09 | ACKR1/CCL2/CD36/CD40/CD81/CSF3/HBB/HGF/IL18/IL6/LRP1/PECAM1/SDC1/SDC2/SELE/SELP/TGFB3/THBS1/THBS2/VCAM1 | 20 |
| GT | hsa05418 | Fluid shear stress and atherosclerosis | 33/615 | 139/8773 | 2.87E-10 | 1.09E-08 | 8.61E-09 | BMP4/BMPR2/CALML3/CALML5/CAV2/CCL2/CDH5/CTSL/EDN1/HMOX1/HSP90B1/IL1A/IL1R1/ITGAV/JUN/KDR/MEF2C/MGST2/MMP2/NPPC/PDGFA/PECAM1/PLAT/PTK2/SDC1/SDC2/SDC4/SELE/THBD/TNFRSF1A/TXN/VCAM1/VEGFA | 33 |
| GT | hsa05146 | Amoebiasis | 27/615 | 102/8773 | 9.29E-10 | 3.13E-08 | 2.48E-08 | ACTN1/COL1A1/COL1A2/COL3A1/COL4A1/COL4A2/CXCL1/CXCL2/CXCL3/FN1/HSPB1/IL1R1/IL6/LAMA3/LAMA4/LAMB1/LAMB2/LAMB3/LAMC1/LAMC2/PLCB1/PTK2/RAB7B/SERPINB13/SERPINB3/SERPINB4/TGFB3 | 27 |
| GT | hsa04151 | PI3K-Akt signaling pathway | 58/615 | 359/8773 | 1.09E-09 | 3.31E-08 | 2.62E-08 | ANGPT2/CCND1/COL1A1/COL1A2/COL4A1/COL4A2/COL6A1/COL6A2/COL6A3/CSF3/EFNA1/EGFR/EPHA2/F2R/FGFR1/FLT1/FN1/GNG11/HGF/HSP90B1/IGF2/IL2RA/IL3RA/IL6/INSR/ITGA1/ITGA2/ITGA6/ITGAV/ITGB1/ITGB4/ITGB5/ITGB8/KDR/LAMA3/LAMA4/LAMB1/LAMB2/LAMB3/LAMC1/LAMC2/LPAR1/MYC/NR4A1/NTRK2/OSMR/PDGFA/PDGFD/PDGFRB/PGF/PTK2/SGK1/THBS1/THBS2/TNC/VEGFA/VWF/YWHAH | 58 |
| GT | hsa05165 | Human papillomavirus infection | 52/615 | 331/8773 | 2.22E-08 | 6.10E-07 | 4.83E-07 | CCND1/COL1A1/COL1A2/COL4A1/COL4A2/COL6A1/COL6A2/COL6A3/CRB3/EGFR/FN1/FZD1/FZD4/FZD6/FZD7/HES1/HES4/HES5/HEY1/ISG15/ITGA1/ITGA2/ITGA6/ITGAV/ITGB1/ITGB4/ITGB5/ITGB8/JAG1/LAMA3/LAMA4/LAMB1/LAMB2/LAMB3/LAMC1/LAMC2/NOTCH3/NOTCH4/PDGFRB/PKM/PTK2/TCF7L1/TCF7L2/THBS1/THBS2/TNC/TNFRSF1A/VEGFA/VWF/WNT2/WNT4/WNT5A | 52 |
| GT | hsa04061 | Viral protein interaction with cytokine and cytokine receptor | 24/615 | 100/8773 | 6.37E-08 | 1.61E-06 | 1.27E-06 | ACKR3/CCL14/CCL17/CCL19/CCL2/CCL20/CCL8/CXCL1/CXCL10/CXCL12/CXCL13/CXCL14/CXCL2/CXCL3/CXCL6/CXCL9/IL18/IL19/IL24/IL2RA/IL34/IL6/TNFRSF1A/TNFSF10 | 24 |
| GT | hsa04933 | AGE-RAGE signaling pathway | 23/615 | 100/8773 | 2.75E-07 | 6.42E-06 | 5.08E-06 | CCL2/CCND1/COL1A1/COL1A2/COL3A1/COL4A1/COL4A2/EDN1/EGR1/F3/FN1/IL1A/IL6/JUN/MMP2/PLCB1/SELE/SERPINE1/TGFB3/TGFBR2/THBD/VCAM1/VEGFA | 23 |

| | | | | | | | | | |
|---|---|---|---|---|---|---|---|---|---|
| | | in diabetic complications | | | | | | | |
| GT | hsa04350 | TGF-beta signaling pathway | 24/615 | 108/8773 | 3.01E-07 | 6.52E-06 | 5.16E-06 | BMP2/BMP4/BMP8A/BMPR2/CDKN2B/DCN/FBN1/FMOD/FST/GREM1/ID1/ID3/ID4/INHBA/LRRC32/LTBP1/MYC/NBL1/NEO1/PITX2/TGFB3/TGFBR2/THBS1/THSD4 | 24 |
| GT | hsa04514 | Cell adhesion molecules | 30/615 | 157/8773 | 3.55E-07 | 6.86E-06 | 5.43E-06 | ALCAM/CADM1/CD276/CD34/CD40/CDH1/CDH3/CDH5/CLDN1/CLDN4/CLDN5/CLDN7/ESAM/ITGA6/ITGAV/ITGB1/ITGB8/JAM2/NEO1/NRCAM/PECAM1/PTPRF/SDC1/SDC2/SDC3/SDC4/SELE/SELP/VCAM1/VCAN | 30 |
| GT | hsa04657 | IL-17 signaling pathway | 22/615 | 94/8773 | 3.62E-07 | 6.86E-06 | 5.43E-06 | CCL17/CCL2/CCL20/CEBPB/CSF3/CXCL1/CXCL10/CXCL2/CXCL3/CXCL6/FOSL1/HSP90B1/IL13/IL17A/IL17F/IL6/JUN/LCN2/MMP13/MMP3/S100A7/S100A7A | 22 |
| GT | hsa05150 | Staphylococcus aureus infection | 21/615 | 96/8773 | 2.17E-06 | 3.88E-05 | 3.07E-05 | C1QA/C1QB/C1QC/C1R/C1S/C3/CFB/CFD/CFH/CFI/DEFB1/DSG1/KRT13/KRT14/KRT15/KRT16/KRT17/KRT18/KRT19/MASP1/SELP | 21 |
| GT | hsa04390 | Hippo signaling pathway | 28/615 | 157/8773 | 3.64E-06 | 6.12E-05 | 4.85E-05 | BMP2/BMP4/BMP8A/BMPR2/CCN2/CCND1/CDH1/FRMD6/FZD1/FZD4/FZD6/FZD7/ID1/MYC/SAV1/SERPINE1/SNAI2/TCF7L1/TCF7L2/TEAD1/TGFB3/TGFBR2/WNT2/WNT4/WNT5A/WWTR1/YAP1/YWHAH | 28 |
| GT | hsa04974 | Protein digestion and absorption | 21/615 | 103/8773 | 7.11E-06 | 0.000113412 | 8.98E-05 | COL11A1/COL12A1/COL14A1/COL15A1/COL17A1/COL18A1/COL1A1/COL1A2/COL3A1/COL4A1/COL4A2/COL5A1/COL5A2/COL5A3/COL6A1/COL6A2/COL6A3/COL7A1/COL8A1/PRCP/PRSS3 | 21 |
| GT | hsa05222 | Small cell lung cancer | 19/615 | 92/8773 | 1.59E-05 | 0.000240369 | 0.000190391 | CCND1/CDKN2B/COL4A1/COL4A2/FN1/GADD45A/ITGA2/ITGA6/ITGAV/ITGB1/LAMA3/LAMA4/LAMB1/LAMB2/LAMB3/LAMC1/LAMC2/MYC/PTK2 | 19 |
| GT | hsa04270 | Vascular smooth muscle contraction | 24/615 | 134/8773 | 1.67E-05 | 0.000240369 | 0.000190391 | ACTA2/ADCY4/ADM/ARHGEF12/AVPR1A/CALCRL/CALML3/CALML5/EDN1/EDNRA/GUCY1A1/GUCY1A2/GUCY1B1/KCNMA1/MYH11/MYL9/MYLK/NPPC/PLCB1/PPP1R14A/PRKG1/RAMP1/RAMP2/RAMP3 | 24 |
| GT | hsa04520 | Adherens junction | 19/615 | 93/8773 | 1.87E-05 | 0.000246903 | 0.000195566 | ACTN1/ARHGAP29/CDH1/CDH5/CTNND1/EGFR/FGFR1/HEG1/INSR/LMO7/MYL9/NECTIN4/PTPRB/PTPRF/SNAI1/SNAI2/TCF7L1/TCF7L2/TGFBR2 | 19 |
| GT | hsa05323 | Rheumatoid arthritis | 19/615 | 93/8773 | 1.87E-05 | 0.000246903 | 0.000195566 | CCL2/CCL20/CTSK/CTSL/CXCL1/CXCL12/CXCL2/CXCL3/CXCL6/FLT1/IL11/IL17A/IL18/IL1A/IL6/JUN/MMP3/TGFB3/VEGFA | 19 |

| | | | | | | | | | |
|---|---|---|---|---|---|---|---|---|---|
| GT | hsa05412 | Arrhythmogenic right ventricular cardiomyopathy | 18/615 | 86/8773 | 2.19E-05 | 0.000276748 | 0.000219207 | DMD/DSC2/DSP/GJA1/ITGA1/ITGA2/ITGA6/ITGAV/ITGB1/ITGB4/ITGB5/ITGB8/JUP/LMNA/SGCB/SSPN/TCF7L1/TCF7L2 | 18 |
| GT | hsa04015 | Rap1 signaling pathway | 31/615 | 210/8773 | 5.73E-05 | 0.000694098 | 0.000549781 | ADCY4/ANGPT2/CALML3/CALML5/CDH1/CTNND1/DOCK4/EFNA1/EGFR/ENAH/EPHA2/F2R/FGFR1/FLT1/HGF/ID1/INSR/ITGB1/KDR/LPAR1/PDGFA/PDGFD/PDGFRB/PGF/PLCB1/RALA/RAPGEF3/RAPGEF5/RASGRP3/THBS1/VEGFA | 31 |
| GT | hsa05224 | Breast cancer | 24/615 | 147/8773 | 7.94E-05 | 0.000924958 | 0.00073264 | CCND1/DLL1/DLL4/EGFR/FGFR1/FZD1/FZD4/FZD6/FZD7/GADD45A/HES1/HES5/HEY1/JAG1/JAG2/JUN/MYC/NOTCH3/NOTCH4/TCF7L1/TCF7L2/WNT2/WNT4/WNT5A | 24 |
| GT | hsa04540 | Gap junction | 17/615 | 88/8773 | 0.000107073 | 0.001201594 | 0.000951758 | ADCY4/CDK1/EGFR/GJA1/GUCY1A1/GUCY1A2/GUCY1B1/LPAR1/PDGFA/PDGFD/PDGFRB/PLCB1/PRKG1/TUBB2A/TUBB2B/TUBB3/TUBB6 | 17 |
| GT | hsa05410 | Hypertrophic cardiomyopathy | 18/615 | 99/8773 | 0.000153068 | 0.001656414 | 0.001312011 | ACE/DMD/EDN1/IL6/ITGA1/ITGA2/ITGA6/ITGAV/ITGB1/ITGB4/ITGB5/ITGB8/LMNA/SGCB/SSPN/TGFB3/TPM1/TPM2 | 18 |
| GT | hsa04148 | Efferocytosis | 24/615 | 156/8773 | 0.000204836 | 0.002140179 | 0.001695191 | ABCA1/AXL/C1QA/C1QB/C1QC/CD24/CD36/CEBPB/CH25H/DOCK1/DUSP5/GAS6/ITGAV/ITGB5/LRP1/MFGE8/PBX1/PECAM1/PTK2/RAB7B/SGK1/SPHK1/STAB1/THBS1 | 24 |
| GT | hsa04924 | Renin secretion | 14/615 | 69/8773 | 0.000250756 | 0.002532636 | 0.002006048 | ACE/AQP1/CALML3/CALML5/CLCA2/CLCA4/CTSB/EDN1/EDNRA/GUCY1A1/GUCY1A2/GUCY1B1/KCNMA1/PLCB1 | 14 |
| GT | hsa04640 | Hematopoietic cell lineage | 17/615 | 99/8773 | 0.00046765 | 0.004570899 | 0.003620514 | ANPEP/CD24/CD34/CD36/CD59/CD9/CSF3/IL11/IL11RA/IL1A/IL1R1/IL2RA/IL3RA/IL6/ITGA1/ITGA2/ITGA6 | 17 |
| GT | hsa05133 | Pertussis | 14/615 | 76/8773 | 0.000706926 | 0.006693708 | 0.005301947 | C1QA/C1QB/C1QC/C1R/C1S/C3/CALML3/CALML5/CXCL6/IL1A/IL6/ITGB1/JUN/SERPING1 | 14 |
| GT | hsa04978 | Mineral absorption | 12/615 | 60/8773 | 0.000784546 | 0.007203558 | 0.005705788 | CYBRD1/HEPHL1/HMOX1/MT1A/MT1E/MT1G/MT1M/MT1X/MT2A/SLC30A1/SLC5A1/STEAP2 | 12 |
| GT | hsa04668 | TNF signaling pathway | 18/615 | 114/8773 | 0.000908577 | 0.008097022 | 0.006413483 | CCL2/CCL20/CEBPB/CXCL1/CXCL10/CXCL2/CXCL3/CXCL6/EDN1/IL6/JAG1/JUN/LIF/MMP3/SELE/SOCS3/TNFRSF1A/VCAM1 | 18 |
| GT | hsa04550 | Signaling pathways regulating pluripotency of stem cells | 21/615 | 143/8773 | 0.000938687 | 0.008126344 | 0.006436708 | BMP4/BMPR2/DLX5/FGFR1/FZD1/FZD4/FZD6/FZD7/ID1/ID3/ID4/INHBA/KLF4/LIF/LIFR/MYC/TBX3/WNT2/WNT4/WNT5A/ZFHX3 | 21 |
| GT | hsa04670 | Leukocyte transendothelial migration | 18/615 | 115/8773 | 0.001008704 | 0.008489927 | 0.006724695 | ACTN1/CDH5/CLDN1/CLDN4/CLDN5/CLDN7/CTNND1/CXCL12/ESAM/ITGB1/JAM2/MMP2/MYL9/PECAM1/PTK2/RAPGEF3/THY1/VCAM1 | 18 |

| | | | | | | | | | |
|---|---|---|---|---|---|---|---|---|---|
| GT | hsa05217 | Basal cell carcinoma | 12/615 | 63/8773 | 0.001233538 | 0.010101676 | 0.008001328 | BMP2/BMP4/FZD1/FZD4/FZD6/FZD7/GADD45A/TCF7L1/TCF7L2/WNT2/WNT4/WNT5A | 12 |
| GT | hsa04810 | Regulation of actin cytoskeleton | 29/615 | 229/8773 | 0.001324954 | 0.010564768 | 0.008368133 | ACTN1/ARHGEF12/BDKRB2/CXCL12/DOCK1/EGFR/ENAH/F2R/FGFR1/FN1/ITGA1/ITGA2/ITGA6/ITGAV/ITGB1/ITGB4/ITGB5/ITGB8/LPAR1/MYH11/MYL9/MYLK/NCKAP1/PDGFA/PDGFD/PDGFRB/PTK2/RDX/SCIN | 29 |
| GT | hsa05219 | Bladder cancer | 9/615 | 41/8773 | 0.001772215 | 0.013768744 | 0.010905936 | CCND1/CDH1/EGFR/HBEGF/MMP2/MYC/THBS1/TYMP/VEGFA | 9 |
| GT | hsa04115 | p53 signaling pathway | 13/615 | 75/8773 | 0.001937549 | 0.014676932 | 0.011625293 | CCNB1/CCNB2/CCND1/CDK1/GADD45A/IGFBP3/PERP/PMAIP1/RRM2/SERPINB5/SERPINE1/SFN/THBS1 | 13 |
| GT | hsa04310 | Wnt signaling pathway | 23/615 | 174/8773 | 0.002323919 | 0.017174331 | 0.013603431 | APCDD1/CCN4/CCND1/DKK1/DKK2/FOSL1/FRZB/FZD1/FZD4/FZD6/FZD7/JUN/MCC/MYC/PLCB1/SERPINF1/SFRP2/SOX17/TCF7L1/TCF7L2/WNT2/WNT4/WNT5A | 23 |
| GT | hsa05414 | Dilated cardiomyopathy | 16/615 | 105/8773 | 0.002502822 | 0.018056071 | 0.014301839 | ADCY4/DMD/ITGA1/ITGA2/ITGA6/ITGAV/ITGB1/ITGB4/ITGB5/ITGB8/LMNA/SGCB/SSPN/TGFB3/TPM1/TPM2 | 16 |
| GT | hsa05130 | Pathogenic Escherichia coli infection | 25/615 | 198/8773 | 0.002890434 | 0.02007492 | 0.015900927 | ABL1/ARHGEF12/CLDN1/CLDN4/CLDN5/CLDN7/CTTN/F2R/IL18/IL1R1/IL6/ITGB1/JUN/LPAR1/MYH11/MYO10/MYO1B/MYO5C/NCKAP1/TNFRSF1A/TNFSF10/TUBB2A/TUBB2B/TUBB3/TUBB6 | 25 |
| GT | hsa05215 | Prostate cancer | 15/615 | 97/8773 | 0.00291517 | 0.02007492 | 0.015900927 | CCND1/EGFR/ERG/FGFR1/HSP90B1/MMP3/PDGFA/PDGFD/PDGFRB/PLAT/PLAU/SPINT1/TCF7L1/TCF7L2/TMPRSS2 | 15 |
| GT | hsa04392 | Hippo signaling pathway - multiple species | 7/615 | 29/8773 | 0.003220525 | 0.021684866 | 0.017176131 | FAT4/FRMD6/RASSF4/SAV1/TEAD1/WWTR1/YAP1 | 7 |
| GT | hsa05226 | Gastric cancer | 20/615 | 149/8773 | 0.003650304 | 0.024044394 | 0.019045064 | ABCB1/CCND1/CDH1/CDKN2B/EGFR/FZD1/FZD4/FZD6/FZD7/GADD45A/HGF/JUP/MYC/TCF7L1/TCF7L2/TGFB3/TGFBR2/WNT2/WNT4/WNT5A | 20 |
| GT | hsa04360 | Axon guidance | 23/615 | 182/8773 | 0.004131195 | 0.026633022 | 0.021095463 | ABL1/ARHGEF12/BMPR2/CXCL12/DPYSL2/EFNA1/ENAH/EPHA2/ITGB1/MYL9/NEO1/NRP1/PTK2/RGS3/RHOD/ROBO1/SEMA3C/SEMA5A/SLIT3/TRPC6/UNC5B/WNT4/WNT5A | 23 |
| GT | hsa04916 | Melanogenesis | 15/615 | 101/8773 | 0.004325013 | 0.027301645 | 0.021625065 | ADCY4/CALML3/CALML5/EDN1/EDNRB/FZD1/FZD4/FZD6/FZD7/PLCB1/TCF7L1/TCF7L2/WNT2/WNT4/WNT5A | 15 |
| GT | hsa04142 | Lysosome | 18/615 | 132/8773 | 0.004789778 | 0.029618422 | 0.023460136 | CD63/CTSB/CTSD/CTSH/CTSK/CTSL/CTSO/CTSV/CTSZ/FUCA1/GM2A/HYAL2/LAMP3/LAPTM4A/LGMN/NCOA7/NPC2/PPT1 | 18 |
| GT | hsa04630 | JAK-STAT signaling pathway | 21/615 | 166/8773 | 0.005912355 | 0.035828874 | 0.028379306 | CCND1/CSF3/EGFR/FHL1/IL11/IL11RA/IL13/IL19/IL22/IL24/IL2RA/IL3RA/IL6/LEPR/LIF/LIFR/MYC/OSMR/PDGFA/PDGFRB/SOCS3 | 21 |

| | | | | | | | | | |
|---|---|---|---|---|---|---|---|---|---|
| GT | hsa04915 | Estrogen signaling pathway | 18/615 | 138/8773 | 0.007617223 | 0.045255265 | 0.035845754 | ADCY4/CALML3/CALML5/CTSD/EGFR/HBEGF/HSP90B1/HSPA6/JUN/KRT13/KRT14/KRT15/KRT16/KRT17/KRT18/KRT19/MMP2/PLCB1 | 18 |
| GT | hsa05202 | Transcriptional misregulation in cancer | 23/615 | 193/8773 | 0.008410866 | 0.048352561 | 0.038299058 | CD40/CEBPB/ERG/ETV1/FLT1/GADD45A/IGFBP3/IL6/JUP/LMO2/MEF2C/MMP3/MYC/NUPR1/PBX1/PDGFA/PLAT/PLAU/PTK2/SPINT1/TGFBR2/TMPRSS2/TSPAN7 | 23 |
| GT | hsa04926 | Relaxin signaling pathway | 17/615 | 129/8773 | 0.008457709 | 0.048352561 | 0.038299058 | ACTA2/ADCY4/COL1A1/COL1A2/COL3A1/COL4A1/COL4A2/EDN1/EDNRB/EGFR/GNG11/JUN/MMP13/MMP2/PLCB1/TGFBR2/VEGFA | 17 |
| PT | hsa05332 | Graft-versus-host disease | 18/142 | 42/8773 | 4.91E-22 | 1.03E-19 | 7.60E-20 | CD86/GZMB/HLA-DMA/HLA-DMB/HLA-DPA1/HLA-DPB1/HLA-DQA1/HLA-DQB1/HLA-DRA/HLA-DRB1/IFNG/IL1B/IL2/KIR3DL2/KLRC1/KLRD1/PRF1/TNF | 18 |
| PT | hsa05140 | Leishmaniasis | 21/142 | 77/8773 | 1.01E-20 | 1.06E-18 | 7.82E-19 | CYBB/FCGR2A/FCGR3A/FCGR3B/HLA-DMA/HLA-DMB/HLA-DPA1/HLA-DPB1/HLA-DQA1/HLA-DQB1/HLA-DRA/HLA-DRB1/IFNG/IL10/IL1B/ITGB2/MARCKSL1/NCF2/NFKB1/PTGS2/TNF | 21 |
| PT | hsa05330 | Allograft rejection | 16/142 | 38/8773 | 1.54E-19 | 1.08E-17 | 7.95E-18 | CD40LG/CD86/GZMB/HLA-DMA/HLA-DMB/HLA-DPA1/HLA-DPB1/HLA-DQA1/HLA-DQB1/HLA-DRA/HLA-DRB1/IFNG/IL10/IL2/PRF1/TNF | 16 |
| PT | hsa04612 | Antigen processing and presentation | 20/142 | 78/8773 | 3.48E-19 | 1.83E-17 | 1.35E-17 | CD74/CD8A/CD8B/CTSS/HLA-DMA/HLA-DMB/HLA-DPA1/HLA-DPB1/HLA-DQA1/HLA-DQB1/HLA-DRA/HLA-DRB1/IFI30/IFNG/KIR3DL2/KLRC1/KLRC2/KLRC3/KLRD1/TNF | 20 |
| PT | hsa04940 | Type I diabetes mellitus | 16/142 | 43/8773 | 1.72E-18 | 7.21E-17 | 5.31E-17 | CD86/GZMB/HLA-DMA/HLA-DMB/HLA-DPA1/HLA-DPB1/HLA-DQA1/HLA-DQB1/HLA-DRA/HLA-DRB1/IFNG/IL1B/IL2/LTA/PRF1/TNF | 16 |
| PT | hsa05323 | Rheumatoid arthritis | 20/142 | 93/8773 | 1.53E-17 | 5.35E-16 | 3.94E-16 | CCL3/CCL5/CD86/CTLA4/CXCL8/HLA-DMA/HLA-DMB/HLA-DPA1/HLA-DPB1/HLA-DQA1/HLA-DQB1/HLA-DRA/HLA-DRB1/IFNG/IL1B/IL23A/ITGB2/LTB/TNF/TNFSF13B | 20 |
| PT | hsa04672 | Intestinal immune network for IgA production | 15/142 | 49/8773 | 6.38E-16 | 1.91E-14 | 1.41E-14 | CD40LG/CD86/HLA-DMA/HLA-DMB/HLA-DPA1/HLA-DPB1/HLA-DQA1/HLA-DQB1/HLA-DRA/HLA-DRB1/ICOS/IL10/IL2/TNFRSF13C/TNFSF13B | 15 |
| PT | hsa05152 | Tuberculosis | 24/142 | 180/8773 | 7.69E-16 | 2.02E-14 | 1.49E-14 | CD14/CD74/CLEC7A/CTSS/FCER1G/FCGR2A/FCGR3A/FCGR3B/HLA-DMA/HLA-DMB/HLA-DPA1/HLA-DPB1/HLA-DQA1/HLA-DQB1/HLA-DRA/HLA-DRB1/IFNG/IL10/IL1B/IL23A/ITGAX/ITGB2/NFKB1/TNF | 24 |

| | | | | | | | | | |
|---|---|---|---|---|---|---|---|---|---|
| PT | hsa05320 | Autoimmune thyroid disease | 15/142 | 53/8773 | 2.40E-15 | 5.59E-14 | 4.12E-14 | CD40LG/CD86/CTLA4/GZMB/HLA-DMA/HLA-DMB/HLA-DPA1/HLA-DPB1/HLA-DQA1/HLA-DQB1/HLA-DRA/HLA-DRB1/IL10/IL2/PRF1 | 15 |
| PT | hsa05321 | Inflammatory bowel disease | 16/142 | 65/8773 | 3.11E-15 | 6.52E-14 | 4.80E-14 | HLA-DMA/HLA-DMB/HLA-DPA1/HLA-DPB1/HLA-DQA1/HLA-DQB1/HLA-DRA/HLA-DRB1/IFNG/IL10/IL1B/IL2/IL23A/MAF/NFKB1/TNF | 16 |
| PT | hsa04640 | Hematopoietic cell lineage | 18/142 | 99/8773 | 1.61E-14 | 3.08E-13 | 2.27E-13 | CD14/CD55/CD8A/CD8B/HLA-DMA/HLA-DMB/HLA-DPA1/HLA-DPB1/HLA-DQA1/HLA-DQB1/HLA-DRA/HLA-DRB1/IL1B/KIT/MME/MS4A1/TFRC/TNF | 18 |
| PT | hsa05310 | Asthma | 12/142 | 31/8773 | 2.19E-14 | 3.84E-13 | 2.83E-13 | CD40LG/FCER1G/HLA-DMA/HLA-DMB/HLA-DPA1/HLA-DPB1/HLA-DQA1/HLA-DQB1/HLA-DRA/HLA-DRB1/IL10/TNF | 12 |
| PT | hsa04060 | Cytokine-cytokine receptor interaction | 26/142 | 297/8773 | 1.15E-12 | 1.85E-11 | 1.36E-11 | CCL3/CCL4/CCL4L2/CCL5/CCR4/CCR6/CCR7/CD40LG/CXCL8/IFNG/IL10/IL1B/IL1RN/IL2/IL23A/LTA/LTB/OSM/TNF/TNFRSF13C/TNFRSF18/TNFRSF4/TNFRSF9/TNFSF13B/XCL1/XCL2 | 26 |
| PT | hsa05150 | Staphylococcus aureus infection | 16/142 | 96/8773 | 2.08E-12 | 3.11E-11 | 2.29E-11 | C1QC/C5AR1/FCGR2A/FCGR3A/FCGR3B/HLA-DMA/HLA-DMB/HLA-DPA1/HLA-DPB1/HLA-DQA1/HLA-DQB1/HLA-DRA/HLA-DRB1/IL10/ITGB2/KRT23 | 16 |
| PT | hsa05416 | Viral myocarditis | 14/142 | 69/8773 | 3.20E-12 | 4.30E-11 | 3.17E-11 | ACTB/CD40LG/CD55/CD86/HLA-DMA/HLA-DMB/HLA-DPA1/HLA-DPB1/HLA-DQA1/HLA-DQB1/HLA-DRA/HLA-DRB1/ITGB2/PRF1 | 14 |
| PT | hsa04145 | Phagosome | 19/142 | 152/8773 | 3.27E-12 | 4.30E-11 | 3.17E-11 | ACTB/CD14/CLEC7A/CTSS/CYBB/FCGR2A/FCGR3A/FCGR3B/HLA-DMA/HLA-DMB/HLA-DPA1/HLA-DPB1/HLA-DQA1/HLA-DQB1/HLA-DRA/HLA-DRB1/ITGB2/NCF2/TFRC | 19 |
| PT | hsa05322 | Systemic lupus erythematosus | 18/142 | 137/8773 | 5.37E-12 | 6.64E-11 | 4.89E-11 | C1QC/CD40LG/CD86/CTSG/FCGR2A/FCGR3A/FCGR3B/HLA-DMA/HLA-DMB/HLA-DPA1/HLA-DPB1/HLA-DQA1/HLA-DQB1/HLA-DRA/HLA-DRB1/IFNG/IL10/TNF | 18 |
| PT | hsa04064 | NF-kappa B signaling pathway | 15/142 | 105/8773 | 1.04E-10 | 1.22E-09 | 8.98E-10 | BCL2A1/CCL4/CCL4L2/CD14/CD40LG/CXCL8/GADD45G/IL1B/LTA/LTB/NFKB1/PTGS2/TNF/TNFRSF13C/TNFSF13B | 15 |
| PT | hsa05164 | Influenza A | 18/142 | 171/8773 | 2.32E-10 | 2.47E-09 | 1.82E-09 | ACTB/CCL5/CXCL8/HLA-DMA/HLA-DMB/HLA-DPA1/HLA-DPB1/HLA-DQA1/HLA-DQB1/HLA-DRA/HLA-DRB1/IFNG/IL1B/NFKB1/NLRP3/RSAD2/TNF/TPSB2 | 18 |
| PT | hsa05145 | Toxoplasmosis | 15/142 | 111/8773 | 2.35E-10 | 2.47E-09 | 1.82E-09 | CD40LG/GNAO1/HLA-DMA/HLA-DMB/HLA-DPA1/HLA-DPB1/HLA-DQA1/HLA-DQB1/HLA-DRA/HLA-DRB1/IFNG/IL10/NFKB1/PPIF/TNF | 15 |
| PT | hsa04061 | Viral protein interaction with | 14/142 | 100/8773 | 5.97E-10 | 5.97E-09 | 4.40E-09 | CCL3/CCL4/CCL4L2/CCL5/CCR4/CCR6/CCR7/CXCL8/IL10/IL2/LTA/TNF/XCL1/XCL2 | 14 |

| | | | | | | | | | |
|---|---|---|---|---|---|---|---|---|---|
| | | cytokine and cytokine receptor | | | | | | | |
| PT | hsa04659 | Th17 cell differentiation | 14/142 | 108/8773 | 1.69E-09 | 1.62E-08 | 1.19E-08 | HLA-DMA/HLA-DMB/HLA-DPA1/HLA-DPB1/HLA-DQA1/HLA-DQB1/HLA-DRA/HLA-DRB1/IFNG/IL1B/IL2/IL23A/IRF4/NFKB1 | 14 |
| PT | hsa04650 | Natural killer cell mediated cytotoxicity | 15/142 | 132/8773 | 2.79E-09 | 2.55E-08 | 1.88E-08 | FCER1G/FCGR3A/FCGR3B/GZMB/IFNG/ITGB2/KIR3DL2/KLRC1/KLRC2/KLRC3/KLRD1/NCR3/PRF1/TNF/TYROBP | 15 |
| PT | hsa05144 | Malaria | 10/142 | 50/8773 | 5.31E-09 | 4.65E-08 | 3.42E-08 | CD40LG/CXCL8/HBA1/HBA2/IFNG/IL10/IL1B/ITGB2/KLRB1/TNF | 10 |
| PT | hsa04658 | Th1 and Th2 cell differentiation | 12/142 | 92/8773 | 2.43E-08 | 2.04E-07 | 1.50E-07 | HLA-DMA/HLA-DMB/HLA-DPA1/HLA-DPB1/HLA-DQA1/HLA-DQB1/HLA-DRA/HLA-DRB1/IFNG/IL2/MAF/NFKB1 | 12 |
| PT | hsa04514 | Cell adhesion molecules | 15/142 | 157/8773 | 3.05E-08 | 2.45E-07 | 1.81E-07 | CD40LG/CD86/CD8A/CD8B/CTLA4/HLA-DMA/HLA-DMB/HLA-DPA1/HLA-DPB1/HLA-DQA1/HLA-DQB1/HLA-DRA/HLA-DRB1/ICOS/ITGB2 | 15 |
| PT | hsa05133 | Pertussis | 11/142 | 76/8773 | 3.16E-08 | 2.45E-07 | 1.81E-07 | C1QC/CD14/CXCL8/IL10/IL1B/IL23A/IRF8/ITGB2/NFKB1/NLRP3/TNF | 11 |
| PT | hsa05166 | Human T-cell leukemia virus 1 infection | 16/142 | 222/8773 | 5.31E-07 | 3.98E-06 | 2.93E-06 | CDC20/CREB5/HLA-DMA/HLA-DMB/HLA-DPA1/HLA-DPB1/HLA-DQA1/HLA-DQB1/HLA-DRA/HLA-DRB1/IL2/ITGB2/LTA/NFKB1/TNF/TNFRSF13C | 16 |
| PT | hsa05142 | Chagas disease | 11/142 | 102/8773 | 6.86E-07 | 4.97E-06 | 3.66E-06 | C1QC/CCL3/CCL5/CXCL8/GNAO1/IFNG/IL10/IL1B/IL2/NFKB1/TNF | 11 |
| PT | hsa05146 | Amoebiasis | 10/142 | 102/8773 | 5.38E-06 | 3.77E-05 | 2.78E-05 | CD14/CTSG/CXCL8/IFNG/IL10/IL1B/ITGB2/NFKB1/SERPINB9/TNF | 10 |
| PT | hsa04625 | C-type lectin receptor signaling pathway | 10/142 | 104/8773 | 6.41E-06 | 4.34E-05 | 3.20E-05 | CLEC7A/FCER1G/IL10/IL1B/IL2/IL23A/NFKB1/NLRP3/PTGS2/TNF | 10 |
| PT | hsa04620 | Toll-like receptor signaling pathway | 10/142 | 108/8773 | 9.01E-06 | 5.91E-05 | 4.35E-05 | CCL3/CCL4/CCL4L2/CCL5/CD14/CD86/CXCL8/IL1B/NFKB1/TNF | 10 |
| PT | hsa05135 | Yersinia infection | 11/142 | 137/8773 | 1.26E-05 | 8.00E-05 | 5.90E-05 | ACTB/CD8A/CD8B/CXCL8/FCGR2A/IL10/IL1B/IL2/NFKB1/NLRP3/TNF | 11 |
| PT | hsa04660 | T cell receptor signaling pathway | 10/142 | 121/8773 | 2.46E-05 | 0.000149258 | 0.00010998 | CD40LG/CD8A/CD8B/CTLA4/ICOS/IFNG/IL10/IL2/NFKB1/TNF | 10 |
| PT | hsa05143 | African trypanosomiasis | 6/142 | 37/8773 | 2.49E-05 | 0.000149258 | 0.00010998 | HBA1/HBA2/IFNG/IL10/IL1B/TNF | 6 |

| | | | | | | | | | |
|---|---|---|---|---|---|---|---|---|---|
| PT | hsa04613 | Neutrophil extracellular trap formation | 12/142 | 191/8773 | 5.90E-05 | 0.000344283 | 0.000253682 | ACTB/C5AR1/CLEC7A/CTSG/CYBB/FCGR2A/FCGR3A/FCGR3B/ITGB2/NCF2/NFKB1/PPIF | 12 |
| PT | hsa05417 | Lipid and atherosclerosis | 12/142 | 215/8773 | 0.000182562 | 0.001036162 | 0.000763488 | CCL3/CCL5/CD14/CD40LG/CXCL8/CYBB/IL1B/NCF2/NFKB1/NLRP3/TNF/XBP1 | 12 |
| PT | hsa05134 | Legionellosis | 6/142 | 56/8773 | 0.000269956 | 0.001466133 | 0.001080309 | CD14/CXCL8/IL1B/ITGB2/NFKB1/TNF | 6 |
| PT | hsa04062 | Chemokine signaling pathway | 11/142 | 192/8773 | 0.000272282 | 0.001466133 | 0.001080309 | CCL3/CCL4/CCL4L2/CCL5/CCR4/CCR6/CCR7/CXCL8/NFKB1/XCL1/XCL2 | 11 |
| PT | hsa05202 | Transcriptional misregulation in cancer | 11/142 | 193/8773 | 0.000284792 | 0.001495157 | 0.001101694 | BCL2A1/CD14/CD86/CXCL8/GADD45G/GZMB/HHEX/HPGD/MAF/NFKB1/NR4A3 | 11 |
| PT | hsa05340 | Primary immunodeficiency | 5/142 | 38/8773 | 0.000338052 | 0.001731485 | 0.001275831 | CD40LG/CD8A/CD8B/ICOS/TNFRSF13C | 5 |
| PT | hsa05169 | Epstein-Barr virus infection | 11/142 | 202/8773 | 0.000420917 | 0.002104584 | 0.001550746 | GADD45G/HLA-DMA/HLA-DMB/HLA-DPA1/HLA-DPB1/HLA-DQA1/HLA-DQB1/HLA-DRA/HLA-DRB1/NFKB1/TNF | 11 |
| PT | hsa04380 | Osteoclast differentiation | 9/142 | 141/8773 | 0.000452975 | 0.002212203 | 0.001630044 | FCGR2A/FCGR3A/FCGR3B/IFNG/IL1B/NCF2/NFKB1/TNF/TYROBP | 9 |
| PT | hsa04610 | Complement and coagulation cascades | 7/142 | 86/8773 | 0.000464478 | 0.002216828 | 0.001633452 | C1QC/C5AR1/CD55/F13A1/ITGAX/ITGB2/SERPINA1 | 7 |
| PT | hsa05163 | Human cytomegalovirus infection | 11/142 | 225/8773 | 0.001033941 | 0.004825059 | 0.003555306 | CCL3/CCL4/CCL4L2/CCL5/CREB5/CXCL8/GNAO1/IL1B/NFKB1/PTGS2/TNF | 11 |
| PT | hsa05171 | Coronavirus disease - COVID-19 | 11/142 | 233/8773 | 0.001372242 | 0.006264582 | 0.004616008 | C1QC/C5AR1/CXCL8/CYBB/F13A1/FCGR2A/IL1B/IL2/NFKB1/NLRP3/TNF | 11 |
| PT | hsa04210 | Apoptosis | 8/142 | 135/8773 | 0.001521745 | 0.006799285 | 0.00501 | ACTB/BCL2A1/CTSS/GADD45G/GZMB/NFKB1/PRF1/TNF | 8 |
| PT | hsa04623 | Cytosolic DNA-sensing pathway | 6/142 | 83/8773 | 0.002196878 | 0.009611343 | 0.007082042 | CCL4/CCL4L2/CCL5/IL1B/NFKB1/NLRP3 | 6 |
| PT | hsa04668 | TNF signaling pathway | 7/142 | 114/8773 | 0.002445329 | 0.010479981 | 0.007722091 | CCL5/CREB5/IL1B/LTA/NFKB1/PTGS2/TNF | 7 |
| PT | hsa04657 | IL-17 signaling pathway | 6/142 | 94/8773 | 0.004101008 | 0.017224234 | 0.012691541 | CXCL8/IFNG/IL1B/NFKB1/PTGS2/TNF | 6 |

| | | | | | | | | | |
|---|---|---|---|---|---|---|---|---|---|
| PT | hsa04614 | Renin-angiotensin system | 3/142 | 23/8773 | 0.005799762 | 0.023881372 | 0.0175968 | CPA3/CTSG/MME | 3 |
| PT | hsa05418 | Fluid shear stress and atherosclerosis | 7/142 | 139/8773 | 0.007268816 | 0.029354833 | 0.021629877 | ACTB/IFNG/IL1B/NCF2/NFKB1/NPPC/TNF | 7 |
| PT | hsa04936 | Alcoholic liver disease | 7/142 | 142/8773 | 0.008137933 | 0.03224464 | 0.023759209 | C1QC/C5AR1/CD14/CXCL8/IL1B/NFKB1/TNF | 7 |
| PT | hsa04621 | NOD-like receptor signaling pathway | 8/142 | 186/8773 | 0.010552048 | 0.041035742 | 0.030236862 | CCL5/CXCL8/CYBB/IL1B/NAMPT/NFKB1/NLRP3/TNF | 8 |
| PT | hsa01523 | Antifolate resistance | 3/142 | 30/8773 | 0.012245737 | 0.046756451 | 0.034452122 | IL1B/NFKB1/TNF | 3 |
| ST | hsa05332 | Graft-versus-host disease | 17/157 | 42/8773 | 1.43E-19 | 3.01E-17 | 2.30E-17 | CD86/GZMB/HLA-DMB/HLA-DOB/HLA-DPA1/HLA-DPB1/HLA-DQA1/HLA-DQB1/HLA-DRA/HLA-DRB5/IFNG/IL1B/KIR2DL3/KIR3DL2/KLRC1/KLRD1/PRF1 | 17 |
| ST | hsa05323 | Rheumatoid arthritis | 22/157 | 93/8773 | 3.35E-19 | 3.53E-17 | 2.70E-17 | CCL3/CCL3L1/CCL5/CD86/CSF2/CTLA4/CXCL8/HLA-DMB/HLA-DOB/HLA-DPA1/HLA-DPB1/HLA-DQA1/HLA-DQB1/HLA-DRA/HLA-DRB5/IFNG/IL1B/IL23A/ITGB2/LTB/TNFSF13/TNFSF13B | 22 |
| ST | hsa05140 | Leishmaniasis | 19/157 | 77/8773 | 4.39E-17 | 3.02E-15 | 2.30E-15 | CYBB/FCGR2A/FCGR3A/FCGR3B/HLA-DMB/HLA-DOB/HLA-DPA1/HLA-DPB1/HLA-DQA1/HLA-DQB1/HLA-DRA/HLA-DRB5/IFNG/IL1B/ITGB2/MARCKSL1/NCF2/NFKB1/PTGS2 | 19 |
| ST | hsa04612 | Antigen processing and presentation | 19/157 | 78/8773 | 5.72E-17 | 3.02E-15 | 2.30E-15 | CD4/CD8B/CTSS/HLA-DMB/HLA-DOB/HLA-DPA1/HLA-DPB1/HLA-DQA1/HLA-DQB1/HLA-DRA/HLA-DRB5/IFI30/IFNG/KIR2DL3/KIR3DL2/KLRC1/KLRC2/KLRC3/KLRD1 | 19 |
| ST | hsa04672 | Intestinal immune network for IgA production | 16/157 | 49/8773 | 1.03E-16 | 4.34E-15 | 3.31E-15 | CD40LG/CD86/HLA-DMB/HLA-DOB/HLA-DPA1/HLA-DPB1/HLA-DQA1/HLA-DQB1/HLA-DRA/HLA-DRB5/ICOS/ICOSLG/TNFRSF13B/TNFRSF13C/TNFSF13/TNFSF13B | 16 |
| ST | hsa04060 | Cytokine-cytokine receptor interaction | 30/157 | 297/8773 | 5.70E-15 | 2.00E-13 | 1.53E-13 | CCL3/CCL3L1/CCL4/CCL4L2/CCL5/CCR6/CCR7/CD4/CD40LG/CSF2/CSF3R/CXCL16/CXCL8/IFNG/IL1B/IL1R2/IL1RN/IL23A/LTA/LTB/OSM/TNFRSF13B/TNFRSF13C/TNFRSF18/TNFRSF4/TNFSF13/TNFSF13B/TNFSF15/XCL1/XCL2 | 30 |
| ST | hsa04940 | Type I diabetes mellitus | 14/157 | 43/8773 | 9.65E-15 | 2.91E-13 | 2.22E-13 | CD86/GZMB/HLA-DMB/HLA-DOB/HLA-DPA1/HLA-DPB1/HLA-DQA1/HLA-DQB1/HLA-DRA/HLA-DRB5/IFNG/IL1B/LTA/PRF1 | 14 |

| | | | | | | | | | |
|---|---|---|---|---|---|---|---|---|---|
| ST | hsa05330 | Allograft rejection | 13/157 | 38/8773 | 4.31E-14 | 1.14E-12 | 8.68E-13 | CD40LG/CD86/GZMB/HLA-DMB/HLA-DOB/HLA-DPA1/HLA-DPB1/HLA-DQA1/HLA-DQB1/HLA-DRA/HLA-DRB5/IFNG/PRF1 | 13 |
| ST | hsa04640 | Hematopoietic cell lineage | 18/157 | 99/8773 | 9.62E-14 | 2.26E-12 | 1.72E-12 | CD14/CD1A/CD4/CD55/CD8B/CSF2/CSF3R/HLA-DMB/HLA-DOB/HLA-DPA1/HLA-DPB1/HLA-DQA1/HLA-DQB1/HLA-DRA/HLA-DRB5/IL1B/IL1R2/MME | 18 |
| ST | hsa05152 | Tuberculosis | 22/157 | 180/8773 | 7.23E-13 | 1.53E-11 | 1.17E-11 | CD14/CLEC7A/CTSS/FCER1G/FCGR2A/FCGR2B/FCGR3A/FCGR3B/HLA-DMB/HLA-DOB/HLA-DPA1/HLA-DPB1/HLA-DQA1/HLA-DQB1/HLA-DRA/HLA-DRB5/IFNG/IL1B/IL23A/ITGAX/ITGB2/NFKB1 | 22 |
| ST | hsa05320 | Autoimmune thyroid disease | 13/157 | 53/8773 | 5.32E-12 | 1.02E-10 | 7.78E-11 | CD40LG/CD86/CTLA4/GZMB/HLA-DMB/HLA-DOB/HLA-DPA1/HLA-DPB1/HLA-DQA1/HLA-DQB1/HLA-DRA/HLA-DRB5/PRF1 | 13 |
| ST | hsa05150 | Staphylococcus aureus infection | 16/157 | 96/8773 | 9.91E-12 | 1.74E-10 | 1.33E-10 | C3AR1/C5AR1/FCGR2A/FCGR2B/FCGR3A/FCGR3B/HLA-DMB/HLA-DOB/HLA-DPA1/HLA-DPB1/HLA-DQA1/HLA-DQB1/HLA-DRA/HLA-DRB5/ITGB2/KRT23 | 16 |
| ST | hsa04145 | Phagosome | 19/157 | 152/8773 | 2.01E-11 | 3.26E-10 | 2.49E-10 | CD14/CLEC7A/CTSS/CYBB/FCGR2A/FCGR2B/FCGR3A/FCGR3B/HLA-DMB/HLA-DOB/HLA-DPA1/HLA-DPB1/HLA-DQA1/HLA-DQB1/HLA-DRA/HLA-DRB5/ITGB2/NCF2/OLR1 | 19 |
| ST | hsa05310 | Asthma | 10/157 | 31/8773 | 8.12E-11 | 1.22E-09 | 9.34E-10 | CD40LG/FCER1G/HLA-DMB/HLA-DOB/HLA-DPA1/HLA-DPB1/HLA-DQA1/HLA-DQB1/HLA-DRA/HLA-DRB5 | 10 |
| ST | hsa05416 | Viral myocarditis | 13/157 | 69/8773 | 1.91E-10 | 2.68E-09 | 2.05E-09 | CD40LG/CD55/CD86/HLA-DMB/HLA-DOB/HLA-DPA1/HLA-DPB1/HLA-DQA1/HLA-DQB1/HLA-DRA/HLA-DRB5/ITGB2/PRF1 | 13 |
| ST | hsa05321 | Inflammatory bowel disease | 12/157 | 65/8773 | 1.26E-09 | 1.66E-08 | 1.27E-08 | HLA-DMB/HLA-DOB/HLA-DPA1/HLA-DPB1/HLA-DQA1/HLA-DQB1/HLA-DRA/HLA-DRB5/IFNG/IL1B/IL23A/NFKB1 | 12 |
| ST | hsa04659 | Th17 cell differentiation | 14/157 | 108/8773 | 6.33E-09 | 7.85E-08 | 5.99E-08 | CD4/HLA-DMB/HLA-DOB/HLA-DPA1/HLA-DPB1/HLA-DQA1/HLA-DQB1/HLA-DRA/HLA-DRB5/IFNG/IL1B/IL23A/IRF4/NFKB1 | 14 |
| ST | hsa04650 | Natural killer cell mediated cytotoxicity | 15/157 | 132/8773 | 1.12E-08 | 1.31E-07 | 1.00E-07 | CSF2/FCER1G/FCGR3A/FCGR3B/GZMB/IFNG/ITGB2/KIR2DL3/KIR3DL2/KLRC1/KLRC2/KLRC3/KLRD1/PRF1/TYROBP | 15 |
| ST | hsa04514 | Cell adhesion molecules | 16/157 | 157/8773 | 1.73E-08 | 1.92E-07 | 1.47E-07 | CD4/CD40LG/CD86/CD8B/CTLA4/HLA-DMB/HLA-DOB/HLA-DPA1/HLA-DPB1/HLA-DQA1/HLA-DQB1/HLA-DRA/HLA-DRB5/ICOS/ICOSLG/ITGB2 | 16 |
| ST | hsa04064 | NF-kappa B signaling pathway | 13/157 | 105/8773 | 3.96E-08 | 4.18E-07 | 3.19E-07 | BCL2A1/CCL4/CCL4L2/CD14/CD40LG/CXCL8/IL1B/LTA/LTB/NFKB1/PTGS2/TNFRSF13C/TNFSF13B | 13 |

| | | | | | | | | | |
|---|---|---|---|---|---|---|---|---|---|
| ST | hsa05166 | Human T-cell leukemia virus 1 infection | 18/157 | 222/8773 | 7.70E-08 | 7.45E-07 | 5.68E-07 | CD4/CREB5/CSF2/EGR2/HLA-DMB/HLA-DOB/HLA-DPA1/HLA-DPB1/HLA-DQA1/HLA-DQB1/HLA-DRA/HLA-DRB5/IL1R2/ITGB2/LTA/NFKB1/SPI1/TNFRSF13C | 18 |
| ST | hsa05145 | Toxoplasmosis | 13/157 | 111/8773 | 7.77E-08 | 7.45E-07 | 5.68E-07 | ALOX5/CD40LG/HLA-DMB/HLA-DOB/HLA-DPA1/HLA-DPB1/HLA-DQA1/HLA-DQB1/HLA-DRA/HLA-DRB5/IFNG/NFKB1/PPIF | 13 |
| ST | hsa05322 | Systemic lupus erythematosus | 14/157 | 137/8773 | 1.37E-07 | 1.26E-06 | 9.60E-07 | CD40LG/CD86/FCGR2A/FCGR3A/FCGR3B/HLA-DMB/HLA-DOB/HLA-DPA1/HLA-DPB1/HLA-DQA1/HLA-DQB1/HLA-DRA/HLA-DRB5/IFNG | 14 |
| ST | hsa04658 | Th1 and Th2 cell differentiation | 11/157 | 92/8773 | 6.55E-07 | 5.76E-06 | 4.40E-06 | CD4/HLA-DMB/HLA-DOB/HLA-DPA1/HLA-DPB1/HLA-DQA1/HLA-DQB1/HLA-DRA/HLA-DRB5/IFNG/NFKB1 | 11 |
| ST | hsa04657 | IL-17 signaling pathway | 11/157 | 94/8773 | 8.16E-07 | 6.89E-06 | 5.26E-06 | CSF2/CXCL8/IFNG/IL1B/MMP13/MMP9/NFKB1/PTGS2/S100A7A/S100A8/S100A9 | 11 |
| ST | hsa04061 | Viral protein interaction with cytokine and cytokine receptor | 11/157 | 100/8773 | 1.53E-06 | 1.24E-05 | 9.46E-06 | CCL3/CCL3L1/CCL4/CCL4L2/CCL5/CCR6/CCR7/CXCL8/LTA/XCL1/XCL2 | 11 |
| ST | hsa05202 | Transcriptional misregulation in cancer | 15/157 | 193/8773 | 1.71E-06 | 1.34E-05 | 1.02E-05 | BCL2A1/CD14/CD86/CSF2/CXCL8/DUSP6/GZMB/HHEX/IL1R2/MMP9/NFKB1/NR4A3/RUNX2/SPI1/ZBTB16 | 15 |
| ST | hsa05146 | Amoebiasis | 11/157 | 102/8773 | 1.86E-06 | 1.40E-05 | 1.07E-05 | CD14/CD1A/CSF2/CXCL8/GNA15/IFNG/IL1B/IL1R2/ITGB2/NFKB1/SERPINB9 | 11 |
| ST | hsa05164 | Influenza A | 14/157 | 171/8773 | 2.08E-06 | 1.51E-05 | 1.16E-05 | CCL5/CXCL8/HLA-DMB/HLA-DOB/HLA-DPA1/HLA-DPB1/HLA-DQA1/HLA-DQB1/HLA-DRA/HLA-DRB5/IFNG/IL1B/NFKB1/NLRP3 | 14 |
| ST | hsa05340 | Primary immunodeficiency | 7/157 | 38/8773 | 4.07E-06 | 2.86E-05 | 2.19E-05 | CD4/CD40LG/CD79A/CD8B/ICOS/TNFRSF13B/TNFRSF13C | 7 |
| ST | hsa05417 | Lipid and atherosclerosis | 15/157 | 215/8773 | 6.52E-06 | 4.44E-05 | 3.39E-05 | CCL3/CCL3L1/CCL5/CD14/CD40LG/CXCL8/CYBB/IL1B/MMP9/NCF2/NFKB1/NLRP3/OLR1/SOD2/XBP1 | 15 |
| ST | hsa04380 | Osteoclast differentiation | 12/157 | 141/8773 | 7.72E-06 | 5.09E-05 | 3.89E-05 | FCGR2A/FCGR2B/FCGR3A/FCGR3B/IFNG/IL1B/LILRA5/LILRB2/NCF2/NFKB1/SPI1/TYROBP | 12 |
| ST | hsa04620 | Toll-like receptor signaling pathway | 10/157 | 108/8773 | 2.18E-05 | 0.000139157 | 0.000106216 | CCL3/CCL3L1/CCL4/CCL4L2/CCL5/CD14/CD86/CXCL8/IL1B/NFKB1 | 10 |
| ST | hsa05144 | Malaria | 7/157 | 50/8773 | 2.69E-05 | 0.000167058 | 0.000127512 | CD40LG/CXCL8/HBA2/IFNG/IL1B/ITGB2/KLRB1 | 7 |
| ST | hsa05133 | Pertussis | 8/157 | 76/8773 | 5.92E-05 | 0.000356649 | 0.000272224 | CD14/CXCL8/IL1B/IL23A/IRF8/ITGB2/NFKB1/NLRP3 | 8 |

| | | | | | | | | | |
|---|---|---|---|---|---|---|---|---|---|
| ST | hsa04062 | Chemokine signaling pathway | 12/157 | 192/8773 | 0.000163145 | 0.00095621 | 0.000729859 | CCL3/CCL3L1/CCL4/CCL4L2/CCL5/CCR6/CCR7/CXCL16/CXCL8/NFKB1/XCL1/XCL2 | 12 |
| ST | hsa05221 | Acute myeloid leukemia | 7/157 | 67/8773 | 0.000181738 | 0.001036396 | 0.000791063 | BCL2A1/CD14/CSF2/DUSP6/NFKB1/SPI1/ZBTB16 | 7 |
| ST | hsa05142 | Chagas disease | 8/157 | 102/8773 | 0.000464799 | 0.002580859 | 0.001969925 | CCL3/CCL3L1/CCL5/CXCL8/GNA15/IFNG/IL1B/NFKB1 | 8 |
| ST | hsa04625 | C-type lectin receptor signaling pathway | 8/157 | 104/8773 | 0.000529742 | 0.002866041 | 0.002187599 | CLEC7A/EGR2/FCER1G/IL1B/IL23A/NFKB1/NLRP3/PTGS2 | 8 |
| ST | hsa04610 | Complement and coagulation cascades | 7/157 | 86/8773 | 0.000845863 | 0.004461929 | 0.003405713 | C3AR1/C5AR1/CD55/ITGAX/ITGB2/PLAUR/SERPINA1 | 7 |
| ST | hsa04668 | TNF signaling pathway | 8/157 | 114/8773 | 0.000973798 | 0.005011497 | 0.003825188 | CCL5/CREB5/CSF2/IL1B/LTA/MMP9/NFKB1/PTGS2 | 8 |
| ST | hsa04662 | B cell receptor signaling pathway | 7/157 | 90/8773 | 0.001108222 | 0.005567495 | 0.004249572 | BANK1/CD79A/DAPP1/FCGR2B/LILRA5/LILRB2/NFKB1 | 7 |
| ST | hsa04660 | T cell receptor signaling pathway | 8/157 | 121/8773 | 0.001433169 | 0.007032525 | 0.005367804 | CD4/CD40LG/CD8B/CSF2/CTLA4/ICOS/IFNG/NFKB1 | 8 |
| ST | hsa04613 | Neutrophil extracellular trap formation | 10/157 | 191/8773 | 0.0022201 | 0.010646388 | 0.008126203 | C5AR1/CLEC7A/CYBB/FCGR2A/FCGR3A/FCGR3B/ITGB2/NCF2/NFKB1/PPIF | 10 |
| ST | hsa05134 | Legionellosis | 5/157 | 56/8773 | 0.003153979 | 0.014788658 | 0.011287926 | CD14/CXCL8/IL1B/ITGB2/NFKB1 | 5 |
| ST | hsa04623 | Cytosolic DNA-sensing pathway | 6/157 | 83/8773 | 0.003625013 | 0.016627776 | 0.012691692 | CCL4/CCL4L2/CCL5/IL1B/NFKB1/NLRP3 | 6 |
| ST | hsa05143 | African trypanosomiasis | 4/157 | 37/8773 | 0.00411759 | 0.018485349 | 0.014109546 | HBA2/IDO1/IFNG/IL1B | 4 |
| ST | hsa05163 | Human cytomegalovirus infection | 10/157 | 225/8773 | 0.007076683 | 0.031107918 | 0.023744133 | CCL3/CCL3L1/CCL4/CCL4L2/CCL5/CREB5/CXCL8/IL1B/NFKB1/PTGS2 | 10 |
| ST | hsa05169 | Epstein-Barr virus infection | 9/157 | 202/8773 | 0.01027362 | 0.044239464 | 0.033767214 | HLA-DMB/HLA-DOB/HLA-DPA1/HLA-DPB1/HLA-DQA1/HLA-DQB1/HLA-DRA/HLA-DRB5/NFKB1 | 9 |
| ST | hsa05135 | Yersinia infection | 7/157 | 137/8773 | 0.011384803 | 0.048043867 | 0.036671049 | CD4/CD8B/CXCL8/FCGR2A/IL1B/NFKB1/NLRP3 | 7 |

**Table 26. GO enrichment of scRNA-seq for each type of tissue**

p.adjust < 1E-11

| Cluster | ID | Description | GeneRatio | BgRatio | pvalue | p.adjust | qvalue | Count |
|---|---|---|---|---|---|---|---|---|
| GT | GO:0045229 | external encapsulating structure organization | 106/1121 | 317/18614 | 6.59E-51 | 3.55E-47 | 2.33E-47 | 106 |
| GT | GO:0030198 | extracellular matrix organization | 105/1121 | 314/18614 | 1.98E-50 | 5.04E-47 | 3.31E-47 | 105 |
| GT | GO:0043062 | extracellular structure organization | 105/1121 | 315/18614 | 2.81E-50 | 5.04E-47 | 3.31E-47 | 105 |
| GT | GO:0050673 | epithelial cell proliferation | 110/1121 | 488/18614 | 1.06E-34 | 1.43E-31 | 9.41E-32 | 110 |
| GT | GO:0008544 | epidermis development | 95/1121 | 375/18614 | 2.23E-34 | 2.40E-31 | 1.58E-31 | 95 |
| GT | GO:0042060 | wound healing | 103/1121 | 439/18614 | 4.18E-34 | 3.75E-31 | 2.46E-31 | 103 |
| GT | GO:0001667 | ameboidal-type cell migration | 109/1121 | 496/18614 | 2.46E-33 | 1.89E-30 | 1.24E-30 | 109 |
| GT | GO:0043588 | skin development | 83/1121 | 308/18614 | 3.27E-32 | 2.20E-29 | 1.45E-29 | 83 |
| GT | GO:0031589 | cell-substrate adhesion | 88/1121 | 359/18614 | 9.73E-31 | 5.82E-28 | 3.83E-28 | 88 |
| GT | GO:0090130 | tissue migration | 90/1121 | 379/18614 | 2.68E-30 | 1.44E-27 | 9.50E-28 | 90 |
| GT | GO:0010631 | epithelial cell migration | 88/1121 | 371/18614 | 1.33E-29 | 6.52E-27 | 4.29E-27 | 88 |
| GT | GO:0090132 | epithelium migration | 88/1121 | 374/18614 | 2.51E-29 | 1.13E-26 | 7.41E-27 | 88 |
| GT | GO:0050678 | regulation of epithelial cell proliferation | 92/1121 | 419/18614 | 3.42E-28 | 1.41E-25 | 9.31E-26 | 92 |
| GT | GO:1901342 | regulation of vasculature development | 82/1121 | 355/18614 | 8.48E-27 | 3.26E-24 | 2.14E-24 | 82 |
| GT | GO:0045765 | regulation of angiogenesis | 80/1121 | 349/18614 | 6.23E-26 | 2.24E-23 | 1.47E-23 | 80 |
| GT | GO:0043542 | endothelial cell migration | 69/1121 | 283/18614 | 3.70E-24 | 1.24E-21 | 8.18E-22 | 69 |
| GT | GO:0003007 | heart morphogenesis | 64/1121 | 262/18614 | 1.55E-22 | 4.90E-20 | 3.22E-20 | 64 |
| GT | GO:0050900 | leukocyte migration | 79/1121 | 393/18614 | 9.05E-22 | 2.71E-19 | 1.78E-19 | 79 |
| GT | GO:0032963 | collagen metabolic process | 40/1121 | 108/18614 | 9.81E-22 | 2.78E-19 | 1.83E-19 | 40 |
| GT | GO:0060485 | mesenchyme development | 70/1121 | 320/18614 | 1.40E-21 | 3.78E-19 | 2.48E-19 | 70 |
| GT | GO:0009913 | epidermal cell differentiation | 59/1121 | 240/18614 | 5.13E-21 | 1.31E-18 | 8.65E-19 | 59 |
| GT | GO:0061041 | regulation of wound healing | 44/1121 | 137/18614 | 6.49E-21 | 1.56E-18 | 1.03E-18 | 44 |
| GT | GO:0060326 | cell chemotaxis | 68/1121 | 312/18614 | 6.69E-21 | 1.56E-18 | 1.03E-18 | 68 |
| GT | GO:1903034 | regulation of response to wounding | 49/1121 | 170/18614 | 7.52E-21 | 1.69E-18 | 1.11E-18 | 49 |
| GT | GO:0045766 | positive regulation of angiogenesis | 51/1121 | 185/18614 | 1.07E-20 | 2.21E-18 | 1.45E-18 | 51 |

| | | | | | | | | |
|---|---|---|---|---|---|---|---|---|
| GT | GO:1904018 | positive regulation of vasculature development | 51/1121 | 185/18614 | 1.07E-20 | 2.21E-18 | 1.45E-18 | 51 |
| GT | GO:0061448 | connective tissue development | 63/1121 | 275/18614 | 1.23E-20 | 2.46E-18 | 1.62E-18 | 63 |
| GT | GO:0007160 | cell-matrix adhesion | 58/1121 | 239/18614 | 2.19E-20 | 4.20E-18 | 2.76E-18 | 58 |
| GT | GO:0030216 | keratinocyte differentiation | 49/1121 | 174/18614 | 2.27E-20 | 4.20E-18 | 2.76E-18 | 49 |
| GT | GO:0048762 | mesenchymal cell differentiation | 60/1121 | 255/18614 | 2.52E-20 | 4.52E-18 | 2.97E-18 | 60 |
| GT | GO:0034329 | cell junction assembly | 82/1121 | 444/18614 | 4.07E-20 | 7.06E-18 | 4.65E-18 | 82 |
| GT | GO:0001837 | epithelial to mesenchymal transition | 48/1121 | 172/18614 | 8.43E-20 | 1.42E-17 | 9.33E-18 | 48 |
| GT | GO:0010632 | regulation of epithelial cell migration | 64/1121 | 295/18614 | 1.25E-19 | 2.03E-17 | 1.34E-17 | 64 |
| GT | GO:0051216 | cartilage development | 52/1121 | 203/18614 | 1.60E-19 | 2.54E-17 | 1.67E-17 | 52 |
| GT | GO:0010810 | regulation of cell-substrate adhesion | 54/1121 | 222/18614 | 4.08E-19 | 6.28E-17 | 4.13E-17 | 54 |
| GT | GO:0052547 | regulation of peptidase activity | 78/1121 | 425/18614 | 5.11E-19 | 7.60E-17 | 5.00E-17 | 78 |
| GT | GO:0030199 | collagen fibril organization | 29/1121 | 64/18614 | 5.23E-19 | 7.60E-17 | 5.00E-17 | 29 |
| GT | GO:0002685 | regulation of leukocyte migration | 54/1121 | 227/18614 | 1.20E-18 | 1.70E-16 | 1.12E-16 | 54 |
| GT | GO:0048732 | gland development | 79/1121 | 441/18614 | 1.34E-18 | 1.85E-16 | 1.22E-16 | 79 |
| GT | GO:0003158 | endothelium development | 41/1121 | 137/18614 | 2.56E-18 | 3.45E-16 | 2.27E-16 | 41 |
| GT | GO:0071559 | response to transforming growth factor beta | 59/1121 | 280/18614 | 1.48E-17 | 1.94E-15 | 1.28E-15 | 59 |
| GT | GO:0050679 | positive regulation of epithelial cell proliferation | 52/1121 | 225/18614 | 2.01E-17 | 2.57E-15 | 1.69E-15 | 52 |
| GT | GO:0097529 | myeloid leukocyte migration | 54/1121 | 241/18614 | 2.07E-17 | 2.60E-15 | 1.71E-15 | 54 |
| GT | GO:0045446 | endothelial cell differentiation | 37/1121 | 119/18614 | 2.84E-17 | 3.47E-15 | 2.28E-15 | 37 |
| GT | GO:0040013 | negative regulation of locomotion | 75/1121 | 427/18614 | 3.23E-17 | 3.80E-15 | 2.50E-15 | 75 |
| GT | GO:2000146 | negative regulation of cell motility | 71/1121 | 390/18614 | 3.24E-17 | 3.80E-15 | 2.50E-15 | 71 |
| GT | GO:0003206 | cardiac chamber morphogenesis | 38/1121 | 126/18614 | 3.34E-17 | 3.83E-15 | 2.52E-15 | 38 |
| GT | GO:0072001 | renal system development | 63/1121 | 320/18614 | 4.03E-17 | 4.52E-15 | 2.97E-15 | 63 |
| GT | GO:0030336 | negative regulation of cell migration | 69/1121 | 375/18614 | 5.16E-17 | 5.67E-15 | 3.73E-15 | 69 |
| GT | GO:0071560 | cellular response to transforming growth factor beta stimulus | 57/1121 | 274/18614 | 9.76E-17 | 1.05E-14 | 6.91E-15 | 57 |
| GT | GO:0090287 | regulation of cellular response to growth factor stimulus | 65/1121 | 345/18614 | 1.26E-16 | 1.33E-14 | 8.76E-15 | 65 |

| | | | | | | | | |
|---|---|---|---|---|---|---|---|---|
| GT | GO:0061138 | morphogenesis of a branching epithelium | 46/1121 | 188/18614 | 1.39E-16 | 1.44E-14 | 9.47E-15 | 46 |
| GT | GO:0042692 | muscle cell differentiation | 72/1121 | 410/18614 | 1.44E-16 | 1.46E-14 | 9.60E-15 | 72 |
| GT | GO:0001570 | vasculogenesis | 30/1121 | 83/18614 | 2.68E-16 | 2.68E-14 | 1.76E-14 | 30 |
| GT | GO:0060537 | muscle tissue development | 73/1121 | 426/18614 | 3.43E-16 | 3.35E-14 | 2.21E-14 | 73 |
| GT | GO:0001503 | ossification | 73/1121 | 429/18614 | 5.04E-16 | 4.85E-14 | 3.19E-14 | 73 |
| GT | GO:0001822 | kidney development | 60/1121 | 310/18614 | 5.28E-16 | 4.98E-14 | 3.28E-14 | 60 |
| GT | GO:0001763 | morphogenesis of a branching structure | 47/1121 | 203/18614 | 6.59E-16 | 6.12E-14 | 4.02E-14 | 47 |
| GT | GO:0048844 | artery morphogenesis | 29/1121 | 80/18614 | 7.71E-16 | 7.03E-14 | 4.63E-14 | 29 |
| GT | GO:0030856 | regulation of epithelial cell differentiation | 42/1121 | 166/18614 | 8.17E-16 | 7.33E-14 | 4.82E-14 | 42 |
| GT | GO:0031099 | regeneration | 45/1121 | 190/18614 | 1.11E-15 | 9.81E-14 | 6.45E-14 | 45 |
| GT | GO:0007229 | integrin-mediated signaling pathway | 34/1121 | 112/18614 | 1.24E-15 | 1.08E-13 | 7.10E-14 | 34 |
| GT | GO:0002687 | positive regulation of leukocyte migration | 39/1121 | 147/18614 | 1.58E-15 | 1.35E-13 | 8.89E-14 | 39 |
| GT | GO:0010594 | regulation of endothelial cell migration | 50/1121 | 232/18614 | 1.73E-15 | 1.45E-13 | 9.55E-14 | 50 |
| GT | GO:0001704 | formation of primary germ layer | 36/1121 | 127/18614 | 2.02E-15 | 1.67E-13 | 1.10E-13 | 36 |
| GT | GO:0002064 | epithelial cell development | 47/1121 | 210/18614 | 2.66E-15 | 2.17E-13 | 1.42E-13 | 47 |
| GT | GO:0050921 | positive regulation of chemotaxis | 38/1121 | 143/18614 | 3.45E-15 | 2.77E-13 | 1.82E-13 | 38 |
| GT | GO:0060840 | artery development | 33/1121 | 109/18614 | 3.57E-15 | 2.82E-13 | 1.86E-13 | 33 |
| GT | GO:0007178 | transmembrane receptor protein serine/threonine kinase signaling pathway | 68/1121 | 398/18614 | 4.26E-15 | 3.32E-13 | 2.18E-13 | 68 |
| GT | GO:0003151 | outflow tract morphogenesis | 28/1121 | 82/18614 | 1.43E-14 | 1.10E-12 | 7.21E-13 | 28 |
| GT | GO:0010466 | negative regulation of peptidase activity | 49/1121 | 236/18614 | 1.55E-14 | 1.18E-12 | 7.73E-13 | 49 |
| GT | GO:0003205 | cardiac chamber development | 40/1121 | 166/18614 | 2.34E-14 | 1.75E-12 | 1.15E-12 | 40 |
| GT | GO:0007044 | cell-substrate junction assembly | 30/1121 | 96/18614 | 2.48E-14 | 1.83E-12 | 1.20E-12 | 30 |
| GT | GO:0010712 | regulation of collagen metabolic process | 21/1121 | 46/18614 | 3.23E-14 | 2.35E-12 | 1.55E-12 | 21 |
| GT | GO:0033002 | muscle cell proliferation | 50/1121 | 249/18614 | 3.33E-14 | 2.39E-12 | 1.57E-12 | 50 |
| GT | GO:0032964 | collagen biosynthetic process | 22/1121 | 51/18614 | 3.40E-14 | 2.41E-12 | 1.58E-12 | 22 |
| GT | GO:0007596 | blood coagulation | 47/1121 | 224/18614 | 3.54E-14 | 2.48E-12 | 1.63E-12 | 47 |
| GT | GO:0030595 | leukocyte chemotaxis | 49/1121 | 241/18614 | 3.65E-14 | 2.52E-12 | 1.65E-12 | 49 |
| GT | GO:0014706 | striated muscle tissue development | 51/1121 | 259/18614 | 4.17E-14 | 2.84E-12 | 1.87E-12 | 51 |

| | | | | | | | | | |
|---|---|---|---|---|---|---|---|---|---|
| GT | GO:0085029 | extracellular matrix assembly | 20/1121 | 42/18614 | 4.81E-14 | 3.24E-12 | 2.13E-12 | 20 |
| GT | GO:0071674 | mononuclear cell migration | 44/1121 | 202/18614 | 5.78E-14 | 3.84E-12 | 2.52E-12 | 44 |
| GT | GO:0033627 | cell adhesion mediated by integrin | 28/1121 | 87/18614 | 7.86E-14 | 5.16E-12 | 3.39E-12 | 28 |
| GT | GO:0010718 | positive regulation of epithelial to mesenchymal transition | 23/1121 | 58/18614 | 7.97E-14 | 5.17E-12 | 3.40E-12 | 23 |
| GT | GO:0060317 | cardiac epithelial to mesenchymal transition | 18/1121 | 34/18614 | 8.35E-14 | 5.34E-12 | 3.51E-12 | 18 |
| GT | GO:0050817 | coagulation | 47/1121 | 229/18614 | 8.44E-14 | 5.34E-12 | 3.51E-12 | 47 |
| GT | GO:0048568 | embryonic organ development | 71/1121 | 453/18614 | 9.32E-14 | 5.83E-12 | 3.84E-12 | 71 |
| GT | GO:0007599 | hemostasis | 47/1121 | 230/18614 | 1.00E-13 | 6.10E-12 | 4.01E-12 | 47 |
| GT | GO:0050920 | regulation of chemotaxis | 47/1121 | 230/18614 | 1.00E-13 | 6.10E-12 | 4.01E-12 | 47 |
| GT | GO:0098773 | skin epidermis development | 33/1121 | 121/18614 | 1.01E-13 | 6.10E-12 | 4.01E-12 | 33 |
| GT | GO:0030193 | regulation of blood coagulation | 25/1121 | 70/18614 | 1.13E-13 | 6.78E-12 | 4.46E-12 | 25 |
| GT | GO:0071675 | regulation of mononuclear cell migration | 33/1121 | 122/18614 | 1.30E-13 | 7.71E-12 | 5.07E-12 | 33 |
| GT | GO:0070371 | ERK1 and ERK2 cascade | 59/1121 | 341/18614 | 1.65E-13 | 9.67E-12 | 6.36E-12 | 59 |
| GT | GO:0022612 | gland morphogenesis | 33/1121 | 123/18614 | 1.68E-13 | 9.73E-12 | 6.40E-12 | 33 |
| PT | GO:0002443 | leukocyte mediated immunity | 52/215 | 401/18614 | 9.90E-40 | 3.22E-36 | 2.24E-36 | 52 |
| PT | GO:0002449 | lymphocyte mediated immunity | 42/215 | 300/18614 | 2.18E-33 | 3.54E-30 | 2.47E-30 | 42 |
| PT | GO:0002697 | regulation of immune effector process | 44/215 | 384/18614 | 3.54E-31 | 3.83E-28 | 2.67E-28 | 44 |
| PT | GO:0050867 | positive regulation of cell activation | 44/215 | 394/18614 | 1.07E-30 | 8.68E-28 | 6.05E-28 | 44 |
| PT | GO:0002696 | positive regulation of leukocyte activation | 43/215 | 377/18614 | 2.22E-30 | 1.44E-27 | 1.00E-27 | 43 |
| PT | GO:0002699 | positive regulation of immune effector process | 37/215 | 267/18614 | 2.94E-29 | 1.59E-26 | 1.11E-26 | 37 |
| PT | GO:0001819 | positive regulation of cytokine production | 46/215 | 489/18614 | 8.43E-29 | 3.91E-26 | 2.72E-26 | 46 |
| PT | GO:0050863 | regulation of T cell activation | 40/215 | 377/18614 | 4.88E-27 | 1.95E-24 | 1.36E-24 | 40 |
| PT | GO:1903037 | regulation of leukocyte cell-cell adhesion | 40/215 | 378/18614 | 5.40E-27 | 1.95E-24 | 1.36E-24 | 40 |
| PT | GO:0051251 | positive regulation of lymphocyte activation | 37/215 | 327/18614 | 4.95E-26 | 1.61E-23 | 1.12E-23 | 37 |

| | | | | | | | | |
|---|---|---|---|---|---|---|---|---|
| PT | GO:0002460 | adaptive immune response based on somatic recombination of immune receptors built from immunoglobulin superfamily domains | 36/215 | 311/18614 | 1.10E-25 | 3.24E-23 | 2.26E-23 | 36 |
| PT | GO:0022407 | regulation of cell-cell adhesion | 43/215 | 491/18614 | 1.20E-25 | 3.25E-23 | 2.26E-23 | 43 |
| PT | GO:0007159 | leukocyte cell-cell adhesion | 40/215 | 415/18614 | 1.95E-25 | 4.87E-23 | 3.39E-23 | 40 |
| PT | GO:0002366 | leukocyte activation involved in immune response | 35/215 | 296/18614 | 2.67E-25 | 6.19E-23 | 4.31E-23 | 35 |
| PT | GO:0022409 | positive regulation of cell-cell adhesion | 36/215 | 321/18614 | 3.34E-25 | 7.23E-23 | 5.04E-23 | 36 |
| PT | GO:0002263 | cell activation involved in immune response | 35/215 | 300/18614 | 4.23E-25 | 8.58E-23 | 5.97E-23 | 35 |
| PT | GO:1903039 | positive regulation of leukocyte cell-cell adhesion | 33/215 | 273/18614 | 3.40E-24 | 6.50E-22 | 4.53E-22 | 33 |
| PT | GO:0050870 | positive regulation of T cell activation | 30/215 | 249/18614 | 5.16E-22 | 9.32E-20 | 6.49E-20 | 30 |
| PT | GO:0045785 | positive regulation of cell adhesion | 38/215 | 482/18614 | 4.51E-21 | 7.71E-19 | 5.37E-19 | 38 |
| PT | GO:0002703 | regulation of leukocyte mediated immunity | 29/215 | 246/18614 | 4.90E-21 | 7.96E-19 | 5.54E-19 | 29 |
| PT | GO:0070663 | regulation of leukocyte proliferation | 29/215 | 268/18614 | 5.48E-20 | 8.47E-18 | 5.90E-18 | 29 |
| PT | GO:0016064 | immunoglobulin mediated immune response | 23/215 | 145/18614 | 7.70E-20 | 1.14E-17 | 7.92E-18 | 23 |
| PT | GO:0002253 | activation of immune response | 37/215 | 495/18614 | 9.58E-20 | 1.35E-17 | 9.42E-18 | 37 |
| PT | GO:0002705 | positive regulation of leukocyte mediated immunity | 23/215 | 148/18614 | 1.24E-19 | 1.62E-17 | 1.13E-17 | 23 |
| PT | GO:0019724 | B cell mediated immunity | 23/215 | 148/18614 | 1.24E-19 | 1.62E-17 | 1.13E-17 | 23 |
| PT | GO:0002440 | production of molecular mediator of immune response | 31/215 | 328/18614 | 1.43E-19 | 1.78E-17 | 1.24E-17 | 31 |
| PT | GO:0002764 | immune response-regulating signaling pathway | 35/215 | 450/18614 | 2.95E-19 | 3.55E-17 | 2.47E-17 | 35 |
| PT | GO:0030098 | lymphocyte differentiation | 34/215 | 422/18614 | 3.35E-19 | 3.89E-17 | 2.71E-17 | 34 |
| PT | GO:0050670 | regulation of lymphocyte proliferation | 27/215 | 239/18614 | 3.71E-19 | 4.15E-17 | 2.89E-17 | 27 |
| PT | GO:0032944 | regulation of mononuclear cell proliferation | 27/215 | 243/18614 | 5.73E-19 | 6.20E-17 | 4.32E-17 | 27 |

| | | | | | | | | |
|---|---|---|---|---|---|---|---|---|
| PT | GO:1903131 | mononuclear cell differentiation | 35/215 | 474/18614 | 1.55E-18 | 1.62E-16 | 1.13E-16 | 35 |
| PT | GO:0002285 | lymphocyte activation involved in immune response | 25/215 | 208/18614 | 1.83E-18 | 1.86E-16 | 1.29E-16 | 25 |
| PT | GO:0002274 | myeloid leukocyte activation | 26/215 | 240/18614 | 5.04E-18 | 4.96E-16 | 3.46E-16 | 26 |
| PT | GO:0001906 | cell killing | 24/215 | 197/18614 | 6.65E-18 | 6.36E-16 | 4.43E-16 | 24 |
| PT | GO:0070661 | leukocyte proliferation | 30/215 | 348/18614 | 7.68E-18 | 7.00E-16 | 4.88E-16 | 30 |
| PT | GO:0019886 | antigen processing and presentation of exogenous peptide antigen via MHC class II | 13/215 | 31/18614 | 7.76E-18 | 7.00E-16 | 4.88E-16 | 13 |
| PT | GO:0030217 | T cell differentiation | 28/215 | 300/18614 | 1.30E-17 | 1.14E-15 | 7.96E-16 | 28 |
| PT | GO:0002768 | immune response-regulating cell surface receptor signaling pathway | 29/215 | 329/18614 | 1.55E-17 | 1.33E-15 | 9.25E-16 | 29 |
| PT | GO:0002706 | regulation of lymphocyte mediated immunity | 23/215 | 184/18614 | 1.88E-17 | 1.56E-15 | 1.09E-15 | 23 |
| PT | GO:0046651 | lymphocyte proliferation | 28/215 | 306/18614 | 2.19E-17 | 1.78E-15 | 1.24E-15 | 28 |
| PT | GO:0002757 | immune response-activating signaling pathway | 32/215 | 423/18614 | 2.61E-17 | 2.07E-15 | 1.44E-15 | 32 |
| PT | GO:0032943 | mononuclear cell proliferation | 28/215 | 313/18614 | 3.97E-17 | 3.07E-15 | 2.14E-15 | 28 |
| PT | GO:0002495 | antigen processing and presentation of peptide antigen via MHC class II | 13/215 | 35/18614 | 5.34E-17 | 4.03E-15 | 2.81E-15 | 13 |
| PT | GO:0030595 | leukocyte chemotaxis | 25/215 | 241/18614 | 6.48E-17 | 4.78E-15 | 3.33E-15 | 25 |
| PT | GO:0031349 | positive regulation of defense response | 32/215 | 441/18614 | 8.75E-17 | 6.31E-15 | 4.40E-15 | 32 |
| PT | GO:0002504 | antigen processing and presentation of peptide or polysaccharide antigen via MHC class II | 13/215 | 37/18614 | 1.26E-16 | 8.91E-15 | 6.21E-15 | 13 |
| PT | GO:0050900 | leukocyte migration | 30/215 | 393/18614 | 2.21E-16 | 1.52E-14 | 1.06E-14 | 30 |
| PT | GO:0002381 | immunoglobulin production involved in immunoglobulin-mediated immune response | 16/215 | 75/18614 | 2.68E-16 | 1.81E-14 | 1.26E-14 | 16 |

| | | | | | | | | |
|---|---|---|---|---|---|---|---|---|
| PT | GO:0050671 | positive regulation of lymphocyte proliferation | 20/215 | 145/18614 | 3.63E-16 | 2.40E-14 | 1.67E-14 | 20 |
| PT | GO:0002708 | positive regulation of lymphocyte mediated immunity | 19/215 | 126/18614 | 3.72E-16 | 2.42E-14 | 1.68E-14 | 19 |
| PT | GO:0070665 | positive regulation of leukocyte proliferation | 21/215 | 166/18614 | 3.89E-16 | 2.48E-14 | 1.72E-14 | 21 |
| PT | GO:0002478 | antigen processing and presentation of exogenous peptide antigen | 13/215 | 40/18614 | 4.13E-16 | 2.58E-14 | 1.80E-14 | 13 |
| PT | GO:0031341 | regulation of cell killing | 18/215 | 110/18614 | 5.12E-16 | 3.14E-14 | 2.18E-14 | 18 |
| PT | GO:0032946 | positive regulation of mononuclear cell proliferation | 20/215 | 148/18614 | 5.45E-16 | 3.28E-14 | 2.29E-14 | 20 |
| PT | GO:0002429 | immune response-activating cell surface receptor signaling pathway | 26/215 | 302/18614 | 1.44E-15 | 8.52E-14 | 5.93E-14 | 26 |
| PT | GO:0001909 | leukocyte mediated cytotoxicity | 19/215 | 139/18614 | 2.43E-15 | 1.41E-13 | 9.80E-14 | 19 |
| PT | GO:0042129 | regulation of T cell proliferation | 21/215 | 183/18614 | 2.89E-15 | 1.64E-13 | 1.15E-13 | 21 |
| PT | GO:0042098 | T cell proliferation | 22/215 | 213/18614 | 5.69E-15 | 3.19E-13 | 2.22E-13 | 22 |
| PT | GO:0019884 | antigen processing and presentation of exogenous antigen | 13/215 | 49/18614 | 8.24E-15 | 4.53E-13 | 3.16E-13 | 13 |
| PT | GO:0071621 | granulocyte chemotaxis | 18/215 | 131/18614 | 1.23E-14 | 6.66E-13 | 4.64E-13 | 18 |
| PT | GO:0019882 | antigen processing and presentation | 17/215 | 114/18614 | 1.70E-14 | 9.05E-13 | 6.30E-13 | 17 |
| PT | GO:0002702 | positive regulation of production of molecular mediator of immune response | 18/215 | 134/18614 | 1.85E-14 | 9.67E-13 | 6.74E-13 | 18 |
| PT | GO:0097530 | granulocyte migration | 19/215 | 156/18614 | 2.12E-14 | 1.09E-12 | 7.61E-13 | 19 |
| PT | GO:0060326 | cell chemotaxis | 25/215 | 312/18614 | 2.74E-14 | 1.39E-12 | 9.70E-13 | 25 |
| PT | GO:0002399 | MHC class II protein complex assembly | 9/215 | 16/18614 | 3.30E-14 | 1.62E-12 | 1.13E-12 | 9 |
| PT | GO:0002503 | peptide antigen assembly with MHC class II protein complex | 9/215 | 16/18614 | 3.30E-14 | 1.62E-12 | 1.13E-12 | 9 |
| PT | GO:1902105 | regulation of leukocyte differentiation | 25/215 | 319/18614 | 4.56E-14 | 2.21E-12 | 1.54E-12 | 25 |

| | | | | | | | | |
|---|---|---|---|---|---|---|---|---|
| PT | GO:0002700 | regulation of production of molecular mediator of immune response | 20/215 | 189/18614 | 6.50E-14 | 3.10E-12 | 2.16E-12 | 20 |
| PT | GO:0042102 | positive regulation of T cell proliferation | 16/215 | 105/18614 | 7.25E-14 | 3.41E-12 | 2.38E-12 | 16 |
| PT | GO:0097529 | myeloid leukocyte migration | 22/215 | 241/18614 | 7.44E-14 | 3.45E-12 | 2.40E-12 | 22 |
| PT | GO:0030593 | neutrophil chemotaxis | 16/215 | 107/18614 | 9.83E-14 | 4.50E-12 | 3.13E-12 | 16 |
| PT | GO:0031343 | positive regulation of cell killing | 14/215 | 74/18614 | 1.22E-13 | 5.43E-12 | 3.78E-12 | 14 |
| PT | GO:1990266 | neutrophil migration | 17/215 | 128/18614 | 1.22E-13 | 5.43E-12 | 3.78E-12 | 17 |
| ST | GO:0050867 | positive regulation of cell activation | 45/258 | 394/18614 | 3.10E-28 | 1.02E-24 | 7.92E-25 | 45 |
| ST | GO:0002696 | positive regulation of leukocyte activation | 43/258 | 377/18614 | 5.79E-27 | 7.73E-24 | 6.00E-24 | 43 |
| ST | GO:0002443 | leukocyte mediated immunity | 44/258 | 401/18614 | 7.05E-27 | 7.73E-24 | 6.00E-24 | 44 |
| ST | GO:0002768 | immune response-regulating cell surface receptor signaling pathway | 40/258 | 329/18614 | 3.56E-26 | 2.93E-23 | 2.27E-23 | 40 |
| ST | GO:0002764 | immune response-regulating signaling pathway | 44/258 | 450/18614 | 8.43E-25 | 5.55E-22 | 4.30E-22 | 44 |
| ST | GO:0002429 | immune response-activating cell surface receptor signaling pathway | 37/258 | 302/18614 | 2.28E-24 | 1.25E-21 | 9.69E-22 | 37 |
| ST | GO:0001819 | positive regulation of cytokine production | 45/258 | 489/18614 | 2.93E-24 | 1.38E-21 | 1.07E-21 | 45 |
| ST | GO:0051251 | positive regulation of lymphocyte activation | 38/258 | 327/18614 | 3.59E-24 | 1.48E-21 | 1.15E-21 | 38 |
| ST | GO:0002253 | activation of immune response | 45/258 | 495/18614 | 4.86E-24 | 1.77E-21 | 1.38E-21 | 45 |
| ST | GO:0007159 | leukocyte cell-cell adhesion | 41/258 | 415/18614 | 2.64E-23 | 8.70E-21 | 6.75E-21 | 41 |
| ST | GO:0002757 | immune response-activating signaling pathway | 41/258 | 423/18614 | 5.48E-23 | 1.64E-20 | 1.27E-20 | 41 |
| ST | GO:0002449 | lymphocyte mediated immunity | 35/258 | 300/18614 | 2.27E-22 | 6.23E-20 | 4.84E-20 | 35 |
| ST | GO:1903037 | regulation of leukocyte cell-cell adhesion | 38/258 | 378/18614 | 6.64E-22 | 1.68E-19 | 1.30E-19 | 38 |
| ST | GO:1903039 | positive regulation of leukocyte cell-cell adhesion | 33/258 | 273/18614 | 1.27E-21 | 2.98E-19 | 2.31E-19 | 33 |
| ST | GO:0022407 | regulation of cell-cell adhesion | 42/258 | 491/18614 | 1.92E-21 | 4.21E-19 | 3.27E-19 | 42 |
| ST | GO:0022409 | positive regulation of cell-cell adhesion | 35/258 | 321/18614 | 2.18E-21 | 4.48E-19 | 3.47E-19 | 35 |

| | | | | | | | | |
|---|---|---|---|---|---|---|---|---|
| ST | GO:0050863 | regulation of T cell activation | 37/258 | 377/18614 | 5.53E-21 | 1.07E-18 | 8.31E-19 | 37 |
| ST | GO:0002697 | regulation of immune effector process | 37/258 | 384/18614 | 1.04E-20 | 1.90E-18 | 1.48E-18 | 37 |
| ST | GO:0050870 | positive regulation of T cell activation | 30/258 | 249/18614 | 1.08E-19 | 1.87E-17 | 1.45E-17 | 30 |
| ST | GO:0030595 | leukocyte chemotaxis | 29/258 | 241/18614 | 4.71E-19 | 7.75E-17 | 6.01E-17 | 29 |
| ST | GO:0002460 | adaptive immune response based on somatic recombination of immune receptors built from immunoglobulin superfamily domains | 31/258 | 311/18614 | 6.66E-18 | 1.04E-15 | 8.10E-16 | 31 |
| ST | GO:0002699 | positive regulation of immune effector process | 29/258 | 267/18614 | 8.02E-18 | 1.20E-15 | 9.30E-16 | 29 |
| ST | GO:0002366 | leukocyte activation involved in immune response | 30/258 | 296/18614 | 1.48E-17 | 2.11E-15 | 1.64E-15 | 30 |
| ST | GO:0045785 | positive regulation of cell adhesion | 37/258 | 482/18614 | 2.13E-17 | 2.83E-15 | 2.20E-15 | 37 |
| ST | GO:0002263 | cell activation involved in immune response | 30/258 | 300/18614 | 2.15E-17 | 2.83E-15 | 2.20E-15 | 30 |
| ST | GO:0030593 | neutrophil chemotaxis | 20/258 | 107/18614 | 2.67E-17 | 3.38E-15 | 2.62E-15 | 20 |
| ST | GO:0050851 | antigen receptor-mediated signaling pathway | 25/258 | 201/18614 | 6.50E-17 | 7.93E-15 | 6.15E-15 | 25 |
| ST | GO:0019886 | antigen processing and presentation of exogenous peptide antigen via MHC class II | 13/258 | 31/18614 | 8.51E-17 | 9.99E-15 | 7.76E-15 | 13 |
| ST | GO:0019884 | antigen processing and presentation of exogenous antigen | 15/258 | 49/18614 | 9.21E-17 | 1.05E-14 | 8.11E-15 | 15 |
| ST | GO:0071621 | granulocyte chemotaxis | 21/258 | 131/18614 | 1.13E-16 | 1.21E-14 | 9.41E-15 | 21 |
| ST | GO:0002478 | antigen processing and presentation of exogenous peptide antigen | 14/258 | 40/18614 | 1.14E-16 | 1.21E-14 | 9.41E-15 | 14 |
| ST | GO:0031349 | positive regulation of defense response | 34/258 | 441/18614 | 4.16E-16 | 4.28E-14 | 3.32E-14 | 34 |
| ST | GO:0060326 | cell chemotaxis | 29/258 | 312/18614 | 5.38E-16 | 5.37E-14 | 4.17E-14 | 29 |
| ST | GO:0002495 | antigen processing and presentation of peptide antigen via MHC class II | 13/258 | 35/18614 | 5.80E-16 | 5.61E-14 | 4.35E-14 | 13 |
| ST | GO:0070663 | regulation of leukocyte proliferation | 27/258 | 268/18614 | 7.78E-16 | 7.31E-14 | 5.68E-14 | 27 |

| | | | | | | | | |
|---|---|---|---|---|---|---|---|---|
| ST | GO:1990266 | neutrophil migration | 20/258 | 128/18614 | 1.03E-15 | 9.43E-14 | 7.32E-14 | 20 |
| ST | GO:0002504 | antigen processing and presentation of peptide or polysaccharide antigen via MHC class II | 13/258 | 37/18614 | 1.36E-15 | 1.21E-13 | 9.42E-14 | 13 |
| ST | GO:0050670 | regulation of lymphocyte proliferation | 25/258 | 239/18614 | 4.01E-15 | 3.47E-13 | 2.70E-13 | 25 |
| ST | GO:0097530 | granulocyte migration | 21/258 | 156/18614 | 4.32E-15 | 3.64E-13 | 2.83E-13 | 21 |
| ST | GO:0001906 | cell killing | 23/258 | 197/18614 | 4.84E-15 | 3.98E-13 | 3.09E-13 | 23 |
| ST | GO:0032944 | regulation of mononuclear cell proliferation | 25/258 | 243/18614 | 5.92E-15 | 4.75E-13 | 3.68E-13 | 25 |
| ST | GO:0050853 | B cell receptor signaling pathway | 16/258 | 77/18614 | 7.49E-15 | 5.87E-13 | 4.55E-13 | 16 |
| ST | GO:0016064 | immunoglobulin mediated immune response | 20/258 | 145/18614 | 1.23E-14 | 9.41E-13 | 7.30E-13 | 20 |
| ST | GO:0002440 | production of molecular mediator of immune response | 28/258 | 328/18614 | 1.55E-14 | 1.16E-12 | 8.97E-13 | 28 |
| ST | GO:0019724 | B cell mediated immunity | 20/258 | 148/18614 | 1.84E-14 | 1.34E-12 | 1.04E-12 | 20 |
| ST | GO:0019882 | antigen processing and presentation | 18/258 | 114/18614 | 2.41E-14 | 1.72E-12 | 1.34E-12 | 18 |
| ST | GO:0050900 | leukocyte migration | 30/258 | 393/18614 | 3.31E-14 | 2.32E-12 | 1.80E-12 | 30 |
| ST | GO:0097529 | myeloid leukocyte migration | 24/258 | 241/18614 | 4.38E-14 | 3.00E-12 | 2.33E-12 | 24 |
| ST | GO:0070661 | leukocyte proliferation | 28/258 | 348/18614 | 6.82E-14 | 4.46E-12 | 3.46E-12 | 28 |
| ST | GO:0001909 | leukocyte mediated cytotoxicity | 19/258 | 139/18614 | 6.83E-14 | 4.46E-12 | 3.46E-12 | 19 |
| ST | GO:0002703 | regulation of leukocyte mediated immunity | 24/258 | 246/18614 | 6.92E-14 | 4.46E-12 | 3.46E-12 | 24 |
| ST | GO:0002285 | lymphocyte activation involved in immune response | 22/258 | 208/18614 | 1.52E-13 | 9.62E-12 | 7.47E-12 | 22 |
| ST | GO:0046651 | lymphocyte proliferation | 26/258 | 306/18614 | 1.58E-13 | 9.82E-12 | 7.62E-12 | 26 |
